# Coiled-Coil Proteins Facilitated the Functional Expansion of the Centrosome

**DOI:** 10.1371/journal.pcbi.1003657

**Published:** 2014-06-05

**Authors:** Michael Kuhn, Anthony A. Hyman, Andreas Beyer

**Affiliations:** 1 Biotechnology Center, TU Dresden, Dresden, Germany; 2 Max Planck Institute of Molecular Cell Biology and Genetics, Dresden, Germany; 3 University of Cologne, Cologne, Germany; Princeton University, United States of America

## Abstract

Repurposing existing proteins for new cellular functions is recognized as a main mechanism of evolutionary innovation, but its role in organelle evolution is unclear. Here, we explore the mechanisms that led to the evolution of the centrosome, an ancestral eukaryotic organelle that expanded its functional repertoire through the course of evolution. We developed a refined sequence alignment technique that is more sensitive to coiled coil proteins, which are abundant in the centrosome. For proteins with high coiled-coil content, our algorithm identified 17% more reciprocal best hits than BLAST. Analyzing 108 eukaryotic genomes, we traced the evolutionary history of centrosome proteins. In order to assess how these proteins formed the centrosome and adopted new functions, we computationally emulated evolution by iteratively removing the most recently evolved proteins from the centrosomal protein interaction network. Coiled-coil proteins that first appeared in the animal–fungi ancestor act as scaffolds and recruit ancestral eukaryotic proteins such as kinases and phosphatases to the centrosome. This process created a signaling hub that is crucial for multicellular development. Our results demonstrate how ancient proteins can be co-opted to different cellular localizations, thereby becoming involved in novel functions.

## Author Summary

The centrosome helps cells to divide, and is important for the development of animals. It has its evolutionary origins in the basal body, which was present in the last common ancestor of all eukaryotes. Here, we study how the evolution of novel proteins helped the formation of the centrosome. Coiled-coil proteins are important for the function of the centrosome. But, they have repeating patterns that can confuse existing methods for finding related proteins. We refined these methods by adjusting for the special properties of the coiled-coil regions. This enabled us to find more distant relatives of centrosomal proteins. We then tested how novel proteins affect the protein interaction network of the centrosome. We did this by removing the most novel proteins step by step. At each stage, we observed how the remaining proteins are connected to the centriole, the core of the centrosome. We found that coiled-coil proteins that first occurred in the ancestor of fungi and animals help to recruit older proteins. By being recruited to the centrosome, these older proteins acquired new functions. We thus now have a clearer picture of how the centrosome became such an important part of animal cells.

## Introduction

The transition from unicellularity to multicellularity occurred independently in many eukaryotic lineages [1]. Compared to other multicellular organisms, animals stand out with respect to the high number of cell types [1], [2], the complexity of body plans, and the necessity of cell migration for development [3]. The evolution of these traits in animals was facilitated by the properties of the cell membrane, cell motility and cell division: Animals retained the ancestral modes of cell motility (amoeboid and flagellar motility) and a soft cell membrane. In plants and fungi, a rigid cell wall evolved, restricting cell motility. However, we know little about how the organelles required for cell division, motility and organization evolved with increasing complexity of animals.

The cytoskeleton is a key player behind cell organization, motility and division [4], [5]. One of the coordinators of the cytoskeleton is the microtubule-organizing center (MTOC). In most animals and many other eukaryotes, the basal body or centrosome is the MTOC. Basal bodies are ancestral to eukaryotes and are composed of paired centrioles [6]. In animals, the centrosome is composed of the centrioles and the surrounding pericentriolar material (PCM). The centrosome acts as a signaling hub [7], [8], coordinating many functions of multicellular organisms, for example cell migration or maintenance of cell orientation during division [9]–[11]. Fungi and slime molds independently evolved spindle pole bodies, while a great diversity of acentriolar MTOC exists for plants [12]. The expansion and loss of functions of the centrosome throughout evolution can thus be traced in the different eukaryotic lineages. Recognized mechanisms for the evolution of novel functions include the expansion of gene families through duplication, the emergence of coordinated regulation, and *de novo* gene birth [13]–[16]. Another mechanism is the rewiring of molecular signaling networks, thereby utilizing existing molecular components of the cell in new contexts [17], [18]. It is, however, unclear which mechanisms are involved in the evolution of whole organelles.

We used the centrosome to study the interplay between macroscopic and cellular evolution: The animal centrosome has an extended PCM compared to other species, but its core dates back to the last eukaryotic common ancestor [6]. Previous studies, which focused on the evolution of centrioles, have indicated that many components of the animal centrosome first appeared in animals [19], [20]. However, many of these apparently novel proteins are coiled-coil proteins. Helices that form coiled coils have a regular, repeating pattern of hydrophobic, charged, and hydrophilic amino acids [21]. This so-called heptad repeat of seven residues causes traditional sequence alignment algorithms to overestimate the significance of the observed sequence similarity, leading to incorrectly predicted homologous proteins. In other words, apparently similar proteins can obscure the actual homologous protein. Previous studies have therefore masked coiled-coil sequences from similarity searches [22], which increases the risk of missing true orthologs. Thus, a complete survey of the evolutionary history of centrosomal proteins needs to be based on a refined alignment of coiled-coil proteins. We have developed a novel method that takes the restricted space of possible substitutions into account, thereby greatly reducing the amount of false positives. Our method distinguishes between coiled-coil and “normal” regions and treats residues in different positions on the heptad repeat differently. We performed an all-against-all alignment for proteins from 108 eukaryotic species to predict orthologs [23]. Combining our predictions with those based on BLAST searches, we created a dataset of protein families that can be used to pinpoint the establishment of a protein family during evolution, similar to phylostratigraphy [24]. We correlated the appearance of protein families, protein networks, and functions to infer important contributors to the evolution of the animal centrosome (see Fig. S1 for an overview).

## Results

### An improved algorithm for aligning coiled-coil proteins

A recent addition to BLAST is composition-based adjustment of substitution matrices [25]. This approach modifies the substitution matrices by adjusting the substitution scores to reflect the amino acid composition observed in the query and database proteins, while keeping the matrices' entropy constant. Proteins with biased sequence composition occur in certain protein families or even in whole organisms with AT- or GC-rich genomes. To some extent, compositional matrix adjustment can also account for the biased composition of coiled-coil proteins, e.g. by observing the abundance of hydrophilic residues and decreasing the substitution scores. Nonetheless, compositional matrix adjustment does not take into account the regular repeat structure of coiled-coil proteins, and it also can not deal with the different compositional biases found within and outside coiled-coil domains.

**Figure 1:**
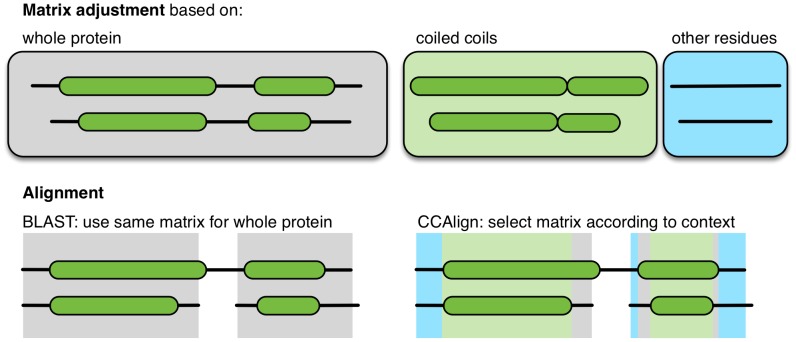
The new alignment algorithm. BLAST adjusts substitution matrices based on the complete sequences of the pair of proteins to be aligned. CCAlign computes adjusted, specific matrices for the coiled-coil domains and the non-coiled-coil parts of the proteins. For alignment, different substitution matrices are then selected, according the coiled-coil state of the individual residues under consideration.

There have been various approaches to create specialized substitution matrices for parts of proteins with different compositions, for example for trans-membrane proteins [26] or to distinguish hydrophobic and non-hydrophobic regions [27]. Initially, we also created specialized substitution matrices for coiled-coil and non-coiled-coil regions of the proteins. While this approach outperformed the standard BLOSUM matrix (data not shown), it did not perform better than BLAST with compositional matrix adjustment. We therefore developed two algorithms that take the sequence properties of the coiled-coil structure into account. In both algorithms, coiled coils are predicted using MultiCoil2 [28] using a cutoff probability of 0.8. In the first algorithm, the pair of proteins to be aligned is divided into coiled-coil and non-coiled-coil subsequences. These subsequences are then used to perform compositional matrix adjustment. Then, a full Smith-Waterman-Gotoh alignment is performed. The substitution matrix is chosen according to the coiled-coil status of the considered residues (Fig. 1, see Methods for details). In the second algorithm, the coiled-coil domains are further sub-divided into three parts: the hydrophobic interface, the charged intermediate residues and the hydrophilic outside.

**Figure 2:**
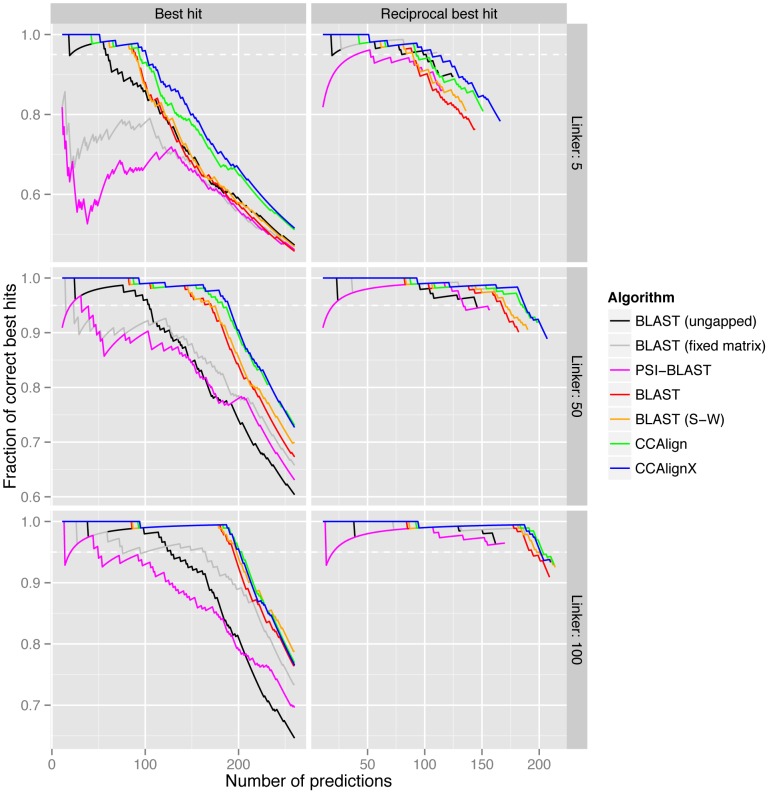
Benchmarking of algorithms. Alignment algorithms were applied to coiled-coil proteins from a set of manually annotated yeast orthologs. For each protein, it was then determined if the best hit was actually annotated as orthologous. Non-coiled-coil parts of the proteins were reduced to respective linker lengths (shown on the right of the panels) to increase the difficulty of detecting the true homolog. CCAlign and CCAlignX, the two algorithms that take the sequence properties of coiled-coils into account, perform better than standard algorithms. Dashed horizontal lines indicate a 5% false discovery rate. For clarity, data for less than ten predictions is omitted. (S-W: Smith-Waterman algorithm).

In order to compare different implementations against each other, we have adopted a benchmarking scheme based on the manually annotated KOGs (eukaryotic orthologous groups) and a separate set of *S. pombe*–*S. cerevisiae* homologs [29], [30]. To simulate proteins with a high fraction of coiled-coils, we took the coiled-coil proteins from these two datasets and created artificial proteins by excising predicted coiled-coil domains together with a linker of variable length. The subsequences (i.e., all instances of linker–coiled-coil–linker) are concatenated and used for the alignment. We first used the KOG database to set parameter choices for our alignment algorithms (Fig. S2). Then, we compared the performance of our algorithms to several BLAST options: standard BLAST, standard BLAST with full Smith-Waterman alignment (not optimized and therefore very slow), PSI-BLAST, and, for reference, BLAST without compositional matrix adjustment and ungapped BLAST (which also employs a fixed substitution matrix). The results from the yeast dataset (Fig. 2) are consistent with those from the KOG dataset (Fig. S3): CCAlign and CCAlignX perform better than the other methods. For example, over the yeast benchmark set with linker length 50 and a 5% FDR, CCAlign correctly identifies 67.7% of all possible reciprocal best hits, CCAlignX identifies 68.4% and BLAST 61.1%. BLAST can also be run with a complete Smith-Waterman algorithm that has not been optimized for speed and can thus not be used for large-scale applications. With this configuration, BLAST identifies 62.3% of all possible reciprocal best hits. PSI-BLAST performs much worse, identifying only 48.8% (at three iterations, and a correspondingly increased runtime). Building position-specific scoring matrix (PSSMs), the hallmark of the iterative approach taken by PSI-BLAST, is partly incompatible with compositional matrix adjustment. The PSSMs pick up on the strong sequence signal of the coiled-coil domains, and therefore detect many false hits. In essence, PSI-BLAST violates the adage “when you find yourself in a hole, stop digging” by iteratively building a profile to detect coiled-coils, but not sequence similarity that is due to homology.

Outside the benchmark set, we compared the performance of BLAST, CCAlign and CCAlignX on the complete set of proteins in our dataset of 108 species. For proteins with at least 20% of their residues in coiled-coils, CCAlign detected 11.1% more reciprocal best hits than BLAST (CCAlignX: 11.3%, bitscore cutoff: 30). A large part of this improvement is due to the full Smith-Waterman alignment done even for non-coiled-coil proteins, where performance increased by 10.7% for CCAlign (CCAlignX: 10.5%). The impact of the adjusted substitution matrices becomes more apparent for proteins with higher coiled-coil content: For proteins with at least 50% coiled-coil residues, performance increased by 13.1% for CCAlign and 13.5% for CCAlignX. The peak performance increase is reached at 17.3% for both methods at coiled-coil contents of at least 86% and 81%, respectively.

### Predicted homologs of centrosomal proteins

**Figure 3:**
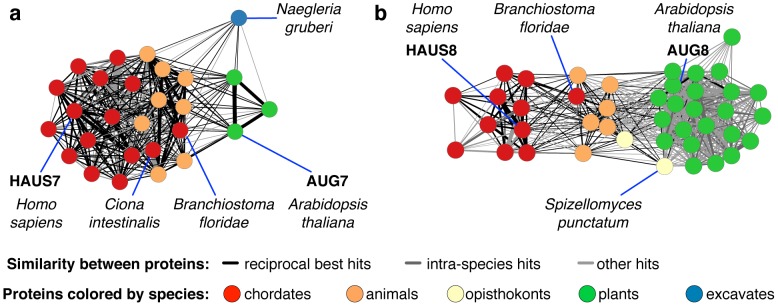
Distant homologs of centrosomal proteins. Contrary to previous reports, HAUS7 and AUG7 (**a**), and HAUS8 and AUG8 (**b**), are in fact homologs, linked by proteins from other species. Edges connect pairs of proteins that can be aligned successfully, both across and within species. Edge width corresponds to the strength (bit score) of the alignment. In both cases, chordate proteins are most similar to the human protein. Proteins from other animals, in turn, are both similar to plant and chordate proteins, and hence make it possible to detect the homology between the human and plant proteins.

**Figure 4:**
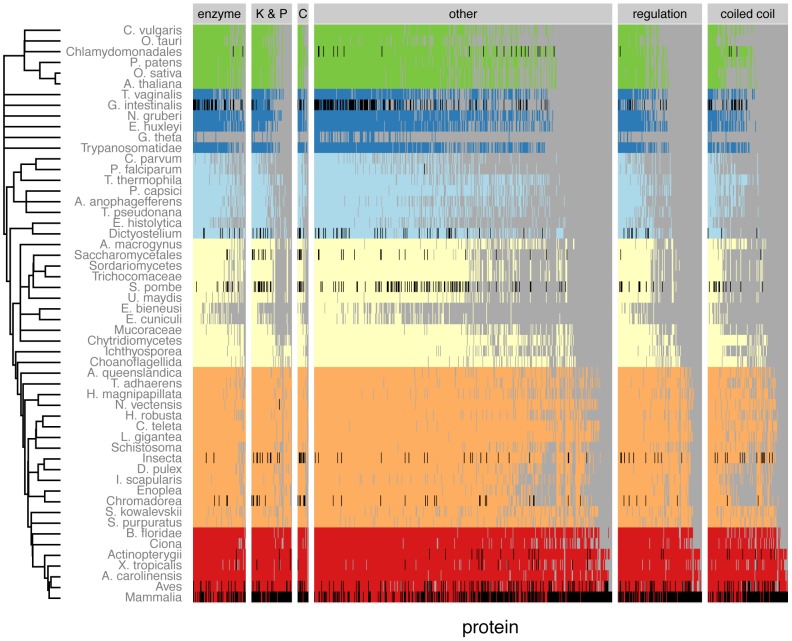
Phylogenetic distribution of protein families. The phylogenetic distribution is shown for all protein families that have been annotated as centrosome, basal body or SPB components, and which also occur in mammals. Cells in black denote species where the centrosomal location is known, colored cells indicated that a homolog has been found. Species of the same taxonomic class have been combined. (K & P: kinase & phosphatases, C: cytoskeleton and motor proteins)

We used CCAlign, CCAlignX and BLAST to perform all-against-all alignments for proteins from 108 eukaryotic species. Combining evidence from all three alignments, we predicted homologs for all proteins (see Methods) regardless of coiled-coil content or centrosome localization, yielding a database of orthologous proteins that can be accessed at http://projects.biotec.tu-dresden.de/orthologs/. To validate our predictions, we searched the literature for homologs of centrosomal proteins that have previously been uncovered by manual investigation of individual proteins. We confirmed, for example, the homology between CDK5RAP2 (CEP215, fly: cnn) and the *S. pombe* proteins mto1 and pcp1 [31], [32], or the occurrence of homologs of DISC1 in plants [33]. For many other proteins (see Table S1), we found homologs beyond what has been shown in previous small- or large-scale studies. For example, our approach identified homologs of AKAP9, PCNT and PCM1 in fungi. (See Dataset S1 for multiple sequence alignments.) We found homologs of the *C. elegans* protein spd-5 in filarial nematodes (e.g. *Brugia malayi*) and *Ascaris suum*. Spd-5 is essential for centrosome formation in *C. elegans*, but had previously only been reported in *Caenorhabditis* species. A recent study uncovered two novel subunits of the *Arabidopsis thaliana* augmin complex, AUG7 and AUG8, and reported these two proteins to be unique to plants [34]. Based on more species and on a more suitable alignment method, we could show that the human augmin subunits HAUS7 and HAUS8 are in fact homologous to AUG7 and AUG8, respectively (Fig. 3). Overall, we found exactly 1000 protein families that are centrosome-related in any species (see Tables S2 and S3 for an overview). Of these, 897 protein families also occur in human, 610 of which are known to be of centrosomal localization in humans or other mammals (Fig. 4).

### Evolutionary age of centrosomal protein families

**Figure 5:**
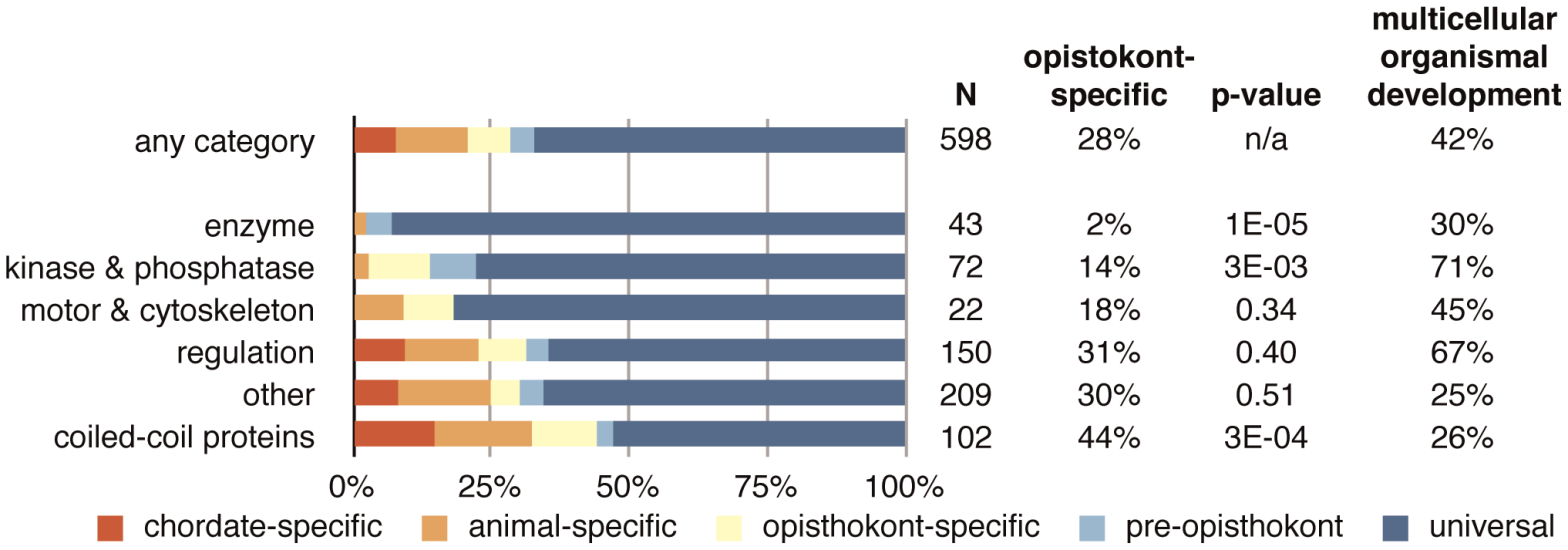
Involvement of new proteins in biological processes. The age of centrosome protein families varies by protein category. Scaffolds are of more recent origin than other proteins, while kinases, phosphatases, and enzymes are more ancient. Despite this, kinases and phosphatases are of great importance for multicellular organismal development. The p-value is calculated with a binomial test comparing the fraction of proteins that first occurred in opisthokonts to the overall fraction of 28%. The last column shows the fraction of proteins that is annotated with the GO term “multicellular organismal development.”

In each protein family, we can now check the species distribution and for example find the species that is most distantly related to human. Thus, we found that most centrosomal protein families are more ancient than other human proteins: 72% of all centrosomal proteins first appeared before the fungi–animal (opisthokont) ancestor (Fig. 5), compared to 46% for all human protein families. For further analysis on the evolution of centrosome functions, we divided proteins into categories based on their known function in human (see Methods, Fig. 5). Of the proteins without annotation, we designated proteins with at least 20% of their residues in coiled-coils as “coiled-coil proteins.” In other words, coiled-coil proteins that have an annotated function (e.g. motor proteins) were grouped with corresponding functional class. Note that the choice of the coiled-coil threshold does not affect the outcome of the network analyses, as explained below.

For proteins that occur in mammals, we determined their evolutionary age by looking for the most distantly related species. Thus, a protein also found in *Ciona* is chordate-specific, while a protein also found in chytrid fungi is opisthokont-specific. Our analysis revealed that coiled-coil proteins are on average significantly younger than most centrosome proteins, whereas kinases, and phosphatases are older (Fig. 5). For example, 86% of kinases and phosphatase families first appeared before the opisthokont ancestor, compared to only 56% for coiled-coil proteins. Many coiled-coil proteins thus evolved earlier than previously thought, but are still younger than other centrosomal proteins.

Interestingly, 76% of all centrosomal kinase families have been shown to be involved in multicellular organismal development, compared to 55% of all kinases. We found similar patterns for other functional categories (Fig. S4). Thus, centrosome-associated kinases and other regulatory proteins (which are often ancient) are enriched for functions related to multi-cellularity. In the (unicellular) eukaryote ancestor, kinases cannot have had these functions, and therefore must have acquired them later through other mechanisms. The novel functions are, for example, reflected in an increased PCM size (Table S4).

### Evolution of the centrosome

To gain insight into the mechanisms by which ancient proteins were recruited to centrosomes, we developed a strategy for simulating the changes in the protein interaction network of the centrosome during evolution. We first assembled the centrosome's protein–protein interaction network, to identify those interactions that contribute to the structural backbone. This network was then used to emulate the course of evolution by iteratively removing the most recently evolved proteins. Using this method, we generated an approximation for the structure of the interaction network at different stages of evolution. In particular, we tested how many of the remaining proteins lost or changed their mode of recruitment to the centrosome. We do not have enough data on the basal body of the eukaryote ancestor to quantify the impact of protein losses. However, the evident increase in complexity and size from the basal body to the animal centrosome make it likely that the gain of proteins played a much larger role than the loss. The impact of the loss of proteins is, however, apparent both in fungi and in plants. In these lineages, the basal body became obsolete, leading to the loss of many centriole proteins.

We first extracted the interaction network from the STRING database [35], using interactions derived from experimental evidence and text-mining (see Methods). This network contains both direct and indirect interactions and represents the functional interactions of centrosome proteins, even if there is not enough detailed structural data for the complete centrosome. The evolutionary and structural core of the centrosome is the centriole, serving as a seed for the formation of the PCM. In particular, two conserved proteins, which were already present in the eukaryote ancestor, are important for centriole formation: SASS6 serves as a template for the barrel-shaped centriole [36]. SAS-4, the *C. elegans* ortholog of CENPJ, controls centrosome size [37]. Its *Drosophila* ortholog, sas-4, has been shown to recruit cytoplasmic complexes of PCM proteins [38]. These PCM proteins can then in turn recruit other centrosomal proteins, forming a protein–protein interaction network that is dominated by a dense core of regulatory proteins, kinases, phosphatases, and their substrates (Fig. S5). In the periphery of this signaling hub, ciliary proteins, the gamma-tubulin ring complex and the augmin complex are situated in less connected areas of the network.

**Figure 6:**
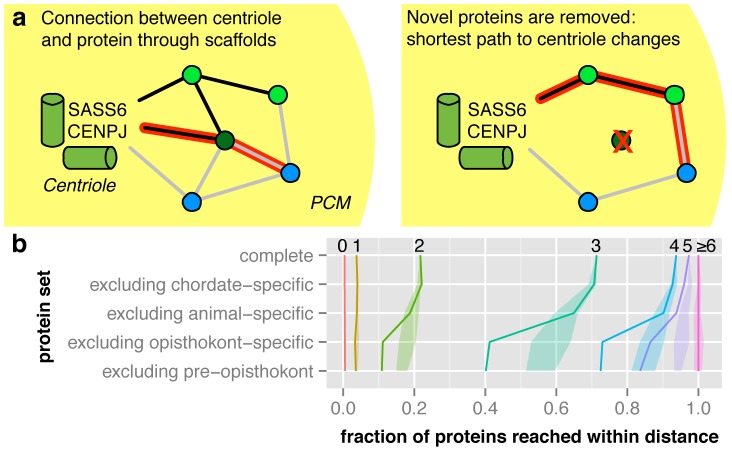
Scaffold proteins that first occur in opisthokonts recruit other proteins to the centrosome. a The length of the shortest path between the centriole (proteins SASS6 and CENPJ) and every protein is calculated, traversing only scaffold and uncategorized proteins. To simulate the topological changes of this network throughout the evolution of the centrosome, novel proteins are iteratively removed. **b** Removing opisthokont-specific proteins leads to a significant increase in shortest path lengths. For each shortest path length, we show the fraction of proteins that can be reached within that distance when iteratively removing the most recently evolved proteins. The shaded area corresponds to the second and third quartile of 10000 randomizations.

The distance in the network between a given protein and the centriole can be calculated as the number of steps along the shortest path between the protein and the centriolar proteins SASS6 and CENPJ. We simplified the analysis by using proteins of the structural backbone as intermediate nodes (i.e. only coiled-coil and uncategorized proteins, see Methods and Fig. 6a). These proteins are likely to mediate interactions of kinases and other proteins with the PCM. To emulate the evolution of the centrosome, we iteratively removed the most recently evolved proteins from the network (see Methods, Fig. 6b). Our analysis showed that in the complete interaction network, 71% of all proteins were reachable within three steps from the centriole. This fraction stayed virtually constant when chordate- and animal-specific proteins were removed. However, when opisthokont-specific proteins were removed, only 41% of all proteins remained reachable within three steps. We ascertained the significance by shuffling the proteins' evolutionary origin 10,000 times. Proteins were divided into five bins according to their coiled-coil content and evolutionary age was shuffled within each of these bins to control for possible biases in the detected ages of proteins. Indeed, we found that the actual change in the fraction of proteins within three steps of the centriole is highly significant (p = 0.007). This means that when coiled-coil proteins that first occurred in opisthokonts are removed, older proteins that had been connected to the centriole by these coiled-coil proteins lose their “main connection” to the centriole. Thus, the number of steps between the centriole and these proteins increases. No further change was observed when only proteins present in the eukaryote ancestor were considered. Thus, structural backbone proteins that evolved prior to, or shortly after, the last common ancestor of fungi and animals are crucial for the formation of the interaction network of the centrosome. In fact, acentriolar MTOCs in mouse oocytes and *Drosophila* mutants still contain PCM coiled-coil proteins like PCNT and Cnn [39], [40]. We further distinguished the evolution of the PCM compared to a reduced network of basal body, cilium and centriole proteins (Fig. S6). The influence of removing coiled-coil proteins on the basal body network is much smaller, consistent with the observation that the PCM is a more recent development. We evaluated the robustness of the model by testing the impact of removing other protein categories, and found that coiled-coil proteins are unique in their effect on the network (see Suppl. Text and Table S5) and that changes in the thresholds for the STRING network and the coiled-coil content do not affect the conclusions (Fig. S7).

### Robustness of the results

The findings presented above rely on the accuracy of the predicted evolutionary age. In order to evaluate the sensitivity of our conclusions on the improved alignment method, we repeated the above analysis using standard BLAST. Whereas most results remained qualitatively similar, coiled-coil proteins were predicted to be older: using our specialized alignment procedure 44% of the coiled-coil proteins were opisthokont-specific or younger, compared to 41% with BLAST (Fig. S8a). Just using BLAST may overestimate the homology between proteins due to high sequence similarity in coiled-coil regions, which leads to an elevated grouping of distant proteins in joint families. When emulating the evolution of the centrosome (Fig. S8b), the change when removing opisthokont-specific proteins was not significant (Suppl. Table S5), but the removal of coiled-coil proteins still has the strongest effect on the network. Removing pre-opisthokont proteins (i.e. keeping only universal proteins), however, led to a significant change (p = 0.029 for coiled-coil and uncategorized proteins, compared to p = 0.017 using all three alignment methods).

Our work showed that coiled-coil proteins are in fact older than previously thought. Taken to the extreme, one could also postulate that all coiled-coil proteins occurred in the eukaryote ancestor. To further corroborate the robustness of our findings we conducted additional tests that are independent of the evolutionary age of protein families: we assessed the importance of nodes in the network according to the number of shortest paths that pass through the nodes (Text S1, Fig. S9 and Table S6). This test exclusively depends on the topology of the network. Proteins with the largest number of shortest paths passing through them were designated as bottlenecks (cut-off: top 5% or 20 shortest paths). We had to control for the influence of hubs, i.e. proteins with very many interaction partners, which are more likely to be part in shortest paths (cut-off: top 5% or 39 edges). Among the proteins that are not hubs, coiled-coil proteins have the greatest enrichment among bottlenecks (P = 0.11, one-sided Fisher's exact test). When we constructed a network where edges leading to hubs receive a larger distance score (i.e. are less likely to be part of a shortest path, see Suppl. Text), we again find that coiled-coil proteins have the strongest enrichment of bottlenecks (P = 0.03). We furthermore assessed the validity of our model's evolutionary explanations in a framework formulated by Scriven [41] (see Text S1 and Fig. S10).

### Functional implications

**Figure 7:**
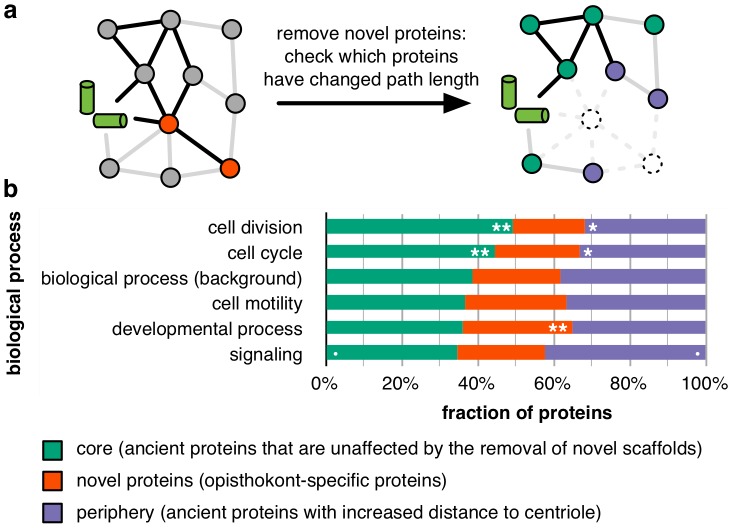
Differences in recruitment correspond to functional differences. a When novel proteins are removed from the interaction network, proteins that become more distant to the centrioles can be identified. **b** Five high-level GO terms corresponding to the centrosome's functions were tested for enrichment among core, novel or peripheral proteins. Core proteins, which remain at the same distance, are more involved in cell cycle and division than in processes important for animals such as development and signaling. Cut-offs for significance levels (calculated with Fisher's exact test): ** 0.01, * 0.05, • 0.1.

Based on these observations, we divided centrosome proteins into three classes (Fig. 7a) according to their change in network distance upon removal of proteins that first occur in opisthokonts: core proteins (that keep their distance to the centrioles, e.g. AURKA, polo-like kinases and the HAUS complex), peripheral proteins (whose distance increases, e.g. CEP290, DISC1 and the BBSome), and novel proteins (that first occur in opisthokonts proteins, e.g. PCNT, AKAP9 and PCM1). Although this classification is only a rough representation of the order of recruitment, we found significant functional differences when testing the main functions carried out by the centrosome (Fig. 7b). Universal functions such as cell cycle and division have a significantly higher fraction of core proteins. In contrast, processes that have become more important for animals compared to their unicellular ancestors are carried out by a lower fraction of core proteins. For example, signaling proteins are enriched (p = 0.07, using Fisher's exact test) in the periphery, underlining that the centrosome became increasingly important as a signaling hub at the transition to multi-cellularity. Thus, the core centrosome reflects the ancestral functions related to individual cells, whereas the novel and expansion proteins are involved in newer functions related to multi-cellularity. An exemplar member of the periphery is the kinase GSK3B, a member of a large family of signaling proteins [42]. In *S. pombe*, it is involved in cytokinesis and bipolar cell growth [43], [44], while in *S. cerevisiae* it has been implicated in stress response [45]. It takes part in cell differentiation in *Dictyostelium* [23], [46]. In animals, the protein localizes to the centrosome and takes part in many developmental processes, for example neural development: It targets centrosomal proteins such as ninein and the asymmetric inheritance of the centrosome with the mother centriole may be a mechanism of regulating neuronal differentiation [47].

## Discussion

In this work, we have extended the space of known homologs of centrosomal proteins over previous studies, finding that proteins that were previously thought to be restricted to animals first occurred earlier in evolution. Nonetheless, the fast divergence of coiled-coil proteins leads to gaps in the matrix of homologs (e.g. within nematodes and insects, see Fig. 4). In the future, comparative structural approaches might make it possible to bridge these gaps, although high-throughput expression of centrosomal proteins is difficult [48]. While our predictions showed that many centrosomal coiled-coil proteins are older than previously thought, future method development, and structural and functional assays may further increase age estimates of these proteins. However, as shown above, the role of coiled-coil proteins on the evolution of the centrosome interaction network could also be demonstrated without assumptions on the age of the proteins.

Coiled-coil proteins at the centrosome have long been recognized to be part of a “centromatrix” or centrosomal matrix [49]–[52]. Indeed, many previous studies have shown that centrosomal coiled-coil proteins function as scaffolds for the recruitment of other proteins (Table S1). This is a general trend: In the Gene Ontology, 44 human proteins are annotated as protein complex scaffolds, 12 of which have coiled-coil sections. This fraction of 27% is a significant enrichment over the background rate of coiled-coil proteins, which is 12% of all human proteins (p-value: 0.005 using a one-sided Fisher's exact test). Hence, proteins with a high fraction of residues in coiled-coils are more likely to be scaffold proteins than proteins without coiled-coil residues. Here, we were able to show that many centrosomal coiled-coil proteins indeed act as scaffold proteins, providing a mechanism for earlier observations. For example, based on the analysis of only five animal species and the non-centrosomal budding yeast as an out-group, Nido et al. observed an increase in coiled-coil content and disorder in centrosomal proteins towards mammals [53]. They linked this increase in coiled-coil content to the ability of these proteins to change their physical properties upon post-translational modification. Consistent with these findings, we discovered an increased fraction of residues in disordered regions for opisthokont-specific proteins. When comparing core and peripheral proteins (Fig. 7a), we found no change in disorder for coiled-coil proteins. However, the fraction of residues in disordered regions is increased in the core for regulatory proteins (p-value 0.054, two-sided Kolmogorov-Smirnov test) and for uncategorized proteins (p-value 0.01), but not for the other functional categories. In general, centrosomal proteins have higher disorder content than non-centrosomal proteins [53]. The further division among centrosomal proteins that we observe is consistent with our finding that coiled-coil proteins facilitated the evolution of the centrosome by acting as scaffolds that recruit ancient proteins for novel functions: Peripheral proteins may have been recruited to the centrosome more recently, and are thus more similar in their disorder content to non-centrosomal proteins.

It was possible for us to quantify the impact of scaffolds on the evolution of the centrosome because of its organization: it has a small proteinaceous core (the centriole) that is used by the cell to control centrosome number and localization. Other non-membrane-bounded organelles are recruited by DNA (e.g. kinetochores and nucleoli) or not controlled in number (e.g. P granules). In the case of membrane-bounded organelles, membranes provide large surfaces for the organization of protein complexes. Thus, additional modes of recruitment of proteins may have acted in those organelles. Nonetheless, we found that coiled-coil proteins are also significantly more novel than other proteins in the case of kinetochores and the Golgi apparatus (Fig. S11). Thus, the recruitment of molecular functions through coiled-coil scaffolds may not be restricted to the centrosome.

## Methods

### Refined alignment of coiled-coil proteins

There are three elements that distinguish our approach to previous algorithms: (1) Scoring matrices are adjusted to take the coiled coils' amino acid composition into account. (2) Scores from different positions in the heptad repeat are weighted. (3) A correct alignment of the heptad repeat between the aligned proteins is rewarded (for the second algorithm only).

**Figure 8:**
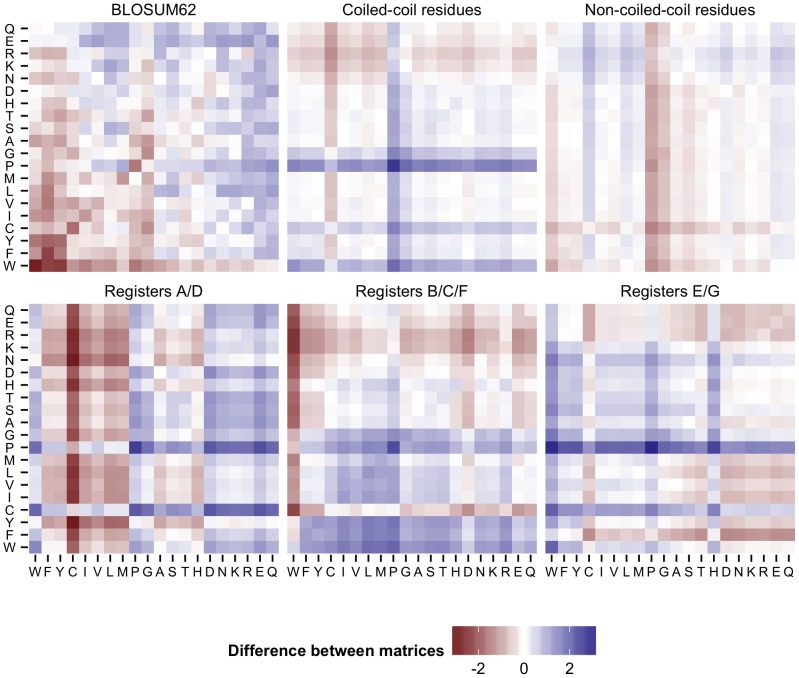
Substitution matrix adjustment. Substitution matrices were generated for the alignment of the human protein CDK5RAP2 with its fly homolog cnn. The difference between the respective matrix and the adjusted matrix for the whole proteins is shown. This reference matrix is the one that BLAST uses for the alignment of these two proteins. When the difference is above zero (blue), then the two amino acids are more rare in the considered part of the protein. For example, proline is known to disrupt helices and hence also coiled-coils, therefore the scores for proline in the coiled-coil matrix are higher. In the substitution matrix for the hydrophobic interface (registers A/D), hydrophobic residues are more common, resulting in lower scores.

In the first algorithm (“CCAlign”), proteins are divided into coiled-coil and non-coiled-coil sections. The sequences of these two classes are concatenated separately, yielding two artificial sequences per protein. To align a pair of proteins, composition-adjusted substitution matrices are calculated for the pair of coiled-coil sections, for the pair of non-coiled-coil sections, and for the complete proteins. The calculation uses BLAST's matrix adjustment algorithm, made accessible by a modified version that does not perform alignments, but only computes and returns the adjusted matrix (Fig. 8). For alignment, we use a modified Smith-Waterman-Gotoh algorithm [54], [55], based on the open-source implementation JAligner. In a Smith-Waterman alignment of two proteins, all possible pairs of residues are considered. In traditional algorithms, the same substitution matrix (e.g. BLOSUM62) is used for all pairs of residues. For this algorithm, the substitution matrix is chosen according to the status of the pair of residues under consideration: the coiled-coil substitution matrix is used when both residues are in a coiled coil. The non-coiled-coil matrix is used if none of the residues is in a coiled coil. In the mixed case, the substitution matrix based on the full-length proteins is used.

Intuitively, the registers of the heptad repeat should contain varying amounts of phylogenetic signal, i.e. be more or less informative with regard to the potential homology of two proteins. To estimate this, we extracted coiled-coil residues from the Blocks database [56], a set of highly conserved sequences that has been used to generate the BLOSUM substitution matrices. We derived sub-databases that correspond to either single registers of the heptad repeat, or groups of registers. Using the entropy of the substitution matrices as a proxy for phylogenetic signal, we observe that the hydrophobic interface residues are more informative (entropy 0.45) than the intermediate residues (0.32) and the hydrophilic outside (0.28). However, all positions of the heptad repeat are less informative than the background (BLOSUM62, 0.70). We benchmarked different weighting schemes for the register-specific phylogenetic signal, with two degrees of freedom: (1) Entropies can be calculated for groups of registers (a/d, e/g, b/c/f) or for individual registers. (2) The entropies can be normalized by the entropy of BLOSUM62, or by the median coiled-coil entropy. Out of these schemes, normalizing group entropies with the median entropy proved to be most successful in the benchmark scheme (see below). Mathematically, the algorithm for determining the score for a pair of residues from the proteins sequences can be described in this way:
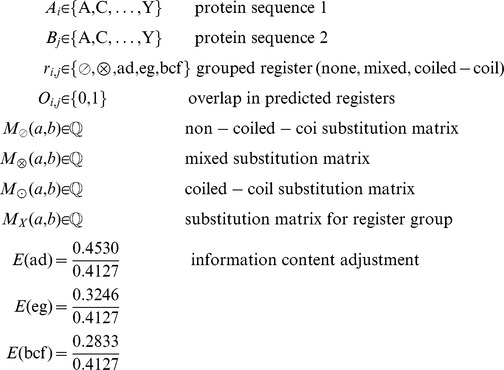


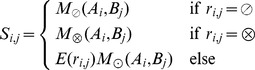
For the second algorithm (“CCAlignX”), the coiled-coil residues are further subdivided by their position in the heptad repeat: the hydrophobic interface (a, d), the hydrophilic outside (b, c, f) and the intermediate residues (e, g). Based on these groups, additional substitution matrices are computed. When two coiled-coil residues are considered for alignment, the matrix that corresponds to the register of the more confident MultiCoil2 prediction is used. For this algorithm, benchmarking indicates that adding another scoring mechanism yields better results: When the predicted coiled-coil registers overlap, an additional bonus score is awarded to the residue pair under consideration.
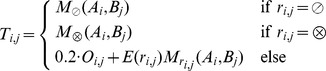
The approach of dividing proteins into regions of different evolutionary constraints could be applicable to other classes of proteins that contain regions with different evolutionary pressures and different amino acid compositions, like trans-membrane proteins. Software implementing the algorithms mentioned above is available from https://bitbucket.org/mkuhn/blast-matrix and https://bitbucket.org/mkuhn/ccalign.

### Prediction of orthologs

Genomes were gathered by extending eukaryotic genomes in the STRING 9 database [35] with a number of other genomes, yielding a total of 108 genomes (see Fig. S12 for a phylogenetic tree [57], [58]). For nematode genomes of interest where only nucleotide sequences were available, genes were predicted with Maker [59]. If a genome was predicted to have more than 50,000 genes, the genes were aligned against the UniProt database (using the metazoan UniRef90 dataset). All genes were then sorted by the bitscore of their top hit. Genes were retained if they were among top 50,000 hits or had a bitscore greater than 50.

An all-against-all protein alignment was performed in multiple steps: First, a speed-optimized Smith-Waterman alignment was computed using ParAlign [60]. Hits from the first step were then re-aligned using the coiled-coil aware alignment algorithms. (For non-coiled-coil proteins, this step adds compositional matrix adjustment.) As an optimization, only the top 50 hits of each protein in each other species were determined by the re-alignment. In addition to the coiled-coil aware alignments, the complete all-against-all alignment was performed with BLAST. Thus, three sets of alignments have been calculated: CCAlign, CCAlignX and BLAST. For each set, groups of homologous proteins are predicted using the eggNOG pipeline [23].

In order to trace common ancestry with more sensitivity, we modified the eggNOG pipeline to allow for more merging of similar groups in the last stages of the pipeline: The eggNOG pipeline first searches for triangles of proteins in different species that are reciprocal best hits (RBH) of each other, and adds other RBHs to these seed groups. Then, through several iterations, orthologous groups are joined (when they have many RBHs between each other) and split (when the set of proteins becomes too diverse). We have added a final step that used the diagnostic output of the last merging step, namely the set of OG pairs and their merging score. Applying a threshold for the score produced filtered set of OG pairs. When two OGs contained overlapping sets of species (and thus proteins that may be paralogous), we used a more stringent threshold to avoid merging paralogs. The filtered set of OG pairs was then converted into a graph. In decreasing order of scores, connected OGs were then combined into clusters. As a precaution to avoid indefinite growth of clusters, we imposed a restriction on the diameter of the cluster: for each pair of OGs in the cluster, the maximum allowed distance is four edges (i.e. there can be up to three OGs in between). With these modifications, we can detect more distant homologs, even in the case of greater sequence divergence.

As can be seen in Fig. 3, orthologous groups connect proteins through intermediate proteins. Within the original eggNOG pipeline, alignment positions are checked to avoid connecting non-homologous proteins through shared domains [61]. In the final merging step that we have added, the stringent cutoff to prevent merging of paralogs also serves as a precaution against such false positives. In our manual inspection of alignments, including those for HAUS7/8 (Fig. 3), we always found shared conserved regions between all orthologs except when additional truncated copies of the protein occurred in certain species along with the full-length protein.

To reduce false positives, predictions from BLAST, CCAlign and CCAlignX were then combined using a voting scheme: if at least two of the three methods agree that two proteins are homologous, then they are accepted to be homologs in the combined prediction (Fig. S13a). In some cases, however, individual proteins caused spurious links between unrelated groups of homologous proteins (Fig. S13b). To avoid these links, we determined the proteins' betweenness centrality for all groups of homologs (using the NetworkX package for Python). Proteins that generate spurious links have a high betweenness centrality, as many shortest paths between other proteins pass through them. These proteins are tentatively removed from the combined groups of homologs. If a link mediated by these proteins was spurious, then the group of homologs disintegrates into sub-groups. If the link was valid, then it will be backed up by other links, and the group does not disintegrate. The newly formed sub-groups are checked for spurious links in turn.

### Annotation of proteins

Known centrosome proteins were extracted from a variety of sources: Gene Ontology (GO) annotations [62], the MiCroKit database [63], and proteomic screens in mammals, *Giardia lambia* and *Chlamydomonas reinhardtii* [64]–[68]. Proteins were assigned to categories based on their GO annotation, InterPro domains [69], Enzyme Commission numbers [70], and limited manual annotation. Motors are assigned based on InterPro domains (dynein, kinesin, myosin). GO annotations are used for these classes: kinases (*protein kinase activity*), phosphatases (*phosphoprotein phosphatase activity*), cytoskeletal proteins (*structural constituent of cytoskeleton*), scaffolds (*protein complex scaffold*), regulators (*regulation of signal transduction*, *regulation of protein modification process*) and transcription factors (*sequence-specific DNA binding transcription factor activity*). As there were only seven transcription factors known to localize to the centrosome, we added these to the regulatory proteins. Proteins that have been assigned an Enzyme Commission number are assigned as enzymes. Lastly, proteins with at least 20% coiled-coil residues are also assigned as scaffolds. Thirty-five percent of centrosome proteins do not fit any of these categories and are designated as “other” proteins. The order in this paragraph reflects the priority of assignment of functions, e.g. ROCK1 (a kinase with a coiled-coil domain) has been annotated as a kinase, not a scaffold.

### Network analysis

A protein interaction network was extracted from the STRING 9 database using a confidence cutoff of 0.5. Only the “experiments” and “text-mining” channels were included. In particular, edges from the “database” channel were not included, as some manually annotated pathway databases contain the centrosome as one very large (unstructured) complex, which is undesirable for the present analysis.

In order to find traces of the expansion of the centrosome and its development into a signaling hub, we analyzed the role of scaffold proteins and their interactions with regulatory proteins in more detail. Only a subset of the interactions in the network belong to the structural backbone. For example, protein interactions involving kinases, phosphatases and regulatory proteins are likely to be transient interactions, whereas interactions mediated by scaffold and uncategorized proteins are more likely permanent physical interactions with higher specificity. This is also reflected by the number of interaction partners: scaffolds and uncategorized proteins have fewer interaction partners than other classes of proteins (Fig. S9). To capture the majority of permanent interactions, we designate scaffolds and uncategorized proteins as the structural backbone of the PCM.

To determine shortest paths within the protein interaction network, scaffold and uncategorized proteins were used as backbone nodes. Computationally, the network was represented as a directed graph, with directed edges going out from backbone nodes. Thus, non-backbone proteins such as kinases are sinks, i.e. they have only incoming edges. The NetworkX package for Python was then used.

### Analysis of disordered residues

We used DISOPRED2 [71] for predicting disordered regions. When a residue was predicted to be both in a coiled-coil domain and in a disordered region, we treated the residue as being not disordered.

## Supporting Information

Figure S1 **Overview of the pipeline.**
(PDF)

Figure S2 **Results of benchmarking.** For multiple parameter combinations, the fraction of correctly predicted homologous proteins is calculated. This fraction is compared to the reference fraction using only the BLOSUM62 matrix using the binomial test. Circled: actual parameter combinations used (left: CCAlignX, right: CCAlign). SW-BLAST: BLAST using the Smith-Waterman algorithm.
(PDF)

Figure S3 **Benchmarking of algorithms, based on the KOG database.** See Fig. 2 for full caption.
(PDF)

Figure S4 **Functions of centrosomal proteins.** The fraction of human protein families annotated for various processes is shown for centrosome specific proteins versus proteins of any localization. We investigated the role of centrosomes in four functions important for the animal organism: multicellular organismal development, cellular response to stimulus, cell cycle and cell motility. The centrosome is very important for these functions: compared to proteins of any localization, a significantly larger fraction of centrosomal proteins is involved with these functions. Pre-metazoan protein families are more important for multicellular organismal development than metazoan protein families. The same is true for cellular response to stimulus and cell motility.
(PDF)

Figure S5 **The protein interaction network of the centrosome.** Protein-protein interactions were extracted from the STRING 9 database (see Methods).
(TIFF)

Figure S6 **The centrosome's evolution compared to the basal body and PCM evolution.** For the basal body network, we combined proteins from the centriole, cilium and basal body. To study the evolution of the PCM, we ran the emulation procedure for the whole centrosome, but only consider shortest paths of proteins that are not part of the basal body network. For each shortest path length, we show the fraction of proteins that can be reached within that distance when iteratively removing the most recently evolved proteins. The shaded area corresponds to the second and third quartile of 10,000 randomizations, with p-values for a path length of three steps shown on the right.
(PDF)

Figure S7 **Exploration of different protein interaction networks.** P-values for the effect of removing proteins are shown for different STRING networks, score cutoffs and coiled-coil thresholds. When all channels from STRING are used, higher score cutoffs lead to a network dominated by database evidence, which tends to group the centrosome in one large complex. Using the combined experimental and text-mining channels, the p-value for removing opisthokont-specific scaffold and uncategorized proteins is below 0.05 in all but two cases. The experimental-only network is sparser and does not show significant effects. Changing the minimum fraction of coiled-coil residues when designating proteins as scaffolds does not impact the findings.
(PDF)

Figure S8 **Using only BLAST as alignment method.** (a) Using only BLAST to estimate the age of protein families makes coiled-coil proteins appear to be older (41% opisthokont-specific for BLAST vs. 44% for the combination of all three alignment methods). (b) As a consequence, only the removal of proteins that evolved after the last eukaryote ancestor leads to a significant change (at path length 3), although the trends are similar (see also Suppl. Table S5).
(PDF)

Figure S9 **Analysis of shortest paths.** For the complete network, the number of shortest paths that pass through a node is plotted against the degree (number of connections) of the node. The top 5% nodes by degree are hubs, the top 5% by number of shortest paths are bottlenecks. When multiple nodes have the same values, a small random offset is added to reduce over-plotting.
(PDF)

Figure S10 **Number of interactions per protein.** The degree of the proteins in the human centrosome protein interaction network is shown as a function of evolutionary age. Top: Proteins are divided into those that have been present in the eukaryote ancestor and those that evolved later. P-values have been computed with a permutation test (R package “exactRankTests”). Bottom: All considered clades are shown, along with the number of proteins that first appeared in this clade.
(PDF)

Figure S11 **Evolutionary age of coiled-coil proteins in different organelles.** For organelles as annotated in the Gene Ontology, the age distribution is shown for all proteins and for scaffold proteins.
(PDF)

Figure S12 **Phylogenetic tree of the 108 species whose genomes have been analyzed.**
(PDF)

Figure S13 **Illustration of the procedure to safeguard against spurious links between OGs.**
(PDF)

Table S1 **Fraction of coiled-coil residues, species distribution and function of centrosomal coiled-coil proteins.**
(DOCX)

Table S2 **Species distribution of centrosome proteins.** For all protein families, the species in which we identified homologous proteins are shown.
(XLSX)

Table S3 **Centrosomal proteins in model species.** Protein identifiers of homologous proteins are given for the species *Homo sapiens, Caenorhabditis elegans, Drosophila melanogaster, Saccharomyces cerevisiae, Schizosaccharomyces pombe, Dictyostelium discoideum, Giardia intestinalis* and *Arabidopsis thaliana*.
(XLSX)

Table S4 **Size of MTOC for different species.** PCM volume is computed by assuming a spherical centrosome with two cylindrical centrioles of length 0.4 µm and diameter 0.2 µm.
(DOCX)

Table S5 **Simulating centrosome evolution with different backbones.**
(DOCX)

Table S6 **Enrichment of bottlenecks in the centrosome interaction network.**
(DOCX)

Text S1 **Supplementary Information.** Includes the sections “Robustness of the model” and “Verification of the model.”
(DOCX)

Dataset S1 **Multiple-sequence alignments.** This file contains alignments for the protein families spd-5, AKAP9/PCNT, PCM1, HAUS7 and HAUS8 in FASTA format and as HTML pages with highlighted coiled-coil domains.
(ZIP)
