# Supplementary figures and images for: Coiled-Coil Proteins Facilitated the Functional Expansion of the Centrosome
Source: PLoS Comput Biol. 2014 Jun 5;10(6):e1003657. doi: 10.1371/journal.pcbi.1003657 (PMC4046923; doi:10.1371/journal.pcbi.1003657)

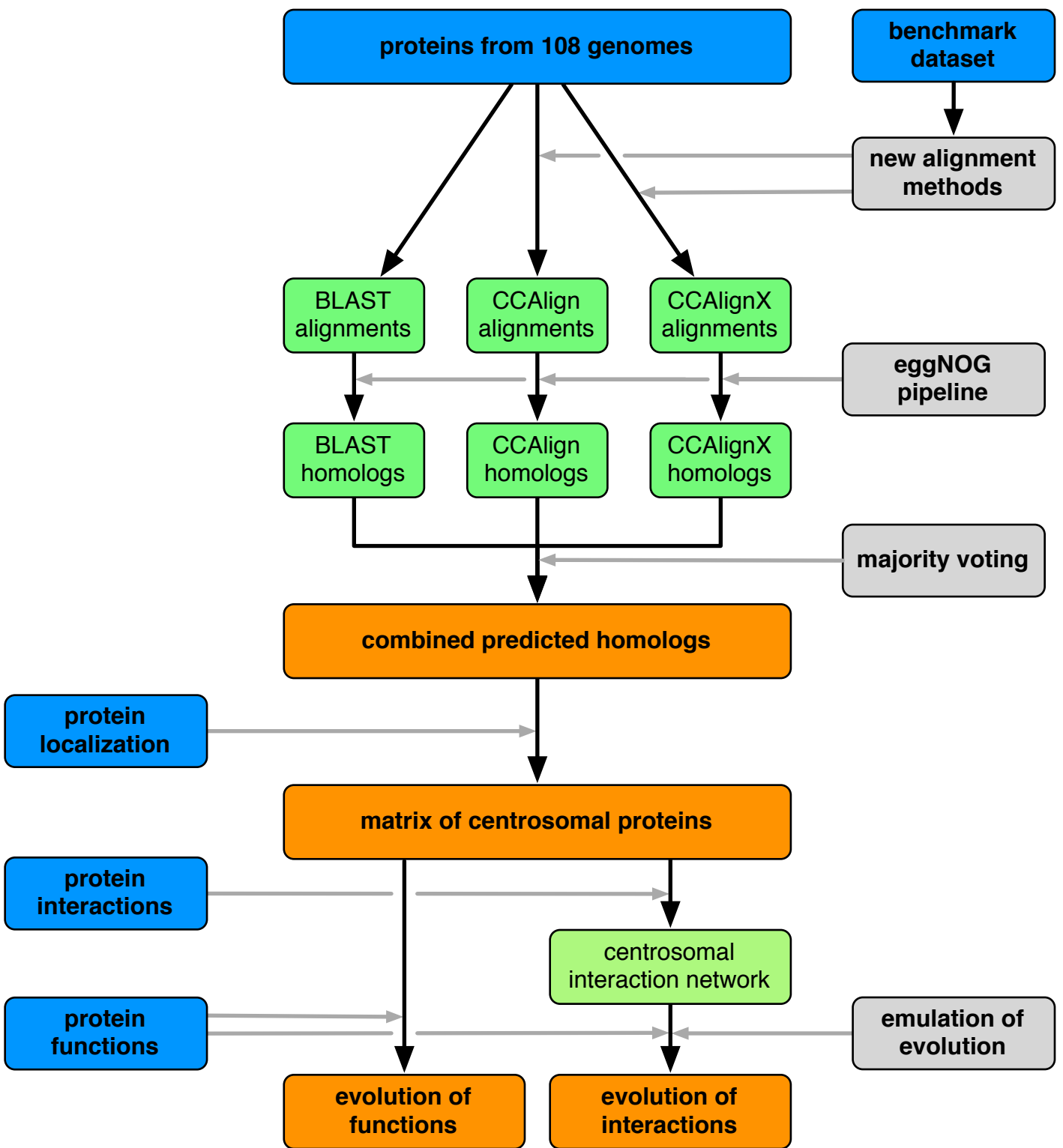

Supplement: Figure S1 — Overview of the pipeline. (PDF) [file pcbi.1003657.s001.pdf]

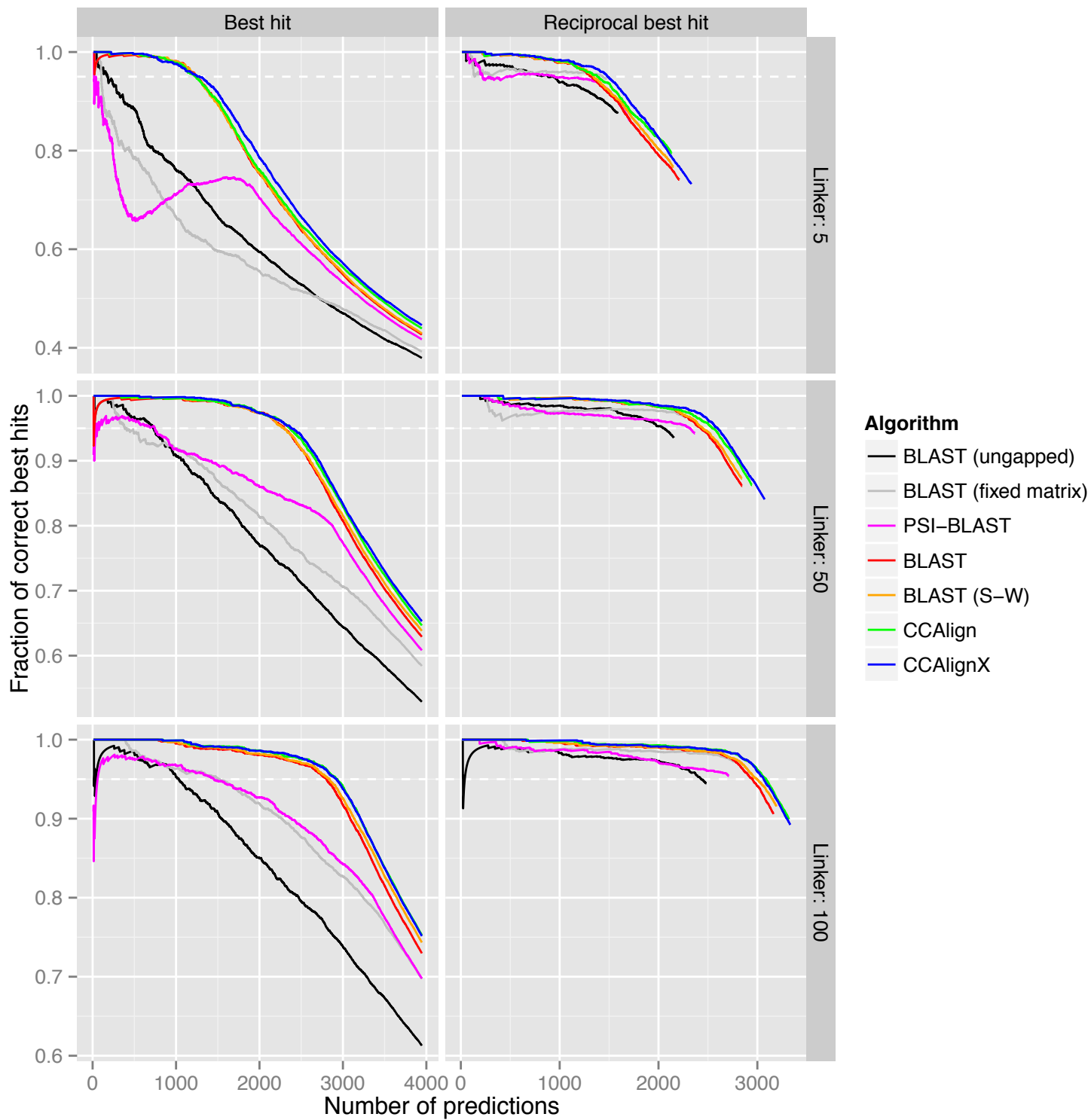

Supplement: Figure S3 — Benchmarking of algorithms, based on the KOG database. See Fig. 2 for full caption. (PDF) [file pcbi.1003657.s003.pdf]

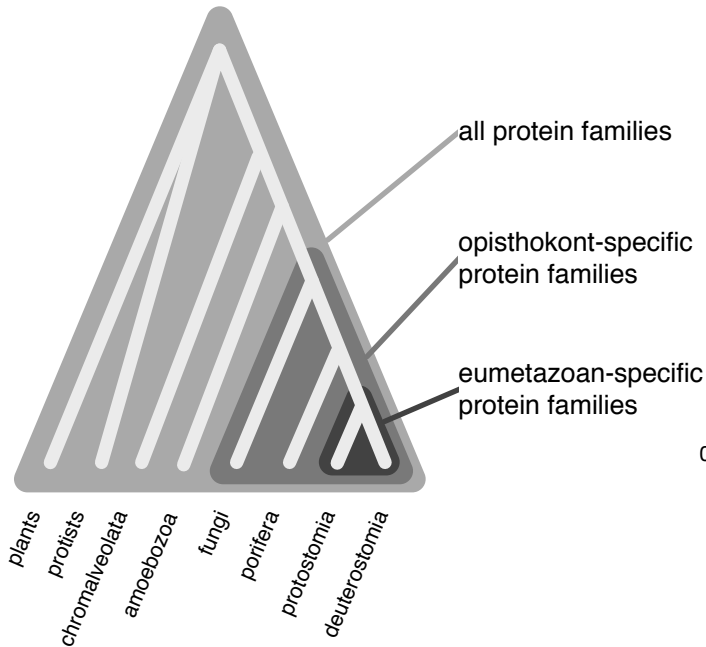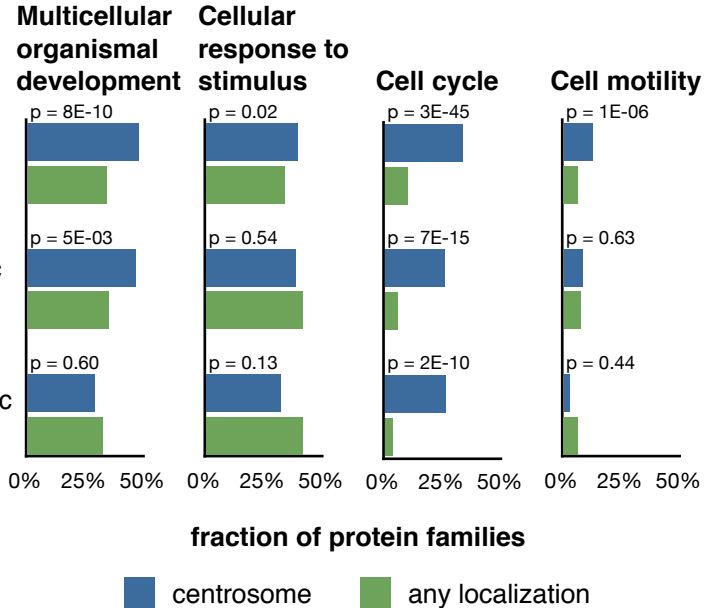

Supplement: Figure S4 — Functions of centrosomal proteins. The fraction of human protein families annotated for various processes is shown for centrosome specific proteins versus proteins of any localization. We investigated the role of centrosomes in four functions important for the animal organism: multicellular organismal development, cellular response to stimulus, cell cycle and cell motility. The centrosome is very important for these functions: compared to proteins of any localization, a significantly larger fraction of centrosomal proteins is involved with these functions. Pre-metazoan protein families are more important for multicellular organismal development than metazoan protein families. The same is true for cellular response to stimulus and cell motility. (PDF) [file pcbi.1003657.s004.pdf]

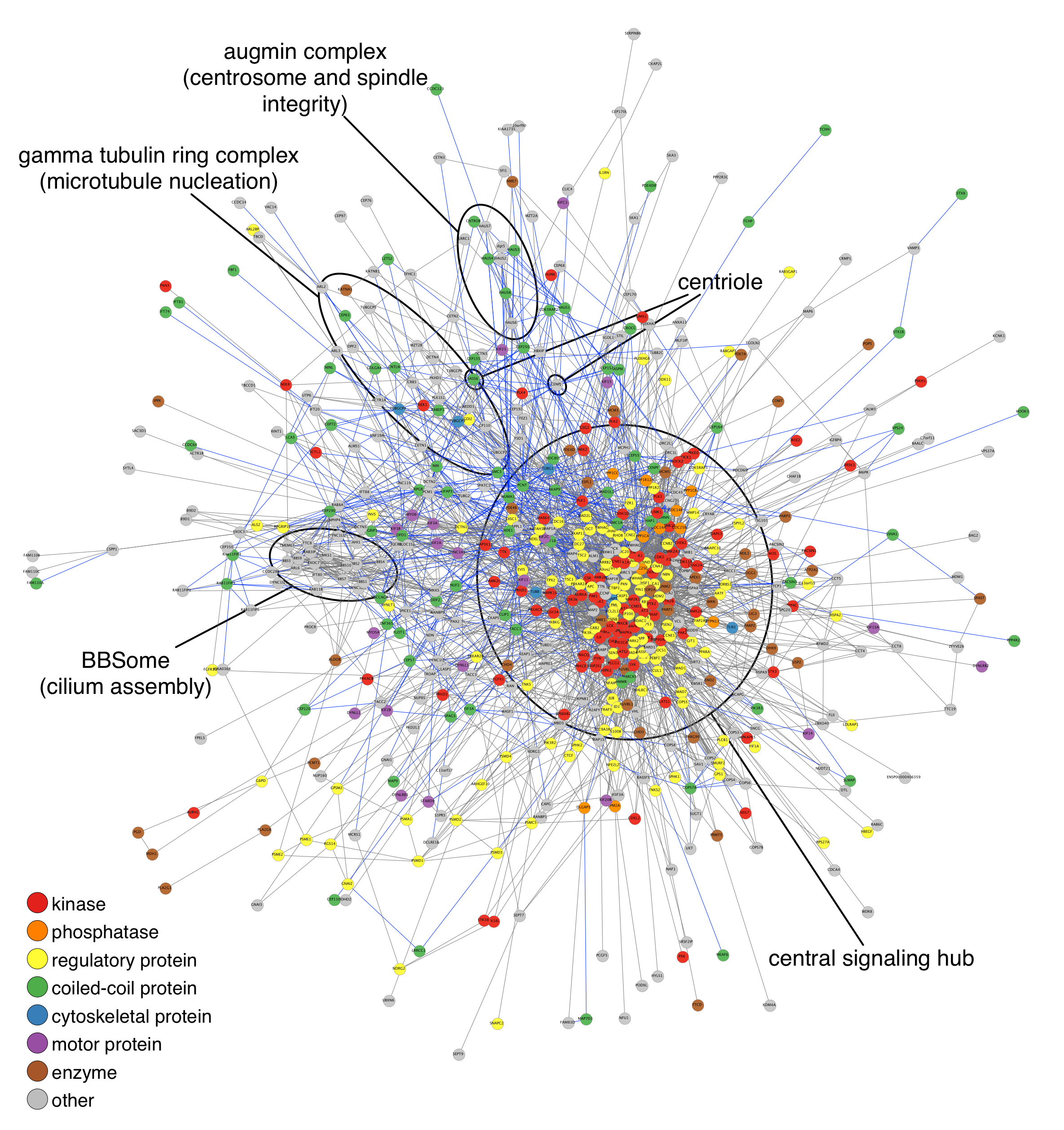

Supplement: Figure S5 — The protein interaction network of the centrosome. Protein-protein interactions were extracted from the STRING 9 database (see Methods). (TIFF) [file pcbi.1003657.s005.tif]

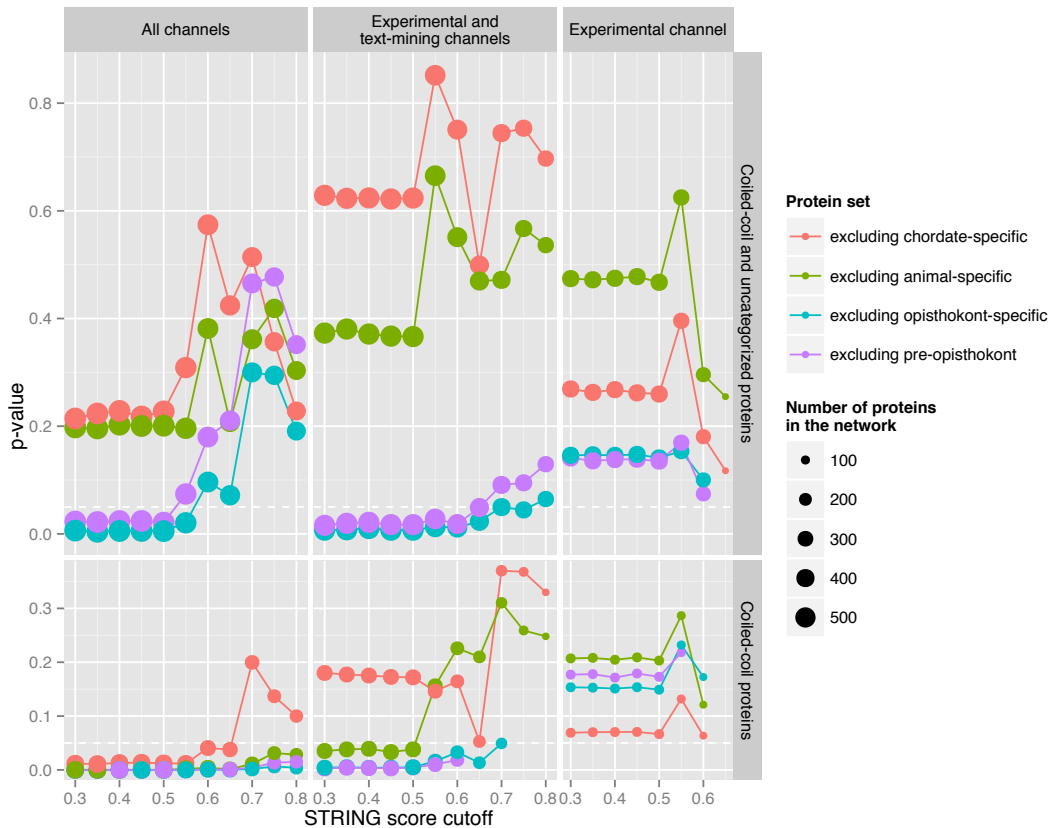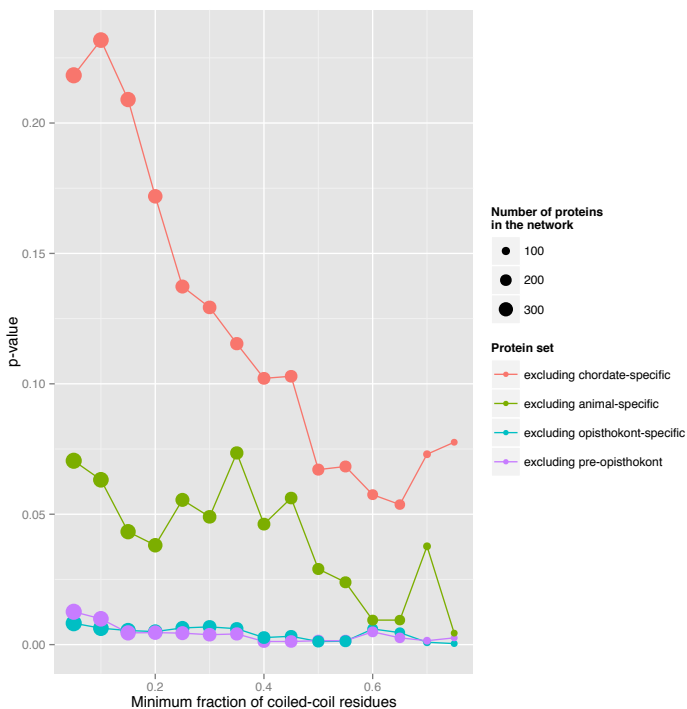

Supplement: Figure S7 — Exploration of different protein interaction networks. P-values for the effect of removing proteins are shown for different STRING networks, score cutoffs and coiled-coil thresholds. When all channels from STRING are used, higher score cutoffs lead to a network dominated by database evidence, which tends to group the centrosome in one large complex. Using the combined experimental and text-mining channels, the p-value for removing opisthokont-specific scaffold and uncategorized proteins is below 0.05 in all but two cases. The experimental-only network is sparser and does not show significant effects. Changing the minimum fraction of coiled-coil residues when designating proteins as scaffolds does not impact the findings. (PDF) [file pcbi.1003657.s007.pdf]

**(a)**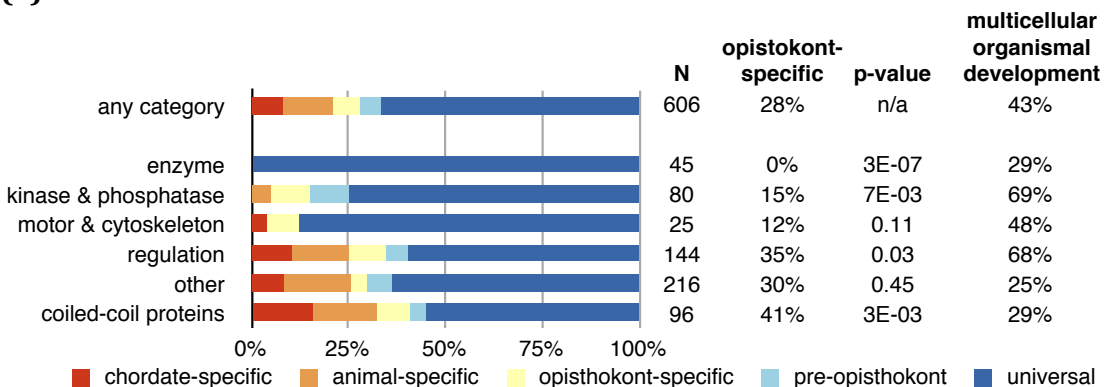**(b)**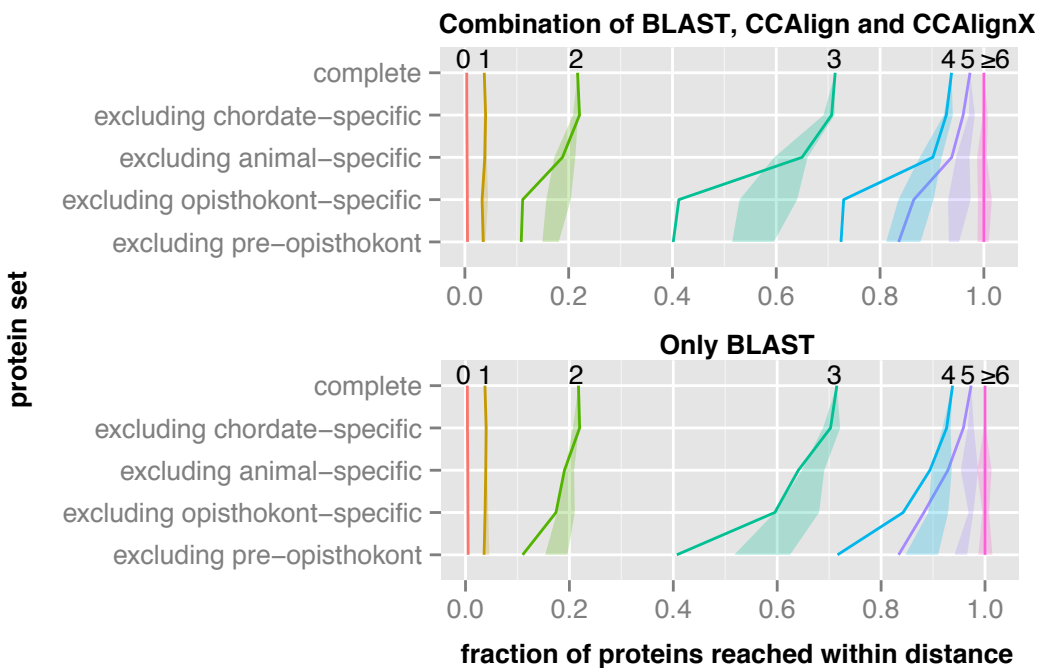

Supplement: Figure S8 — Using only BLAST as alignment method. (a) Using only BLAST to estimate the age of protein families makes coiled-coil proteins appear to be older (41% opisthokont-specific for BLAST vs. 44% for the combination of all three alignment methods). (b) As a consequence, only the removal of proteins that evolved after the last eukaryote ancestor leads to a significant change (at path length 3), although the trends are similar (see also Suppl. Table S5). (PDF) [file pcbi.1003657.s008.pdf]

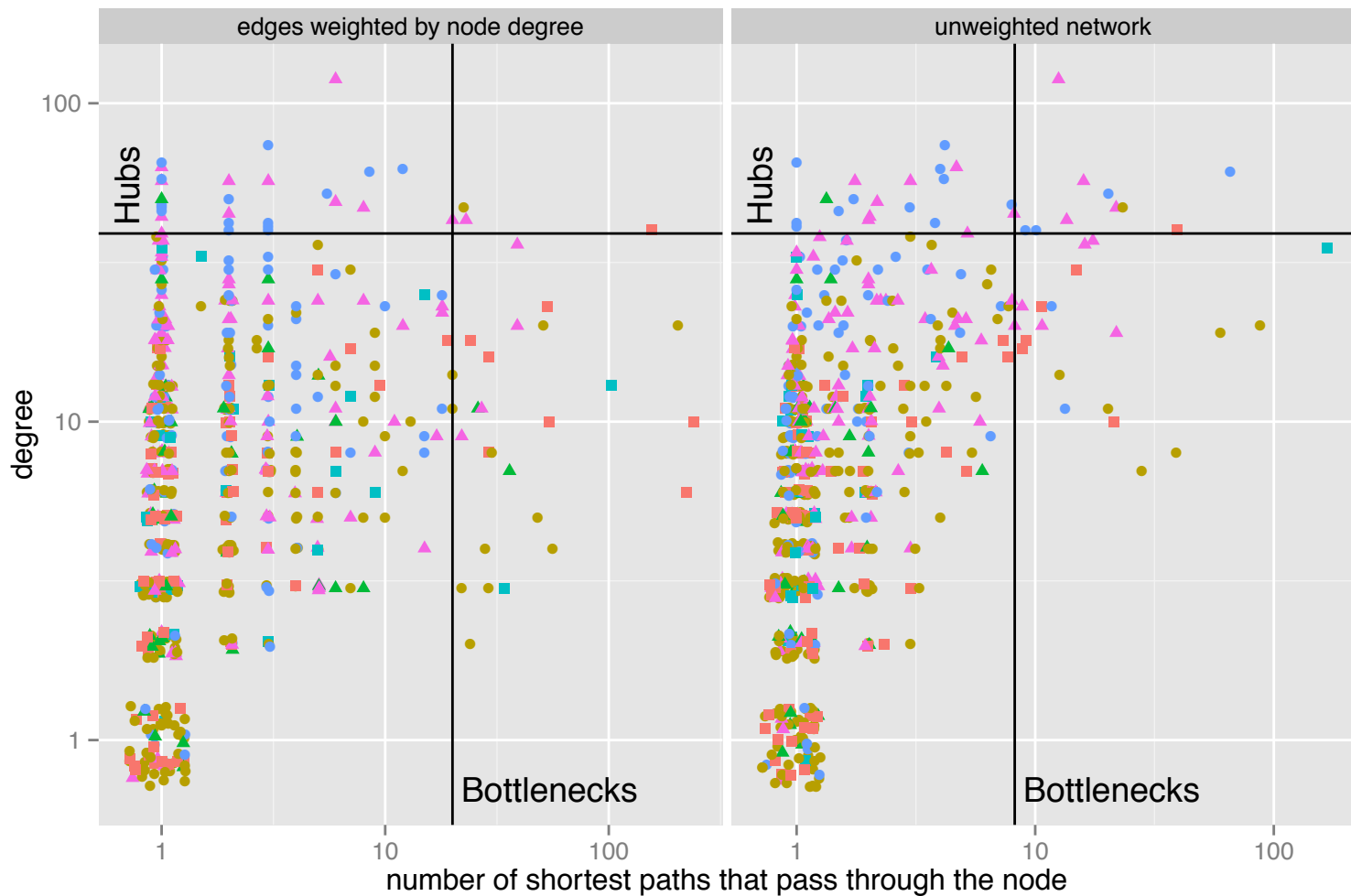

**kind** ■ coiled coil ● other ▲ enzyme ■ motor & cytoskeleton ● kinase & phosphatase ▲ regulation

Supplement: Figure S9 — Analysis of shortest paths. For the complete network, the number of shortest paths that pass through a node is plotted against the degree (number of connections) of the node. The top 5% nodes by degree are hubs, the top 5% by number of shortest paths are bottlenecks. When multiple nodes have the same values, a small random offset is added to reduce over-plotting. (PDF) [file pcbi.1003657.s009.pdf]

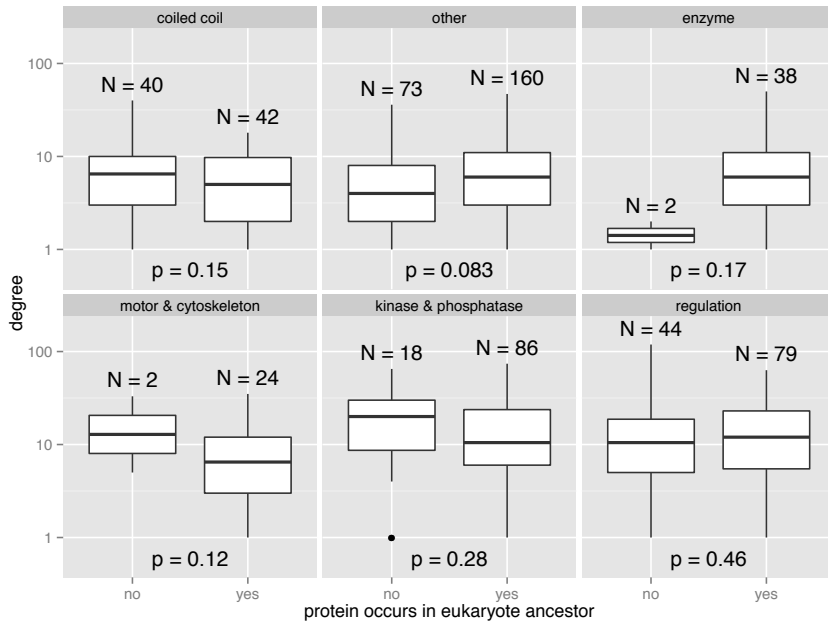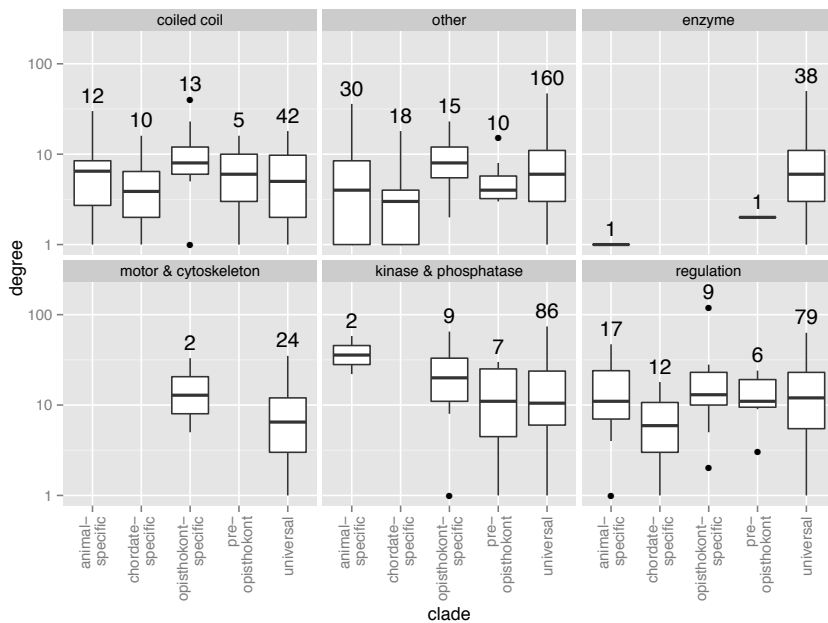

Supplement: Figure S10 — Number of interactions per protein. The degree of the proteins in the human centrosome protein interaction network is shown as a function of evolutionary age. Top: Proteins are divided into those that have been present in the eukaryote ancestor and those that evolved later. P-values have been computed with a permutation test (R package “exactRankTests”). Bottom: All considered clades are shown, along with the number of proteins that first appeared in this clade. (PDF) [file pcbi.1003657.s010.pdf]

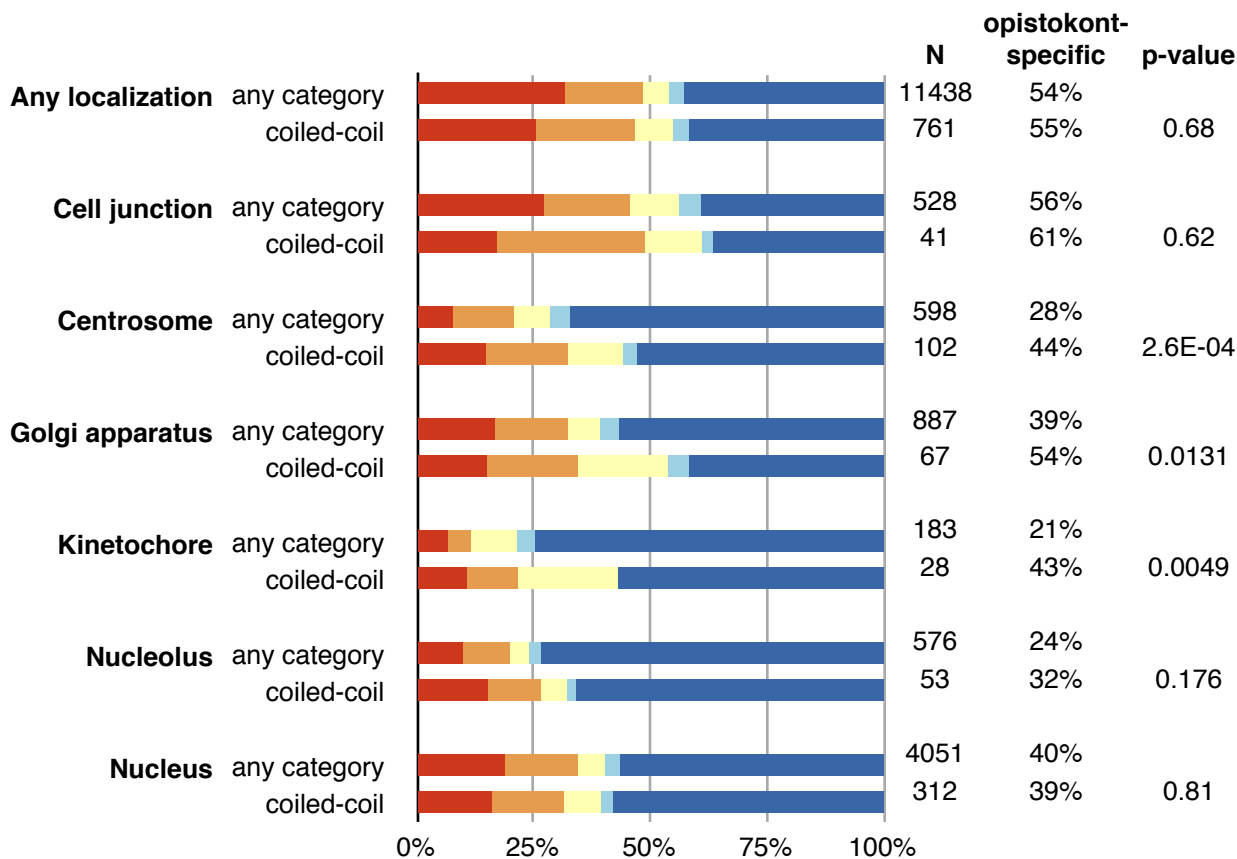

■ chordate-specific 
 ■ animal-specific 
 ■ opisthokont-specific 
 ■ pre-opisthokont 
 ■ universal

Supplement: Figure S11 — Evolutionary age of coiled-coil proteins in different organelles. For organelles as annotated in the Gene Ontology, the age distribution is shown for all proteins and for scaffold proteins. (PDF) [file pcbi.1003657.s011.pdf]

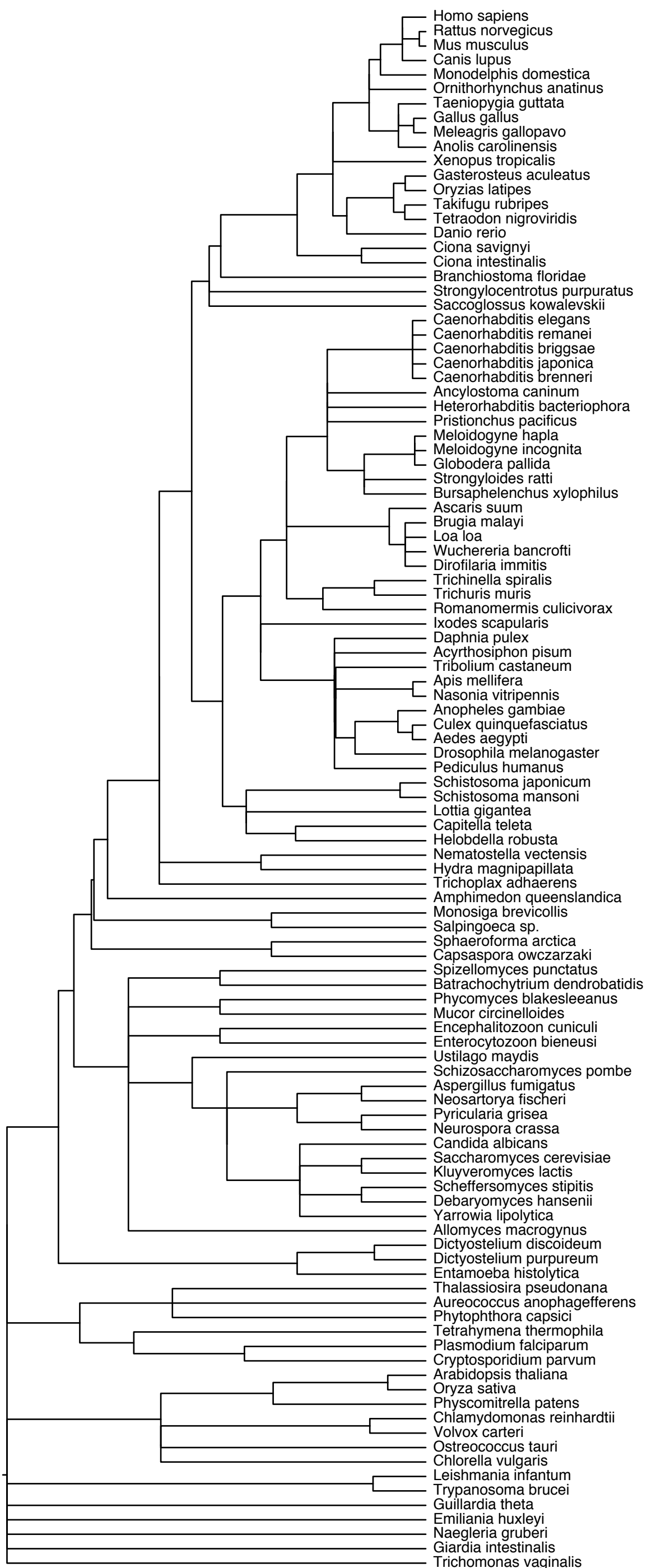

Supplement: Figure S12 — Phylogenetic tree of the 108 species whose genomes have been analyzed. (PDF) [file pcbi.1003657.s012.pdf]
