# Supplementary material for: Coiled-Coil Proteins Facilitated the Functional Expansion of the Centrosome
Source: PLoS Comput Biol. 2014 Jun 5;10(6):e1003657. doi: 10.1371/journal.pcbi.1003657 (PMC4046923; doi:10.1371/journal.pcbi.1003657)
Supplement: Figure S2 — Results of benchmarking. For multiple parameter combinations, the fraction of correctly predicted homologous proteins is calculated. This fraction is compared to the reference fraction using only the BLOSUM62 matrix using the binomial test. Circled: actual parameter combinations used (left: CCAlignX, right: CCAlign). SW-BLAST: BLAST using the Smith-Waterman algorithm. (PDF) [file pcbi.1003657.s002.pdf]

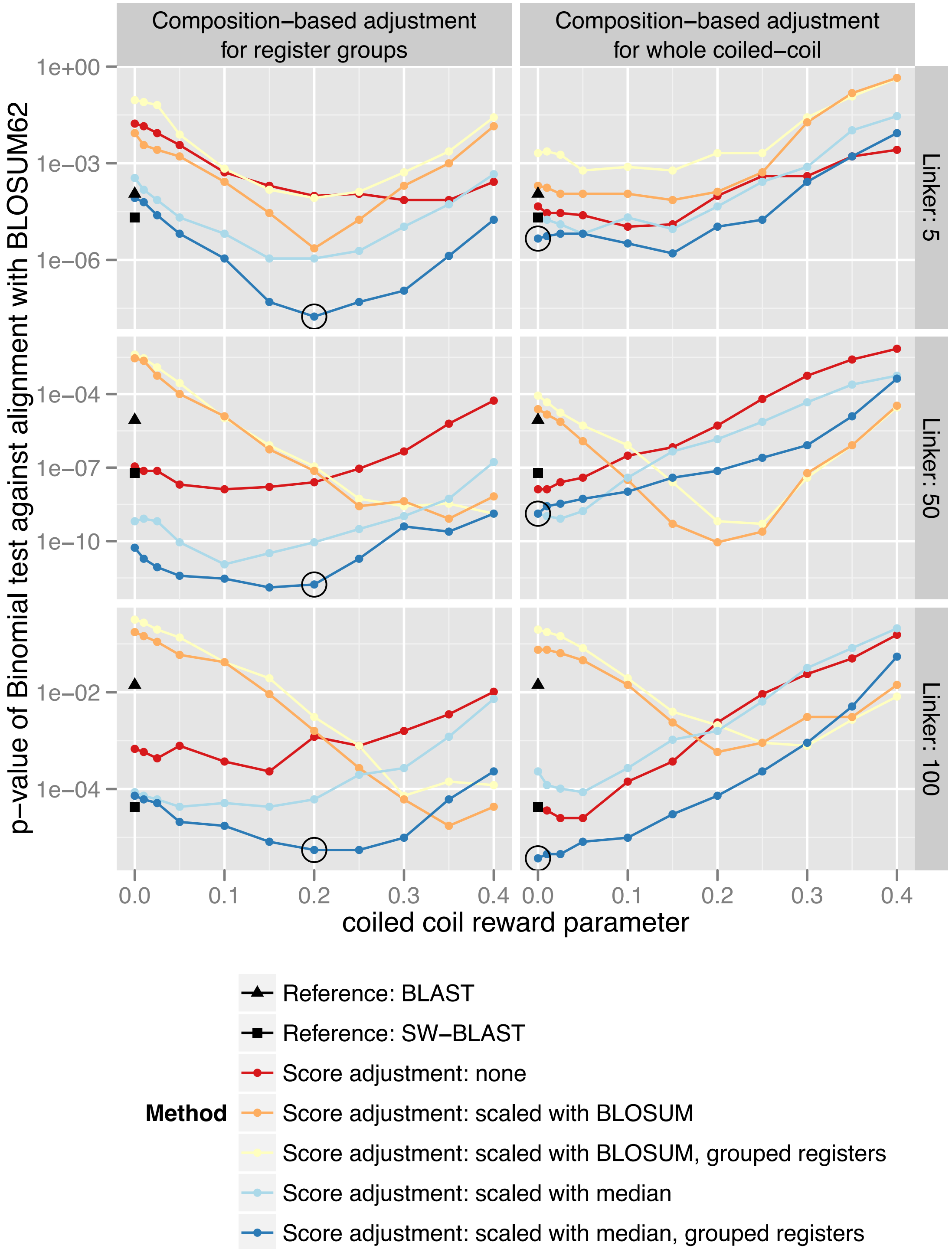
Composition−based adjustment for register groups
Composition−based adjustment for whole coiled−coil
p−value of Binomial test against alignment with BLOSUM62
1e+00
1e−03
1e−06
1e−04
1e−07
1e−10
1e−02
1e−04
Linker: 5
Linker: 50
Linker: 100
0.0
0.1
0.2
0.3
0.4
coiled coil reward parameter
Method
Reference: BLAST
Reference: SW−BLAST
Score adjustment: none
Score adjustment: scaled with BLOSUM
Score adjustment: scaled with BLOSUM, grouped registers
Score adjustment: scaled with median
Score adjustment: scaled with median, grouped registers
