# Supplementary material for: Coiled-Coil Proteins Facilitated the Functional Expansion of the Centrosome
Source: PLoS Comput Biol. 2014 Jun 5;10(6):e1003657. doi: 10.1371/journal.pcbi.1003657 (PMC4046923; doi:10.1371/journal.pcbi.1003657)
Supplement: Figure S6 — The centrosome's evolution compared to the basal body and PCM evolution. For the basal body network, we combined proteins from the centriole, cilium and basal body. To study the evolution of the PCM, we ran the emulation procedure for the whole centrosome, but only consider shortest paths of proteins that are not part of the basal body network. For each shortest path length, we show the fraction of proteins that can be reached within that distance when iteratively removing the most recently evolved proteins. The shaded area corresponds to the second and third quartile of 10,000 randomizations, with p-values for a path length of three steps shown on the right. (PDF) [file pcbi.1003657.s006.pdf]

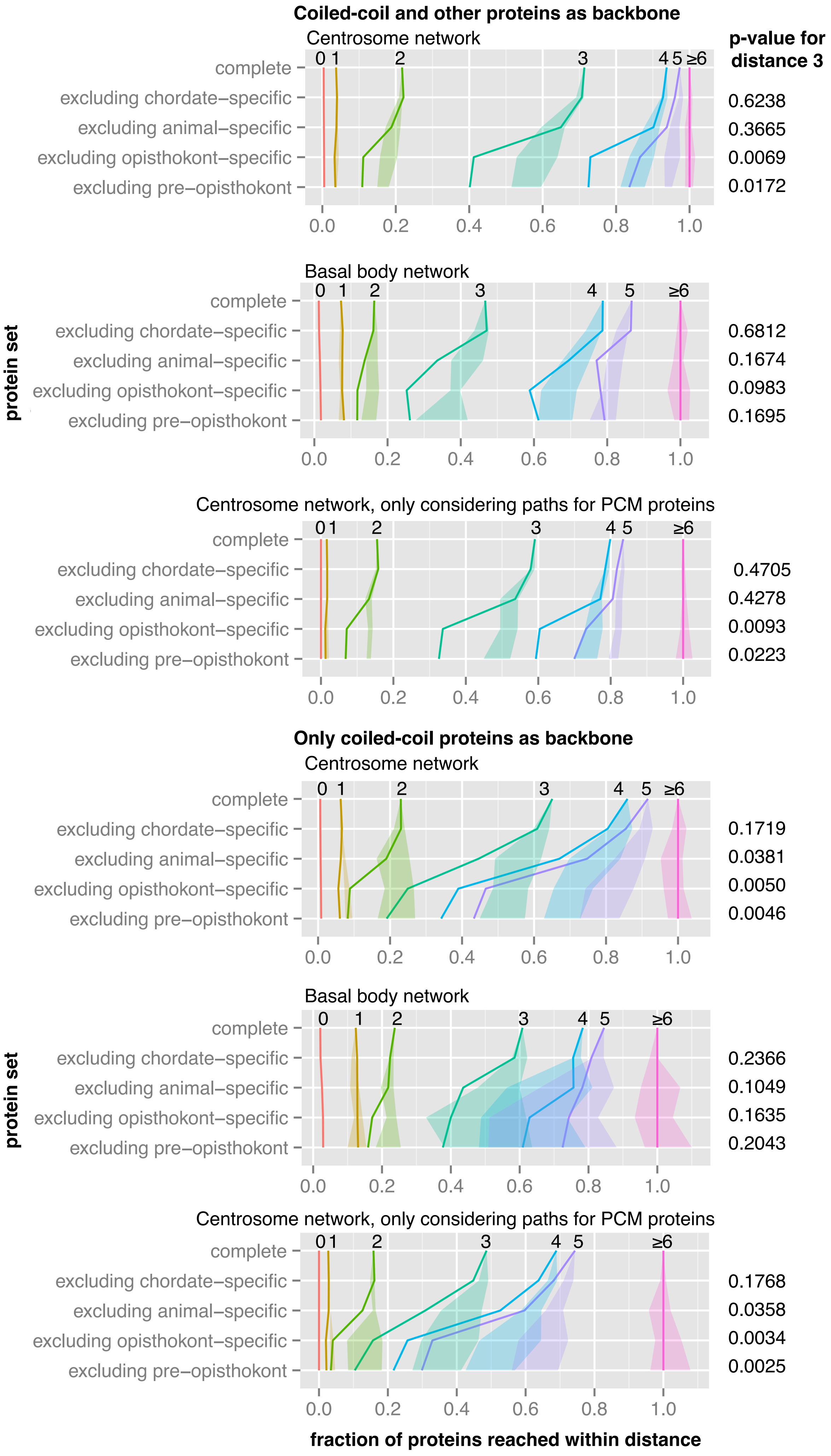

Coiled-coil and other proteins as backbone
Centrosome network
p-value for distance 3
complete
excluding chordate–specific
excluding animal–specific
excluding opisthokont–specific
excluding pre–opisthokont
0 1 2 3 4 5 ≥6
0.6238
0.3665
0.0069
0.0172
Basal body network
0.6812
0.1674
0.0983
0.1695
Centrosome network, only considering paths for PCM proteins
0.4705
0.4278
0.0093
0.0223
Only coiled-coil proteins as backbone
Centrosome network
0.1719
0.0381
0.0050
0.0046
Basal body network
0.2366
0.1049
0.1635
0.2043
Centrosome network, only considering paths for PCM proteins
0.1768
0.0358
0.0034
0.0025
0.0 0.2 0.4 0.6 0.8 1.0
protein set
fraction of proteins reached within distance
