# Supplementary material for: Coiled-Coil Proteins Facilitated the Functional Expansion of the Centrosome
Source: PLoS Comput Biol. 2014 Jun 5;10(6):e1003657. doi: 10.1371/journal.pcbi.1003657 (PMC4046923; doi:10.1371/journal.pcbi.1003657)
Supplement: Figure S13 — Illustration of the procedure to safeguard against spurious links between OGs. (PDF) [file pcbi.1003657.s013.pdf]

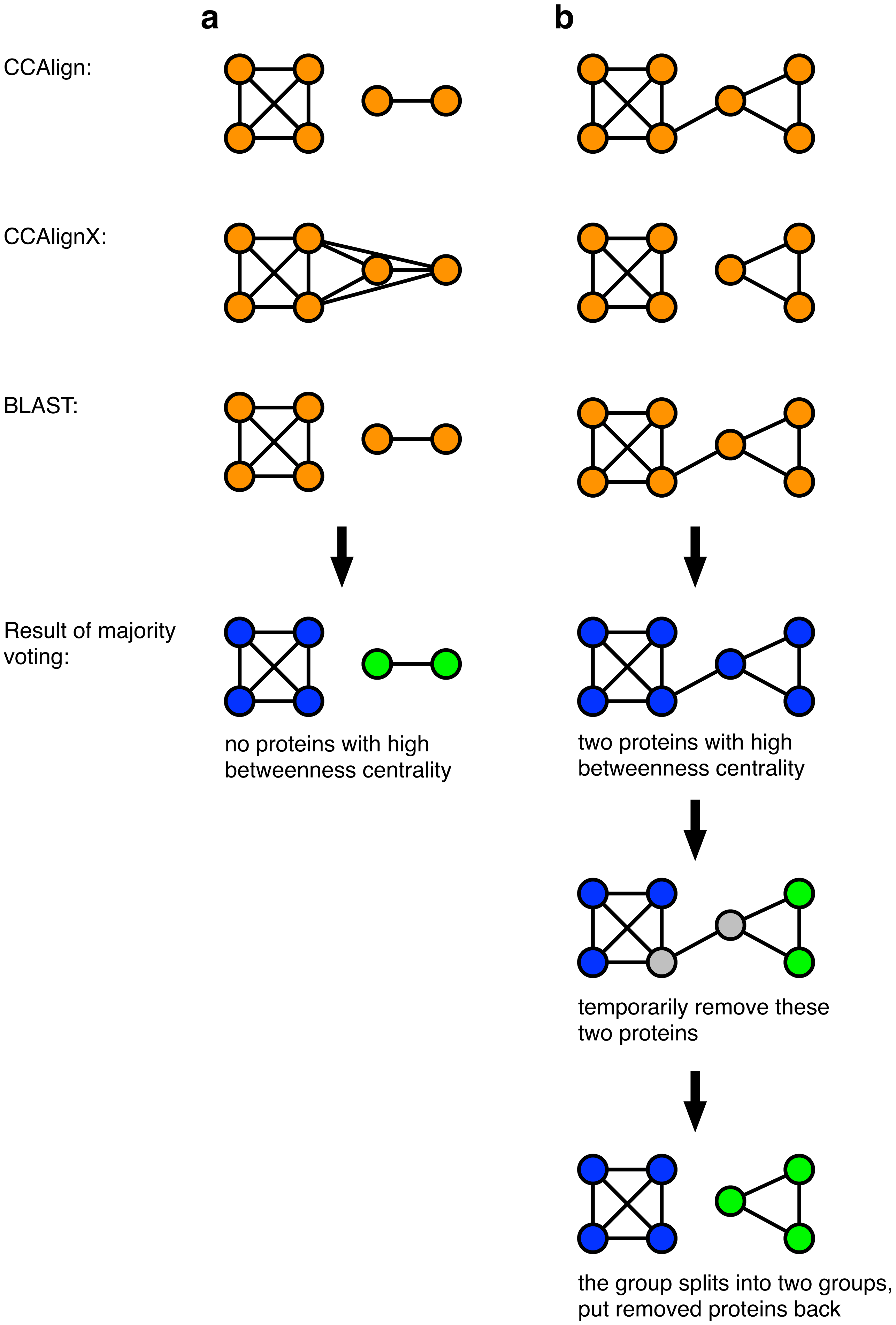
a
b
CCAlign:
CCAlignX:
BLAST:
Result of majority voting:
no proteins with high betweenness centrality
two proteins with high betweenness centrality
temporarily remove these two proteins
the group splits into two groups, put removed proteins back
