# Supplementary material for: Coiled-Coil Proteins Facilitated the Functional Expansion of the Centrosome
Source: PLoS Comput Biol. 2014 Jun 5;10(6):e1003657. doi: 10.1371/journal.pcbi.1003657 (PMC4046923; doi:10.1371/journal.pcbi.1003657)
Supplement: Table S1 — Fraction of coiled-coil residues, species distribution and function of centrosomal coiled-coil proteins. (DOCX) [file pcbi.1003657.s014.docx]

| Human | Fly | *C. elegans* | Species distribution | Previously reported species distribution | Function |
| --- | --- | --- | --- | --- | --- |
| AKAP9 (58%), PCNT (56%) | cp309 (48%) | – | Opisthokonta | none outside metazoa | PCNT is “a multifunctional scaffold for anchoring numerous proteins and protein complexes” [1] |
| CDK5RAP2 (42%) | cnn (49%) | – | Opisthokonta | *S. pombe* (mto1 and pcp1) [2,3] | Targeting and anchoring gamma-tubulin to the centrosome, controlled by aurora kinase A [4] |
| CEP97 (3%) | Cep97 (0%) | R02F11.4 (7%) | LECA | none outside metazoa, not *C. elegans* [5] | Suppression of cilia assembly together with CP110 [6] |
| CEP152 (43%) | asl (80%) | – | Metazoa | Bilateria [5] | Scaffold connecting PLK4 (kinase) and sas-4 (centriolar scaffold) [7] |
| DISC1 (30%) | ­– | – | LECA | plants [8] | Regulation of embryonic and adult neurogenesis. Interacts with many proteins, including PCM1, PCNT (scaffolds), and BBSome proteins (ciliary proteins) |
| KIAA1731 (7%) | ana1 (23%) | – | Metazoa | orthology with Drosophila [9] | Required for centriole duplication in fly [10] |
| NIN (68%), NINL (38%) | Bsg25D (57%) | T04F8.6 (22%) | Opisthokonta and Dictyostelium | Automated annotation in DictyBase, large-scale studies: Metazoa [5] | Anchoring of microtubules to the centriole [11] |
| PCM1 (4%) | CG10732 (21%) | Y56A3A.7 (54%) | Opisthokonta | none outside metazoa | Required for targeting centrin, pericentrin and ninein to the centrosome [12] |
| – | – | spd-5 (52%) | Nematodes | – | Required for centrosome growth, air-1 (kinase), rsa-2 (adaptor for phosphatase) and tac-1 (scaffold) depend on spd-5 for proper localization. [13] |
| – | – | rsa-2 (9%) | Caenorhabditis | – | Mediates interaction between spd-5 (scaffold) and rsa-1 (phosphatase). [13] |

1. Delaval B, Doxsey SJ (2010) Pericentrin in cellular function and disease.

2. Sawin KE, Lourenco PCC, Snaith HA (2004) Microtubule nucleation at non-spindle pole body microtubule-organizing centers requires fission yeast centrosomin-related protein mod20p. Curr Biol 14: 763–775. doi:10.1016/j.cub.2004.03.042.

3. Flory MR, Morphew M, Joseph JD, Means AR, Davis TN (2002) Pcp1p,an Spc110p-related calmodulin target at the centrosome of the fission yeast Schizosaccharomyces pombe. Cell Growth Differ 13: 47–58.

4. Terada Y (2003) Interaction of Aurora-A and centrosomin at the microtubule-nucleating site in Drosophila and mammalian cells. J Cell Biol 162: 757–764. doi:10.1083/jcb.200305048.

5. Hodges ME, Scheumann N, Wickstead B, Langdale JA, Gull K (2010) Reconstructing the evolutionary history of the centriole from protein components. Journal of Cell Science 123: 1407–1413. doi:10.1242/jcs.064873.

6. Bettencourt-Dias M, Carvalho-Santos Z (2008) Double life of centrioles: CP110 in the spotlight. Trends Cell Biol 18: 8–11. doi:10.1016/j.tcb.2007.11.002.

7. Dzhindzhev NS, Yu QD, Weiskopf K, Tzolovsky G, Cunha-Ferreira I, et al. (2010) Asterless is a scaffold for the onset of centriole assembly. Nature 467: 714–718. doi:10.1038/nature09445.

8. Sanchez-Pulido L, Ponting CP (2011) Structure and evolutionary history of DISC1. Hum Mol Genet 20: R175–R181. doi:10.1093/hmg/ddr374.

9. Knorz VJ, Spalluto C, Lessard M, Purvis TL, Adigun FF, et al. (2010) Centriolar association of ALMS1 and likely centrosomal functions of the ALMS motif-containing proteins C10orf90 and KIAA1731. Molecular Biology of the Cell 21: 3617–3629. doi:10.1091/mbc.E10-03-0246.

10. Dobbelaere J, Josué F, Suijkerbuijk S, Baum B, Tapon N, et al. (2008) A genome-wide RNAi screen to dissect centriole duplication and centrosome maturation in Drosophila. PLoS Biol 6: e224. doi:10.1371/journal.pbio.0060224.

11. Delgehyr N, Sillibourne J, Bornens M (2005) Microtubule nucleation and anchoring at the centrosome are independent processes linked by ninein function. Journal of Cell Science 118: 1565–1575. doi:10.1242/jcs.02302.

12. Dammermann A, Merdes A (2002) Assembly of centrosomal proteins and microtubule organization depends on PCM-1. J Cell Biol 159: 255–266. doi:10.1083/jcb.200204023.

13. Boxem M, Maliga Z, Klitgord N, Li N, Lemmens I, et al. (2008) A protein domain-based interactome network for C. elegans early embryogenesis. Cell 134: 534–545. doi:10.1016/j.cell.2008.07.009.
