# Supplementary material for: Coiled-Coil Proteins Facilitated the Functional Expansion of the Centrosome
Source: PLoS Comput Biol. 2014 Jun 5;10(6):e1003657. doi: 10.1371/journal.pcbi.1003657 (PMC4046923; doi:10.1371/journal.pcbi.1003657)
Supplement: Table S4 — Size of MTOC for different species. PCM volume is computed by assuming a spherical centrosome with two cylindrical centrioles of length 0.4 µm and diameter 0.2 µm. (DOCX) [file pcbi.1003657.s017.docx]

| Species | Diameter of MTOC | Volume of PCM |
| --- | --- | --- |
| **Excavates** |  |  |
| *Giardia intestinalis* [1] | 0.5 µm | 0.04 µm^3^ |
| *Naegleria gruberi* [2] | acentriolar mitotic MTOC inside nucleus, either with polar cap (0.7 µm) or MTs end at nuclear membrane | |
| **Plants** |  |  |
| *Chlamydomonas reinhardtii* [3-5] | acentriolar mitotic MTOC: 0.5 µm reported by Johnson, however later studies mention basal bodies at the spindle poles | |
| *Hydrodictyon reticulatum* [6] | 0.6 µm | 0.09 µm^3^ |
| **Chromalveolata** |  |  |
| *Surirella ovalis* [7] | acentriolar mitotic MTOC: 0.5 µm | |
| **Fungi** |  |  |
| Chytrids |  |  |
| *Monoblepharella* [8] | 0.5 µm | 0.04 µm^3^ |
| *Rhizophydium spherotheca* [9] | 0.25 µm | (almost none) |
| **Animals** |  |  |
| Invertebrates |  |  |
| *Caenorhabditis elegans* [10] | 4 µm (oocyte) | 33 µm^3^ |
| *Drosophila melanogaster* [11] | 0.8 µm (embryo) | 0.2 µm^3^ |
| *Spisula* [12] | 1 µm (oocyte) | 0.5 µm^3^ |
| Vertebrates |  |  |
| *Homo sapiens* [13] | 0.9 µm (prostate cells) | 0.4 µm^3^ |
| *Potorous tridactylus* [14] | 1 µm (PtK_1_ cells) | 0.5 µm^3^ |
| *Xenopus laevis* [15] | 0.7 µm (A6 cells, interphase)  1 µm (A6 cells, metaphase) | 0.2 µm^3^  0.5 µm^3^ |

**References**

1. Sagolla MS, Dawson SC, Mancuso JJ, Cande WZ (2006) Three-dimensional analysis of mitosis and cytokinesis in the binucleate parasite Giardia intestinalis. Journal of Cell Science 119: 4889–4900. doi:10.1242/jcs.03276.

2. Schuster FL (1975) Ultrastructure of mitosis in the amoeboflagellate Naegleria gruberi. Tissue Cell 7: 1–11.

3. Johnson UG, Porter KR (1968) Fine structure of cell division in Chlamydomonas reinhardi. Basal bodies and microtubules. J Cell Biol 38: 403–425.

4. Gaffal KP (1988) The Basal Body-Root Complex of Chlamydomonas-Reinhardtii During Mitosis. Protoplasma 143: 118–129.

5. Dutcher SK (2003) Elucidation of basal body and centriole functions in Chlamydomonas reinhardtii. Traffic 4: 443–451.

6. Marchant HJ, Pickett-Heaps JD (1970) Ultrastructure and differentiation of Hydrodictyon reticulatum. I. Mitosis in the coenobium. Aust J Biol Sci 23: 1173–1186.

7. Drum RW, Pankratz HS (1963) Fine Structure of a Diatom Centrosome. Science 142: 61–63.

8. Dolan T, Fuller MS (1985) The Ultrastructure of Nuclear Division in Monoblepharella Sp. Mycologia 77: 791–809.

9. Powell MJ (1980) Mitosis in the Aquatic Fungus Rhizophydium-Spherotheca (Chytridiales). Am J Bot 67: 839–853.

10. Decker M, Jaensch S, Pozniakovsky A, Zinke A, O'Connell KF, et al. (2011) Limiting amounts of centrosome material set centrosome size in C. elegans embryos. Curr Biol 21: 1259–1267. doi:10.1016/j.cub.2011.06.002.

11. Moritz M, Braunfeld MB, Fung JC, Sedat JW, Alberts BM, et al. (1995) Three-dimensional structural characterization of centrosomes from early Drosophila embryos. J Cell Biol 130: 1149–1159.

12. Schnackenberg BJ, Khodjakov A, Rieder CL, Palazzo RE (1998) The disassembly and reassembly of functional centrosomes in vitro. Proc Natl Acad Sci USA 95: 9295–9300.

13. Pihan GA, Purohit A, Wallace J, Malhotra R, Liotta L, et al. (2001) Centrosome defects can account for cellular and genetic changes that characterize prostate cancer progression. Cancer Res 61: 2212–2219.

14. Rieder CL (1990) Formation of the astral mitotic spindle: ultrastructural basis for the centrosome-kinetochore interaction. Electron Microsc Rev 3: 269–300.

15. McNally FJ, Thomas S (1998) Katanin is responsible for the M-phase microtubule-severing activity in Xenopus eggs. Molecular Biology of the Cell 9: 1847–1861.
