# Supplementary material for: Coiled-Coil Proteins Facilitated the Functional Expansion of the Centrosome
Source: PLoS Comput Biol. 2014 Jun 5;10(6):e1003657. doi: 10.1371/journal.pcbi.1003657 (PMC4046923; doi:10.1371/journal.pcbi.1003657)
Supplement: Table S5 — Simulating centrosome evolution with different backbones. (DOCX) [file pcbi.1003657.s018.docx]

|  |  | p-value* | | | | | |
| --- | --- | --- | --- | --- | --- | --- | --- |
| **Protein classes**  **in backbone** | Fraction of nodes reached | Excluding  chordate-  specific  proteins | Excluding  animal-  specific  proteins | Excluding  opisthokont  specific  proteins | | Excluding  pre-opisthokont  proteins |  |
| **Orthologs generated using CCAlign, CCAlignX and BLAST** | | | | | | |  |
| all proteins | 100% | 0.931 | 0.597 | **0.030** | | 0.060 |  |
|  |  |  |  |  | |  |  |
| enzyme | 35% | 0.466 | 0.575 | 0.355 | | 0.471 |  |
| kinase & phosphatase | 66% | 0.668 | 0.330 | 0.387 | | 0.424 |  |
| motor & cytoskeleton | 29% | 0.614 | 0.541 | 0.564 | | 0.556 |  |
| other | 74% | 0.822 | 0.527 | **0.015** | | **0.031** |  |
| regulation & TF | 52% | 0.888 | 0.653 | 0.083 | | 0.140 |  |
| coiled-coil | 35% | 0.172 | **0.038** | **0.005** | | **0.005** |  |
|  |  |  |  |  | |  |  |
| enzyme & other | 90% | 0.894 | 0.642 | **0.020** | | 0.049 |  |
| kinase & phosphatase & other | 95% | 0.829 | 0.604 | 0.097 | | 0.137 |  |
| motor & cytoskeleton & other | 88% | 0.792 | 0.587 | **0.027** | | 0.067 |  |
| regulation & TF & other | 86% | 0.940 | 0.705 | **0.024** | | 0.052 |  |
| coiled-coil & other | 96% | 0.624 | 0.367 | **0.007** | | **0.017** |  |
| **Orthologs generated using only BLAST** | | | | | | |  |
| all proteins | 100% | 0.911 | 0.668 | | 0.469 | 0.184 |  |
|  |  |  |  | |  |  |  |
| enzyme | 35% | 0.476 | 0.578 | | 0.554 | 0.470 |  |
| kinase & phosphatase | 66% | 0.714 | 0.342 | | 0.407 | 0.384 |  |
| motor & cytoskeleton | 29% | 0.529 | 0.568 | | 0.571 | 0.653 |  |
| other | 74% | 0.558 | 0.399 | | 0.257 | 0.061 |  |
| regulation & TF | 52% | 0.827 | 0.760 | | 0.387 | 0.303 |  |
| coiled-coil | 35% | 0.177 | 0.057 | | **0.044** | **0.006** |  |
|  |  |  |  | |  |  |  |
| enzyme & other | 90% | 0.704 | 0.564 | | 0.321 | 0.083 |  |
| kinase & phosphatase & other | 95% | 0.821 | 0.530 | | 0.372 | 0.208 |  |
| motor & cytoskeleton & other | 88% | 0.548 | 0.456 | | 0.370 | 0.130 |  |
| regulation & TF & other | 86% | 0.948 | 0.859 | | 0.373 | 0.158 |  |
| coiled-coil & other | 96% | 0.492 | 0.316 | | 0.281 | **0.029** |  |

* P-values are calculating by shuffling the evolutionary age 10,000 times.
