# Supplementary material for: Coiled-Coil Proteins Facilitated the Functional Expansion of the Centrosome
Source: PLoS Comput Biol. 2014 Jun 5;10(6):e1003657. doi: 10.1371/journal.pcbi.1003657 (PMC4046923; doi:10.1371/journal.pcbi.1003657)
Supplement: Table S6 — Enrichment of bottlenecks in the centrosome interaction network. (DOCX) [file pcbi.1003657.s019.docx]

|  | Fraction among all nodes | **unweighted network** | | | | **weighted network** | |
| --- | --- | --- | --- | --- | --- | --- | --- |
|  |  | Bottlenecks | | Nonhub-bottlenecks | | Bottlenecks | |
| Functional class |  | Fraction | p-value* | Fraction | p-value | Fraction | p-value |
| enzyme | 7% | 0% | 1.00 | 0% | 1.00 | 7% | 0.61 |
| kinase & phosphatase | 17% | 20% | 0.41 | 10% | 0.88 | 0% | 1.00 |
| motor & cytoskeleton | 4% | 3% | 0.75 | 5% | 0.59 | 7% | 0.38 |
| other | 38% | 23% | 0.98 | 30% | 0.84 | 40% | 0.49 |
| regulation | 21% | 33% | *0.07* | 30% | 0.21 | 20% | 0.61 |
| coiled-coil | 13% | 20% | 0.18 | 25% | *0.11* | 27% | **0.03** |

* P-values are calculated with a one-sided Fisher’s exact test.
