# Supplementary material for: Coiled-Coil Proteins Facilitated the Functional Expansion of the Centrosome
Source: PLoS Comput Biol. 2014 Jun 5;10(6):e1003657. doi: 10.1371/journal.pcbi.1003657 (PMC4046923; doi:10.1371/journal.pcbi.1003657)
Supplement: Text S1 — Supplementary Information. Includes the sections “Robustness of the model” and “Verification of the model.” (DOCX) [file pcbi.1003657.s020.docx]

# Robustness of the model

In the model for emulating the history of the centrosome, there are a number of choices: Which protein classes make up the structural backbone? Is it necessary to assume a structural backbone? In this section, we show that coiled-coil proteins are unique among the protein classes regarding their ability to recruit other proteins to the centrosome. Furthermore, we use a different approach that has less statistical power, but also makes fewer assumptions on the behavior of proteins. First, we repeated the above analysis, but replaced coiled-coil proteins with other functional classes in the structural backbone. We tested the individual classes, and the combination of the individual classes with the uncharacterized proteins. Using a p-value cut-off of 0.05, only coiled-coil proteins (p-value 0.005) and uncharacterized proteins (p-value 0.015) significantly changed the network when opisthokont-specific proteins were removed (Table S5). Due to the strong individual signal of uncharacterized proteins, all but one class changed the network significantly when combined with uncharacterized proteins in the structural backbone. However, the combination of coiled-coil and uncharacterized proteins has the most significant p-value.

Second, we propose an additional test for the importance of coiled-coil proteins to the cohesion of the centrosome. In this model, we used all protein–protein interactions. We computed how many shortest paths pass through each protein, originating from the centriole (i.e. from CENPJ or SASS6). This is equivalent to calculating the betweenness of the proteins relative to the centriole. In this additional test, the evolutionary age of the protein is not relevant. Thus, even if all coiled-coil proteins were more ancient than currently known, the findings from this test would still hold. In this analysis we distinguished bottlenecks and hubs [1]: bottlenecks are proteins with a high betweenness, and hubs are proteins with a high degree (Fig. S5). Using the top 5% as a cut-off value in each of the classes, we found that regulatory proteins were most over-represented among bottlenecks (33% vs. 21% among all nodes, p-value of one-sided Fisher’s exact test: 0.07, see Table S6). However, when we considered bottlenecks that are not hubs, coiled-coil proteins were most over-represented (25% vs. 13%, p-value 0.11). To control for hubs regardless of their functional class, it is also possible the assign weights to the edges based on the normalized degree of the connected nodes. For each protein, its degree (i.e. the number of interaction partners) is determined, and normalized by the number of residues. This way, relatively short proteins with many connections, like kinases, receive a high weight. The shortest path calculation will then avoid these proteins. In this weighted network, only coiled-coil proteins are over-represented among the hubs (27% hubs vs. 13% among all nodes, p-value 0.03).

# Verification of the model

To our knowledge, this is the first study to trace the evolutionary trajectory of a whole organelle. Thus, there are no accepted concepts for the verification of the presented model. Michael Scriven proposed criteria to verify a model of evolution: “We have to show […] that (i) this cause was in fact present, (ii) *independent* evidence supports the claim that it can produce this effect, and (iii) no other such causes were present.” [2] In our model, the cause for the complex metazoan centrosome is the recruitment of pre-existing proteins to the newly forming organelle by coiled-coil scaffolds. Through our refined sequence alignment, we have shown that the present-day coiled-coil scaffold proteins were already present in the opisthokont ancestor. Independent evidence for the capabilities of coiled-coil proteins comes from synthetic biology. Coiled-coil proteins have been designed to form three-dimensional hydrogel networks with varying physical properties [3]. Furthermore, it is possible to optimize coiled-coil proteins to achieve specific binding towards specific targets [4,5]. Thus, independent evidence shows that coiled-coil proteins can form three-dimensional structures like the PCM, and can rapidly change their binding specificities. Lastly, is it possible that other causes contributed to the formation of the centrosome? In the above section, we have shown the contribution of novel proteins from other protein classes is less significant than the contribution from coiled-coil proteins. The growth of a protein interaction network through gene duplication and formation of novel links between existing proteins has been independently modeled [6,7], with the prediction that evolutionary ancient proteins should have more connections in the network. This, however, is not true for the centrosome interaction network, which evolved through the innovation of new coiled-coil scaffold proteins: there is no significant difference between proteins that occurred in the eukaryote ancestor and proteins that evolved later (p-value of permutation test: 0.41, see Fig. S10).

# References

1. Yu H, Kim PM, Sprecher E, Trifonov V, Gerstein M (2007) The importance of bottlenecks in protein networks: correlation with gene essentiality and expression dynamics. PLoS Comput Biol 3: e59. doi:10.1371/journal.pcbi.0030059.

2. Scriven M (1959) Explanation and prediction in evolutionary theory. Science 130: 477–482.

3. Robson Marsden H, Kros A (2010) Self-assembly of coiled coils in synthetic biology: inspiration and progress. Angew Chem Int Ed Engl 49: 2988–3005. doi:10.1002/anie.200904943.

4. Mason JM, Schmitz MA, Müller KM, Arndt KM (2006) Semirational design of Jun-Fos coiled coils with increased affinity: Universal implications for leucine zipper prediction and design. Proc Natl Acad Sci USA 103: 8989–8994. doi:10.1073/pnas.0509880103.

5. Mason JM, Müller KM, Arndt KM (2007) Positive aspects of negative design: simultaneous selection of specificity and interaction stability. Biochemistry 46: 4804–4814. doi:10.1021/bi602506p.

6. Barabasi A, Albert R (1999) Emergence of scaling in random networks. Science 286: 509–512.

7. Peterson GJ, Pressé S, Peterson KS, Dill KA (2012) Simulated evolution of protein-protein interaction networks with realistic topology. PLoS ONE 7: e39052. doi:10.1371/journal.pone.0039052.
