# Supplementary material for: Coiled-Coil Proteins Facilitated the Functional Expansion of the Centrosome
Source: PLoS Comput Biol. 2014 Jun 5;10(6):e1003657. doi: 10.1371/journal.pcbi.1003657 (PMC4046923; doi:10.1371/journal.pcbi.1003657)
Supplement: Dataset S1 — Multiple-sequence alignments. This file contains alignments for the protein families spd-5, AKAP9/PCNT, PCM1, HAUS7 and HAUS8 in FASTA format and as HTML pages with highlighted coiled-coil domains. (ZIP) [file pcbi.1003657.s021.zip › alignments/AKAP9_PCNT.html]

Multiple Alignment


1  
|

5  
|

10  
|

15  
|

20  
|

25  
|

30  
|

35  
|

40  
|

45  
|

50  
|

55  
|

60  
|

65  
|

70  
|

75  
|

80  
|

85  
|

90  
|

95  
|

100  
|

105  
|

110  
|

115  
|

120  
|

125  
|

130  
|

135  
|

140  
|

145  
|

150  
|

155  
|

160  
|

165  
|

170  
|

175  
|

180  
|

185  
|

190  
|

195  
|

200  
|

205  
|

210  
|

215  
|

220  
|

225  
|

230  
|

235  
|

240  
|

245  
|

250  
|

255  
|

260  
|

265  
|

270  
|

275  
|

280  
|

285  
|

290  
|

295  
|

300  
|

305  
|

310  
|

315  
|

320  
|

325  
|

330  
|

335  
|

340  
|

345  
|

350  
|

355  
|

360  
|

365  
|

370  
|

375  
|

380  
|

385  
|

390  
|

395  
|

400  
|

405  
|

410  
|

415  
|

420  
|

425  
|

430  
|

435  
|

440  
|

445  
|

450  
|

455  
|

460  
|

465  
|

470  
|

475  
|

480  
|

485  
|

490  
|

495  
|

500  
|

505  
|

510  
|

515  
|

520  
|

525  
|

530  
|

535  
|

540  
|

545  
|

550  
|

555  
|

560  
|

565  
|

570  
|

575  
|

580  
|

585  
|

590  
|

595  
|

600  
|

605  
|

610  
|

615  
|

620  
|

625  
|

630  
|

635  
|

640  
|

645  
|

650  
|

655  
|

660  
|

665  
|

670  
|

675  
|

680  
|

685  
|

690  
|

695  
|

700  
|

705  
|

710  
|

715  
|

720  
|

725  
|

730  
|

735  
|

740  
|

745  
|

750  
|

755  
|

760  
|

765  
|

770  
|

775  
|

780  
|

785  
|

790  
|

795  
|

800  
|

805  
|

810  
|

815  
|

820  
|

825  
|

830  
|

835  
|

840  
|

845  
|

850  
|

855  
|

860  
|

865  
|

870  
|

875  
|

880  
|

885  
|

890  
|

895  
|

900  
|

905  
|

910  
|

915  
|

920  
|

925  
|

930  
|

935  
|

940  
|

945  
|

950  
|

955  
|

960  
|

965  
|

970  
|

975  
|

980  
|

985  
|

990  
|

995  
|

1000  
|

1005  
|

1010  
|

1015  
|

1020  
|

1025  
|

1030  
|

1035  
|

1040  
|

1045  
|

1050  
|

1055  
|

1060  
|

1065  
|

1070  
|

1075  
|

1080  
|

1085  
|

1090  
|

1095  
|

1100  
|

1105  
|

1110  
|

1115  
|

1120  
|

1125  
|

1130  
|

1135  
|

1140  
|

1145  
|

1150  
|

1155  
|

1160  
|

1165  
|

1170  
|

1175  
|

1180  
|

1185  
|

1190  
|

1195  
|

1200  
|

1205  
|

1210  
|

1215  
|

1220  
|

1225  
|

1230  
|

1235  
|

1240  
|

1245  
|

1250  
|

1255  
|

1260  
|

1265  
|

1270  
|

1275  
|

1280  
|

1285  
|

1290  
|

1295  
|

1300  
|

1305  
|

1310  
|

1315  
|

1320  
|

1325  
|

1330  
|

1335  
|

1340  
|

1345  
|

1350  
|

1355  
|

1360  
|

1365  
|

1370  
|

1375  
|

1380  
|

1385  
|

1390  
|

1395  
|

1400  
|

1405  
|

1410  
|

1415  
|

1420  
|

1425  
|

1430  
|

1435  
|

1440  
|

1445  
|

1450  
|

1455  
|

1460  
|

1465  
|

1470  
|

1475  
|

1480  
|

1485  
|

1490  
|

1495  
|

1500  
|

1505  
|

1510  
|

1515  
|

1520  
|

1525  
|

1530  
|

1535  
|

1540  
|

1545  
|

1550  
|

1555  
|

1560  
|

1565  
|

1570  
|

1575  
|

1580  
|

1585  
|

1590  
|

1595  
|

1600  
|

1605  
|

1610  
|

1615  
|

1620  
|

1625  
|

1630  
|

1635  
|

1640  
|

1645  
|

1650  
|

1655  
|

1660  
|

1665  
|

1670  
|

1675  
|

1680  
|

1685  
|

1690  
|

1695  
|

1700  
|

1705  
|

1710  
|

1715  
|

1720  
|

1725  
|

1730  
|

1735  
|

1740  
|

1745  
|

1750  
|

1755  
|

1760  
|

1765  
|

1770  
|

1775  
|

1780  
|

1785  
|

1790  
|

1795  
|

1800  
|

1805  
|

1810  
|

1815  
|

1820  
|

1825  
|

1830  
|

1835  
|

1840  
|

1845  
|

1850  
|

1855  
|

1860  
|

1865  
|

1870  
|

1875  
|

1880  
|

1885  
|

1890  
|

1895  
|

1900  
|

1905  
|

1910  
|

1915  
|

1920  
|

1925  
|

1930  
|

1935  
|

1940  
|

1945  
|

1950  
|

1955  
|

1960  
|

1965  
|

1970  
|

1975  
|

1980  
|

1985  
|

1990  
|

1995  
|

2000  
|

2005  
|

2010  
|

2015  
|

2020  
|

2025  
|

2030  
|

2035  
|

2040  
|

2045  
|

2050  
|

2055  
|

2060  
|

2065  
|

2070  
|

2075  
|

2080  
|

2085  
|

2090  
|

2095  
|

2100  
|

2105  
|

2110  
|

2115  
|

2120  
|

2125  
|

2130  
|

2135  
|

2140  
|

2145  
|

2150  
|

2155  
|

2160  
|

2165  
|

2170  
|

2175  
|

2180  
|

2185  
|

2190  
|

2195  
|

2200  
|

2205  
|

2210  
|

2215  
|

2220  
|

2225  
|

2230  
|

2235  
|

2240  
|

2245  
|

2250  
|

2255  
|

2260  
|

2265  
|

2270  
|

2275  
|

2280  
|

2285  
|

2290  
|

2295  
|

2300  
|

2305  
|

2310  
|

2315  
|

2320  
|

2325  
|

2330  
|

2335  
|

2340  
|

2345  
|

2350  
|

2355  
|

2360  
|

2365  
|

2370  
|

2375  
|

2380  
|

2385  
|

2390  
|

2395  
|

2400  
|

2405  
|

2410  
|

2415  
|

2420  
|

2425  
|

2430  
|

2435  
|

2440  
|

2445  
|

2450  
|

2455  
|

2460  
|

2465  
|

2470  
|

2475  
|

2480  
|

2485  
|

2490  
|

2495  
|

2500  
|

2505  
|

2510  
|

2515  
|

2520  
|

2525  
|

2530  
|

2535  
|

2540  
|

2545  
|

2550  
|

2555  
|

2560  
|

2565  
|

2570  
|

2575  
|

2580  
|

2585  
|

2590  
|

2595  
|

2600  
|

2605  
|

2610  
|

2615  
|

2620  
|

2625  
|

2630  
|

2635  
|

2640  
|

2645  
|

2650  
|

2655  
|

2660  
|

2665  
|

2670  
|

2675  
|

2680  
|

2685  
|

2690  
|

2695  
|

2700  
|

2705  
|

2710  
|

2715  
|

2720  
|

2725  
|

2730  
|

2735  
|

2740  
|

2745  
|

2750  
|

2755  
|

2760  
|

2765  
|

2770  
|

2775  
|

2780  
|

2785  
|

2790  
|

2795  
|

2800  
|

2805  
|

2810  
|

2815  
|

2820  
|

2825  
|

2830  
|

2835  
|

2840  
|

2845  
|

2850  
|

2855  
|

2860  
|

2865  
|

2870  
|

2875  
|

2880  
|

2885  
|

2890  
|

2895  
|

2900  
|

2905  
|

2910  
|

2915  
|

2920  
|

2925  
|

2930  
|

2935  
|

2940  
|

2945  
|

2950  
|

2955  
|

2960  
|

2965  
|

2970  
|

2975  
|

2980  
|

2985  
|

2990  
|

2995  
|

3000  
|

3005  
|

3010  
|

3015  
|

3020  
|

3025  
|

3030  
|

3035  
|

3040  
|

3045  
|

3050  
|

3055  
|

3060  
|

3065  
|

3070  
|

3075  
|

3080  
|

3085  
|

3090  
|

3095  
|

3100  
|

3105  
|

3110  
|

3115  
|

3120  
|

3125  
|

3130  
|

3135  
|

3140  
|

3145  
|

3150  
|

3155  
|

3160  
|

3165  
|

3170  
|

3175  
|

3180  
|

3185  
|

3190  
|

3195  
|

3200  
|

3205  
|

3210  
|

3215  
|

3220  
|

3225  
|

3230  
|

3235  
|

3240  
|

3245  
|

3250  
|

3255  
|

3260  
|

3265  
|

3270  
|

3275  
|

3280  
|

3285  
|

3290  
|

3295  
|

3300  
|

3305  
|

3310  
|

3315  
|

3320  
|

3325  
|

3330  
|

3335  
|

3340  
|

3345  
|

3350  
|

3355  
|

3360  
|

3365  
|

3370  
|

3375  
|

3380  
|

3385  
|

3390  
|

3395  
|

3400  
|

3405  
|

3410  
|

3415  
|

3420  
|

3425  
|

3430  
|

3435  
|

3440  
|

3445  
|

3450  
|

3455  
|

3460  
|

3465  
|

3470  
|

3475  
|

3480  
|

3485  
|

3490  
|

3495  
|

3500  
|

3505  
|

3510  
|

3515  
|

3520  
|

3525  
|

3530  
|

3535  
|

3540  
|

3545  
|

3550  
|

3555  
|

3560  
|

3565  
|

3570  
|

3575  
|

3580  
|

3585  
|

3590  
|

3595  
|

3600  
|

3605  
|

3610  
|

3615  
|

3620  
|

3625  
|

3630  
|

3635  
|

3640  
|

3645  
|

3650  
|

3655  
|

3660  
|

3665  
|

3670  
|

3675  
|

3680  
|

3685  
|

3690  
|

3695  
|

3700  
|

3705  
|

3710  
|

3715  
|

3720  
|

3725  
|

3730  
|

3735  
|

3740  
|

3745  
|

3750  
|

3755  
|

3760  
|

3765  
|

3770  
|

3775  
|

3780  
|

3785  
|

3790  
|

3795  
|

3800  
|

3805  
|

3810  
|

3815  
|

3820  
|

3825  
|

3830  
|

3835  
|

3840  
|

3845  
|

3850  
|

3855  
|

3860  
|

3865  
|

3870  
|

3875  
|

3880  
|

3885  
|

3890  
|

3895  
|

3900  
|

3905  
|

3910  
|

3915  
|

3920  
|

3925  
|

3930  
|

3935  
|

3940  
|

3945  
|

3950  
|

3955  
|

3960  
|

3965  
|

3970  
|

3975  
|

3980  
|

3985  
|

3990  
|

3995  
|

4000  
|

4005  
|

4010  
|

4015  
|

4020  
|

4025  
|

4030  
|

4035  
|

4040  
|

4045  
|

4050  
|

4055  
|

4060  
|

4065  
|

4070  
|

4075  
|

4080  
|

4085  
|

4090  
|

4095  
|

4100  
|

4105  
|

4110  
|

4115  
|

4120  
|

4125  
|

4130  
|

4135  
|

4140  
|

4145  
|

4150  
|

4155  
|

4160  
|

4165  
|

4170  
|

4175  
|

4180  
|

4185  
|

4190  
|

4195  
|

4200  
|

4205  
|

4210  
|

4215  
|

4220  
|

4225  
|

4230  
|

4235  
|

4240  
|

4245  
|

4250  
|

4255  
|

4260  
|

4265  
|

4270  
|

4275  
|

4280  
|

4285  
|

4290  
|

4295  
|

4300  
|

4305  
|

4310  
|

4315  
|

4320  
|

4325  
|

4330  
|

4335  
|

4340  
|

4345  
|

4350  
|

4355  
|

4360  
|

4365  
|

4370  
|

4375  
|

4380  
|

4385  
|

4390  
|

4395  
|

4400  
|

4405  
|

4410  
|

4415  
|

4420  
|

4425  
|

4430  
|

4435  
|

4440  
|

4445  
|

4450  
|

4455  
|

4460  
|

4465  
|

4470  
|

4475  
|

4480  
|

4485  
|

4490  
|

4495  
|

4500  
|

4505  
|

4510  
|

4515  
|

4520  
|

4525  
|

4530  
|

4535  
|

4540  
|

4545  
|

4550  
|

4555  
|

9606.ENSP00000348573  
9606.ENSP00000352572  
10116.ENSRNOP00000059746  
10090.ENSMUSP00000046129  
9615.ENSCAFP00000002847  
13616.ENSMODP00000010433  
13616.ENSMODP00000013265  
9258.ENSOANP00000021390  
9258.ENSOANP00000022853  
9258.ENSOANP00000023911  
9258.ENSOANP00000025554  
9258.ENSOANP00000025820  
9258.ENSOANP00000026533  
59729.ENSTGUP00000001490  
59729.ENSTGUP00000011566  
9103.XP\_003207115  
9103.XP\_003207389  
9031.ENSGALP00000011342  
9031.ENSGALP00000015160  
28377.ENSACAP00000015482  
28377.ENSACAP00000015489  
8364.ENSXETP00000007486  
69293.ENSGACP00000005460  
8090.ENSORLP00000017124  
8090.ENSORLP00000017141  
8090.ENSORLP00000017149  
99883.ENSTNIP00000020348  
99883.ENSTNIP00000020680  
31033.ENSTRUP00000033403  
31033.ENSTRUP00000038673  
7955.ENSDARP00000095611  
7955.ENSDARP00000099073  
7955.ENSDARP00000099417  
7739.JGI141041  
7668.XP\_001190091  
7668.XP\_783515  
10224.XP\_002741592  
7176.CPIJ005546-PA  
7176.CPIJ005547-PA  
7176.CPIJ005548-PA  
7159.AAEL006395-PA  
7165.AGAP010655-PA  
7227.FBpp0099786  
7070.XP\_972088  
7425.XP\_001605300  
7460.XP\_001122485  
7460.XP\_392766  
121224.XP\_002427616  
7029.XP\_001946305  
6412.189990  
6183.Smp\_051510  
6085.XP\_002159594  
45351.JGI27057  
109871.jgi|Batde5|22429|fgenes  
645134.SPPG\_07328T0

Homo sapiens  
Homo sapiens  
Rattus norvegicus  
Mus musculus  
Canis lupus familiaris  
Monodelphis domestica  
Monodelphis domestica  
Ornithorhynchus anatinus  
Ornithorhynchus anatinus  
Ornithorhynchus anatinus  
Ornithorhynchus anatinus  
Ornithorhynchus anatinus  
Ornithorhynchus anatinus  
Taeniopygia guttata  
Taeniopygia guttata  
Meleagris gallopavo  
Meleagris gallopavo  
Gallus gallus  
Gallus gallus  
Anolis carolinensis  
Anolis carolinensis  
Xenopus (Silurana) tropicalis  
Gasterosteus aculeatus  
Oryzias latipes  
Oryzias latipes  
Oryzias latipes  
Tetraodon nigroviridis  
Tetraodon nigroviridis  
Takifugu rubripes  
Takifugu rubripes  
Danio rerio  
Danio rerio  
Danio rerio  
Branchiostoma floridae  
Strongylocentrotus purpuratus  
Strongylocentrotus purpuratus  
Saccoglossus kowalevskii  
Culex quinquefasciatus  
Culex quinquefasciatus  
Culex quinquefasciatus  
Aedes aegypti  
Anopheles gambiae  
Drosophila melanogaster  
Tribolium castaneum  
Nasonia vitripennis  
Apis mellifera  
Apis mellifera  
Pediculus humanus corporis  
Acyrthosiphon pisum  
Helobdella robusta  
Schistosoma mansoni  
Hydra magnipapillata  
Nematostella vectensis  
Batrachochytrium dendrobatidis  
Spizellomyces punctatus DAOM BR117

----------------------------------------------------------------------------------------------------------MEDEERQKKLEAGKAKLAQFRQRKA-----QSDGQSPSKKQKKKRKTSSSKHDVSAHHDLNIDQSQCNEMYINSSQRVESTVIPESTIMRTLHSGEITSHEQGFSVELESEISTTADDCSSEVNGCSFVMRTGKPTNLLREEEFGVDDSYSEQGAQDSPTHLEMMESELAGKQHEIEELNRELEEMRVTYGTEGLQQLQEFEAAIKQRDGIITQLTAN-LQQARREKDETMRE--FLELTEQSQKLQIQFQQLQASETLRNSTHSSTAADLLQAKQQILT-HQQQLEEQDHLLEDYQKKKEDFTMQISFLQEKIKVYEMEQDKKVENSNKEEIQEKETIIEELNTKIIEEEKKTLELKDKLTTADKLLGELQEQIVQKNQEI---KNMKLELTNSKQKERQSSEEIKQLMGTVEELQKRNHKDSQFETDIVQRMEQETQRKLEQLRAELDEMYGQQIVQMKQELIRQHMAQMEEMKTRHKGEMENALRSYSNITVNEDQIKLMNVAINELNIKLQDTNSQKEK----LKEELGLILEEKCALQRQLEDLVEELSFSREQIQRARQTIAEQESKLNEAHKSL-STVEDLKAEIVSASESRKELELKHEAEVTNYKIKLEM--LEKEKNAVLDRMAESQEAELERLRTQLLFSHEEELSKLKEDLEIEHRINIEKLKDNLGIHYKQQIDGLQNEMSQKIETMQFEKDNLITKQNQLILEISKLK---DLQQSLVNSKSEEMTLQINELQKEIEILRQEEKEKGTLEQEVQELQLKTELLEKQMKEKENDLQEKFAQLEAENSILKDEKKTLEDMLKIHTPVSQEERLIFLDSIKSKSKDSVWEKEIEILIEENEDLKQQCIQLNEEIEKQRNTFSFAEKNFEVNYQELQEEYACLLKVKDDLEDSKNKQELEYKSKLKALNEELH-LQRINPTTVKMKSSVFD---EDKTFVAETLEMGEVVEKDTTELMEKLEVTKREKLELSQRLSDLSEQLKQKHGEISF-LNEEVKSLKQEKEQVSLRCRELEIIINHNRAENVQSCDTQVSSLLDGVVTMTSRGAEGSVSKVNKSFGEESKIMVEDKVSFENMTVGEESKQEQLILDHLPSVTK--------------ESSLRATQPSENDKLQKELNVLKSEQNDLRLQMEAQRICLSLVYSTHVDQVREYMENEKDKALCSLKEELIFAQEEKIKELQKIHQLELQTMKTQETGDEGKPLHLLIGKLQKAVSEECSYFLQT-LCSVLGEYYTPALKCEVNAEDKENSGDYISENED--PELQDYRYEVQDFQENMHTLLNKVTEEYNKLLV-----LQTRLSKIWGQQTDGMKLEFGEENLPKEETEFLSIHSQMTNLEDIDVN--HKSKLSSLQDLE--KTK-LEEQVQELESLISSLQQQLKETEQNYEAEIHCLQKRLQAVSESTVPPSLPVDSVVIT---ESDAQRTMYPGSCVKKNIDGTIEFSGEFGVKEETNIVKLLEKQYQ--EQLEEEVAKVIVSMSIAFAQQTELSRISGGKENTASSKQAHAVCQQEQHYFNE-MKLSQDQIGFQTFETVDVKFKEEFKPLSKELGEH-GKEILLSNSD-PHDIPESKDCVLTISEEMFSKDKTFIVRQSIH-DEISVSSMDASRQLMLNEEQLEDMRQELVRQYQEHQQATELLRQAHMRQMERQREDQEQLQEEIKRLNRQLAQRSSIDNENLVSERERVLLEELEALKQLSLAGREKLCCELRNSSTQTQ-------NGNENQGEVEEQTFKEKELDRKPEDVPPEI-LSNERYALQKANNRLLKILLEVVKTTAAVEETIGRHVLGILDRSSKSQSS-ASLIWRSEAEASVKSCVHEEH-------TRVTDESIPSYSGSDMPRNDINMWSKVTEEGTELSQRLVRSGFAGTEIDPENEELMLNISSRLQAAVEKLLEAISETS----------------------SQLEHAKVTQTELMRESFRQKQEATESLKCQEELRERLHEESRAREQLAVELSKAEGVIDGYADEKTLFERQIQEKTDIIDRLEQELLCASNRLQELEAEQQQIQEERELLSRQKE---AMKAEAGPVEQQLL-------------QETEKLMKEKL---EVQCQAEKVRDDLQKQVKALEIDVEEQVSRFIELEQEKNTELMDLRQQNQALEKQLE--KMRKFLDEQAIDR--EHERDVFQQEIQKLEQQLKVVPRFQPISEHQ-------TREVEQLANHLKEKTDKCSELLLSKEQLQRDIQERNEEIEKLEFRVRELEQA--LLVSADTFQKVEDRKHFGAVEAKPELSLEVQLQAERDAIDRKEKEITNLEEQLEQFREELENKNEEVQ-QLHMQLEIQKKE-------------STTRLQELEQENKLFKDDMEKLGLAIKESDAMST---QDQHVLFGKFAQIIQEKEVEIDQLNEQVTKLQQQLKI-----------------------------------TTDNKVIEEKNELIRDLETQIECLMSDQECVKRNREE--EIEQLNEVIEKLQQELANIGQKTSMNAHSLSEEADSLKHQLDVVIAEKLALEQQVETANEEMTFMKNVLKETNFKMNQLTQELFSLKRERESVEKIQSIPENSVNVAIDHLSKDKPELEVVLTEDALKSLENQTYFKSFEENGKGSIINLETRLLQL-ESTVSAKDLELTQCYKQIKDMQEQGQFETEMLQKKIVNLQKIVEEKVAAALVSQIQLEAVQE----YAKFCQD--N--QTISSEPERTNI-QNLNQLREDELGSDISALTLRISELESQVVEMHTSLILEKEQVEIAEKNVLEKEKKLLELQKLLEGNEKKQREKE------------KKRSPQDVEVLKTTTELFHSNEESGFFNELEALRAESVATKAELASYKEKAEKLQEELLVKETNMTSLQKDLSQVRDHLAEAKEKLS-ILEKEDETEVQESKKAC-----MFEPLPIKLSKSIASQTDGTLKISSS-NQTPQILVKNAGIQINLQSE--CSS---EEVTEIISQFTEKIEKMQELHAAEILDMESRHISETETLKREHYVAVQLLKEECGTLKAVIQCLRSKEGSSIPELAHSDAYQTREICSSDSGSDWGQGIYLTHSQGFDIASEGRGEES-ESATDSFPKKIKGLLRAVHNEGMQVLSLTESPYSDGEDH--------SIQQVSEPWLEERKAYINTISSLKDLITKMQLQREAEVYDSSQSHESFSDWRGELLLALQQVFLEERSVLLAAFRTELTALGTTDAVGLLNCLEQRIQEQGVEYQAAMECLQKADRRSLLSEIQALHAQMNG----RKITLKREQESEKPSQELLEYNIQQKQSQMLEMQVELSSMKDRATELQEQLSSEKMVVAELKSELAQTKLELETTLKAQHKHLKELEAFRLEVKDKTDEVHLLNDTLASEQ-----KKSRELQWALEKEKAKLGRSEERDKEELEDLKFSLESQKQRNLQLNLLLEQQKQLLNESQQKIESQRMLYDAQLSEEQGRNLELQVLLE---------------SEKVRIREMSSTLDRERE----LHAQLQSSDGTGQSRPPLPSEDLLKELQKQLE-----EKHSRIVELLNETEKYKLDSLQTRQQMEKDRQVHRKTLQTEQEANTEGQKKMHELQSKVEDLQRQLEEKRQQVYKLDLEGQR-------------------LQGIMQEFQKQELEREEKRESRRILYQNLNE-PTTWSLTSD---RTRNWVLQQKIEG-ETKESNYAKLIEMNG-GGTGCNHELEMIRQKLQCVASKLQVLPQKASERLQF-ETADDEDFIWVQENIDEIILQLQKLTG---QQGEEPSL-VSPSTSCGSLTERLLRQNAELTGHISQLTEEKNDLRNMVMKLEEQIRWYRQTGAG--RDNSSRFSLNGGANIEAIIASE-KEVWNREKLTLQKSLKRAEA-------------------EVYKLKAELRNDSLLQTLSPDS---------EHVTLKRIYGKY-L-RAESFRKALIY-QKKYLLLLL-GGFQECEDATLALLARMG--------GQPAFTD-LEVITNRPKGF---------------------------------------------------TRFRSAVRVSIAI--SRMKFLVRRWHRVTG---------------------SVSININRDGFGLNQGAE-KTDSFYHSSGGLELYGEPRHTTYRSR-SDLDYIRSPLP-FQNRYPGTPADFNPGSLACSQLQN-------------YDPDRALTDYITRLEALQRRLG-TIQSGST---TQFHAGMRR-------------------------------------------------------------------------------------------------------------------  
-----------------------------------------------------------------------------------------------------------------------------------------------------------------------------------------------------------------------------------------------------------------------------------------------------------------------------------------------------------------------------------------------------------------------------------------------------------------------------------------------------------------------------------------------------------------------------------------------------------------------------------------------MEVEQEQRRRKVEAGRTKLAHFRQRKTKGDSSHSEKKTAKRKGSAVDASVQEESPVTKEDSALC-GGGDICKSTSCDDTPDGAGGAFAAQPEDCDGEKREDL--EQLQQKQVNDHPPEQCGMFTVSDHPPEQHGMFTVGDHPPEQRGMFTVSDHPPEQHGMFTVSDHPPEQRGMFTISDHQPEQRGMFTVSDHTPEQRGIFTISD--HPAEQRGMFTKECEQECELAITDLESGREDEAGLHQSQAVHGLELEALRLSLSNMHTAQLELTQANLQKEKETALTELREMLNSRRA------------------------QELALLQSRQQHELELLREQHAREKEEVVLRCGQEAAELKEKLQSEMEKNAQIVKTLKEDWESEKDLCLENLRKELSAKHQSEMEDLQNQFQKELAEQRAELE-------------KIFQDKNQAERALRN-LESHHQAAIEKLREDLQSEHGRCLEDLEFKFKES-EKEKQLELENLQASYEDLKAQSQEEIRRLWSQLDSARTSRQELSELHEQLLARTSRVEDLEQLKQ-------REKTQHESELEQLRIYFEKKL--------------------------RDAEKTYQEDLTLLQQRLQGAREDALLDSVEVGLSCVGLEEKPEKGRKDHVD--ELEPERHKESLPRFQAELEESHRHQLEALESPLCIQHEGHVSDRCCVETSALGHEWRLEPSEGHSQELPWV-HLQGVQDGDLEADTERAARVLGLETEHKVQLSLLQT------ELKEEIELLKIENRNLYGKLQHETRLK-----DDLEKVKHNLIEDHQKELNNAKQKTELMKQEFQRKETDWKVMKEELQREAEEKLTLMLLELREKAESEKQTIINKFELREAEMRQLQDQQAAQILDLERSLTEQQGRLQQLEQDLTSDDALHCSQ---CGREPPTAQDGELAALHVKEDCALQLMLARSRFLEERKEITEKFSAEQDAFLQEAQEQHARELQL----LQERHQQQLLSVTAELEARHQAALG--------ELTASLESKQGALLAAR---VAELQTKHAADLGALETRHLSSLDSLESCYLSEFQTIREEHRQALELLRADFEEQLWKKDSLHQTILTQELEKLKRKHEGELQSVRDHLR-TEVSTELAGTVAHELQGVHQGEFGSEKKTALHEKEETLRLQSAQAQPFHQEEKESLSLQLQKKNHQVQQLKDQVLSLS---HEIEEC-------RSELEVLQQRRERENREGANLLSMLKADVNLSHSERGALQDALRRLLGLFGETLRAAVTLRSRIGERVGLCLDDAGAGLALSTAPALEETWSD-------VALPEL-----DRTLSEC--AEMSSVAEIS---------------SHMRESFLMSPESVRECEQPIRRVFQSLSLAVDGLMEMALDSS----------------------RQLEEARQIHSRFEKEFSFKNEETAQVVRKHQELLECLKEESAAKAELALELHKTQGTLEGFKVETADLKEVLAGKEDSEHRLVLELESLRRQLQQAAQEQAALREECTRLWSRGE---ATATDAEAREAALR-------------KEVEDLTKEQS---ETRKQAEKDRSALLSQMKILESELEEQLSQHRGC-AKQAEAVTALEQQVASLDKHLR--NQRQFMDEQAAER--EHEREEFQQEIQRLEGQLRQAA-KPQPWGPRDSQQAPLDGEVELLQQKLREKLDEFNELAIQKESADRQVLMQEEEIKRLE------------EMNINIRKKVAQLQEEVEKQKNIVKGLEQDKEVLKKQQMSSLLLASTLQSTLDAGRCPEPPSGSPPE-GPEIQLEVTQRA-------------LLRRESEVLDLKEQLEKMKGDLESKNEEILHLNLKLDMQNSQTAVSLRELEEENTSL--------------------------------------------------------KVIYTRSSEIEELKATIENLQENQKRLQKEKAE--EIEQLHEVIEKLQHELS--LMG------PVVHEVSDSQAGSLQSELLCSQAGGPRGQALQGELEAALEAKEALSRLLADQERRHSQALEALQQRLQGAEEAAELQLAELERNVALREAEVE------DMASRIQEFEAALKAKEATIAERNLEIDAL-NQRKAAHSAELEAVLLALARIRRALEQQPLAAGAAPPELQWLRAQCARLSRQLQVLHQRFLR----CQVELDR--RQARRATAHTRVPGA-HPQPRMDGGAKAQVTGDVEASHDAALEPVVPDPQGDLQPVLVTLKDAPLCKQEGVMSVLTVCQRQLQSELLLVKNE----------MRLSLEDGGKG-KEKVLEDCQLPKVDLVAQVKQLQEKLNRLLYSMTFQNVDAADTKSLWPMASAHLLESSWSDDSCDGEEPDISPHIDTCDANTATGGVTDVIKNQAIDACDANTTPGGVTDVIKNWDSLIPDEMPDSPIQEKSECQDMSLSSPTSVL--GGSRHQSHTAEAGPRKSPVGMLDLSSWSSPEVLRKDWTLEPWPSLPVTP-HSGALSLCSADTSLGDRADTSLPQTQGPGLLCSPGVSAAALALQWAESPPADDHHVQRTAVEKDVEDFITTSFD-SQETLSSPPPGLEGKADRSEKSDGSGFG-ARLSPGSGGPEAQ---TAGPVTPASISGRFQPLPEAMKEKEVRPKHVKALLQMVRDESHQILAL-SEGLAPPSG-EPHPPRKEDEIQDISLHGGKTQEVPTACPDWRGDLLQVVQEAFEKEQEMQGVELQPRLSGSDLGGHSSLLERLEKII--REQGDLQEKSLEHLRLPDRSSLLSEIQALRAQLRMTHLQNQEKLQHLRTALTSAEARGSQQEHQLRRQVELLAYKVEQEKCIAGDLQKTLSEEQEKANSVQKLLAAEQTVV-----RDLKSDLCESRQKSEQLSRSLCEVQQEVLQLRSMLSSKENELKAALQELESEQGKGRALQSQLEEEQLRHLQRESQSAKALEELRASLE---------------TQRAQSSRLCVALKHEQTAKDNLQKELRIEHSRCEALLAQERSQLSELQKDLAA-----EK-SRTLELSEALRHERLLTEQLSQRTQEACVHQDTQAHHALLQKLKEEKSRVVDLQAMLEKVQQQALHSQQQLEAEAQKHCEALRREKEVSATLKSTVEALHTQKRELRCSLEREREKPAWLQAELEQSHPRLK-EQEGRK---AARRSAEARQSPAAAEQWRKWQRDKEKLR-ELELQRQRDLHKIKQLQQTVRDLES-KDEVPGSRLH-LGSARRAAGSDADHLREQQRELEAMRQRLLSAARLLTS-FTSQAVDRTVNDWTSSNEKAVMSLLHTLEELKSDLSRPTSSQKKMAAELQFQFVDVLLKDNVSLTKALSTVTQEKLELS-RAVSKLEKLLKHHLQKGCSPS-------------RSERSAWKPDETAPQSSLRRPDPGRLPPAASEEAHTSNVKMEKLYLHY-L-RAESFRKALIY-QKKYLLLLI-GGFQDSEQETLSMIAHLG--------VFPSKAE---RK-ITSRPF---------------------------------------------------TRFRTAVRVVIAI--LRLRFLVKKWQEVDRKGA------------------LAQGKAPR--PGPRAR---QPQSP-PRTRESPPTRDVPS-GHTRDP-ARGRRLAAAASPHSGG-RATPSP-NSRLERS-LTASQ------------DPEHSLTEYIHHLEVIQQRLGGVLPDSTSKKSCHPMIKQ---------------------------------------------------------------------------------------------------------------------  
--------------------------------------------------------------------------------------------------------------------------LARFRQRKA-----QCDGDIP-KKQKKKR-TPSSKQDSS----LHTDQ-QSAEICSESSQRADSAGSPDSSSAEKA------KHGQVFAADPESEISTTADECSSE------------------EEDFSIDDSYSEQGAQSSQTCLQMGENELAEKQHEVEELTQELEEMRASFGTEGLKQLQEFEAAIKQRDGIITQLTAN-LQQARKEKDDTTIE--FLELTEQSQKLQIQFQHLQANETLQNSTLSRTATDLLQAKRQIFT-LQQQLQ-------DYQKKEEDVQAQISFLQEKIRMLEMEKDRKVESLNTKEIEEKQAIIEELNTRVIEEERKTAELENKVTTADELLGQLQGQVTEKDQEI---KSLKLELVNSQQNERKCSEEIKELMRTVEELQKRNLKDSQLGTGTVRRVEQETQRKLSLLQAELDEMYGKQIVQMKQELMKQHMSQIEELKSQHKRDLENTLKSDASTTVSKEQVNLMSAAISELNVRLQDTHAQKEK----LKGELGAALGEKSALQSQFEDLLEEVRFLREQVQKARQTIAEQENRLSDARKSL-STVEDLKAEIVAASESRKELEVKHEAEITNYKIKLEM--LEKEKNAVLDRMAESQEAELERLRTQLLFSHEEELSKLKEDLEVEHRINVEKLKDNLGIHYKQQIDGLQNEMNRKMETMQCETDNLITRQNQLILENSKLR---DLQEHLVNSKSEEMNLQINELQKEIEILRREEKEKGSLEQEVQGLQLKTEQLERQLKEKGDDLQEKCAQLDAENSVLKDEKKALEDMLKMYTPLDQE------DPSTRKSEDPRWQKEVGMLRKENEDLQQQCLHLSEEIEKQKNTFAFAEKNFEVNYQELQREYTCLLKIRDDLEDTLAKQALQYESKLSALEEELHSQRGSPTTPPKGENS--------GTFPLEPLEVGEVVEKDTTELMEKLEVTRREKLELSEKVSGLSEQLKQTHGTIDS-LSEEVRALKQEKEQLLLRCGELELLTNANGTENASVCPVQRSPYQAGPVMGTVRDSQGPISKVSEDLAEESKPMTEDKIPFK------ESGREPLLL---PTRTK--------------KPSRVTVEPCESEKLQQELYALKAEQDDLRLQMEAQRLCLFVVYSTHADQVRAYMEKEREEALCSLKDELVSAQQKKIDELHKIHQCQLQHVKVQETGNE--PLQVLIERLQKAVSEKCFHISKT-LNNVFDECYTP-LKCEMNIEEKENSGVYTSQNQS--PELREYRYEVQDFQENMQILLGKVTEECSKLLV-----LQTRLNKINEQQVDGVTFEFAEENVAEVEAGLLSGRSQSG-LQGAVFS--SEVKRNTVNHES--KVSEYERQVQELQSLVAAGQLQLKETEANYRAEIQCLQERLQAVSEATVQPSLSMDSVVFK---GSGAQKPVYCGSCLREQVDGTAEFSDGFEVRQETNMVKLMEKQYQ--ERLEEEIAKVIVSMSIAFAQQTELSRLSEEKENVIQSEQAHALCSQNEHHLND-IT-SQCQVGLQTVEATGKNLKEEFKPLSKELGEY-RKAVPLSNHDDLDDVLKSEEHGLTISEEIFSKDETFLVRNSSP-DEVLVSNMDTSRQLILN-EQLEDMRQELVRQYEEHQQATELLRQAHMQQMERQREDQEQLQEEIKRLNQQLTQRSSIDTEHVVSERERVLLEELEALKRLSLAGRKELCCELQHSSTQTQ-------DGNDNQ-EVEEQTLKDKTLERKPEDIPLDINLSNERYALKKANNRLLKILLDVVKTTSAAEETIGRHVLGILDRSSKSQPA-TSLLWRSETEASATVCVQEEC-------ARAMDESVPSYPGSAIATHDS-VWSKVTEEGAELSQRLVRSGFAGPVIDPENEELMLNISSRLQAAVEKLLEAISETS----------------------SQLEHAKVTQTELMRESFRQKQEATESLHCLEELRERLQEESRAREQLAVELNKAESVIDGYSDEKTLFERQIQEKTDIIEHLEQELLCTNNRLQELESDQRRMEEERELLSRQRE---AMRADAGPVEQQFL-------------QETEKLMKEKL---EVQCQAEKVRGDLQKQVKALEIDVEEQVSRFIELEQEKNAELIDLRQQSQALEKQLE--KMRKFLDEQAIDR--EHERDVFQQEIQKLEHQLKAVPRLLPVSEHQ-------TREVEQLTNHLKEKTDKCSELLLSKEQLQRDVQERNEEIEKLECRVRELEQA--LLATADPFPKVEEQKRPGAVEVDPELSLEVQLQAERDATDRKQKEITNLEEQLEQFREELENKNEEVQ-ELLMQLEIQKKE-------------STTRLQELQQENRLFKEEIEKLGFTMKESDSLST---QDQPMLFGKFAQLIQEKEIEIDRLNEQFIKLQQQLKL-----------------------------------TTDNKVIEEQKDQIRDLETQIECLMSEQELMKKQRAE--EAEQLNGVIEKLQQELVSTEQKR-EGARALPEDEESFKHQLDRVTVEKLGLEKQVETTNQELTDLKNVLKEINFKMDHMTQALCNLNK------ELPNVPKESVHVTVHELGCDKLQPEGASAQDASQPLESQTSLMCLQESTKASQI-LEIKSLPL-QGSGSTKDFELAQCHKQIDTIQEQDQSEIGMLQKKITNLQKIL-EKFSAALVSWVQMEAAQD----RVQLYQE--KQTQTVSSAPERTDT-QNVNCLAENNLESHVPTLAVRPAELESRVAEVLSDIMSEK-HMETVGKNASETEKEVVALQKLLEEAKKRL-KEE------------REQSPRDRE--------------------------------------------------VKESYITSLQKDLGQVKDQLTEAKEKLSHYLEKENGTGEQESRRVP-----IPESPSVEGGGG-SGTMERTGKVSSS-NQTPQILVRHAGVQIDLQSE--RSQ---EELRDTINQFTEKMERIQELHAAEILDMESRHILETESLKKEHSVAIQLLTKECETLKDMTQYLRCKKGSSISDLADSVAYQSREVYSSDSESDWGQ------SQGFDTATEGR-EEG-ETS-DLFPKKIKGLVKAVHSEGMQVLSLSNPLCDDGEDR--------SVQQLSESWLKERQTYLNTISSLKDLISKMQVQRETEVYDSCH----FSDWRGELLLACQRVFIKERSVLLATFQTELTSLSTRDADGVLNSLEQRIQEQGIEYQTAMDCLQKADRRSLLTEIEDLRAQING----RKMTLEREQEIEKPSQELLDCNLQQKQSHVLEMQLELGSLRDRAAELQEQLSSEKMVVAELKSELAQAKLELGTTLKAQHKRLKELEAFRSEIKEKTDEIHFLSDTLAREQ-----KKSLELQWALEKEKAKSGHHEEWEKEELEDLKFSLEDQKRKNTQLNLLLEQQKQLLNESQQKMESQKMLHDAQLSEEQGRNLGLQALLE---------------SEQVRVQEMKSTLDKERE----LYAQLQSRDDGGQPLPVLPSEDLLKELQKQLE-----EKHTRIVELLSETEKYKLDSLQTRQQMEKDREVHQKTLQTEQEANTQGQKKMQELQSKVEELQRQLQEKRQQVYKLDLEGKR-------------------LQGIMQEFQKQELEPEEKRGSRGLVYQNLNE-PASWTFTDD---RTRNWVLQQKMG--ETKDTNFTKLIEING--ELDHNHDLEVIRQTLQHVASKLQHVAQKACNRLQF-ETASDDAFIWIQENIDGIILQLQKLTG---QPGDEHSL-APPSSSCGSLTESLMRQNTELTRLINQLTEEKNTLRSIVVKLEELNRCHWHTGAG--RDCCSRFSFIDPADIEAIIASE-KEVWNREKLSLQKALKRAEA-------------------KVYKLKAELRNDALLRSLSPDS---------EHAALQKIYNKY-L-RASSFRKALIY-QKKYLLLLL-GGFQECEDATLGVLARMG--------GHPALKD-PKTITSHPKGF---------------------------------------------------TRFRSAVRVSIAI--SRMKFLVRRWQQVTS---------------------TSSININRDGFGLSPGIE-KTDPFYHSSGGLELYGEPRHTTYRSR-FDLDYPRSPLP-LQNRYPGAP-DLNSTSMASSQLHQ-------------YNPDKSLTDYVTRLEALKRRLG-AIQSGST---TQFHFGMRR-------------------------------------------------------------------------------------------------------------------  
----------------------------------------------------------------------------------------------------------MEDEERQRKLAAGKAKLARFRQRKA-----QYDGDIP-KKQKKKR-TSSSKHDSS----LHTDQ-QSGELCSESSQRVDLAGNPDCSGPER-------KHGQVFSAEPESEISTTADECSSE------------------EEEFSLDDSSSEQGAQSSQTCLQMVEKELAEKQHDIEELTQELEEMRASFGTEGLKQLQEFEAAIKQRDGIITQLTAN-LQQARREKDDTMVE--FLELTEQSQKLQIQFQHLQANETLQNSTLSRTATDLLQAKRQIFT-QQQQLQ-------DYQKKEEDLQAQISFLQEKLRAFEMEKDRKIENLNAKEIQEKQALIDELNTRVVEEEKKTVELKNKVTTADELLGGLHEQLTQRNQEI---QSLKLELGNSQQNERKCSEEIKELMRTVEELQKRNLKDSWLETSAVRRVEQETQRKLSHLQAELDEMYGKQIVQMKQELINQHMSQIEELKSQHKREMENTLKSDTNAAISKEQVNLMNAAINELNVRLQETHAQKEE----LKGELGVVLGEKSALQSQSNDLLEEVRFLREQVQKARQTIAEQENRLSEARKSL-STVEDLKAEIVAASESRKELELKHEAEITNYKIKLEM--LEKEKNAVLDRMAESQEAELERLRTQLLFSHEEELSKLKEDLEVEHRINIEKLKDNLGIHYKQQIDGLQNEMNRKMESMQCETDNLITQQNQLILENSKLR---DLQECLVNSKSEEMNLQINELQKEIEILKQEEKEKGTLEQEVQELQLKTEQLEKQLKEKEDDLQEKCAQLDAENNILKEEKRVLEDKLKMYSPSEQEERSIAVDPSTSKSADSRWQKEVAMLRKETEDLQQQCLYLNEEIEKQRNTFAFAEKNFEVNYQELQREYTCLLKIRDDLEATQTKQALEYESKLRALEEELLSKRGNPAAP-KGKSS--------GIFPSETLEIGEVVEKDTTELMEKLEVTKREKLELSEKVSGLSEQLKQTHCTINS-LSAEVRALKQEKEQLLLRCGELELLANPSGTENAAVCPVQMSSYQAGLVMGKVGDSGGSISKISKDLAEESKPMIEDKIPFK------ESGREQLLL---PTRAQ--------------KPSHATVEPCESEKLQQELHALKAEQDDLRLQMEAQRICLFVVYSTHADQVRAHMEKEREEALCSLKDELISAQQKKIDELHKMHQCQLQNFKIQETGDE--PLQVLIERLQQAVSEKCFHISKT-LNNVFDECYTP-LKCEMNIEEKENSGVYTSQNQS--PELQEYRYEVQDFQESMQVLLGKVTEECRKLSG-----LQTRLGKIHEQQTDGVALEFAEQNAAEEEAGLLSGCSQSA-LQSTDVS--LESKVSSLPASE--KSRECERQVQELQSPVAAGQLQLTETEANHRAETESLQQRLEAVSEAPVQPSLSIDSVVFK---GSGAQKPVYCGSCLRECVDGTAKFSDRFEVRQETNMVNLMEKQYQ--ERLEEEIAKVIVSMSIAFAQQTELSRLSEGKENTIQSEQAHTLCSQNKHQLND-IT-SQSQVGLQTFEATDKNFKEEFKPLSKELGEY-RKAVPLSSHDDLDDILKSEEHGLAISEEIFSKDETFIVRKSMH-DEVLVSSMDTSRQLILN-EQLEDMRQELVRQYEEHQQATEMLRQAHMQQMERQREDQEQLQEEIKRLNEQLAQKSSIDTEHVVSERERVLLEELEALKQLPLAGRKELCCELRHSSTQTQ-------DGHDDQ-EVEEQTLKDKTLERSPEDALLDRNLSNERYALKKANNRLLKILLEVVKTTSAAEETIGRHVLGILDRSSKGQTA-SSLLWRSEADASATTCAPEDC-------ARAMDESIPSYPGTAIATHDS-IWSKVTEEGAELSQRLVRSGFAGPVIDPENEELMLNISSRLQAAVEKLLEAISETN----------------------TQLEHAKVTQTELMRESFRQKQEATESLHCLEELRERLQEESRAREQLAEELNKAESVIDGYSDEKTLFERQIQEKTDIIEHLEQEVLCMNNRLQELESDQRRVEEERQLLCRQRE---AMRAEAGPVEQQFL-------------QETEKLMKEKL---EVQCQAEKVRGDLQKQVKALEIDVEEQVSRFIELEQEKNAELTDLRQQSQALEKQLE--KMRKFLDEQAIDR--EHERDVFQQEIQKLEHQLKAVPRIQPVSEHQ-------AREVEQLTNHLKEKTDRCSELLLSKEQLQRDIQERNEEIEKLECRVRELEQA--LLASAEPFPKVEDQKRSGAVEADPELSLEVQLQVERDATDRKQKEITNLEEQLEQFREELENKNDEVQ-ELLMQLEIQRKE-------------STTRLQELQQENRLFKDEIEKLGFAMKESDSVST---RDQPMLFGKFAQLIQEKEIEIDRLNEQFIKLQQQLKL-----------------------------------TTDNKVIEEQKEQIQDLETQIERLMSEREHEKKQREE--EVEQLTGVVEKLQQEVVSTEQQR-EGARTLPEDEESFKHQLDKVTAEKLVLEQQVETTNQVMTHMNNVLKEINFKMDQITQSLCNLNKECASNEELPSLPKESVHMTVHELGSDNLQPEDAPAQDVTKPLEKQTSLTRLQKSPEASRT-QEIESLAS---SVGAKDVELTQCREQTETIQEQAQSETDRLQKKLTDLQRSL-EKFAAALVSQVQMEAAQE----YVPFHQE--K--QPVSSAPGSTDI-QNANGLTGASTESLIPTVTLRLAEVESRVAEVHSGTMSEK-LVGIVGGNASETEKRVIELQKLLEEAEERP-EEG------------GEQSSRDGE--------------------------------------------------VRESYMTSLQKDLGQVKDPLTEAKEKLSYSLEKEKRTGEQESREAP-----IPEPPSVEVGGC-SGLTERTDKVSSSGNQTLQILLRDAAIQTDLQSE--SSQ---EEVRDTINQLTKKMEHIQELHAAEILDMESRHILETESLKKEHYVAIQLLTKECETLKEMTQCLRCKEGSSIPELADSVAYQSREVYSSDSESDWGQ------SQGFDTAIEGR-EEG-ETSADLFPKKIKGLVKAVHSEGMQVLSLSSPLCDDGEDR--------SIQQLSESWLKERQAYLNTISSLKDLISKMQVRRETEVYDRCH----LSDWRGELLLACQRVFIKERSVLLATFQTELTSLSTRDVDGLLNSLEQRIQEQGIEYHTAMDCLQKADRRSLLAEIEDLRAQING----GKMTLEREQGTEKSSQELLDCSMQQKQS--LEMQLELSSLRDRAAELQEQLSSEKMVVAELKSELAQAKLELGTTLKAQHKRLKELEAFRSEVKEKTDEIHFLSDTLAREQ-----KNSLELQWALEKEKARSGHHEGREKEELEDLKFSLEDQKRRNTQLNLLLEQQKQLLNESQQKIESQKMLHDAQLSEEQGRNLGLQALLE---------------SEQVRIQEMKSTLDKERE----LYAQLQSREDGGQPPPALPSEDLLKELQKQLE-----EKHSRIVELLSETEKYKLDSLQTRQQMEKDRQVHQKTLQTEQEANTQGQKKMQELQSKVEELQRQLQEKRQQVYKLDLEGKR-------------------LQGLMQEFQKQELEPEEKPGSRGLVDQNLNE-PATWNFTDD---RTRNWVLQQKMG--EAKDRNFTKLIEING-GELDHNHDLEMIRQTLQHVASKLQHVAQKACSRLQF-ETAGDDAFIWIQENIDGIILQLQKLTG---QPGDEHSL-GPPSSSCGSLTESLMRQNTELTRLINQLTEEKNTLRSIVIKLEELNRCYWHTGAS--RDCCSRFSFIDPADIEAIIASE-KEVWNREKLSLQKALKRAEA-------------------KVYKLKAELRNDALLRNLGPDT---------DHAALQKIYNKY-L-RASSFRKALIY-QKKYLLLLL-GGFQECEDVTLGVLARMG--------GHLALKD-SKTITNHPKAF---------------------------------------------------SRFRSAVRVSIAI--SRMKFLVRRWQQVTS---------------------TSSININRDGFGLSPGIE-KTDPFYHSPGGLQLYGEPRHTMYRSR-FDLDYPRSLLP-LQNRYPGTPGDLNSISMASSQLHQ-------------YNPDKSLTDYVTRLEALRRRLG-AIQSGST---TQFHFGMRR-------------------------------------------------------------------------------------------------------------------  
----------------------------------------------------------------------------------------------------------KESDSKDTCFFHSDGKLAQFRQRKA-----QSDGQNPSKKQKKKRKTSSSKHDVSTYHALNIEHSQSDEMYINSSQRVGTTVTPESALIK---------RDEVFSVEPESEISTTADDYSSE------------------EEEFGADDSYSEHGAQYNQTHLEMMENELAGKQHEIEELSRELEEMRATYGTDGLQQLQEFEAAIKQRDGIITQLTAN-LQQARREKDETMRE--FLELTEQSQKLQIQFQHLQASETLRNSTHSSTAADLLQAKQQILT-HQQQLEEQDHLLEDYQKKKEDFKMQISFLQEKIRAYEMEENRKHTNLNVSRIL----LSSAFSLFHIYIDRFISNILDNFELCNTVLRGF--LLSSENQEI---EQLRLELSQKQMNLKKCSEEIKQLMGTVEELQKKNHKDSQFETDILQRMEQEAQRKLEQLRAELDEMYGQQIVQMKQELIKQHMSQIDELKIQHKGEMENALRSYPSVTVNEDQIKLMNMAINELNIKLQDTNSQKEK----LRGELGVISGEKSVLQRQLKDLFEELSFSREQIQRARQTIAEQENKLSEAHKSL-STVEDLKAEIVSASEYRKELELKHEAEVTNYKIKLEM--LEREKNAVLDRMAESQEAELERLRTQLLFSHEEELSKLKEDLEIEHRIDIEKLKDNLGIHYKQQIDGLQNEMSQKIETMQFEKDSLITKQNQLILEISKLK---DLQQSLMNSKSEEMTLQINELQKEIEILRQEEKEKGTLEQEVQELQLKTELLEKQMKEKEDDLQEKFTQLETENSTLKDEKKALEDMLKMYTPVNQEE-LIFIDSIKSKSQDCKWQKEIEILTEENEDLKKQCIQLTEEIERQRNTFSFAEKNFEVNYQELQEDYACLLKVKTDLEDSKNKQEVEYKSRLKTLSEELHHLQRINPTILKMKSSVFD---DDKTYIGEPLETVEVVEKDTTELMEKLEVTKREKLELSERLSDLSEQLKQKHGEISF-LSEKVKSLKQEKEQVLLRCRELEIIINHKRTENINVCDDQLDSLKDGVMTITSKDSGGSISKINKDCGEESKIMEEYKIPFENTTVGKESKQEQLFLDHLPLVRN--------------GSLLGTTEPNTNDKLQWELSVLKSEQNDLRLQMEAQRICLSLVYSTHVDQVREYMENEKDKALCSLKEELISAQEEKIKELQEIHQQELQNIKAQETGGKAKPLQMLIGKLRKAVSEECSYFTQT-FCNVLGEHYTPALKCEVNVEERESSGVHTSENQE--LELQDYKYEVQDFQENMQTLLNKVTEEYNKLLV-----LQTRLSKIQGRQTEDVKLKFAEGNLPKEETEFLS-ASQMTSLQDIDIDVSHKSKLSTLQDSE--KIKQLEGQVQELENLISSLQQQLKETEVNYGAEIHCLQERLQAVSESTVQPSFSIDSMVIT---ESDVQKTIYPGSCVNQNIDGTIEFCNDFGVKEETNVVKLLEKQYQ--ERLEEEVAKVIVSMSIAFAQQTELSRISGEKENTTLSKQAHAFCQQER--LNE-VKLSQG----QTFEAMEMKFKEEFKPLSKELGED-GTEVLLSNSDNLDDILESKDRELTISEEMFSKDKTFLVREPIH-DEILVSSMDASRQLMLNEEQLEDMRQELVRQYQEHQQATELLRQAHMRQMERQREDQEQLQEEIKRLNRQLAQRSSIDNENLVSERERVLLEELEALKQLSLDGREKLCCELRNSSTQTQ-------NGNENQEEIEEETFKEKELDRKPEDVLPDI-LTSERFALQKANNRLLKILLEVVKTTAAVEETIGRHVLGILDRSSKVPSS-ASLIWRSEAETPVKSCIHEEH-------TRVTDESIPSYSGSDKPRNDS-MWSKVTEEGTDLSERLVRSGFAGTEIDPENEELMLSISSRLQAAVEKLLEAISETS----------------------SQLEHAKVTQTELMRESFRQKQEVTESLKCQEELRERLHEESRAREQLAVELSKAEGVIDGYADEKTLFERQIQEKTDIIDRLEQELLCAGNRLQELEAEQQQIQEERELLSRQKE---AMKAEAGPVEQQLL-------------QETEKLMKEKL---EVQCQAEKVRDDLQKQVKALEIDVEEQVSRFIELEQEKNAELMDLRQQNQALEKQLE--KMRKFLDEQAIDR--EHERDVFQQEIQKLEQQLKVVPRFQPINEHQ-------TREVEQLTNHLKEKTDKCSELLLSKEQLQRDIQERNEEIEKLEFRVRELEQA--LLVSADTFQKVEDQKQCGAVEAKAELSLEVQLQAERDAIDRKEKEITNLEEQLEQFREELENKNEEVQ-QLHMQLEIQKKE-------------STTRLQELEQENKLFKDEMEKLGYAIKESDAIPT---QDQHVLFGKFAQIMQEKEIEIDRLNEQIAKLQYQLKI-----------------------------------TTDNKVIEEKNELIRDLETQIECLMSDQERVKKNREE--EIEQLNEVIEKLQQELANIEQKTSVDANSFPEEADSLKHQLDMVIAEKLALEQQVETTNEEMAFTKNVLEETNLKMNQLTQELCSLKRERENMEKIRSVPEKSVNMAMDDLSKIHPELEVVLAENALKPLENQTYFTSFEENSKGSISSLETKVLQL-EGTVSEKDIELTQCYKQIKDMQEQGQSETEMLKRKIVNLQNILEEKVAAALVSQVQLEAVKE----YAKFCQD--E--QAVSSELERTNV-QNLNQVTDNEMESNVLALTLRISELESQVVEMQTSLILEKERVEIVEKNALEKEKKLLELQRLLE-EEKKQGNKE------------RKRSPE-----------------------------------------------------VKETNMASLQKDLSQVRNQLTEAKEKLSYFLEKEDKTKVQENRIVC-----VSEPLPTNMGASLASQTEGTLKVNSS-SQPPQVLVRNAGTQIDLRSE--CSS---EEVTEIISQFTEKIEQMQELHAAEILDMESRHISETETLKREHYVAVQLLMEECGTLKAVIQCLRSKEGSSVPELTHSDAYQTREICSSDSGSDWGQGIYLTQSQGFDTASEERGDEG-ESSTDSFPKKIKGLLRAVHNEGMQVLSLTESPYSDGEDH--------SVQQVSESWLEERRAYLSTISSLKDLITKMQVQRDAEVYEGSQSHETFPDWRGELLLALQQVFLKERSVLLAAFQTELTALGTKDAVGLLNCLEQKIQEQSIEYQAAMECLQKADRRSLLSEIQALHAQMNG----RKMTLKREQENDQPNQELLEYTMQQKQSQMLEMQVELSSVKDRAAELQEQLSSEKMVVAELKSELAQTKLELETTLKAQHKHLKELEAFRLEVKDKTDEVHLLNDTLASEQ-----KKSRELQWALEKEKAKLGRNEERDKEELEDLKFSLEGQKQRNIQLNLLLEQQKQLLNESQEKIESQRVLHDAQLSEERGRNLELQVLLE---------------SEKVRIQEMSSTLDRERE----LHAQLQSSDDSGQPRPSLPSEDLLKELQKQLE-----EKHSRIVELLNETEKYKLDSLQTRQQMEKDRQVHRKTLQTEQEANTEGQKKMHELQSKVEDLQRQLEEKRQQVYKLDLEGKR-------------------LQGIMQEFQKQELEREEKRESRRILYQNLNE-PTTWSLTSD---RTRNWVLQQKIEG-ETKESSYLKLIEMNE-GGTGCNHELEVIRQKLQCVASKLQHLAQKASNRLQF-ETADDEDFIWVQENIDEIILQLQKLTG---QPGEEPGL-VSPGTSCGSLTERLLRQNAELTGHISQLTEEKNDLRNTVMKLEEQIRRYRQIGAG--RDYSSRFSFSGGASIEAIIASE-KEIWNREKLTLQKSLKRAEA-------------------EVYKLKAELRNETLLHNLSPDS---------EHAAIKRIYGKY-L-RAESFRKALIY-QKKYLLLLL-GGFQECEDATLALLARMG--------GQPAFTD-LEVITNRPRGF---------------------------------------------------TRFRSAVRVSMAI--SRMKFLVRRWQRVTS---------------------SGSININRDGFGLNPGAE-KTDPFYHSSGGLELYGEPRHTTYRSR-SELDYPRSPLP-FQNRYPGPPADLNPSSLACSQLQN-------------YDPDRALTDYITRLEALQRRLG-TVQSESS---TQFHAGMRR-------------------------------------------------------------------------------------------------------------------  
---------------------------------------------------------------------------------------------------------------------------------------------------------------------------------------------------------------------------------------------------------------------------------------------------------------------------------------------------------------------------------------------------------------------------------------------------------------------------------------------------------------------------------------------------------------------------------------------------------------------------------------------------------------------------------------------------------------------------------------------------------------------------------------------------------------------------------------------------------------------------------------------------QTLHTLELEALRLSLSNMHTAQLELTQANLRKEKETALVELREMLNDKRA------------------------QELALLQSRLQFELEHVREQHRKEKEEMALKHQQERADLGKKLELQVETQARTLETLRGDWASERDSSLRRLQEELATKHQAEMESLRGALRAQLAEQQAELD-------------KMGHDKSQAEATLRS-LQAQHQLALQDLRQELQLQHDQYLEDLNLKC-----REKQTELDTLQASYEELKVQSHREIQQLWAQLDSTRASRHELNELKEQLVARTSHVEELERLKHGFEQRQRQWRNEHETELEQLRVYFEQKL--------------------------RDAEESYREDLHLLQQRLREVREDSFLDSEDVSLSVGFVEEEAEMERKAHLEQLILQLEQHKDGIAHLRLQLEGKHREESATLEASLRARHEEEHSRRDWMGVEAGCLGLGILSQQNHTPDPVWL-HLQCAQLAARQVEAEVAERVSVLEDDHLARVAQLQSEEQLISQLREQVDLARRENMELQEKVQQEVRLR-----EEAEKMRCRLVKDHQTEIERMKEEIRQMIQKDQGREDAWR-------AQSEESLAALALELNGKAEQERQALRQALEAHVTELEQLRDRQAADIARLETSLGEARAQEEATGR--------------------------------LREECALELQANRARFLEEQREMMQKATEKQDVLLRELQDKHASELQIQKTELQAQHQAQLEARETELQAQHQAQLE--------ARETELQAQHQAQLEAR---ETELQAQHASETSGLEAKHLSNLDSLESCYLSEIQTIQEEHNRELEQLQARLEEQLREKDTQYEELLAQEKEKHQQELQQAQESLKIEMT-TKHIEKLK-ALTAQLQDAHQEDLETEKRAALREAEELLALEREKAQALCQRE-EALTLQLQEQSGLILQLKEKVLSLN---SEVSES-------HEELKKLQERRHREHEEGTNLLSLLRSDMDHTQNERKTLQETNQWLLSLFGDTLKATIAMRSQISKRIGICLD-EDSLSKESRPMGTPGQLAQGACELQCCTMPEC-----DETLLPECDETLLPGNDIS---------------CHVRESFFLSPEVTVECEQPIRRIYQSLGAAVDNLLEMTLDSN----------------------KQLEETRERHARFEKEFNRKNEETAQVARQHQELMDCLNQESAAKNQLAVELHKAEGLAEGYRAEKAALEEALAQKEESEHRLAVELENLKAQFQALTREQAKLKEEQNLLLRQKE---MLIVEAEEKAAALR-------------KEVEHLVREQA---ETRQQSEKDCASLRSHLRALELEVEEQLPQQQEL-VRQAAEIQDLRQQIVSLDKHLR--NQRQFMDEQAIER--EHERDEFQQEIQKLEEQLKQQPPKSQPWG------EPWDSEVESLQRQLREKIDELNELVIKKELAERQLLAQKDEIKNLE------------DANAEIRKKVSWLEEELEKQKKIGKELRQDKEALQEQQMNNLIQISTLQSKLDEAKHLQPVQGSPSEEDLREQLRAEQTA-------------LQLKAREVQNLEDELGELKKNLTSKSEEIVQLNLELALQNRQISGSIQELSAENADL--------------------------------------------------------KVMGDTSAEMEELKAIIDNLCENHERFRANKAE--EIERLHEVIEKLQKELS--VLG------PALHELSDSQGDSDGSQAENLQRELAQGLSSVQAREERLPLPPDELGQRLRDESQFRLRVAALEQELQDIRDASAQQL-------TLKEAEAR------KLAALARRWKTAARQKEA-------EVEAL-NEERGVLLAELETLVMACSRLEKELERKRPAGPHEPPELQMLRAQCVGLNLKLQALSQRLVT----YQLELDQ--HQACGPQCRVQALGA----SLMEPGRSAGQRGRFWATDHWALMLPSGPGPQLPLWESPTVSSKPSSSKESGLSVLTVCQEQLESELLLMKAK----------MQMGAEDRSKLIQQKPVDVCQLQKGDLITQVRHIQEKLHHLIRSISCRDSHVQSPPPQGPVSTVLQHSWTLHDRTESPDPPGQGGPTPTLDK------TPDAWLSSPSDADPSTPDFSCSEDCVAAQRAPPCTETPGSPPPGGSARG--SPPSARSAE--EASQDADPLDAVQHFRDTILDMDSSSLGSPELVRKDSTLDRLPSVHLTP-LSQAADLQSPDSAW--RSLGLQDSSGCLAFPALPGLEASPQPSLTEKARPRAGRATEATVSEKDVEDFILTPSN-SQEHLESTAPGERGESDGHDNSESGRSRLACLGGGGRDGPGV---RAPGGTPPSGPPGPRPLPPVAASSLCPPWRGPALLKMVCDESQQILVL-SKCEAPASDLGSALPAPTPDCFQKERQGLLDAEPSDTGFDWGGEFLQAVQEVLEKERNMLKIELQSKLCSLDSGDERSLLEKLEDVV--KEQVGGAPSVGGAALPRDIEYQIFLKGQDYSPPLLSFVGNSSFQQRLNSFLLSSHPKSISHFHHHDEHHTKLIQTPQMQFETEQELEDLKKQKQKERRGIGGLFTASPSSF-----SDTRYKTFFIQLSYWSVGPSPFHLEAPAVPLW-----GPALANGLGPPPRPSLSTLEPELSPRKPAEWGAPHPKSSGSRALLPPRVSVE---------------RPTAFLFGQWSASGPSSAPSPWLPRQVVLRLLESLASHPPSAWARGPLGSLLLP-----FRGSLLSRQSPYILGLRVIFEPLGGFSSKTCLCRYFFPFENQQDQNIV--AIVASLSLFLCQHSLPCSSGSNPFSSPASSDLGAVRLEA------RPASPPCSFHRPPLSCPWAFARAARVPLESLPQAPGSRLPRQGLQRG---WLGRVASGRGPSGCVTRRLLVSRSEGWQR-ELELQRQRDEHKIQQLQQTISELQNDKGFVPSRGWQ-ECGVGVPLPPNTEKLKEHQEQLQSVRQQLLHAVALLTS-FMNQTVNRTINDWASSNEKAVTSLLLTLEELKSELMMTLPQKK--NTQAQISLIDVLLKENGSLTKALSTMTQEKVELC-KTVSKLERTLKHHLQKGCGHIVR----------AGPGGLVSRRAAVSGAPDSRLPSQYKYVDRIGTLQKVDKTTMRRLYLHF-L-RAESFPEGPSFIRKRYLLLLI-GGFQDSEQETLSMIAHLG--------VFPSKLD---KRAFSSRPF---------------------------------------------------T-FRTAVRVVIAI--LRLRFLVKKWQELDRRGI------------------TAAGRLPR--PPSKLR---APHVP-WQTR--PMGRMFPLTSHRASPVVLGNAMEGVSRPRGWGGRFSPHPLAPPLERPPRRNSQ------------DPEHSLTEYIHHLEIIQQRLGGVQPELSSKKPGHLKTKK---------------------------------------------------------------------------------------------------------------------  
-------------------------------------------------------------------------------------------------------------------------KLAQFRQKKA-----QSDGQTPSKKQKRKKKSSARKYEDSSYTSN-IDQSQGDQLYMNSSQR-GASMTPEFSIMRTLQSGEIIKHDQSYTIEPESEISTTADDYSSEVNGCS-LVRTDIPTHLLMEEEFGVDESYSEQGAQYSSTRLEMMENELAGKQQEIEELSRELEEMRAAFGTEGLQQLQEFEAAIKQRDGIITQLTAN-LQQARKEKDETMRE--FLELTEQSQKLQIQFQHLQASETLRNSSHSNTAADLLQAKQQILT-HQQQLEEQDHLLEDYQKKKEEFKKQINLMQEKIRTYEMGKVIQI-GLIKEIPCELN------------------FFSKRQKLESGMEVKLCEDRDKRQNVR---KTNQHTSPELEIWNREGRTRRSSIFLEPSLLWSGRLERLNLECGLSLVDGEEPLKMFKQHRRRRNRNKHQQIVQMKQELIKQHVLQIDELNAQHKGELENTLKSCPNITVNEDQIQLMNMAINELNLKLQDANSQKEK----IKEQLEVISGEKSILQRQLEDLFEELSFSRDQIQRARQTIVEQEGKLNEAHRLL-SSVEDLKAQIVSASEFRKELELKHEAEVTNYKIKLEM--LEKEKNAVLDRMAESQEAELERLRTQLLFSHEEELSKLREDLEIEHQINIEKFKDKLGIHYKQQIEELHSEMSQKIETMQFEKDNLITKQNQLLLEISKLK---DLQQSLVNSQSEEMTLQISELQKEIEVLRQEEKEKGTLEQEVQELQLKTEFLEKQIKEKEDDLQEKFTQLEAENNILKNEKKALEDMLKIYVPISKEESLIIIDSAKSKSEDYDWQKKMDILLEENEKLTKQCDQLNEEIEKQRNTYSFAEKNFEVNYQELREEYACLLKVKADLEDSKSKQEAEYQMQVQALTDELCNLQRIKPILVKSKSSDSESCKDDKPFTTEISEVVEVVEKDTTELMEKLEVTQREKLELSERLSDLSEQLKQKHGEIDH-LSEEVKSLKQEKEQVLKKCKELEIIIGHSRAEHMDELKLKLCDFKDGIQTEESKDSERFVFKKNKDFSEGAKITEENEISSEDAAVKKECKQGLVKQEKHTLLDHLDSVTDQLAN--ESTSSSLAIAQSENYQLQQELRILKSEQADLRLQMEAQRFCLSLVHSAHVKQVQACMEEETASALNSLREDLVSAHEEEMKELQKMHHLELQNRKGQKIDEEVMSLEILMEKLHRAVTEECSCFNQS-LCNILGAYNATPFKYEIKVEEKEVSDIHSPEVQEDNSELQNYTFEASDLQSNMQSLVNKIAEEYNKLLV-----LQSQL-TINSWAPQGLKTRKGKLLWPKGSTLETDSIHSIDSKTEGKGLKK-KNNLPPLEDIEKLKNQLEAQDFQGQGHFPCNFQPQLKEIEIKNPSEILHLQEALQPVQESVLQPSVISESLKNM---DQVVGKTKYIKSCLQKTEDGSAKFTDETGRKEEMDIQ-FLEKQYQ--ERLEEEVAKVIVSMSIAYAQQSEIARISRQKEDVASVTQINAHQ-QKELFLSD-RKCSRNEISIQTFDQEEMKF-KELKPLSKELREEEFKEILLSNSSNLESTFELKDQELDFSREVFSSNREFIFTEHTR-DEMLASNTEASRQLMMSEEQLEDLRQELVRQYQEHQQATELLKQAHMHQMERQREDHEQLQEEIRRLHQELAQSASIDNENLVTERERVLLEELEALKQRSLAGREKLCCELRSSYTQTQ-------DENESQGEIEEPIIQEELEEKPP--AVSSDILSKERYALQKANNRLLKILLEVVKTIAAVEETIGRHVLGILDRSNKGQSS-TNIIWKSESEGTVKSCIHEED-------TE--EESMGSYCGSGMPSHATSTWSEITDEGMELCQRLLRCSLAGTEIDPENEELVLNVSSRLQAAVEKLLEAISETS----------------------SQLEHAKVTQTELMRESFRQKQEATEFLKCQEELLERLFVELKAEQLAVELNKGDASIIDGYADEKALFERQIQEKTDIIDHLQQELLCVGNRLQELEAERQQIQEERELLSRQKD---AMKAEAGPVEQQLL-------------QETEKLMKEKL---EVQCQAEKVCDDLQKQVKTLEMDVEEQVSRFMELEQEKNAELMDLRQQNQALEKQLE--KMRKFLDEQAIDR--EHERDVFQQEILKLEQQLKATPKFQPISEHQ-------TREVEQLTNHLKEKTDKCSELLLSKEQLQRDVQERNEEIEKLESRIRELEQA--LLISADNLQKVENRKHFGAVEAK-ELPLEVQLQAEREALDRKEKEITNLEEQLEQFREELENKNEEVQ-QLHMQLEIQKKE-------------ATTRLEELEQENRLFKNEMERLGFAVQEFEDVST---KDHHQLFGKFTQMIEEKELEIGRLNGQITRLQQQLEI-----------------------------------TTDNKIIEEKNQLIQYLESQLEFLKSDQERIKKNSEE--EIEQLSDVIEKLQQELSSIEQKAPEDFSSLPEEPNDLKQQLEAVRADKLALEQEVGRTREEAALAHAGLRESSARMNHLQEELDALREEQHGWSQTQSPRGSVQGAGQSQPKQLGIEDPTSLQPPAGKSRGFGGELELALLHPG-------------------------EKGAEPRWMEDSKGQADKETLQREVSGLRAALEEKVAAFVVSQAQLRAIQE----HVSRWQQ--RAVSELSGGAQEAQG-DSRPLCQKEPKEAAEERKEKKLLGLEETPEEVEKKEKKEEDAGLAQVSSDSLIFFPKHSALTLGLWTLSGWLLAT------------KAGDPSLI--------------------------------------FPKKAEKLQEMPVQVREANMALQEELNQIKHQLMEAEKKLEYYLKKEGELRVSESQKASCELGQFLAERDSPLTGQNSSSQMDRILQVSSGHQTPHIVTKHTGIQSDPPSS--CPS---EDVTEVRRQLTEKMAHMQELHAAEILDMEARHIHETDSLKREQAVAVRLLTEECGALKAAIHCLRAREVPAGVGRGQLCAY---------SGSDWSQGVYLTQSQGFDSASDGPGEEG-ESSTDSFPKRIKGLLRAVHNEGIQVLSLTEPPYSEGDAN--------FAHQASEFWLEERKTYLNAISSLKDLITKMQVQRETELYESSQPQENPPDWRGEFLCALQQVFLMERNILLAMFKTDVTALGTRDVLGLLNQVEQRLQEQGLEYRAAIEYIQSADRRSLLTEIQTLQAQVKS----KIMTLKREQESDLQNQELLEYNMQQKQAQILEMQVELSTLKERAAELQDQLNAERVRAAELQDGWTQAKLELETTLKAQHKHLKELEAFRMEVKEKTDKVHLITETCHLPYSFYFFNLRNIFYWHKGSGFHLVTKINIEIKLHDRDLKYSLEGQKQRNVQLNQLLEQQKQLLTDLQQKIVAQGALYDAQISEERSQKSELQGLLE---------------SEKVRVLEIQETLERERE----LHAQLQAHDDHEQDKASTSSEDVLKELQKQLY-----EKHNRIVELVSAIEKYKLDFLQMRQQTEKDRQMHRKTLLTEQQANTEGLKKIHELQSKLEDLQWQLEEKRQQIYKLELERKR-------------------SQEIIQELQKQEQEKEEKIETERVSYQNLNE-PTTWDSSSD---RTRNWVLQQKIEGAEEKESSYPPLIEMNGDGTIEDIREPEAIRQKLQIISSKLQQLVHKASSRLQF-EAADNEDLIWVQKNIDHILLQLQKFTGL--SQQGEATL-VSPGLSCNSLTERLLRQNAELTGHIRQLTEEKNDLRNVVMKLEEEIRWFRKTGPC--GDYSNRHSFSRGTNIEAVIASE-KEIWNREKLALQKSLRQAEA-------------------EVCRLKAELRNEALLQDLNPNS---------ESTALKRIYGKY-L-RAESFRKALIY-QKNYLLLLL-GGFQECEDATLALIARMG--------GQPSCTD-LTVITSQSKGF---------------------------------------------------TRFRSAVRVSIAI--SRMKFLVRRWHRVTG---------------------SCSININRDGFGQNPGTD-KTDPFYHSPGGLELCGEQRHSSYRSR-SDLDCPRSPLH-FQHRYPGTPADLNPGSLACSQLQN-------------YDPDRALTDYIHRLEALQRRLG-NVQSGSTPSSTQFHAGMRR-------------------------------------------------------------------------------------------------------------------  
-------------------------------------------------------------------------------------------------------------------------------------------------------------------------------------------------------------------------------------------------------------------------------------------------------------------------------------------------------------------------------------------------------------------------------------------------------------------------------------------------------------------------------------------------------------------------------------------------------------------------------------------------------------------------------------------------------------------------------------------------------------------------------------------------------------------------------------------------------------------------------------------------------------------------------------------------------------------------------------------------------------------------------------------------------------------------------------------------------------------------------------------------------------------------------------------------------------------------------------------------------------------------------------------------------------------------------------------------------------------------------------------------------------------------------------------------------------------------------------------------------------------------------------------------------------------------------------------------------------------------------------------------------------------------------------------------------------------------------------------------------------------------------------------------------------------------------------------------------------------------------------------------------------------------------------------------------------MLRSDIELSQNERKTLQETNQRLLNLFGETIQAVIAMKSHIHKKIGHCLEGIDSFGEENYPLLGEGESSQDSCH---ETLPEC-----GKTLLEH--EQISSVAEIS---------------SHLCESFFMNPEGTLECEQPVKKIYQSLGAAVDNLLEMILDST----------------------KQLEETREIHSQFEKEFNRKNEEIAQVVKQHKELVECLNEESTAKNQLTVELHKAEGIIEGYRAEKAALEEMLSQKEKSEHHLVVELENLKEQFQLLTYERTKLGEERNLLLSQKE---ALAAEAEEREVGLL-------------KKVECLAKEQT---ETKKQSEKDRSTLLSQMKVLEVELEDQLSQNQEL-AKKTAEIMDLKQQIVSLDKHLR--NQRQFMDEQAIER--EHERDEFQHEIKKLEEQLKYTT-KFQSVG------EFRPNEVSLL------------------------------------------------------------------------------------------------------------------------------------------------------------------------------------------------------------------------------------------------------------------------------------------------------------------------------------------------------------------------------------------------------------------------------------------------------------------------------------------------------------------------------------------------------------------------------------------------------------------------------------------------------------------------------------------------------------------------------------------------------------------------------------------------------------------------------------------------------------------------------------------------------------------------------------------------------------------------------------------------------------------------------------------------------------------------------------------------------------------------------------------------------------------------------------------------------------------------------------------------------------------------------------------------------------------------------------------------------------------------------------------------------------------------------------------------------------------------------------------------------------------------------------------------------------------------------------------------------------------------------------------------------------------------------------------------------------------------------------------------------------------------------------------------------------------------------------------------------------------------------------------------------------------------------------------------------------------------------------------------------------------------------------------------------------------------------------------------------------------------------------------------------------------------------------------------------------------------------------------------------------------------------------------------  
---------------------------------------------------------------------------------------------------------RERRTRRRRLKASEGSKLAQFRQRKA-----RSDGQKTKKKKQQPPPGS------------------RYEVSSAKAVV-GPDGARDDEGP-----------DQAYAVEPESEISTTADGFSSEEE----------------EGEFGVGEVFSERGAQDGQARLEAAETELAGRLQEIEELSRELEEARAAYGPEGLQQLQEFEAAITQRDGIISQLTAH-LQQARREKDESMRE--FLELTEQSQKLQLQFQHLQASETLRNSTHSSTAADLLQARQQAAT-QQQQLEEQ------------------------------------------------------------------------------------------------------------------------------------------------------------------------------------------------------------------------------------------------------------------------------------------------------------------------------------------------------------------------------------------------------------------------------------------------------------------------------------------------------------------------------------------------------------------------------------------------------------------------------------------------------------------------------------------------------------------------------------------------------------------------------------------------------------------------------------------------------------------------------------------------------------------------------------------------------------------------------------------------------------------------------------------------------------------------------------------------------------------------------------------------------------------------------------------------------------------------------------------------------------------------------------------------------------------------------------------------------------------------------------------------------------------------------------------------------------------------------------------------------------------------------------------------------------------------------------------------------------------------------------------------------------------------------------------------------------------------------------------------------------------------------------------------------------------------------------------------------------------------------------------------------------------------------------------------------------------------------------------------------------------------------------------------------------------------------------------------------------------------------------------------------------------------------------------------------------------------------------------------------------------------------------------------------------------------------------------------------------------------------------------------------------------------------------------------------------------------------------------------------------------------------------------------------------------------------------------------------------------------------------------------------------------------------------------------------------------------------------------------------------------------------------------------------------------------------------------------------------------------------------------------------------------------------------------------------------------------------------------------------------------------------------------------------------------------------------------------------------------------------------------------------------------------------------------------------------------------------------------------------------------------------------------------------------------------------------------------------------------------------------------------------------------------------------------------------------------------------------------------------------------------------------------------------------------------------------------------------------------------------------------------------------------------------------------------------------------------------------------------------------------------------------------------------------------------------------------------------------------------------------------------------------------------------------------------------------------------------------------------------------------------------------------------------------------------------------------------------------------------------------------------------------------------------------------------------------------------------------------------------------------------------------------------------------------------------------  
-------------------------------------------------------------------------------------------------------------------------------------------------------------------------------------------------------------------------------------------------------------------------------------------------------------------------------------------------------------------------------------------------------------------------------------------------------------------------------------------------------------------------------------------------------------------------------------------------------------------------------------------------------------------------------------------------------------------------------------------------------------------------------------------------------------------------------------------------------------------------------------------------------------------------------------------------------------------------------------------------------------------------------------------------------------------------------------------------------------------------------------------------------------------------------------------------------------------------------------------------------------------------------------------------------------------------------------------------------------------------------------------------------------------------------------------------------------------------------------------------------------------------------------------------------------------------------------------------------------------------------------------------------------------------------------------------------------------------------------------FQEELKALSQGLSEDESRKILVLGSERGDGELELEARELACAGRVFPRDQDFVLKDTMR-HDVP----EASLQLRLHEEKLEDLRQELVRQCEEHQQVTERLRQAHMRQMERQREDQEQLQEEIHRLNRQLAQSSSVDTENLVSERERVLLEELESLKQRALAGREQLCFQVHSRSTQTQ--------GNHFSQEAQEPIPGGEERERNAEDVAPDG-LSAERHALRQANRRLLKGLLEVAKTTVAAEETIGRHVLGLLNRSGGGPPA-EGPTLHGASERQAVSLRNEWC-------VLSGEPAAP-FLEREGPGRAPGPWTEAPGGGEDLCQLVLRSGSAGPDVGPESEELALNVSSRLQAAVGKLLDAINTTS----------------------DQLEHARVAQTELLRESSRQTQEAAELLRCQEE------------------------------------------------------------------------------------------------------------------------------------------------------------------------------------------------------------------------------------------------------------------------------------------------------------------------------------------------------------------------------------------------------------------------------------------------------------------------------------------------------------------------------------------------------------------------------------------------------------------------------------------------------------------------------------------------------------------------------------------------------------------------------------------------------------------------------------------------------------------------------------------------------------------------------------------------------------------------------------------------------------------------------------------------------------------------------------------------------------------------------------------------------------------------------------------------------------------------------------------------------------------------------------------------------------------------------------------------------------------------------------------------------------------------------------------------------------------------------------------------------------------------------------------------------------------------------------------------------------------------------------------------------------------------------------------------------------------------------------------------------------------------------------------------------------------------------------------------------------------------------------------------------------------------------------------------------------------------------------------------------------------------------------------------------------------------------------------------------------------------------------------------------------------------------------------------------------------------------------------------------------------------------------------------------------------------------------------------------------------------------------------------------------------------------------------------------------------------------------------------------------------------------------------------------  
-----------------------------------------------------------------------------------------------------------------------------------------------------------------------------------------------------------------------------------------------------------------------------------------------------------------------------------------------------------------------------------------------------------------------------------------------------------------------------------------------------------------------------------------------------------------------------------------------------------------------------------------------------------------------------------------------------------------------------------------------------------------------------------------------------------------------------------------------------------------------------------------------------------------------------------------------------------------------------------------------------------------------------------------------------------------------------------------------------------------------------------------------------------------------------------------------------------------------------------------------------------------------------------------------------------------------------------------------------------------------------------------------------------------------------------------------------------------------------------------------------------------------------------------------------------------------------------------------------------------------------------------------------------------------------------------------------------------------------------------------------------------------------------------------------------------------------------------------------------------------------------------------------------------------------------------------------------------------------------------------------------------------------------------------------------------------------------------------------------------------------------------------------------------------------------------------------------------------------------------------------------------------------------------------------------------------------------------------------------------------------------------------------------------------------------------------------------------------------------------------------------------------------------------------------------------------------------------------------------------------------------------------------------------------------------------------------------------------------------------------------------------------------------------------------------------------------------------------------------------------------------------------------------------------------------------------------------------------------------------------------------------------------------------------------------------------------------------------------------------------------------------------------------------------------------------------------------------------------------------------------------------------------------------------------------------------------------------------------------------------------------------------------------------------------------------------------------------------------------------------------------------------------------------------------------------------------------------------------------------------------------------------------------------------------------------------------------------------------------------------------------------------------MQREELKAKTLEELKSSLD---------------IQHTQNNQLSIALKHEQMAKENLKKELQIEYSRCEALLFQERNKLLELQKTLET-----EK-NHSAELSEALNHERVLTEQLSKRANETCALKEAQSQQTLLRKLKEEKSRTMELQAALEKAQQQAIRSKKKLEAEIQMHREEIKKEK----------------ERQLEVQHQRDEHK----IKELQQMLVKLE-EKE--------RNFASTKNCPEAAAKTSCN---------------------------------------PDSNLG-LAFLP-----DSERFESQQ--LEKIRQQLLYVAVLLTN-FINQTIDRTINDWSTSNDKAVFSLLHTLEELKSELCISSTPQK--TAQVQISLVDSLLKENGSLTKTLTTLTQEKVQLT-QTVHKLEKTLKHHLQKGCGQMNKYVDRIDGGPLQKPEKNVWKRQKISLKHAEPGPVKSVLRNEPSPVVRTSNVKVEKLYLHF-L-RAESFRKALIY-QKRYLLLLI-GGFQESEQETLSMISRLG--------VFPSKAD---HKAIPPRPF---------------------------------------------------TKFRTAVRVVIAI--LRLRFLVKKWQAVDRKGV------------------LTGGTELDTVPGEILE---VTKQPLPDARDSPPTRDVSL-CHTANG-IQVTKPQPAPINRFWE-RSISSP-SPVSEKS-VACSQ------------DPERSLTEYIHHLEIIQQRLGGAQSEFASKNPAIKRPKK---------------------------------------------------------------------------------------------------------------------  
-------------------------------------------------------------------------------------------------------------------------------------------------------------------------------------------------------------------------------------------------------------------------------------------------------------------------------------------------------------------------------------------------------------------------------------------------------------------------------------------------------------------------------------------------------------------------------------------------------------------------------------------------------------------------------------------------------------------------------------------------------------------------------------------------------------------------------------------------------------------------------------------------------------------------------------------------------------------------------------------------------------------------------------------------------------------------------------------------------------------------------------------------------------------------------------------------------------------------------------------------------------------------------------------------------------------------------------------------------------------------------------------------------------------------------------------------------------------------------------------------------------------------------------------------------------------------------------------------------------------------------------------------------------------------------------------------------------------------------------------------------------------------------------------------------------------------------------------------------------------------------------------------------------------------------------------------------------------------------------------------------------------------------------------------------------------------------------------------------------------------------------------------------------------------------------------------------------------------------------------------------------------------------------------------------------------------------------------------------------------------------------------------------------------------------------------------------------------------------------------------------------------------------------------------------------------------------------------------------------------------------------------------------------------------------------------------------------------------------------------------------------------------------------------------------------------------------------------------------------------------------------------------------------------------------------------------------------------------------------------------------------------------------------------------------------------------------------------------------------------------------------------------------------------------------------------------------------------------------------------------------------------------------------------------------------------------------------------------------------------------------------------------------------------------------------------------------------------------------------------------------------------------------------------------------------------------------------------------------------------------------------APWERVDVAELKSQLAQVRLELAAALQARHRQAQELEALRAELRGKKDEVDAANDSLAAEQ-----RKGRELRWALEKAEAQAARSQDRGREELEDLRLSLEDQAQRNVELSRLVAQQRQQLSDLREKTESRETLHAAQLSAERGWSAELQRLLD---------------SEKAREPEGSAVAEQ------------GGTHAPPPARDGEAAEEAAAELRRQVQ-----EQQRRLAELVREAEASRLESLRAGQQLERERQAQGAALRAAQEAQAQGRQRLQASQARLEELQAQLDGRTQQVRSLERECRR-------------------SQGIVQDLRNQLRARE------------------------------------------------------------------------------------------------------------------------------------------------------------------------------------------------------------------------------------------------------------------------------------------------------------------------------------------------------------------------------------------------------------------------------------------------------------------------------------------------------------------------------------------------------------------------------------------------------------------------------------------------------------------------------------------------------------------------------------------------  
------------------------------------------------------------------------------------------------------------------------------------------------------------------------------------------------------------------------------------------------------------------------------------------------------------------------------------------------------------------------------------------------------------------------------------------------------------------------------------------------------------------------------------------------------------------------------------------------------------------------------------------------------------------------------------------------------------------------------------------------------------------------------------------------------------------------------------------------------------------------------------------------------------------------------------------------------------------------------------------------------------------------------------------------------------------------------------------------------------------------------------------------------------------------------------------------------------------------------------------------------------------------------------------------------------------------------------------------------------------------------------------------------------------------------------------------------------------------------------------------------------------------------------------------------------------------------------------------------------------------------------------------------------------------------------------------------------------------------------------------------------------------------------------------------------------------------------------------------------------------------------------------------------------------------------------------------------------------------------------------------------------------------------------------------------------------------------------------------------------------------------------------------------------------------------------------------------------------------------------LEQELLRAGNRLQELEAERQQVQEEKALLSRQKE---ALSAEAGPAEQRLLGAAVHAALQAEFLQETEKLAKEKL---DVQRQAEKERDDLQKQVRTLEVDVEEQVSRFLELEQEKNAELADLRQQNQALEKQLE--KTRKFLDRNSVGRHVPAHKKLTDHRGRQTLKQIKDRGKKQKCSAHS-------KHSVEQLTEQLKEKTDRCSEGLLAAEQLQRDLQERDDQLDRLGCRLRELEQA--LVLGPHGRPEAEEQQRAGAADGKGELALEAQLQVEREAVDRKEKEIANLEEQLEQFREELENKNEEVQ-QLHMQLEIQKKE-------------STTRLAELQLDNQLFKEELGRLGLAVQGSEGTVA---PDPHLIVGKFARILQEKELEIQELNEQMAKLQQQLET-----------------------------------AADNKVIEEKNELLRELEAQVERLKSDREWVKKRSQE--EVEQLNDVIEKLQQELAGLDQKEPVGGALPPGGADSLKRQLEAALA-------------------------------------------------------------------------------------------------------------------------------------------------------------------------------------------------------------------------------------------------------------------------------------------------------------------------------------------------------------------------------------------------------------------------------------------------------------------------------------------------------------------------------------------------------------------------------------------------------------------------------------------------------------------------------------------------------------------------------------------------------------------------------------------------------------------------------------------------------------------------------------------------------------------------------------------------------------------------------------------------------------------------------------------------------------------------------------------------------------------------------------------------------------------------------------------------------------------------------------------------------------------------------------------------------------------------------------------------------------------------------------------------------------------------------------------------------------------------------------------------------------------------------------------------------------------------------------------------------------------------------------------------------------------------------------------------------------------------------------------------------------------------------------------------------------------------------------------------------------------------------------------------------------------------------------------------  
--------------------------------------------------------------------------------------------------------------------------------------------------------------------------------------------------------------------------------------------------------------------------------------------------------------------------------------------------------------------------------------------------------------------------------------------------------------------------------------------------------------------------------------------------------------------------------------------------------------------------------------------------------------------------------------------------------------------------------------------------------------------------------------------------------------------------------------------------------------------------------------------------------------------------------------------------------------------------------------------------------------------------------------------------------------------------------------------------------------------------------------------------------------------------------------------------------------------------------------------------------------------------------------------------------------------------------------------------------------------------------------------------------------------------------------------------------------------------------------------------------------------------------------------------------------------------------------------------------------------------------------------------------------------------------------------------------------------------------------------------------------------------------------------------------------------------------------------------------------------------------------------------------------------------------------------------------------------------------------------------------------------------------------------------------------------------------------------------------------------------------------------------------------------------------------------------------------------------------------------------------------------------------------------------------------------------------------------------------------------------------------------------------------------------------------------------------------------------------------------------------------------------------------RKEKEVTNLEEQLEQFREELENKNEEVQ-QLHMQLEIQQKE-------------STTHLQELEQENKLFKDEMEILGLAIQKSEDAAI---KDHHLVAGRLAHIMQEKDQEIYHLHEQIAKLQLQLEV-----------------------------------TADNKVIEEQNEHIQELEAQVECLKSDQERVKKKKDE--EVEQLNDVIDKLQQELAIIGQTIPTDVTGFQEDADNPKHILEAVLAEKEALEKQVENINTEASQTKNELEETKLKMSQLKQEITMLKKEHARITEKCG-------KSKPEDRSNGKTEQMEGDLCQDIDLSDRSQLRTSDENARVTISKIENQLQQL-QACIRQKDLELCQCYNEIKDMKEQSKAEKETLKKRILELEKTLMEKVAAALVSQVQLNAVQE----QRRFMQEIQEASKSVDEACKNAQK-EDLSDRTENETESKLSHLTQRLSEMEDQLAMVNHSLELEKENLKVAQKEAAVKEERFLELQQFLEEIKEKHK---------------GEIQRYIKQE----------------------------------------------ELQTYQASLIHLQEDLCKVKDNLIQAEEKLASYLRKNKEVVKTESKKDAEISCDLSATESTLTGNSSSSQTDKTMEIS-------PVLVKNAEIQIDLQNG--CSS---EEIAEIVREFTEKIDQMQELHAAEIMDMETRHISESEALKREKFVAVQVLTEECNTLKEVIETLQAKERIPVSGLAHSPPHQARDGGSSDSSSDWSQGMYLAQTQGSDTISE-GIDEG-ETSTDLLPKKIKGLLRAVHHEGIQVLSLTEFPYSEKDLS--------AHKQEPESWLEERKAFLSTISSLKDLITKMQVYREAEWAD----ELCVSDWRGELLHAIQQLFVREQNVLLAAFQTELAEMGTRDAVMLMNQLEHRLQEQATNQRAAMDSLQNADRRSLLMEIQVLHAQMNS----KKSNPKREQEIDSKSQEMLEYNMQQKQLQILEMQVELRSMKDRAAELQEQLNSERMMGSELKNELAQAKLELEATLKAQHKHFKDLETIRTEVKEKAAELDILKDTMANEQ-----KKSRELQWALEKEKAKMERSEERGREELEDLKFSLEDQKQKNLELSKLLEQEKQLSSDLQQRIESQEALSAAQLSRERGRNSELQVLLE---------------SEKVRALEISSALEREKE----LCAQLQSAEDKGQAGTPNPSEELLKELQKQMD-----EKHDRIVDLVSEMEKYKLESVQVRQQMEKERQIHRQALQAEQDANIIVQKKLHELETKVEDLQWQLGEKKQEVHKLDNETKK-------------------LQEIIQELQKKEQEDEGRKEAKRTPSHNPN--ETTWDTPND---RTRNWVLQQKMEGAETNELTYRTLTG----DLSAATEILEKVRQKLQNASPKLKQLAWKAASRLQF-ETADDQDFVS-----------------------LKMVL-PPGTPSS-SLTERLLRQNAELTGFVSRLSEEKNNLRNAVMKLEEELRRYQHRQPS--ADYSSRHSADVGVNIDTLVASE-KEIWNREKLSLQKSLKQADA-------------------ELSKLRAELRSEAFLRELGSDT---------ENAVLRRIYGKY-L-RAESFRKALIY-QKKYLLLLL-GGFQECEEATLALIARMG--------GQPSYTD-LEIITHHSKGF---------------------------------------------------TRFRSAVRVSIAI--SRMKFLVRRWHRVTG---------------------SGILTINRDVFSQNTGNELRPDSF---PGGIDLYGEQRHS-SRSRKLNFNSPW--IL-LQ--LHGMHADLNPVSFTCPQLQN-------------YDPERALTDYIHRLEALQKRLG-SVQSGST-SYTQLHLGIRR-------------------------------------------------------------------------------------------------------------------  
----------------------------------------------------------------------------------------------------------------------------------------------------------------------------------------------------------------------------------------------------------------------------------------------------------------------------------------------------------------------------------------------------------------------------------------------------------------------------------------------------------------------------------------------------------------------------------------------------------------------------------------------------------------------------------------------------------------------------------------------------------------------------------------------------------------------------------------------------------------------------------------------------------------------------------------------------------------------------------------------------------------------------------------------------------------------------------------------------------------------------------------------------------------------------------------------------------------------------------------------------------------------------------------------------------------------------------------------------------------------------------------------------------------------------------------------------------------------------------------------------------------------------------------------------------------------------------------------------------------------------------------------------------------------------------------------------------------------------------------------------------------------------------------------------------------------------------------------------------------------------------------------------------------------------------------------------------------------------------------------------------------------------------------------------------------------------------------------------------------------------------------------------------------------------------------------------------------------------------------------------------------------------------------------------------------------------------------------------------------------------------------------------------------------------------------------------------------------------------------------------------------------------------------------------------------------------------------------------------------------------------------------------------------------------------------------------------------------------------------------------------------------------------------------------------------------------------------------------------------------------------------------------------------------------------------------------------------------------------------------------------------------------------------------------------------------------------------------------------------------------------------------------------------------------------------------------------------------------------------------------------------------------------------------------------------------------------------------------------------------------------------------------------------------------------------------------------------------------------------------------------------------------------------------------------------------------------------------------------------------------------------------------------------------------------------------------------------------------------------------------------------------------------------------------------------------------------------------------------------------------------------------------------------------------------------------------------------------------------------------------------------------------------------------------------------------------------------------------------------------------------------------------------------------------------------------------------------------------------MLKQAES-------------------QLAKAKAEIENKPVAEIS--------------NPKLQRLYRKY-I-RAESFRKALVY-QKKYLLLLL-GGFQDCEQATLSLIARMG--------IYPS-AD-LQLSGSRSRPF---------------------------------------------------TKFRCAVRAIIAI--SRLKFLVKKWNKVGR-KS------------------TQGESISHSI--------------------------------------------------------------------------------------------------------------------------------------------------------------------------------------------------------------------------------------------------  
-------------MAGHYIRPLPWSEGARYALTCINTARGLLTAYPVPKAKQAYSIKALAKLMSAYGTPQVFENEQGTHFTGATIQCWAEENNIXXXXXXLPYNPTGYVLGWMKKVEFTKIKLAQFRQRKA-----QTDGQSTSKKQKKKRKTASIKDEESVQDG--IDRSRGDETSTCSSRR-GAAATADFPVTRTLHSGEIIKHIQAYTNEPEIEISTAVEDYSSEVNGCSFVTRTAIPTDLIREEEFGVGEICSEHGMQHSLMQLEVMENELAGKQQEIEELNRELEEMRAAYGTEGLQQLQEFEAAIKKRDDIITQLTTN-LQQARKEKDETMRE--FLELTEQSQKLQIQFQHLQASEALRNTSHSCTAADLLQAKQQILS-HQQQLEDQECLLKNYQKKNEEFEVQITHLQGMIKTYELEKQRNE-EEDMNKRQEKEALVEELEAKLKEEEKKSFQLKEKLSDVSKLHEELKEQISLKNLEI---SNMRIELTTYKQKERQCSDEIKQLMGTVEELQKRCYKDSQSEADIVQRMELETQRRLAQLQAELDEMHGQQIVQMKQELIKQHAMEIEQRLAQQKVELEKTSSLCLSDNVNKDQMHLMNIKINELNVKLQDADNEREKIKQELSQQMEVISAEKSLQQTKIEDLLQELNFSREQVQRAKQTIMDMECRLNEAEKYQ-FVIEDLETQLASASEFRKELELKHEAEVTNYKIKLEM--LEREKDAVLDRMAESQEAELERLRTKLLFSHEEELSRLKEDLQKEHMVNMESLRDNLNMHHKQQLDGVKKEMSQKIEAMRLEKDNLITKQNQLILEISKLK---DLQQSIVNSKSEEMTLQIHELQKEIEMLRQEEKEKGSLEQEIQELQLKTEMMQKQMRETEDSLQNKCSELETQNNLLQGENKSLEEKLKSMLVIS-EENAILNDSVSSNSENLDVQKRIENLITENEQLKRQNIQLQEDNERQKSAFLSTEKELEANYKGLQKECESLLKAKNVLEENMNKLEAEYKIKLKALSEELHHLQSNRPVLFKSRTSSFEERNEKRVFRADTPEAGEVVEKDATELMEKLEVTQREKLELSRRLSDLSEQLKSKHSEVCH-LSEEVKSLKDEKDKVLAKCKELECIISHAKRENISNTEPKTKCFKGKGQEIATPAYESRNKTSAPSNEVGEGINVRENWGSGKAISHQITEVQEVQQMLKPEQPAFVEHLHEMTDGLTDETSLIAITQTENNNLQQQVNTLKSEQADLQLQMEAQRICLSLVYSAHVDQVREHLKAEKESELCSLKEELVCSHLQEMNDLKKVHQMELQTMKIQPADDELSSSQRLIEKLNEAITEECSRLTQI-FCDSWNKNSST--VIEAGDREQDIFNRPAAEKENLGMELTRHSGQIQAMQEHMEALLHKILEEYSRLRK-----LQTEM-MKDCKQVKEPCLSSSEPERRKEEEPNSLETRKESLQASSQSLMDPKIHEHCWKEIENVKTQLEEQHAQEVEHLKSYFQQQLKESEERYTAEIVHLQDKLRSDEVSSDYTSISPESQIKL---KESSKKVKCTQNPHQQREDSALELANEIEMKNDLDVV-QLLEKQY--Q------------------------------------------------------------------------------------------------------------------------------------------------------------------------------------------------------------------------------------------------------------------------------------------------------------------------------------------------------------------------------------------------------------------------------------------------------------------------------------------------------------------------------------------------------------------------------------------------------------------------------------------------------------------------------------------------------------------------------------------------------------------------------------------------------------------------------------------------------------------------------------------------------------------------------------------------------------------------------------------------------------------------------------------------------------------------------------------------------------------------------------------------------------------------------------------------------------------------------------------------------------------------------------------------------------------------------------------------------------------------------------------------------------------------------------------------------------------------------------------------------------------------------------------------------------------------------------------------------------------------------------------------------------------------------------------------------------------------------------------------------------------------------------------------------------------------------------------------------------------------------------------------------------------------------------------------------------------------------------------------------------------------------------------------------------------------------------------------------------------------------------------------------------------------------------------------------------------------------------------------------------------------------------------------------------------------------------------------------------------------------------------------------------------------------------------------------------------------------------------------------------------------------------------------------------------------------------------------------------------------------ERLEEE-I-AK--------------VLLLF-CYFKIC----------------------------FKIL-----------------------------------------------------------P---------------LLFVVPD---SSY---------------------SCNV--------------------------------------------------------------------------------------------------------------------------------------------------------------------------------------------------------------------------------------------------------  
------------------------------------------------------------------------------------------------------------------------------------------------------------------------------------------------------------------------------------------------------------------------------------------------------------------------------------------------------------------------------------------------------------------------------------------------------------------------------------------------------------------------------------------------------------------------------------------------------------------------------------------------------------------------------------------------------------------------------------------------------------------------------------------------------------------------------------------------------------------------------------------------------------------------------------------------------------------------------------------------------------------------------------------------------------------------------------------------------------------------------------------------------------------------------------------------------------------------------------------------------------------------------------------------------------------------------------------------------------------------------------------------------------------------------------------------------------------------------------------------------------------------------------------------------------------------------------------------------------------------------------------------------------------------------------------------------------------------------------------------------------------------------------------------------------------------------------------------------------DEFEDEKTALIHDQEQVFIQEREKAQAACQKEKETLSAQLQEKAARIIQLEKRVESLNCEIKETNCE-------LETLI---ERRDRENQEGGNLVAMLRSDIEQSKDERKKMQDSYECLLKWLIELVKATIAVEDLICSKIGLCLDNSMVSADSMESHSIMEEIGYAR------YFKAK-----EKSHLEKDELLDETLTEHSQLSLVTEEQYELSQYLYESVFAKP--EMAYENEEMILKICDRLRTAVERLLELVTEST----------------------KQLEKTHEIHAQFEEEFTRRNQETAQVVSQHQELMECLSEESEAKNQLVLELHKAEGIIEGYVAEKAALEEALNLKEESERRLVVELENMRERFQELTQEQAILG--------------------------LL-------------KEVELLAKEKL---ELECQAEKDHSNLCSQMKVLEMELEEQLRSNQDLTKQL-MEAAELKQQIEVLEKQLK--NQRQFMDEQAIER--EHERDDFQQEIKKLEEQLKLSAKLQTSGEPKEYGNYDLILQVESLQGEVKQKVDDYNKLLLEKEQKHQEITARDKEIEKLLTQVQELEH-----SSTEVSKTVNYLQQQLQKMKKVETELKQDKEALQQQQYNNLIQISALQSKLDEVRHGVPVEGTSGQ-GLKEQLQAEQES-------------LERKEGEIASLLDHLEQHKDKLTSKNEEILQLKLQLEVQKNLNTFTINQLQLENAQLKDLTKLNVKQNQDLDVSDSSALSFPQALLKEKNQEIDHLNEQLRRLKYELVNTLENKVVEDQKSEIEELRSIIEHLRGDQERLSKDKDE--EVEQLHGVIEKLQKELA--QFG------PVCHEVSDNQGDIFQFGLEKSVENLQKQLKKGLIDCQGDTDQGGRNALLLSKVRELEEELEFASAARKDLQQQLEEKESQIKMEVEILEKKCQ------NLQESSRQHFAELNALRLQYSALQEEYSLL-QTHISQREFEARIAASCVQELEDSVKKRELSILGKDKQVKTMADQRKTDEGELQYLTKQASE----LKTELKK--KDTSQAQDAYSREFE-VSRLDLHVQAMNQKEVSNQRETDELQGNMTKLKDQIKIYTKELEALRLERDELISQLELYKPKE---------------------VLKKGMEELEALEIHVDQSFASLTDNLEKVALFDVT-AHDTNKQSQVKQSEDSISIQKLDMISSSEEEQTLKKHHHQT-PSSPKITVNAGCTEGTLKN--FQDFIVDVTSWDSPEIMRKQDISMELQPVLN----LTPFS--EAESTGLEMVHSRSSLQ--GDNSVLLGFSYLYENGSEEAADGADVKSPVTSESTYSVDDQD------------------------------------------------------------------------------------MEKMILT-----VLKLVYEESHKILALS--------------------------------EHSFGFRELKSIHQTR-----------------------AVMEGWQKEGLALLN-AIQSLKDHLSKVAD------RGD---------------------------------------------KEEQRQMV-VEHIFSSDRNSLLSEIQELRAQLRMTHLQNQEKLQQLQETLTNVEDHGSKQEHQLRRKVELLEYKLQQEKCIVSDLHSTLSEEQKRVSKTCELLNQEKAAV-----SILKSDLCECKQENERLQNSLEEHQREISKLRSELESKEKDLTATLQELQSERVTETELRVLLEEQQLQHKKAEDEKKKALEDLQAALD---------------LQMVQNSKLSVSLEHEQIINDNLRKELQIEYSRCEALLSQEHDKVLELQKNLDA-----ER-NRSLELLNSLNHERVLTEQLSVQAKEDASCQRRELLLEQAFIRELQAQLGEERSRKAELAAIIEKTHQKAIRSKRQ------------------------------------LEAEVQMCCEETQ------------------------------------------------------------------------------------------------------------------------------------------------------------------------------------------------------------------------VEKEISN-------------------KLQVTLESLQSQKQEVIH--------------SSEAQK--EKE-A-KL-----------KEQLHLLF-------------------------------------------KTM---------------------------------------------------RD------------------KIKK---E-----------------------------------------------------------------------------------------------------------------------------------------------------------------------------------------------------------------------------------------------------------------------------------  
----------------------------------------------------------------------------------------------------------------------------------------------------------------------------------------------------------------------------------------------------------------------------------------------------------------------------------------------------------------------------------------------------------------------------------------------------------------------------------------------------------------------------------------------------------------------------------------------------------------------------------------------------------------------------------------------------------------------------------------------------------------------------------------------------------------------------------------------------------------------------------------------------------------------------------------------------------------------------------------------------------------------------------------------------------------------------------------------------------------------------------------------------------------------------------------------------------------------------------------------------------------------------------------------------------------------------------------------------------------------------------------------------------------------------------------------------------------------------------------------------------------------------------------------------------------------------------------------------------------------------------------------------------------------------------------------------------------------------------------------------------------------------------------------------------------------------------------------------------------------------------------------------------------------------------------------------------------------------------------------------------------------------------------------------------SVDELLDETLTEHSQLSPVTEEQYELSQYLYESVFAKP--EMAYENEEMILKICRRLRTAVERLLELVTEST----------------------KQLEKTHEIHAQFEEEFTRRNQETAQVVCQHRELMECLTEESEAKNQLALELHKAEGIIEGYVAEKAALEEALNLKEESERRLVVELENMRERFQELTQEQAILGEEWSKKWKTSESFTLTSKADEECETGLL-------------KEVELLAKEKL---ELECQAEKDHSNLRSQMKVLEMELEEQLHSNQDLTKQL-METAELKQQIEVLEKQLK--NQRQFMDEQAIER--ENERDDFQQEIKKLEEQLKLSAKLQTSGEPRESSLLQFDSEVESLQAEVEEKVDDYNKLLLEKEQKHQEIAARDKEIEKLVAQVQELEH-----SSTEVSKTVNYLQQQLQKMKKVETELKQDKEALQQQQYNNLIQISALQSKLDEVRHGVPVEGTSDQ-ELKEQLQAEQES-------------LERKEG-----------------------------------------------------------------------------------------------------------EVVEDQKSEIEELRSVIEYLRGDQERLCKDKDE--EVEQLHEVIEKLQKELA--QIG------PVCHEVSDNQD--------------------------------GRNALLLCKVRELEEDLEFASAAKKDLQQQLEEKESQFKMEVEILEKKCQ------NLQESSRQHFAE------------EEYSLL-QTHISQRELEARIASSCVQELKDSMKEREVSILGKDSQIQAMAHQREADEGELRYLTEQASD----LETELKK--RDASQAQDVHSPELE-VSRLDLHVQAMNQKEVSKQREIDELQGSTTKLKDQIKIYTKELEALHLERDELISQLELYKPKEQCDKENANCLELFCWGQRQGEVLKEGMEELESLETHVDQSFASPADNLCCNNRNKKADSMACTTKDSKKEWKAQLYIFHNDYLLQVEKILIMSMETAQGGLFSFGLNAGSACILPCLSATGSRDFIVDVTSWDSPEIMRKQDISMELPPALN----LTPFS--EAESTGLEMAHSRSSLQ--GDNSVLLGFSHLYENGSEEAAGRADVKSPVSSESIYSGADRDQDLKKTT-LFTVTFFHCSPLVLKKKKKKKKKKKNPPPTKKTQVGWMLAVLIEGEKNSDLQDDVRSGASAIDYCGMFPDIMES-VREKEILSPQLKTVLKLVYEESHKILALS--------------------------------EHSFGFRELKNVHQTK-----------------------ALMEGWQKEGLALLKVADRGDKEHLNFAIDG-----RGELLEVQSMFEKERNVLQIDLKFHFSNPGSGDEVALVEKLVYVV--KQQEQRQMV-VEHLFSSDRNSLLSEIQELRAQLRMTHLQNQEKLQQLQETLTSVEDHGSKQEHQLRRKVELLEYKLQQEKCIVSDLHSTLSEEQKRASETRELLSQEKAAV-----STLKSDLYERKQENERLKKSLEEHQREINKLCFELESKEKDLTATLQELEAERLTETELRVLLEEQQLQHKKAEDEKKKALEDLQAALD---------------LQTAQNRKLSVSLEHEQIINDNLRKELRIEYSRCEALLSQEHDKVLELQKNLDA-----ER-NRSLELLNSLNHERILTEQLSVQAKEDASCQRRELLLEQAFIRELQAQLEEERSQKTELAVITEKTHQRAIRSKRQ------------------------------------LEAEVQMCCEETQKEKEQNLELQHQRD---EQRIKEMQEIVAILDKDQNAKEPMTIEIG-KFNLQQQQKQQLEKIRQQLLFVAAHLIELMCKTVDK-TVNDWYVSNDEAVASLLQTLKELKSDLLSPASQVRKSF-CVLWVQFMREVLSDSCSVTDIFTQMLRS-------------------------KKSLVSEIVKEVAKSTDDSNPVQELE-RAAWQQERNILQNALKQAES-------------------KLAKATAETENKPAMEAF--------------NPKLQRLYRKY-L-RAESFRKALVY-QKKYLLLLL-GGFQDCEQATLSLIARMG--------IYPSPAD-LQLSASRSRPF---------------------------------------------------TKFRCAVRAVIAV--SRLKFLVKKWNKLNR-KN------------------AQAETVSQNTGSNTASGARTETLKQQQLTAVHVSSPPTWDTGLCHR---TSSARSVSCSSKSSHRSHSRLPPSTSEKSPVPT-------------QDPERSLTEYIHRLEIIQQRL----------------------------------------------------------------------------------------------------------------------------------------  
----------------------------------------------------------------------------------------------------------------ISLKLYVEQLLAQFRQRKA-----QTDGQSTSKKQKKKRKTASIKDEESIQDGLDTDRSRGEETSTCSSRRGAAAT-ADFAVTRTLHSGEIIKHIQAYTTEPESEISTAIEDYSSEVNGCSFVTRTAIPADLIREEEFGVGEMCSEHGMQHSLMQLEVMENELAGKQQEIEELNRELEEMRAAYGTEGLQQLQEFEAAIKKRDDIITQLTTN-LQQARKEKDETMRE--FLELTEQSQKLQIQFQHLQASEALRNTSHSCTAADLLQAKQQILS-HQQQLEDQECLLKNYQRKNEEFEVQITHLQGLIKTYEMEKQRNEEEDMN-KLQEKEALVEELEAKLKEEEKKSLQLKEKISDISKLHEELKEEISLRNLEI---SNMRIELTTYKQKERQCSDEIKQLMGTVEELQKRCYKDSQSEADIVQRMELETQRRLAQLQAELDEMHGQQIVQMKQELIKQHAVEIEQRLAQQKVELEKTSSLCLSDNVNQDQMHLMNIKINELNVKLQDADNEREKIKQELSQQMEVISAEKSLQQTKIEDLLQELNFSREQVQRAKQTIMDMECRLNEAEKYQ-FVIEDLKTQLASASEFRKELELKHEAEVTNYKIKLEM--LEREKDAVLDRMAESQEAELERLRTKLLFSHEEELSRLKEDLQKEHMVNMESLKDNLNMHHKQQLDGVKKEMSQKIEAMQLEKDNLITKQNQLVLEISKLK---DLQQSIVNSKSEEMTLQIHELQKEIEVLRQEEKEKGTLEQEIQELQLKTELMQKQMRETEDSLQNKCSELETQNNLLQGENKTLEEKLKS-MLVISEENVILNDSVSSKSENLDVQKRIESLITENEQLKKQNIQLQEDTERQKSAFLSTEKELDANYKGLQKECESLLKAKNVLQENMNKLEAEYKIKLKALSEELHHLQSNRPVLFKSRTSSFEESNENKVFRADTQEAGEVVEKDATELMEKLEVTQREKLELSLRLSDLSEQLKSKHSEVCH-LSEKVKSLKDEKEKVLAKCKELEFIVSHAKRENISNTEPKTKCFKGKARETATPPYESRNKTSAPSNEVGEGMVRENLGSEVQEVQQMSKPEQPAFVEHLHEMTDR----------LTDETSLIAITQTENNHLQQQVNTLKSEQADLQLQMEAQRICLSLVYSAHVDQVREHLKAEKESELCSLKEELIRSHLQEMNDLKKVHQMELQTMKIQPADDEVSSSQRLIEKLNEAITEECSRLTQI-FCDSWNEKSST--VIETGDKEQDIFSRPAAEKENLDMELTRHRGQIQAMQEHMEALLHKILEEYSRLKA-----LQTQLVKELWLSSSEPERRKEEESNCLETRKESLHASSHNLMDNLHID-PKIH-ELCWKEMENVKTQLEEQHAQEVEHLRSYFQQQLKDSEVRYTAEIAHLQDKLRSEEVSSDYTSISAESQMKL---KESFRKVKCTENPHQQGEDVALELANEIEMKNDLDVVQLLEKQYQ--ERLEEEIAKVIVSMSVAFAKQSELSRIARQKKEETQTQIEHRQGMHFEMRRECSIEEVDGPLKKATEKSKHEELKSLCKELSEESGEMGLLGEQLCSNSQSGYVLRQTTRESVTSERLFSHVQGHTAEETLCNNVVAASDMETSSQLLIYEERLEDMRQELVRQYQEHQQATELLRQGHMQQMERQKENQEQLLAELESLKLQLAERVSMENDNLAAERERMLLEELKSLRQHPVPGKERLFCELQNSSTQTE-------NENEDQNDVREQITEDEDEGRKPDEVSSALLSKERHVYQKAN-EKLMKILLEVVKTTVAMEETIGRHVLVLLDRSGKVQPSKPAG-WDTEAEDSVKPSIHEDRILRVCVKTTVFPESCSSYHGSSMGDDDITMWSGTAEEGLLSQH----LAESGVELDPENEELVLNISSRLQAAVEKLLEAINETS----------------------NQLEHAKITQTELMRESFKKQEEATEFIRYQEELQERLSEETKAREQLALELNKAEGLIDGYADEKAFLEKQLQEKIDVIDHLEQELLCTGNKLQELEAEQQQIQEEKELLARQKD---AMRADAGPVEQQLL-------------EETEKLMKEKI---EVQRQAEKEYDDLQKQVKVLEIDLEEQVSRFIELEQEKNAELMDLRQQNQALEKQLE--KTRKFLDEQAVDR--EHERDVFQQEIQKLEQQLKVPQRSQPVNEHQ-------SREVEQLTNHLKEKTDKCSELLLSKEQLQRDIQERNEEIEKLECRIRELEQA--LIISADNLQKVEERKQFGTIIVKGELPLEIQLQAEREAVDRKEKEVTNLEEQLEQFREELENKNEEVQ-QLHMQLEIQRKE-------------STTHLQELEQENKLFKDEMEILGLAIQKSEDGTI---KDHHLVAGKLAHIMQEKEQEIDNLHEQIAKLQQQLEG-----------------------------------TTDNKIFEEQNEHIRELEAQVECLKSDQERVKKKNDE--EIEQLNDVIDKLQQELANIEQ-IPSDITAFHEDADSLKHTLETVLAEKEALEKQVESINLEASQTKNELEETKLEMNQLKQEINILRKEREEVVEKYKCGLMKGDREKTEVGSNRKTEKVDEGSCEMTELLDQTQLRSSDENTRVTISKMEVQLQQL-QACIKEKDSELCQSYNEIKDLKEQGKAERDMLGNKILELEKALVEKVAAALVSQVQLNAVQE----QGKFLQEIQKASKCLEEASKHPLT-EGWNDTTENEVESEVSLLTQRLREVEEQLAKTNHSLELEKENVKVTQQESKLKEERLLELQQLLEEVQEKHR---------------SEIQKYIKQEVEAQHTERQNDNALSNELELEKVKEEAAAAKEELSSYREETEKLQKELSVKEANLVHLREDLCKVKEELVQVEEKLANYMRMEKNMAKTESRKDAEILCDLLTSESTSTGKSTSSQTDKTVNVSSY-IQTSPVLVKNAEIQID------CSS---EEIAEIIREFNEKIEQMQELHAAEIMDMETRHISESEALKREKFVAVQVLTDECNALKEVIESLRAKEYF---GFCFCS--------CLDSSSDWSQGMYLTQTQGSDTISE-GPDEG-ETSTDLLPQKIKGLLRAVHREGVQVLSLTEFPYGEREMP--------PLKQGPESWLEERKAFLSTISSLKDLISKMQLHREAEIYASSEPPEGDSDWRGELLRAIQQVFVTEQNVLLAVFQTELAELGTRDAVILMSQLEHRLQEQAINQRTAMDCLHDADRRSLLMEVQVLHAQMNS----MKNNPKREQEIDSKSQEMLEYSMQQKQSQIVEMQVELRSVKDRAAELQEQLNSERMMSAELKNELAQAKLELETTLKAQHKHFKDLETIRTEVKEKAAELDVLKDTMASEQ-----KKSRELQWALEKEKAKMERTEERRREELEDLKFSLEDQKQKNLQLNELLEEEKQLSSDLQQKIESQEALSAAQLSRERGRNSELQVLLE---------------SEKVRALEISSALEREKE----LCAQLQSAEHKGQAGTPNPSEELLKELQKQMD-----EKHDRIVELVSEMEKYKLESVQVRQQMEKERQIQRKALQAEQDANIITQKKLHELESKVEDLQWQLGQKEQEVHKLGNETKK-------------------LQEVIKELQMKEQESEGKKEAERTPSHNQNEVQTTWDTPNE---RTRNWVLQQKMEGAETNESTYATLTGGGG-DLSAATAILEAVRQKLQSASPKLKQLAQKAASRLQF-EAADDQDFIAIQNAIEEVISELQKLPGVSCLEELKLAL-PPGTPSS-SLTERLLRQNAQLTGFVSRLSEEKNNLRNAVMKLEEELRRYQLRRTS--GEHSSKHSSDLGVNIDTLVASE-KENWNREKLSLQKSLKQADA-------------------ELSKLRAELRSEAFLRELGSDS---------ENVILRRIYGKY-L-RAESFRKALIY-QKKYLLLLL-GGFQECEEATLALIARMG--------GQPSYTD-LEIITHHSKGF---------------------------------------------------TRFRSAVRVSIAI--SRMKFLVRRWHRVTG---------------------SGILSLSRDVFSQNTGNELRPDSF---SSGMDLYGEQRHS-YRSR-SDMESPRSPIN-FQQKFHSMHADLNPVSFACSQLQN-------------YDPERALTDYIHRLEALQRRLG-SVQSVST-SYTQLHSAMRR-------------------------------------------------------------------------------------------------------------------  
--------------------------------------------------------------------------------------------------------------------------------------------------------------------------------------------------------------------------------------------------------------------------------------------------------------------------------------------------------------------------------------------------------------------------------------------------------------------------------------------------------------------------------------------------------------------------------------------------------------------------------------------------------------------------------------------------------------------------------------------------------------------------------------------------------------------------------------------------------------------------------------------------------------------------------------------------------------------------------------------------------------------------------------------------------------------------------------------------------------------------------------------------------------------------------------------------------------------------------------------------------HSVSDG----------LAEDSSCMKLVQGEN-KLQEQLDVLKAEQSDLKLQMEAQRICLSLVYSAHVDQVRECMENEKENALSALKKELQFHHAQELCELKKSQLDLQKIESIK--TEEVQPALVDLERLSKTVAEECSKVIQV-RNKSTPRKGNS--LLEELAMRKAALGSGVSERENLLLMS---RADAQVLQGTLQGILNKIVEEYQKLLE-----YHVQFMKDIKKVITFFHSNQRERNTNLTLAYVEKNVESLPTPSQDCVG-PEVITSPSWEDIENFKSQLEEQHAQEIEHLRSYFHQQLKETEERYTVEIMHLQSRLQDTNESSEHFSISVGSQVN-----EVEEKEKHTENDLQQSTDRIVEIIGNPVEMISPMKVVILEKQYQ--EKLLQEIAKVVVTMSIEFAQKNELARITNPKEDEMSLMVPKQTGQDCNFSIKECHKGLDS-HRNEKRDAKCEEFKSLSREISEECGETLSLEGQFQPDVKPAVVLGLHNVDPSFPTDTSSNVKDLMPKGSLDANEVSTSSTASSSQISLYEERLEDMRLELVRQYQEHQQAIEILKEGHMQQMESQKENQDLLLAELDRLKMQLAENITLDNDSIITEREQMLLEELESLKKQTIPEREKVSHEMKDNGTQTQ-------VENVCQNESKELILESEVEGGKQEEISPDIISKERYAYLKKANNKLTKILLEVVKTTVAVEETIGLHVVGLLDRSSKSQLPFKMLDWEARTDDSIKPSISVG---------YEPEKTSSSYHGSDLEDDGHSMWSEGADEGFELTQ----YLG--MDIDSKTEELVLNISPRLQAAVEKLLETINETT----------------------TQLEHAKVTQTELMRESFKREQDTTELLRCHEELQERFDEEVKAREQLALELSKAENFIDGYADEKALLEKQIQDKTDAIDHLEQELLCTGNKLQELESERQQLQEEKELFFRQKD---AMKADAGPIEQQLL-------------AETEKLMKEKI---EVQKQAEKEYDYLQKQVKVLETDIEEQVNQFFELEQEKNAELMDLRQQNQALEKQLE--KTKKFLDEQAIDR--EHERDVFQQEIQKLEQQIKIPQRFQPVSENQ-------NREVEQLISHLKEKTDKCSELLLSKEQLQRDVQERNEEIEKLECRISELEHA--LMTSADSLQKVEERKQFGSSTIRGELPLEAQLQAEQEAVDRKEKEIINLEEQLEQFREELENKNEEVQ-QLHMQLEIQRKE-------------SATRQQELEQENKLFKEDMEKMGLAIQNPNDTSI---KNHHPVAGKCVQIMHEKEQEINDLSEQISKLQLQLEI-----------------------------------ATDNKVIEEKNEHIIELEAQIEHLKSDQERVKQNSER--EIEELNEVIETLQKELANIGHK-ILDLSSTQEDADNLKHQLDAVLAEKETLMKQVENYDVELSLAKNELKETKLIISTLEKELDGLKGEYEIMAERYKNIHMKSDYSESTIKEKTENSEVE-NKRKASILKNEISHHSVAENAMESSSNAAMKLQQL-QESLEEKESELKHCCNQFKDLKEQAQAESEVFKQKIKELEEKLEQNVAAALGSQAQLSVVLE----QSELLPEMDAISETAKEAITSVQS-EKVSSLAGEDAASRLSELLEKLTEMESELKNAQINLQLEKSKVEVAQKKEKEKEEKLTELQHLLVKVEEKYKKEKMQ---------SSQKGKIQTTQEKTKLDENQERNILTADPELERVKAECAAAKEELSSYIENTEKLKEQLTVKESSLLHLQEELKQTKNKLAQAEEKIEQKNFEQSEGIRTNR-------------------TNASCQTEKMLHIHSC-NQTSELLVKNEGTQIDVQNG--CSS---EEVAEIIREFSEKIDKMQELHAAEIMDMETRHISESEALKREQYIVVQELTEECNTLKDVIEALRNKEGIP--GVTQSATSNARDGCSSDSSSDWSHGAYPAQTQGSDTLSEEARDES-EASTDFLPNKIKGLLRAIHHEGVQVLSLTEFPYTESDKY--------SDKQGPDSWLEERKTLLSAISSLKDLIAKMQIHRENEIYCKQGLPKNILDWRTELIYAIQEVFQKEKDVLLAAFQTELAEGGIKDVMTLMDHIEKRLQEQGTYQRTAMDCIQNADRRSLLMEIDVLHAQLNN----RKTDPKGKLEIEPQCQGNIWFPSQEKKSQNMEMQMELSSTKDKATELQEQVNSERMMVAELKNELGQTKLELETTLKAQHKHLRDLEAIRNELKGKAAELDLLKDTVVSEQ-----KKSRELQWSLEKERTKVERSEEREKEELEDLKYSLENEQQKVIELTNLLELERVLSNDLQQKIESQETLNAAQLSQERSYNSELQVLLE---------------SERFRVLEVSGALEREKE----LCAQLQMADQKMQDGSFTPMEELLRELQKQLD-----EKHDRVVALVSEMERYKLESVQLKQQMEKERQIQRKTLQTEQDANILAQKKVHDLESKLEDLQWQLGEKRQQLHQLQHEGKK-------------------LQEKVQELQNKEQRDEAEAKVENTTCHNLN--EVTWDTSNE---RTRKWVFQQKVGGSETKELSYSALIGIGE-DAIKECQDLQMARQKLRNVSSKFKQLVNKAAGRLQF-EKSDDEDFVGIQNDIEDVILQLGVLVGLPNLESEAPPL-PNNLPSSASLTERLLKQNAELTGFVGRLSEEKNNLRNVVMKLEEELRRYKQMGPC--ADSTFRLSLHDGGNIDTLIASE-REIWNKEKLSLQHSLRQAEA-------------------ELAKLRAELRNEAFLRELGSDS---------ENAALKRIYGRY-L-RAESFRKALIY-QKKYLLLLL-GGFQECEEATLSLIARMG--------GQPSYTD-LEVITHRSKGF---------------------------------------------------TRFRSAVRVLIAI--SRMKFLVRRWHRVTG---------------------YSLIGINRDGFGQNSGNELRTDSV---SSGLELYGEHRHSSYRSR-SELESLRSPLC-YQPRFQGIHSELSPVSLACSQLQN-------------YDPDRALTDYINRLEALQRRLG-SVQSAPA-SYTQLHSGIRR-------------------------------------------------------------------------------------------------------------------  
------------------------------------------------------------------------------------------------------------------MDAWLSGLLAQFRQRKA-----HTDGHAP-KKQKKKKKASNIKDDELVQEGLDIDQLQGDDASTYRCQR-GAAATSDFAIIRTLHSDEIIKHDQAYTTELESEISTTADDYSSEVNGCSFLTRTGASTDCIREEEFGFGENYSEHGMQHSLTQLEIMENELSGKQQEIEELNKELEELRAAYGTEGLQQLQEFEAAIKKRDDIITQLTAN-LQQARKEKDETMRE--FLELTEQSQKLQIQFQHLQASEALRNTSHSSTASDLLQAKQQIFT-YQQQLEEQEQLLKKYQMKNEDFEVQVSLLQKRVTDFEMEQYRAE-DDT--------------------------------------------------------------------------------------------------------------------------------------------------------------------------------------------------------------------------------------------------------------------------------------------------------------------------------------------------------------------------------------------------------------------------------------------------------------------------------------------------------------------------------------------------------------------------------------------------------------------------------------------------------------------------------------------------------------------------------------------------------------------------------------------------------------------------------------------------------------------------------------------------------------------------------------------------------------------------------------------------------------------------------------------------------------------------------------------------------------------------------------------------------------------------------------------------------------------------------------------------------------------------------------------------------------------------------------------------------------------------------------------------------------------------------------------------------------------------------------------------------------------------------------------------------------------------------------------------------------------------------------------------------------------------------------------------------------------------------------------------------------------------------------------------------------------------------------------------------------------------------------------------------------------------------------------------------------------------------------------------------------------------------------------------------------------------------------------------------------------------------------------------------------------------------------------------------------------------------------------------------------------------------------------------------------------------------------------------------------------------------------------------------------------------------------------------------------------------------------------------------------------------------------------------------------------------------------------------------------------------------------------------------------------------------------------------------------------------------------------------------------------------------------------------------------------------------------------------------------------------------------------------------------------------------------------------------------------------------------------------------------------------------------------------------------------------------------------------------------------------------------------------------------------------------------------------------------------------------------------------------------------------------------------------------------------------------------------------------------------------------------------------------------------------------------------------------------------------------------------------------------------------------------------------------------------------------------------------------------------------------------------------------------------------------------------------------------------------------------------------------------------------------------------------------------------------------------------------------------------------------------------------------------------------------------------------------------------------------------------------------------------------------------------------------------------------------------------------------------------------------------------------------------------------------------------------------------------------------------------------------------------------------------------  
--------------------------------------------------------------------------------------------------------MEEEDQERKRKLEAGKAKLAQFRQRKG-----QADGQHAAKKPKKKKASTGSKGQQTAEDAQETSYSQSHHS-HSQSTE-GASATEEFSIMRTLSQGESVKHDKTYTIEPESEISSTADDYSSEEE-----------------EEFEVRDTYSEQGTCSSLTRLEVMEDELAGKQQEIEELNKELEEMRAACGTQGLQQLQEFEMAIKQRDDIITQLTTN-LQQARKEKDEIMKE--FLELTEQSQKLKIQFQHLQASEALRNNSHTSTAADLLQSKQQILA-YQQQLEEQEHRLKLYQKDNESYKAHNESLQAKIQDMEQLKELEL-SY---------------------------------------------------------------------------------------------------------------------------------------------------------------------------------------------------------------------------------------------------------------------------------------------------------------------------------------------------------------------------------------------------------------------------------------------------------------------------------------------------------------------------------------------------------------------------------------------------------------------------------------------------------------------------------------------------------------------------------------------------------------------------------------------------------------------------------------------------------------------------------------------------------------------------------------------------------------------------------------------------------------------------------------------------------------------------------------------------------------------------------------------------------------------------------------------------------------------------------------------------------------------------------------------------------------------------------------------------------------------------------------------------------------------------------------------------------------------------------------------------------------------------------------------------------------------------------------------------------------------------------------------------------------------------------------------------------------------------------------------------------------------------------------------------------------------------------------------------------------------------------------------------------------------------------------------------------------------------------------------------------------------------------------------------------------------------------------------------------------------------------------------------------------------------------------------------------------------------------------------------------------------------------------------------------------------------------------------------------------------------------------------------------------------------------------------------------------------------------------------------------------------------------------------------------------------------------------------------------------------------------------------------------------------------------------------------------------------------------------------------------------------------------------------------------------------------------------------------------------------------------------------------------------------------------------------------------------------------------------------------------------------------------------------------------------------------------------------------------------------------------------------------------------------------------------------------------------------------------------------------------------------------------------------------------------------------------------------------------------------------------------------------------------------------------------------------------------------------------------------------------------------------------------------------------------------------------------------------------------------------------------------------------------------------------------------------------------------------------------------------------------------------------------------------------------------------------------------------------------------------------------------------------------------------------------------------------------------------------------------------------------------------------------------------------------------------------------------------------------------------------------------------------------------------------------------------------------------------------------------------------------------------------------------  
----------------------------------------------------------------------------------------------AMDEERQKKLEAGKAK---VSLGFSVFSLAEYRQRKAHADS-----------PGEDSEGDPQGAVEVE-------DEVSGGGQYGSQEGNKDPPTTEFTFARTLRSGDTIKHDQTYTIEPESEMSTTAEDYSSEVHEMIFAQREQKQKTFLKSAAFPVQEEVESLQQVTKGG---RMEDALAAKTQAVEELSRELEEIRAVFGTEGVHQLQDFEAALKQRDGIITQLT-TNLQQARDEKDEIMKE--FLELTEQSQNLHIQFQQLQAGETLRNTSHSSAAADLLQAR-QQLLQYQPQLEELQLQLSQMEVQTAIITEHELSLT-LLREELAHVGRTTDENIINPSDEKDLIIAEQERVISERDCSLTQLEDELESSERRLRDLQRQMAAKDSELESCK---GELSASRHKERMSSNEIMQLMGTVEDLQKRCHQGSLSEGDTIQQMQGETLRRLELLRAELDEMYGQQIVQMKREVNLQHAAKVEQMTEQHRAELELLKAQQLSQ-SSTVCVEVD--TLNAKIRELQETLEQSQAMQHRARQELSQLAQEKVDLQAKVEDLLEDLRSAKEKVEQVSHSLVSQESQQVELRRLQ-EATDNLKSELAAAQKAAQEAEVQHDSETTNYKIKLEM--LEREKDAVLDRMAQSQEAELDRLRTLLLFSHEEELTLLREDLQRENFLNTENLLNEAAIKHEKASATLRSAYEDELHLLRREKAEFATERDELFHQIVGLK--EDLKLALHSSKADELVQQLQELQVELDELRKGGGERARMEREIQSLHKNAEMLDSQTKERELCWESKRKQHQLEKEALIESNKALKE------------------------ELNSKVLKIESLVAENNKIQQRVVRLGEEIETQRSTFSFAEKNFEVNYQELKEEYKCLIEAKTQLEERTLKETLEFEAKIASLQSQVRALEESGGDLKMEDVS-------------TDGREQAVIEKDTTELMEKLNVSLSENERLAERLSEATDRLMFTESKVGR-LEEELARARQANVEVAPQSEEI-KALR-----SLLWAAEPERDATRQTSELHGPR----------QTPSAAAAGEEEPVDGRSPPHKPTDPGS--------------------------SRNRRRRRSKQERKLLSAFARVTFERSCQAEPCCERKKRSRTISNYKSTQGECRLQFEAQRISLSQMHAAQLELLQEGSDARAHILELK-LQSWKDRGEQDDPKTFKRHNLLKAVSEECNEIIQV-ICGCIVMFGKQFLESVGAEDQPPPSVERPDTSESSSIILEARELCR-DLQQARERIEKEHYRLTLLQTQ-----LRADGNKKKKSKQSLIK---RKTNTLFIY---AIYNLD-------PTISLPEEFQRLKVEEQEKQLQLEESHRQEMERLRAHYQQQATESEERYATELFMLQQRLQELAG-AQTHD-SLSVNMIT---PLVFNTCLTFFPTVMLKGNDSHINWARSSGSSPGAAGPIGLEDTQHWERVQEEVAKAIVQMSVEFAQQIELARINKSAGQRSAAMQTG-SDKEE--------VED--RERMTPRASLPSGARLEEVEKERLERELEQRNAEIRKLKEELQKTESRAEQVRLSFKCVIAVAVRFSVLSKMICSISDTVMIIHLNLHLHCMLSFPPDTMKRTMVRLKSLSLTSSVCGRSDGRSAAAFQSFFQEMELLPLQVTVPQHKDSSVSLGRRELEPTPTFSMTGLLSVFSLLPVQALKRREDSQEE-L-------EEEEDRGGEK--AGGGKLPSEDVGGSSD---------VLRNANEKLSQVLVDVLKTTAAAEETMGLHMQSLRDAFSGAQQAAPPESTTPTATRPRATTGPARSHAA-----GLSA-ESCQGSETAADHVSLWSGDTDDGQ------QMMERLLL------CNEEYLMGISRRLQTALEKMLMAITDTT----------------------NQLEHARLTQTELMRESFRHNQEMSELLQKQEELQERLSEEARAREQLALELHHAEDLIDGYTGERAGLEEQLRQKEELQLSLEQELQVTGSRLHELEQERLQMQDERDLLSRQQD---AMREHAGPRELHLL-------------EETEKLMKEKV---DVQRQAEKENADLLKQVKLLEAELEEQVNRVIELENAQSTESGDLRQQVQALEKQLD--KNRRFLDEQAVDR--EHERDVFQQEIQKLEEQLRNPQKLQAGSEQRNKEPLSKSNHLKKQLLKLSSSLSCKSGDEGRCKSNLAEDDSLSIQLYPLFAYLTIFFTS--LDICFSW-LTTNKNVSSLVSGKKLHLKNPLELTPIHQQMFTCRLIICNLEEQLEQFREELENKGEEVQ-QLHMQLEIQRKE-------------ISSQQQYLETKDSMLQQDCH-LHCVSFLTSTTMVTLERTLFIDSHHIIIVMEEKDREIALLNEQITKLQHTGT------------------------------------SSDNKEIDERDELIKDFESQVECLRSEQERLKRNSEE--ELDQLNAVIDRLQQELANIEQK------PAAEEDED--AKEESAVGAP--SKEEYDEMKQRMDMATGEINTLKTKHTALLETYLRLKESAEALAET------ESAES---ELEDALREKTA------GLVVMQAQVQALEQSATSKVEELDLRIRQL-EVEVGEKDSELSRCLLLLEQTRSHAADLQQRVSNLEENLRENLDAALVNQAPPEAFQQQQSK----E-----S--EGNQDLQSRAEPDMD-FSGIGQTRQAPKGKVVHLTQRLRELEVGLSGIQKDQELQKQLLSSSEEEVLEYERRLSVLMDLLGNNVRAYRLHPK---------LTNPSSNRVHSVTK--HAVASHEREITISRLKGEHVQEAASTKEELNTYKQHNKKLLEDLQFRDVSISKLKEELQEMRGNVDTTKHELQLFRQHNEKLQISSERSSRSQPVPSASSSSATQPK--SSRLNSSSTQQ--HVTHSFAQTEPLQMS-DLDPE--SESANRDEIEEVIGEFEEKIVQMQELHAAEILDMEARHISESESLRRDT-QALEDECKALKAVIDKLRSTQVR----------AGKDSCVKTTADSSSDYSQRTGFDFPSLQQEFRSTPEGARR-ETD-DPLPDCIKTLLREVHQEGMQVLS-LSELP-LAEGE-------SGSQFDAPGWLKERDALLATVESLKGLITQMQTHRETQVFDLPQTSG-GVDWRAELLDAVRQVFTRERSVLKSALYSQLDLLDISDAIVHLNQLERRLAEQDTQHREAIGSLHTADRSSLISEIHQLRGQLERLH--QDFCPSAALPVASASRERS-------------VKKQGGGADGAAEADMLLVEGLKSELSQTRLELETTLTAQHKHLK--ELDTL-----RAEASQKAAEVDALNDRLTEER-----KKSRDLQWAMEKERCRTGNDEESKREELEDLNFSLEEQKSHVAQLTLTLEQERQVSSRLSRQAE----QERLS--LHS-RLKEFQVQLE---------------TERAKALEMGSALGRERELRTGASSDGGPSSEQRVDEDRRELDKEESLLARLQR-----QL-DDKHAQVVHLLGQVEAQKLEVVRKEEELTLGSQRSTQDQEALLEARAKLERLVAQMSEVQEQLERETGRRKRLEGEKER------------------LEERLNQLAE---QKEGGEVSADLQAIVFEPKQAAVHSEPTS---RTEDWVFQQLEASPTGRTSGPQHGPQHGP-QHGPWRTVDKILDKLHLVSAKICGMANENTGRLTAE-VDSEELSWLQSKVNEVITMLQQSPVLPSVPESVSLLPG-GSSTSSSNTLTERLLRQNAELTGFVSRLTEEKNDLRNHTLRLEEELRRYRHAGPGSADRFSTRRGVSKADSSGLVLSEE-REAWTREKVRLEKALLLAQA-------------------QVARLRGEIR-ADNLREMTGPE--------ADNAALKRMYGKY-L-RAESFRKALIY-QKKYLLLLL-GGFQECEEATLSLLTRMA--------GRPALPVGEESPIQRRRGL---------------------------------------------------TRFRSAVRVSIAI--SRMRFLVKRWHKATGMSS------------------STSCGVNKNGAGEISDSEVRG-SPYLHPGSVDTYRERGSGGGVSTGRGRSGRESPRSAVSSTQHRFHTAGDHGALTCSHLQS-------------YDPDRALTDYISRLEALQRRLGGVTSGAS--SYAQLHFGLRR-------------------------------------------------------------------------------------------------------------------  
--------------------------------------------------------------------------------------------------------------------------------------------------------------------------------------------------------------------------------------------------------------------------------------------------------------------------------------------------------------------------------------------------------------------------------------------------------------------------------------------------------------------------------------------------------------------------------------------------------------------------------------------------------------------------------------------------------------------------------------------------------------------------------------------------------------------------------------------------------------------------------------------------------------------------------------------------------------------------------------------------------------------------------------------------------------------------------------------------------------------------------------------------------------------------------------------------------------------------------------------------------------------------------------------------------------------------------------------------------------------------------------------------------------------------------------------------------------------------------------------------------------------------------------------------------------------------------------------------------------------------------------------------------------------------------------------------------------------------------------------------------------------------------------------------------------------------------------------------------------------------------------------------------------------------------------------------------------------------------------------------------------------------------------------------------------------------------------------------------------------------------------------------------------------------------MDEDERQRKLEAGRAKLASFRQKRAKSNAGEAKKTQKRKGPTDPKNDSPAQDCHVEQTPPSASATKTNHKDALKPDDSDVEGDESSKQPPTP-----ERSPSPVEVLGE-------------GELAALTGKEQ-----LKQLQLAVEKRNDIIAKLSSNLQEALASRDLVQLEAHSLAEQIRALQKQLQQTSVEFQRIKVQSGAEVSSVSQHHHSLPSQDL----------------------------------------------------------------------------------------------------------------------------------------------------------------------------------------------------------------------------------------------------------------------------------------------------------------------------------------------------------------------------------------------------------------------------------------------------------------------------------------------------------------------------------------------------------------------------------------------------------------------------------------------------------------------------------------------------------------------------------------------------------------------------------------------------------------------------------------------------------------------------------------------------------------------------------------------------------------------------------------------------------------------------------------------------------------------------------------------------------------------------------------------------------------------------------------------------------------------------------------------------------------------------------------------------------------------------------------------------------------------------------------------------------------------------------------------------------------------------------------------------------------------------------------------------------------------------------------------------------------------------------------------------------------------------------------------------------------------------------------------------------------------------------------------------------------------------------------------------------------------------------------------------------------------------------------------------------------------------------------------------------------------------------------------------------------------------------------------------------------------------------------------------------------------------------------------------------------------------------------------------------------------------------------------------------------------------------  
-----------------------------------------------------------------------------------------------------------------------------------------------------------------------------------------------------------------------------------------------------------------------------------------------------------------------------------------------------------------------------------------------------------------------------------------------------------------------------------------------------------------------------------------------------------------------------------------------------------------------------------------------------------------------------------------------------------------------------------------------------------------------------------------------------------------------------------------------------------------------------------------------------------------------------------------------------------------------------------------------------------------------------------------------------------------------------------------------------------------------------------------------------------------------------------------------------------------------------------------------------------------------------------------------------------------------------------------------------------------------------------------------------------------ALELEALRLSLTSLHTSHMEHCQENVKQDQTAAQTLHQLDVDHSKQSQEQEGRLLHQQKME-----EQWDIRVAEERAFLEEKHSKDMQVKLQD-ILALKAQWQME--------------KKAFQESFSDIQNTLTLTQAELTS--------------TKSSLTETTEALSEAKATLREVQAELQQAQAR-------MQELQTSSK-------EQLHKLQEELKQVLKVVL-LEEEKILLKQNSEEEMGQLWTQLESMRASRRELGELKEQLLARSSHVDDIERLKVEFNEQKKEIKEQNEAELENLRRYFEKRLHASEESYKEEIALLQMRLVESALEESVILTSEDCTLSQVQVEEKTHVPDVAKFEEQVPKGYEFSLEQTRSDITHHYHKELQEQKTMHALELEQLRAKVSDVHLKVEIEQRMWCLTEELHAKKSLENLSKQHDEEMQQNSQKHLLSNSKQLDQLEKALNQEREILKALQQKTKSEVSAIN-------SEEGENKFLTRNPKKGPQSPMMKPETRYDIELEKAKTCMSAEIKELTIQLQKQSEEKLHQAQQRHTAQQNSLNASHKSQLAAVVAELESKHRAELVALEATLH------------SKRKETNQVQLEALEAELSRKHQEETDELEKRMLGNMDTLEATYLKEVQALRNDIVQLKEKHKQELIVKDAEH----------------------KNILEQLAREQLSIREELRK-----ELAQLHMEKFKAMAAELSFVQKE--QQHNTALQDLTNTYAAEKDQELRGTSARELQAARRELEEESSRQRQHYLEEVELLKVRSEEKLQDKIN-QLRAEVGCLQGELQ--------------GKSAEMETMD---TLLQRRERESQEGANLLKMLTDDLQAAKEE----KFKLHHTNEKLAKVLVEMFRGIR--ATEEQIGQKINVRTKSSEQATQQRSSTENKDAQDCKVILMILVCVS------------GISVADLTSDDLEMTQLLCESLLVSDSQINPVGEESVLNACCRLRQA--VDMLLDLLNQANTQLEETRNVHLSLEEKFSKGKEDSVQLLEQHKLLLEQLDEEAKMKSQLQLELHKAEG-LLEGYVAEKAV-------------LEESLQQKENQEERLVEELEDLKTQLQQMQGFAA----EVQSLRLKHQELVEENAILLRQ---------------------------------------------------KEHLSAGLGEREKALLAETERLTQDRLDLQRQAEK--DHKTLTVRLRNVERELEEQETMVLETEMHNKTHTEDLNQRVQALEKQLKHNRQFIEEQAVEREHERDEFQQEIHRLEAQLRQPTNVDHRGSR--------------------------------------------VRAGRGQRKPCEMLKFKLETL-HAIIKDKTEDYDSLMAANQQAQRDLTKRNEEIN---KLEQQQLSSRLQISALQSKLDETRH--------------------------------------------------------------------------------------------------------------------------------------------------------------------------------------------------------------------------------------------------------------------------------------------------------------------------------------------------------------------------------------------------------------------------------------------------------------------------------------------------------------------------------------------------------------------------------------------------------------------------------------------------------------------------------------------------------------------------------------------------------------------------------------------------------------------------------------------------------------------------------------------------------------------------------------------------------------------------------------------------------------------------------------------------------------------------------------------------------------------------------------------------------------------------------------------------------------------------------------------------------------------------------------------------------------------------------------------------------------------------------------------------------------------------------------------------------------------------------------------------------------------------------------------------------------------------------------------------------------------------------------------------------------------------------------------------------------------------------------------------------------------  
---------------------------------------------------------------------------------------------------------------------------------------------------------------------------------------------------------------------------------------------------------------------------------------------------------------------------------------------------------------------------------------------------------------------------------------------------------------------------------------------------------------------------------------------------------------------------------------------------------------------------------------------------------------------------------------------------------------------------------------------------------------------------------------------------------------------------------------------------------------------------------------------------------------------------------------------------------------------------------------------------------------------------------------------------------------------------------------------------------------------------------------------------------------------------------------------------------------------------------------------------------------------------------------------------------------------------------------------------------------------------------------------------------------------------------------------------------------------------------------------------------------------------------------------------------------------------------------------------------------------------------------------------------------------------------------------------------------------------------------------------------------------------------------------------------------------------------------------------------------------------------------------------------------------------------------------------------------------------------------------------------------------------------------------------------------------------MEPLQSRTITPDQSTQRTHSPGSLSISDNFSLVDSIDAEQVHELEGIDQTAPPSP-----------------------LGSTSSMSAQEWASDGYGSNVSSELGARLRVELEQTERLDAQFVEYLRCRGMNPTVNTDSAAGSMSYTEDLLSPELQGLLKKVYHESCKILTLSQRRVSASIKPQVLAYSCSQTQ----SKDNSGPVDCENE---------------------------SQLPHPPMSWQQEKRALQETVIALRELLCRMAQRHSQMDFCEDNWRTAQLQIDGTVDVQMRTELEEKQKQLQCAHDALKEHKSNILSLRLTIEENDEALQREKSR----------VQELQCELEQEKALNLCKEKDKEEQREALQVSSEELKAEVLSLKSQVKQ-EMVTCSNLRQELQIEQSRSALLEKRLEDTLKMLEDGRQHSVQQQELNLKEKNHLGQLLDEAESRLADMHSKLADCHRKIDDE----------------------------------------------------------------------------------------------------------------------------------------------------------------------------------------------------------------------------------------------------------------------------------------------------------------------------------------------------------------------------------------------------------------------------------------------------------------------------------------------------------------------------------------------------------------------------------------------------------------------------------------------------------------------------------------------------------------------------------------------------------------------------------------------------------------------------------------------------------------------------------------------------------------------------------------------------------------------------------------------------------------------------------------------------------------------------------------------------------------------------------------------------------------------------------------------------------------------------------------------------------------------------------------------------------------------------------------------------------------------------------------------------------------------------------------------------------------------------------------------------------------------------------------------------------------------------------------------------------------------------------------------------------------------------------------------------------------------------------------------------------------------------------------------------------------------------------------------------------------------------------------------------------------------------------------------------------------------------------------------------------------------------------------------------------------------------------------------------------------------------------------------------------------  
-----------------------------------------------------------------------------------------------------------------------------------------------------------------------------------------------------------------------------------------------------------------------------------------------------------------------------------------------------------------------------------------------------------------------------------------------------------------------------------------------------------------------------------------------------------------------------------------------------------------------------------------------------------------------------------------------------------------------------------------------------------------------------------------------------------------------------------------------------------------------------------------------------------------------------------------------------------------------------------------------------------------------------------------------------------------------------------------------------------------------------------------------------------------------------------------------------------------------------------------------------------------------------------EMDEDERQRKLQAGRAKLASFQQKRAKGDIAGASKKTQKRKGQAVTQIHS-------TTQDCPVKSDSCQAGDPNLSKTSHEEVERKGREEEREEKEMQLQELQTQFSSIQTPPEQS-HSSVIQALQLKLEEARTTSLTTSALLDERTKELNEALKALETTHAQLQKVQTEENLTNLEDVQTQALLE-----KEDHKKKEAKMDEQIKKLEQVMEEELEQFEHLLKTKDDEE--------------LKTIELEALRQSLSAMSELEQAQVNFQQKE-----LESTKVSVFQTNTTLTVARITLSQTQSELQESQERL---DGDLEDLVSSHKTVLQQKGQQALHLEEKAKELQKEVLRLQEEKKLLKQNSEEEVGQLWTQLESMRASRQELGELKEQLLARSSRVDDLERLKAEFNNQKREIKEQNEAELESLRRYFEQRLRVTEENYREDIALLQLRLLEEKHQLELANLQSSLSISFKEELEQVRS--------KLTEHYYDELQEMKSRHALELEQLKAKLSERHLQVQLNQEFPEELIRFEETLKQERRHVQDEMKRLK--EELQEKHKAELSCLKAELEKEMTKETTDLKNTLMQEKEKLQSLQTALENDEMLPK-------SK-IPKMFKEEK-----KELEQRLTQKCEICLVELNKKHQAELENERVTLVNKHKEEITKLHDKHNAQLESLGASHRAELAAMVGKLESKHNTELVAVEAALN------------SKRKKTNQAQLEALEAELAHKHQEEKDELEKRMLANMDIVEATYLKEVQALRDEMVQLENKHQEELSRQKSEY----------------------RQLIECHTAEQLSFKEALKK-----EQAQVHMEKFSAMAAELSHLHKRELSQSYNSEKEQLVKQHQLQLQELRSVSARELEACRRELEEESSRQRQHFLEEAELLKVQSEERLQDRINLLKQTEIGCLQEELK--------------GKRAQMETLD---TLLQRRERESQEGGNLLKMLTDDLQSVKQENDILRHNLDRANEKFKKVLVEMIRGTI--AIEELIGRKLNAGAKLTEES--QRNLTGNKDLQES-----------------------GMSAGDFSSEDPELTHMLCESLLVSDIQISPDGEEAAQNACARLRQT--VETLLDLLNQANAQLEQTHRVHLSLEEKFSKAKEDSAHLLEQHKIILEQLNQEAKQKSELQVELHKAEEGLLDGYVAEKAN-------------LDEMLQQKEAQEEGLVEELEDLKVKMHQMQALSA----ELDSLRSKHQELAEEHAALLRQ---------------------------------------------------KDHLSAGLGEREKALLEETERLVQDKLDLQRQAEK--DHSSFMQRLKAMERDLEEQETQGLEMEQHHKSQTEDLNQRVQALEKQLKHDRQFIEDQAVEREHERDEFQQEIIRLEAQLRQPNNVDNKGHR-------------------------------------------------EKNQEIDHLTNEIQRL-EQELEN-TRDAKVGLQVKIDFRDNALVELETINRQLKAENQ-----------------------------------NLQEELATQEEELA-CQKR----------------------------------------------------------------------------------------------------------------------------------------------------------------------------------------------------------------------------------------------------------------------------------------------------------------------------------------------------------------------------------------------------------------------------------------------------------------------------------------------------------------------------------------------------------------------------------------------------------------------------------------------------------------------------------------------------------------------------------------------------------------------------------------------------------------------------------------------------------------------------------------------------------------------------------------------------------------------------------------------------------------------------------------------------------------------------------------------------------------------------------------------------------------------------------------------------------------------------------------------------------------------------------------------------------------------------------------------------------------------------------------------------------------------------------------------------------------------------------------------------------------------------------------------------------------------------------------------------------------------------------------------------------------------------------------------------------------------------------  
----------------------------------------------------------------------------------------------MEDEERQKKLEAGKAK------------LAEYRQRKAYADS-------------EDPEGDSQERVEVC----VGGEESSGAGQDGSQEENKEPPSSEFTFTRVLQSGETVKHRQNYTIEPESEVSTTAEDCSSE------------------------QDEVEPLQQMKKAGTIQAMEVELAAKTKVVEELTLELEEIRAAFGTEGVQQLQDFEAALKQRDGIITQLT-ANLQQAREEKDETMKE--FLELTEQSQMLQIQFQQLQAGETLRNTSHSSTAADLFQAR-QQLSLYQQQVEELNAELKKHHESSREQLENIEQLQNKLKEAEVKSGRKTEEFLTQSINEKDRLIAEQEKVILSQEQSLTRLGLTEESCAQALKEKNQE--EERTEFETCK---AELATSRQKERMSSNEIMQLMGTIEDLQKRVHQGNLSESDAIQKMQEDTVRKLEVLRAELDEMYGQQIVQMKQELHLQHAARVEQMTTQHRAELELLKENHLSQISAATAVEMD--TMNAKIQELQFLLDESHAKHNKTRHELSQVSEEKINLQVKVEALLQDLDSAKEKVEQVSKSVISQESHHIELHRLQ-EVIENLKGELALAREDAQEMEAKHDSEITNYKIKLEM--LEREKDAVLDRMAESQEAELERLRTQLLFSHEEELTGLREELQRENFLNTENLLNEAAVRHERVTDELRAGYEKKLHLSENEKASCVLEREELLHQILELK--EDLKIALHSSKADELVQQLQELQVELEELRKRGAKQAQMENEMQQLAKKTEVLESKSRDQEKCWENRWREQEEEKRRLMESNNSLEE------------------------ELAAKRLMIETLEADNIQIHQQVAELAEEIKKQRTTFSFAEKNFEVNYQELKEEYTCLIEAKAQLEERALKETLEFEAKIARLQSQLQELEESSRDTETEG-------------------------KHTTELMEKLNVTLREKESLVGKISEATEQLMLSQSRVKE-LEEQRLRAREENEGVTTLNESLAKMLE-----EKQMESQVMSHSQERDGEADST-----------RGCQGDGDSISKPF-VKRLSEQQHEMGC--------------------------DMEEEETTTEHVSITHPALVRRPLQKNCKTCKLFSEEII-VELGTLSFSQGECRLQMEAQRISLSQIHAAQLELLQEETEARTHSLELR-LQQKSSQEDPAEPKLLQSINISQAVQKECADIIED-FR---KIFGEKLLESIEVEGWKPSPSKRKDTTGSTSVLMEARALYK-DLQQVRETIEQEHLRLSQLQSL-----LKDDVNKVKTGHKSRIKKNEKQTVAVVVQLKKKTSAKHVKEQSGTPPACMSEELQRLRAEEQEKQLKLEESHREEVERLRAHYQQQATETEERYLTELLMLQQQLQDVTGPHRSRSRTVDY-VVV---RGILAESN---VRVSLSSWKMVLPG--CSGENIASVGQKVQGKKQDWERIEEEVAKAIVQMSVQFAQQTELLRLNKGACQKSTSMQTMQSGEDE--------MQE---EELTLRASPSLSSRLEEV--ERLKRELEERNVVISMLKEELLKIKSGPEQVCG--LCEPV-TARVG-FKGYRVRV--LVA----QTEIFICIVQRK--AKNTQKHKKATAVVLYLHQRVALFARPDRHCVSGAPVAMAA-----EAHCSSGLQWELASAS-YDFKMTGFLSVFSLLPIQNQKQRENGQAEDS-------DERDSQGLERKQDNEGKVTFHDISGSTDRNCFSHLETSLLKANKNLSHVLVEVLKTTAAAEETLGLHMQSR-SPSS--APTSHIIFKRKSADLNLLRSPPLTVPEY-----STECYESS---ETTTEDADLCSMEMKADESLKMSHQMIESLLLGVGSQLENEDYLVGISCRLQTALEKMLMVITDTS----------------------NQLEHARLTQTELMRESFRHNEEINGLLQKQEELQERLTEEARAREQLALELHRAEGLIDGYTGERAALEEQLHQKEEFRLNLEQELQVTSSRLHELEQERLQMQEERELLSRQQD---AMREHAGPRELHLL-------------EETEKLMKEKV---EVQRQAEKENADLLKHNKLLEAELEEQVSRVFELEQAQMTERRDLQQQIQALEKQLE--NNRKFLDEQAVDR--EHERDFFQQEIQKLEQQLKNQQKQQAGSEHRNQEVERLTSQLKEK-ADWCSELLLSS--E-QLRREVQERDEEIDKLESRIRELEQALLA--SAESLEE-VKQKNQHATIAEAEQSTLES--QLQTEREALERKEKEICNLEEQLEQFREELENKSEEVQ-QLHMQLEIQRKE-------------ISSQQDYLENRDSLLQVDKD-AVTGAWHSILSLF-VASMAKIHSCPMCNVMEAKDREIALLNEQIIKLQHKET------------------------------------TSDNKELDGREEVIKELESQVECLRSEQERLKRNNEE--EVDQMSAVIDKLQQEMTNIEQK------RVTEEDEDTRPELESSTWAL--NKEEYAEMKQRMDLATEELRTLKTEHSKLLESYLCLKESAKALAET------EQQRSPDTELEDALLQKTA------GLVVMQAQVQALEQSASSRLEELGLRVQEL-EDTVNEKECELRHCRLQLEQTERRAEGLQQKASELEVNLRDKVTEAFTIQQQPEGSKETAER-------------PGHYDFDI-VIPKMD-SSDIGRLRPVSTGKVFHLTQRLQELEVGLSGIQKDQELQKQLLSSSEEEVLEYEKRLSVLMDLLG-QMKN-RTHQR---------ASPA----------------------------------------------------LEDLHLRDLSVSELQQELQKLRENLNTTEEEFSIYKEH------------QG------RTSNQASGKSHSTRPSSRSEQPTVAVAHSSTQTEHVHLS-DLRPE--AKTHTSDEMEEVIAEFQEKIVQMQELHAAEILDMEARHISESDSLRRDT-QALEEECKALQAVIDKLRSPEARKLLPKLKLKASCSCGLCDSVADSSSDYSQRTGFDIPSLQQELRTTPEGARR-DPD-DPLPDRIKTLLREVHQEGMQVLS-LSELP-ISEGE-------SGR---IQGWVKERDDLLTTVESLKGLVAHMQMK--------VATSA-SENWRAQLLDAVRRVFSSERNVLKSALYRQLDALDTSDAIIHLNQLERRLAEQDAQHREAMDSLHIADRASLTSEIHQLRSQLEHAH--RGESAGYSLIERRGGGGGMKPDDKIRKIYSNVVGGGGGGEEQVVESDRLLMEELKGELSQTKLELETTLKAQHKHLK--ELDTLSCWGNRAEVSQKAAEVDALNDRLTEEK-----KRSRDLQWAMEKERCESGRSEESKREELEDLQLSLEEQEGRVAQLAATLEEERQASARLSQRAE----EERLG--LHR-RLQELQVRLE---------------TEQAKALEMSAALGRERELR-----RSGLSSELEEDEAGQEVDG--RLLDKLQR-----EL-DDKQAQVVNLLSQLEVQRLGAVQKEEELSTGRQKLRREQEALRVARAQLEALEAQMSESQEQLGRELEKSRSLEEEREQ------------------LEEKLCWLRE---QSGRREVVGGDRGMVELRKDAVWPGGSTE---RTKDWVFQQKSAS----AQSPS------------FGAADAVLSKLYSISSKVGSMAAPPAGSSAAA-VQPAELSWVQSSLEEAISVLQQAAGPPSVPESLSLLAG-GSSS----SLTQRLLRQNAELTGFVSRLTEEKNHLRNQTLRLEEEIRRHRQAGPGTGDGFG--KGIAKSDSSGTLLSQE-REAWAREKVRLERALHQAQA-------------------QVARLRGEIR-SDTLREITGPQ--------ADNAALKRIYGKY-L-RSESFRKALVY-QKKYLLLLL-GGFQECEEATLSLLTRMG--------GQPALSS-LGPLSQRRRGL---------------------------------------------------VRFRSAVRVSIAL--SRMRFLVKRWQKTMGTSA------------------TTSGAASKPQAGQTAGTDARD-SPYLRPGSLETYRD-G-GGGVPGGRGRSGRESPRSAASSALHRFHLAGEHGALTCSHLQS-------------YDPDRALSDYISRLEALQRRLGSVTSGAA--SYAQLHFGLRR-------------------------------------------------------------------------------------------------------------------  
----------------------------------------------------------------------------------------------MEDEERQKKLEAGKAKGEALNLISSSFQLAEYRQRKAYADSQKKQKKKKKKKSAEDSEGDSQERGEVEPQHGVGGEESSGGGRDGSQEGNKDAPSSEFTFTRMLQSGETVEHHQNYSVEAESEVSTTAEDCSSEVNGCLDELKSVKRKINGEVRGI-QEDEVEPLQQLKKAGAIQDMEKALAEKTKVVEELTEELEDIRATFGTEGVQQLQDFEAALKQRDGIITQLT-ANLQQAREEKDEIMKE--FLELTEQSQKLQIQFQQLQAGETLRNTSHSSTAADLFQAR-QQLSLYQQQVDEMNAELDKHQESSSEQLENIKQLQNKLTETETDAGRKAEEFFRQRINEKDRLIAEQEKVILARECSITGLEDELERSKKCLHELQQQMAAKETHLGKCLLELAELAASKQKERMSSNEIMQLMGTIEDLQKRVHQGNLSESDAIQKMQEDTGRKLEVLRAELDEMYGQQIVQMKQELNLQHAARVEQMATQHRAELELLKESQRSQTSAATAVEVD--VLNVKIQELQFLLEESQAICHETKHELIQVTEEKTNLQVKVEALLQ--DSAKAKVEQVSNSVATQESHHTELRRLQ-EIIENLKEELALAREEAQEMEAKHDSEITNYKIKLEM--LEREKDAVLDRMAESQEAELDRLRTQLLFSHEEELSSLREALQRENFLNTENLLNEAAVKHERTTDELRVGYEEKLHLLEAEKAGCVMERDELLHQILELK--EDLKIALHSSKADELVQQLQELQVELVELRKRGAKQTQMESEVQQLVKKTEMLEIKSREKEESWENKWREQEVEKGTLTESNHNLKE------------------------ELATKNQTIETLEAENIQIHQQVAELTEEIKKQRTTFSFAERNFEVNYQELKEEYTCLIEAKTQLEERSLKETLEFEAKIARLQSQIQELEECG--TKTEE-------------------------KHTTELMEKLSVTLNEKESLVGRISEVTEQLMLTESRVKE-LEEQLLRAREETAAAITQNESLGKTLEGTQEIKKQGAAEPLCSAEEHHLQIQSLQEEIKALQSLLQATESERDDIQQALELQRLALTPSPVAA--------------------------AAPTEEGPVEGRSSPRKSTATGSNRR--KRRQRSKQERR-LATAVSDCREKQKTWEEEVLCRDLRKIHAAQFELLQEETEALTHSLELK-LQQQSGRGDPAGPKLLQSINVSQVVQKECTEIIKS-FQ---KIFGEKLLESIEVEEWKP-PLKREDTSDSPSVLMEARELYG-DLQQLKETIEQEHFRLSQLQSL-----LRDDGSKIIELQTACPCFAAERTTVVFLSPDLSIFFLDVEEQTVTPSACISEELQRLRAEEQVKQLQLEESHREEVECLRAHYQQQATETEERYLTELLMLQQQLQDVTR-AQAHSRLFYYKIIV---SHLNALKNSKKQKICFNIDKATESGREWSEAHSEELQKNVHGKKQDWERTEEEVAKAIVQMSVQFAQQTELVRLNKRSCQRSSSMQTVQSGPEQ--------MEETGNEEPTPRASPSLSSWLEEV--ERLKTELEERNIVIGTLKKELQKIQSGPEQVCASHMCVLLNIIRLQLFSGSLVRM--QIIHELRRTVIGASVSQEKWFLKTSTLDSRMHFIDFKLVQFKDFTVLRTSQCNTTASDKHNKNTYWWQKHIHALLDSPAVSLSIYDFEMTGLLSVFSLLPIQNLKKRENGQEEDL-------EEGESERLERTQEDGGKLR-RDPGEAPDR-VASFFRNILLKTNENLSQVLLEVLKTTAAAEETLSLHMQSL-AASSGSTPPSEPLRPSSSEQFIWLCSFQDHSNTL-----LLSVHPSSPSIEPSTEDADSWSREMEADKSLKMSHQTIDSLLLGVGTRLDSEDYLMGISSRLQTALEKMLMVITDTT----------------------NQLEHARLTQTELMRESFRHNQEMNELLQKQEELQERLTEEARAREQLALELHRAEDLIDGYTGERAALEEQLHQKEELHLSLEQELQVTSSRLHELEQERLQMQEERELLSRQQD---AMREHAGPRELHLL-------------EETEKLMKEKV---EVQRQAEKENTDLLKHNKLLEAELEEQVSRVFELEQTQMMERRDLQQQIQALEKQLE--KNRKFLDEQAVDR--EHERDFFQQEIQKLEQQLKAQQKQQPGSEHRNQEVDRLTSQLKEK-ADWCSELLLSS--E-QLRRELQERDEEIDKLESRIRELEQALLT--SAESLEK-VEQKNQHATITETKQTTLES--QLQTEREALERKEKEICNLEEQLEQFREELENKSEEVQ-QLQMQLEIQRKE-------------ISSQQEYLETRDSLLQVNSR-ART----RTLSWI-VSTVS---ECVISEVMEAKDREITLLNEQITKLQHMET------------------------------------TSDNKELDDREELIKELESRVECLRGEQERLKRNKEE--EVDQLNAVIDKLQQELSNIEQK------QVSEEDEDTGAEQESAAWAL--DKEEYDQMKQSMDLATKELTKLKTEHTRLLKNYLCLKESAKALAET------EQPQSPDPELEDALIQKTA------GLVVMQAQVQALEQSATSRLEELGLRVQEL-EEALNEKEGELQHCRLQLEQAEGHAEDWQQKASELEVKLGDKGDEVNEGSQEMEARLGHVEP----RLMANVD--PHRYDFG---IPKMD-FREIGQLRQASTGKVFHLTQRLQELEVGLSGIQKDQELQKQLLSSSEEEVLEYERRLSVLMDLLR-QMKS-RTHQR---------ASAAAEVRVSTSNTKFEYCRSHLEASGCFDELQEVRAEVLVTKEELVSYKDTCGRLTEELQVDESRVSELRGELTEAQSEAAAAKEEITLLKEREMTIAHLSGELCQDKPTLSRKTSNQTSGKSHSTRLNSRSEQQSVAVTHSSTQTEPLRLS-DLGPD--SKTHTNDEMEEVLGEFREKIVQMQELHAAEILDMEARHISESESLRRDT-QALEEECKALRAAIDKLRCPELR---PG---NVASQFNFS-TVTDSSSDYSQRTGFDIPSLQQELRTTPEGARR-DPD-DPLPDRIKTLLREVHQEGMQVLS-LSELP-ISEGE-------SGH---VQGWVKERDDLLTTVESLRGLITQMQINRVPSL--LLQTSG-NEDWRVQLLDAVRRVFLSERNVLRSALYSQLDTLDTSDAIIHLNRLEQRLAEQDAQHREAMDSLHLADRSSLMSEIHQLRGQLERVH--HGESTGHGLIEKREGGGG----------------------DRAAESDRLLLEELKGELSQTKLELETTLKAQHKHLK--EMDALRLVA-RAEVSQKAAEVDALTDRLTEEK-----KRSRDLQWAAEKERCETDRKEESKREELEDLQLTLEDQKDRVAQLTVTLEQERQASARLSEQAE----RERLS--LHR-RLQELQVQLE---------------TEKAKALEMSTALGRERELRTGVSSRSGPSSELGEDTAGQEVDG--SLLEKLQR-----EL-DDKHAQVVHLLSQLEVQKLEVVQKEEELTAGRRELRREQEALQVARAQLETLEAQISESQEQLGRELEKSKDLEEERER------------------LEGKVHWFIE---QSGAREP---HQTVFLFKKDDVSLGDSTD---RTKDWVFQQKSGN----IQSPSASPAA----CGPWRTVDKILSKLYLISSRVSSMASKPTDRPTAE-VEHEDLSWVQTNVEEVITMLQQCPGLPAVPE-----PG-GSSS----TLTQRLLRQNAELTGFVSRLTEEKNDLRNQTLRLEEEIRRYRQAGPATGEGFNFRKGVSKSDSSGMLLSQE-REAWTREKVRLEKALHQAQA-------------------QVARLRGEIR-SDTLREMTGPE--------ADNAALKRIYGKY-L-RSESFRKALIY-QKKYLLLLL-GGFQECEEATLSLLSRMG--------GQPALST-LESIHQRRRGL---------------------------------------------------TRFRSAVRVSIAL--SRMRFLVKRWHKTTGISS------------------ATSGTVSKTGAGQTPGTDARD-SPYLHPGSVETYRERG-GGGVSGGRGRSGRESPRSVASSTHHRFHLAGEHGGLTCSHLQS-------------YDPDRALTDYISRLEALQRRLGSVTSGAT--SYAQLHFGLRR-------------------------------------------------------------------------------------------------------------------  
----------------------------------------------------------------------------------------------------------------------------------------------------------------------------------------------------------------------------------------------------------------------------------------------------------------------------------------------------------------------------------------------------------------------------------------------------------------------------------------------------------------------------------------------------------------------------------------------------------------------------------------------------------------------------------------------------------------------------------------------------------------------------------------------------------------------------------------------------------------------------------------------------------------------------------------------------------------------------------------------------------------------------------------------------------------------------------------------------------------------------------------------------------------------------------------------------------------------------------------------------------------------------------REMDEDERQRKLQAGRAKLASFQQKRAKGDVAGASKKTQKRKGQAVKTSPSLESANVETTAELSVESVDSETGALLQSLRTQLEEERQRSKCAYADLAVEIEKHQHVLSLLEEE-RKG-REEEREEREMQLQELQTQLSSVQNQCLELQQDKAEKEKLNREVLELKERLQMKENAESTTALQFQALEE-----EMEMLKEEHRKEVEKMKLHQ--EEQLKEKEEEMKTLLEKQ--------------VSSVKEDMDRLELSLQLEIKDLTMKHQQEVGELNAKHSEDLSEMKAELRESLEAAHLAELQ-QLQSFVKCF---SGELEDLVSSHKTALLQKEQQAIELEDKGRQLQTEVLRLQEEKKLLKQSSEEEVGQLWTQLESMRANRQELGELKEQLLARSSRVEDLELLKADFNVQKREIKEQNEAELESLRRYFEQRLRVSEENYREEIALLQLRLVESALEESVLKISDD-SLQLEEKHALELA--------NLQSSLTLSFKEELEQEALKEERQEE---MKRLTEELQQKHQAELSVLKAELEKE---MKTKTTDLQ--FTLEKEKGPEVVIIKQRLEAQYDGELKKAKTCMATEIKELNALLLEQHQEQLCQA-------QKRCDNSFQEEK-----TELEQSLAHKYEMCLVELNKTHQTELEHQRVTLVNKHKEELTTLHAKHNAQLESLGASHRAELAALVVKLESKHNTELVAVEAALS------------SKRK-TTKAQLEAAEAELAHKHQEEKDELEKRMLANMDIVEATYLKEVQALRDEMVQLKNKYQEELGRQKSEH----------------------RQIIERHTAEHLSLKEALRK-----ELAQVHMEKFSAMAAELSCVHKKSIILALK--KKKHVVFVSGAETEFEEQKQVEIENLRRSFTSEQEETEQSYTVKMSHLTAQLQQ--LDAVVSQLRSEIGCLQEELK--------------GKRAEMETLD---TLLQRRERESQEGGNLLKMLTDDLQSVKQE----KHKLDQANEKFRKVLIEMIRSTI--ATEELIGHKLNARAELCEES--QGSSTGNKDSPDSGLFHSSFKDVF-----------LGILAAGSSSEDPELTHLLCESLLVSDTQISPEGEEAAQSACVRLRQT--VETLLDLLNQANTQLEQTHQVHLSLEEKFSKAKEDSAKLLEQHKIILEQLDQEAKLKNELQLELHKAEG-LLDGYVAEKAI-------------LEEMLQQKETQEESLVEELEDLKMKMHQMQGLTA----ELDSLRSKHQQLAEEHAALLRQ---------------------------------------------------KDHLSAGLGEREKALLRETERLVEDKLDLQRQSEK--DQSSLMQRLRALERDLEEQETKGLEMEHHHKSHTEDLNQRVQALEKQLKHDRQFIEDQAVEREHERDEFQQEITRLEAQLRQPINVDNKGHRFEDLVLQLRIQVESLQAIIKDKTDDYASL--------LAANQQAQRDLAERNDEIDKLAGRIREL-EQALLNSTDNNRLASQLEQELHRVKLREQELTQDKQALEQQQLSYRLQISALQSKLDETKHC----YNDHARDPTQDLRNALDTAQQNLC-SKEQEIVHRHTKAELEDLRSQVEHLKSEVSRVRENKQEEEERLHEVISTLQAELATLGPNLHEVSDSQDGDSINPSLAPSPEPHHCTIQEQSRLKALQTQLETAVAEKESLEQLLFKQEEDYRGHGEEFGKRLQAERDKVEELQVLLSLKETEVAEAKGLIEEEREKRKQSEKERDGCKSQEEEVDTLHEKNSHLSSLILELQKNEQALRTKEQDTKIEMEVLREANITLERQVQEIRGPDSSA--PREGNQSKPGSERCAGECCSERITQQRGSNKSFLSSQTHAESKDRALVDLETINRQLKAESQSLQEELAAREEELLCHKRELEQLRQHRHQQEKIHHQQDYQQKVNMMGKVIYMTTKGFENVLS-VSREEASLSSPEVLRRLESSEDRIPECIHASVLGSRLPEISA---LNSPGLDLPHAKTSPRVVMETPCSRTITPVPDPQSTNSPGSVSLSDNFSVLDSLDTDRVCDLYTYYPSSTSSLSAPEWAMTEKMGLKISNKNLWSTVRLFFFAFFPPSDVSSELGARLRVELEQTERLDAQFVEYL--RCRGINPAVNTDSAAGSMSYSDDLLSPELQVDRFVSLLFRQGLLKKVYQESCRILTLSQRRVIAQPPFSDLKVVSLGQMEHQDSEDASASLDHRDKSSTKRPMSWQQEKRALQETVIALRELLCRMAQRYSEVSLGILSSWEIELNCIFVTDGGDGWQRDELSRQIESQLRTEMEESQKQLKCAQDTQQEQKNQIQSLRTVVEEG---------------EDSLRREQNRVQELQQQLEQERAVSLRKEKAEEERRGAWHTRSEQQRSELMALK-----GQVEQEKVACSNLRQELQIEQSRSVFLEKRLEDIQKELENVHQRYSHQQVVNAQEKAHLEHLLAEAESRLADIHAKLRDAHGKLDELRDQCS---RQVDEVTRKQEKDATRDRNFISDLRAQLEQERRRGEELTTEAEIMQS---KRKLEEQEKAWSKKLKREQEAAVTHHVALEVLKEQKQEVSHALDVEQEQSRQQRAELAELKERLQL-LKEKEKEREEQWERERTKERQEQIERERRQQRMNNKLL-RCSGTKADSHDAFCFQCELELLRQQDQKRMQTLQCTLAELQKEEREMAAQRLCVQTHTDGTQHKTQQTPSPKLVERLVT-ENSDLTERVTSLSQERATFKHRLAYLERQLRRAESALTKVTTETENRPVCDGASNTKLLKPKMPIREVNMISFQVQRLYERY-L-RAESFRKALVY-QKRYLLLLL-GGFQECEQATLCLIATMG--------VRPS-----PLLSSQRRPR---------------------------------------------------VRFRAAVRVVIAV--SRMKFLTRKWHKAIR-RF------------------SSSAAVNGHTPVLRQQQPRMNSDFPTNRDSIVDLKETVL-------VHVPPTKSPFKLHNRSYCSLASGPSAGAS--------------------HDPEHSLTDYIHHLEKVQQRLMGARQGECQKSCRK--------------------------------------------------------------------------------------------------------------------------  
------------------------------------------------------------------------------------------------------------------------------------------------------------------------------------------------------------------------------------------------------------------------------------------------------------------------------------------------------------------------------------------------------------------------------------------------------------------------------------------------------------------------------------------------------------------------------------------------------------------------------------------------------------------------------------------------------------------------------------------------------------------------------------------------------------------------------------------------------------------------------------------------------HNLELEALRLSLTNLHTAQLELSQSNMQKDKDVALSELQTMLREKWA------------------------QESAMLQTRQQFELERIREQNREQEERNQRVHQQEIGESHNSESILKPSASIIIGNLNQKWETRLVEMKTSVEQLQSCKLEDLKAEYLLESEKIKTEFQSELN-------------NVRHKLSETETALTQAEAALSDTQGKLEELQRSSDHDLKNLEKELKQALM-DRDAAAQVDRLQSEKEELKTSSEQEISNLWSQLESMRTSRQELGELKEQLLARSSRVDDIERLKQEFTQQRQEIKEQNEVELENLRTYFEQRL--------------------------RVTEESHREEIALLQLRLVEGALEDSVLKTGDASISIHLGLHDLEGNCEDDNSDAMAEITRQLEKHKEELDSLRLQLEERHQQELEQLRSSMTLAYKEDLLQARTELTDRYYKDIDQLNTKHAHE-LEQLRAKLSDNHIKEINKIRLQSAQEAARQAEAEVNERTKDYQARVTQLESRLSQLSKMHSEELEKAAS-----QHREQLRQMEETVRSFFLLKHQKDVAEKLETQKAERNRASEEFTEKQDRLREQAKNQLASQREELQKSALEERGKLEQAFLEVQKQLDMESEKLGALQKSLECQDIGSVRQLIQHLMMDSSLKVT---TLKNKIKKQNCKEKCFHVCKRVYLLFVCLTHRHQEEQKQLEDNLSLQRDAALDQQREQHAIELQTQKTLLEQRANHKTALETLEMELTNKHKVE--------LDKLEAVLQET-NLAQLEAQEAELQARHKREKEELEERLLANMDTLESTYLKEIQLVQDEKRGELRDLQEHYSNEIERVRKEEQEFRGVSVRELEALRRELEEEASRQRLHFLEEAELLKTLYNFVFGTRIEESDQEKAVALEELEKTLRQQHEQTELTYSDKMSQLTAQLQQLDTVVSQLRAEVSGLQGELEGKGAE-------METLESLLHRREREKQEGDNLMAMLRTDLSTATQQRQDLQTARDKLQRIVKEMLRITITTEEHISHKLGSCVSGGQS-----------EEKTPSS------MTARN-----SRDSHETAELFNN-LTEDKIASLAVDEVSELSQRLCESVFSGPEGELEAEGEELMLGACTRLRTSVDKLLELLSESS----------------------QQLEQMCSLQAVLNERFRDGGEAGASLLFQNSQLLEQLDHEASLKSQLQLELHKAEGLIEGYVAEKATLEEVLQQKETQEQRLAEELESARMQLKQIGENHTLLLRQRE----------ALTADLGDTEKALL-------------AEAERLGQECV---EVQRQAEKDRGGLASRLHMLEQALEEQESRAHQLEEQHRLQTEDLQQHIDALEKQLK--HNRQFIDEQAVER--EHERDEFQQEIKNLEAQLKHPSKGHASVDSKGQRVRFNAENVESLQALIKDKMEDYSVLLLAKEQCQRDVEERNDEIEKLATRIRELEQA--LLSCAEASRTVTQLEQELQKGHKNFQELTQDNEALQQQLYANKLQISALQSKLDETRHRFP-DVTPDP-NLLEQLETTQQD-------------LLAKEQEVELLSERASELEKDLVVREEEVRQLTLQLELTTRDSRAAEEQLHAHITHL--------------------------------------------------------QILEEKQAEIEELRSLVERLQCDQERLQQAKEE--EMEQLHEVINKLQEEIS--QLD------PNHHEISDPNTDSPESSDFPWSPRPRQQRASEELQTELEQATGEKDSLQ----RLLHSQEEQYGGQVEALGRSLGEERG----KVAVLEQEVK------KLTLQLEEKRTEAERLAACVEELEDQERSL-QSCLRESELHLRMVEERRDEFQEEVKKQRAEKELLENQISELQHREQENQGELEDLRSRLEE----LEEHVQA--DMVN----LSALETS-KCELSMERNALRKREGRLQEEIERLRQEMTSKTNYIKELHEQQEAAVAKQGEAQKEVLTCAEETLAKAETALRER-----EQQLILLRAEHEALRAELAAVKEGLSNSTERGDLPVMLNVVASLFVKYTAMNLLVIFSTAQMITLSLWKKSNLVLLNFLPSDERSCIGVFPRTRPCLPHCLLLKSCGGWTAAKSARPACRYLTSQSSARSTTPAWSSTASLPPWRGTKREHTSDKLSAQPQRKL--HPARTLPPHALLPCLNTSLCSSRWMQKSKWYIFPWHTVRMVDDLDNSDV-TPSRSPIGSTSPVSVPEWVSDGYGSSTDVSSELEAMLKLELENTERLDAHFVEYLRCRGMSPAEN-TDSAAGSM-HDSHEALTPELQAMLKRVYQESCRILSLSHRPAPSGQSRPDS---DPAPPPSWQRERRALQETILSLRELLCRMADREPKTMEVREFYINIFC-PSDGISEIFSGNPESKFVCLNLIFSKVVCLLLKADDGDVDWRRELLQAVRSVFDSEREWLHTQLQAFMATSTQMDLTPLLDQLKKLF--QKQEEQQWKSLEQIFNADRHSLLAEVRSLHAQLHASTQQSQQQLRQLQDSLHTTKEQGMETQHQLHSDVKELEMRLQQEQTQSHDLRCSLEAEKSRGLEQQKQLEAELKAV-----SVLKAELEEMRTELQSSYKHQEELKSEIHKLRLSLENFEAERQTCLQAVEKEQAKVQLLQEELKQQQLHSQQTTVQNKQTQESLHQALN---------------EQASQVSQLNSALDQERITSSNLRSELQIEQSRCEALLAQERERTELALSRLEE-----ER-SRSTELSNSLSRQ---AQEHARRLEEEARRQESASAHDRKFIQDLRAQLEHERSQGEDLAAMLERLQGQVLEGKRRQAEAAEQEAHKEQEEIRRLRAALEGLQNQKTEVGRTLEAERGRAAQLQVELDAMKEKMRAVRV---KEEQMERQRKQEQAEKERRQEHANEKLLE-LELLHQKDQQRLKQLQQTLTDLEREEKRLTSERLQR-ETHSNALNKHTNISQQLYYYYYYYFTSPLINMLWQFTN-VNVRLEGQAEEIIKDSSTPSLIERLMKENSEFSECLAALSEEKISLKHTISCLERDLQSLKRMEQVKSSAVTEECMVSE-KLAWQKEKATLQSALRKAEA-------------------ELSKATASNENRPINDLS--------------NSKVQRLYERY-L-RAESYRKSLVY-QKRYLLLLL-GRFQECEQATLALIARMG--------ALPTLSQ-----SRTSRPL---------------------------------------------------NRFRTAVRVVIAI--SRLKFLNRKWQRATR-KI------------------SAGTATAPAL--------RTEVLRPQQ-GGVAFNSPPTRDHAFTQRGVVSSVVPPIKSPFRLNHRMYTSPVLGAAERSFSST-------------QDQERSLTEYIQHLETVQQRLGAACPGSPSVLPNARKSAR---------------------------------------------------------------------------------------------------------------------  
------------------------------------------------------------------------------------------------------------------------------------------------------------------------------------------------------------------------------------------------------------------------------------------------------------------------------------------------------------------------------------------------------------------------------------------------------------------------------------------------------------------------------------------------------------------------------------------------------------------------------------------------------------------------------------------------------------------------------------------------------------------------------------------------------------------------------------------------------------------------------------------------------------------------------------------------------------------------------------------------------------------------------------------------------------------------------------------------------------------------------------------------------------------------------------------------------------------------------------------------------------------------------------------------------------------------------------------------------------------------------------------------------------------------------------------------------------------------------------------------------------------------------------------------------------------------------------------------------------------------------------------------------------------------------------------------------------------------------------------------------------------------------------------------------------------------------------------------------------------------------------------------------------------------------------------------------------------------------------------------------------------------------------------------------------------------------------------------------------------------------------------------------------------------------------------------------------------------------------------------------------------------------------------------------------------------------------------------------------------------------------------------------------------------------------------------------------------------------------------------------------------------------------------------------------------------------------------------------------------------------------------------------------------------------------------------------------------------------------------------------------------------------------------------------------------------------------------------------------------------------------------------------------------------------------------------------------------------------------------------------------------------------------------------------------------------------------------------------------------------------------------------------------------------------------------------------------------------------------------MDLREHAEIDLQE-HAGMD--VREHSGMDLQEHICGLQQKIQQMQELHAAEIMDMETRHISESESLRREN-QQLEDECRALRDAIHTLSHTQGPAVRSERPAVSPFKDGYTSANQN---QWSQRTGLDHTLLQQEMLITPEGARR-DAEPDVLPDRIKNLLREVHQEGMQVLS-LSELP-LGEVERET---HTRAHTHTWSAERERDAFCSAVETLKSLISRLQXVRERELLKVCVHHD-EHFSVCPCNKILTSLLEERLLSSDRLEERLLSSDRPEERLLSSDRLEERLLSSDRLEERLLSSDRLEERLLSSDRPEERLLSSDRLE--ERLLSSDRPEERLLSSDRPEERLLSSDRLEERLLSSDRPEERLLSSD--RLEEMKSELNHTKLELESALNTQHTHLR--ELDTL-----RSEVSLRVSEVDTLTDRLAEEQ-----KRGRELQWALEKQKHRLDRKEEADREEVEELRLALEEQRSRVSELSISLEQQKQISDQQREIQHSDVLLQILE--HYI-YCVTVTAQLD---------------AQRARCAELSGALEKEKQLNTQLIQRFQSGSST------HTLPSVESLLQTLQS-----QL-MEKQTQVVQMMEEMEKQQLEALQHRRQSQEERSALAR---TAAEHQSALQTARESLREMQSQLQAERENGRRMETEREK------------------LQETLTLLEDRLHSHTARNHGTLTITALYNTRSKTLDGQPVD---GTRDWVLQHEPANTLNLESTCVQQTSADP-KHMEKSSDPKHMD---HILSRLQLIAAKINSLTSQS-PHSKAFSWLHSNVQDVMSLLQHIPSAP------SALPE-GSCS----LLNERLLRQNAELTGFVSRLTEEKNELRNQILKLEDELRRYRQHR---DNTHTAWSRSAVGRQDLLLSSSE-RESWAQERNSLQKSLRQTEA-------------------ELNRVRSELR-CDTLRDLSSAD--------PDNTALKRMYGKY-L-RAESFRKALIY-QKKYLLLLL-GGFQECEDATLTLISHMG--------ALPGHCS-PHTQQRR--GI---------------------------------------------------TRFKSAARVSIALSKSRLRFLVKRWQRASGGST------------------TPQ-ILSRNGLAHGTGADVRNESSFLSPAAVELYRDRR-----ATSRGRTGRESPRSAASVQHRFVGVPGEAGGLSCSHLQN-------------YDPDRALTDYISRLEALQRRLGSVQSGSS--SYA--HFSVRR-------------------------------------------------------------------------------------------------------------------  
--------------------------------------------------------------------------------------------------------------------------------------------------------------------------------------------------------------------------------------------------------------------------------------------------------------------------------------------------------------------------------------------------------------------------------------------------------------------------------------------------------------------------------------------------------------------------------------------------------------------------------------------------------------------------------------------------------------------------------------------------------------------------------------------------------------------------------------------------------------------------------------------------------------------------------------------------------------------------------------------------------------------------------------------------------------------------------------------------------------------------------------------------------------------------------------------------------------------------------------------------------------------------------------------------------------------------------------------------------------------------------------------------------------------------------------------------------------------------------------------------------------------------------------------------------------------------------------------------------------------------------------------------------------------------------------------------------------------------------------------------------------------------------------------------------------------------------------------------------------------------------------------------------------------------------------------------------------------------------------------------------------------------------------------------------------------------------------------------------------------------------------------------------------------------------------------------------------------------------------------------------------------------------------------------------------------------------------------------------------------------------------------------------------------------------------------------------------------------------------------------------------------------------------------------------------------------------------------------------------------------------------------------------------------------------------------------------------------------------------------------------------------------------------------------------------------------------------------------------------------------------------------------------------------------------------------------------------------------------------------------------------------------------------------------------------------------------------------------------------------------------------------------------------------------------------------------------------------------------------------------------------------------------------------------------------------------------------------------------------------------------------------------------------------------------------------------------------MDSYRSYWSSPVLRDG---------------WLETQDQDLYEVESRIPLPRPFPLTPLLTERNAVVVQTQISHVRQRRDGGHLLKVVSRISLPTPPYTSLKCMKTSVYIELR---------EKFRHNEEMEELLRRQEELQERVCEEERAREQYCMCVCVCSGLIDGYSDERRALEQQVCERAELQLHLEQELQVTSTRLQELEQERCSLLEHTELMSRQRDAMRDSAG-----------GGPELRLVEAALDAAPEADLLEETEKLLQEKVEVQRQAQKQSSELQAQVKQLEAQLEEQQMRLQEQQELQRSQEEDLQQQIQ---------------ALEKQTENHRRFIDEQAADREHERDVFQQEIFNLEQQLKNPTKTQTGSERRDRE-------VQELSAALQEKSDRCSELLLSSEQLQRDVKDREEEIQTLAARVHQLEHTLMHEVSLCDAVSLEAQLQTEREALDRKEKE-------------------VLPLLFSIYEFQNEMHELNPHPESVLMSRLLQ---------------HQCMSVNLLKVLEEKERQISVLN-----QQISKRQHAGTHPEEESLNLLPSTELHQLNQTVFDLHRYFEELNMNHAVEQKDEALGEMEALVECLKSEQQRLKK--DNEDEVEQLNAVIEKLQQELSHIEARDDHEEMKQRVDELTSECNTLRLQYEQLQEETRDHEEMRKKVEELMSECSSLNLQYNQLQEETRDHEEMRK----------------------KIEELTIECNSLQLQYKQLQEET-------KDHEEMKQRMEEL------TAESNALRLQCEQVQVET----RDPEEMKLK---------------------------------------------------------------------------------------------------------------------------------------------------------------------------------------------------------------------------------------------------------------------------------------------------------------------------------------------------------------------------------------------------  
-------------------------------------------------------------------------------------------------------------------------------------------------------------------------------------------------------------------------------------------------------------------------------------------------------------------------------------------------------------------------------------------------------------------------------------------------------------------------------------------------------------------------------------------------------------------------------------------------------------------------------------------------------------------------------------------------------------------------------------------------------------------------------------------------------------------------------------------------------------------------------------------------------------------------------------------------------------------------------------------------------------------------------------------------------------------------------------------------------------------------------------------------------------------------------------------------------------------------------------------------------------------------------------------------------------------------------------------------------------------------------------------------------------------------------------------------------------------------------------------------------------------------------------------------------------------------------------------------------------------------------------------------------------------------------------------------------------------------------------------------------------------------------------------------------------------------------------------------------------------------------------------------------------------------------------------------------------------------------------------------------------------------------------------------------------------------------------------------------------------------------------------------------------------------------------------------------------------------------------------------------------------------------------------------------------------------------------------------------------------------------------------------------------------------------------------------------------------------------------------------------------------------------------------------------------------------------------------------------------------------------------------------------------------------------------------------------------------------------------------------------------------------------------------------------------------------------------------------------------------------------------------------------------------------------------------------------------------------------------------------------------------------------------------------------------------------------------------------------------------------------------------------------------------------------------------------------------------------------------------------------------------------------------------------------------------------------------------------------------------------------------------------------------------------------------------------------------------------------------------------------------------------------------------------------------------------------------------------------------------------------------------------------------------------------------------------------------------------------------------------------------------------------------------------------------------------------------------------------------------------------------------------------------------------------------------------------------------------------------------------------------------------------------------------------------------------------------------------------------------------------------------------------------------------------------------------------------------Q-SSSWQQEKAELLYSLQEAQS-------------------EVARLRAEG------RQLVQEVQ-----TEPHQTDSSHMYGRY-L-RAESFRKALVY-QKKYLLLLL-GGFQDCEEATLALIAKMG--------AYPSSADIQRR-SRHSKAF---------------------------------------------------TRFRSAACVIIAV--WRLQFLVQKWKRAT----------------------------------------------------------------------------------------------------------------------------------------------------------------------------------------------------------------------------------------------------------------------------------  
--------------------------------------------------------------------------------------------------------------------------------------------------------------------------------------------------------------------------------------------------------------------------------------------------------------------------------------------------------------------------------------------------------------------------------------------------------------------------------------------------------------------------------------------------------------------------------------------------------------------------------------------------------------------------------------------------------------------------------------------------------------------------------------------------------------------------------------------------------------------------------------------------------------------------------------------------------------------------------------------------------------------------------------------------------------------------------------------------------------------------------------------------------------------------------------------------------------------------------------------------------------------------------------------------------------------------------------------------------------------------------------------------------------------------------------------------------------------------------------------------------------------------------------------------------------------------------------------------------------------------------------------------------------------------------------------------------------------------------------------------------------------------------------------------------------------------------------------------------------MAARMFRLYSADQSFDGESVDEDSDSR---------YDVASP----------------------------------VHN-------ASSDRILELEETLE-----GKELALRMMQ-------QELDDATKAMEEAQDHALSVQADAQAAAGATPPPNNIDAHQQAGDNHHWQSASIQ------PEPLTSERE----------------AEYNAAIEQYNLK-IIEFQTALAQRD-DIIKQLS--------DNMQGALQSR----------------------DEVQREASNQAENLS-------HQIQSLQSQLRTAGETLQSQGKTQVNELAEELTKAHD-QVVNLR----QTVADKEALMHELANRYSKKSEELFDLKEEKEAVVR---EHAGEVA---TLKH-----QVSELR------------GDGGALPKE-----ELIEVLRAELDESYGN-QMMLMKQQLMENHH-----QETELLQQRCNELQVLARSNAGAADVEVGALKNQLDEQGKAFQMKADEAEVLKEQLQKLVTEFSR---------------QNEEIEQKHAADNDILRR--ELYAESKVKMDELESEKQRER-EYQADV--VSDLTDKLNQALNDSKELTEIRDTYEGQITRLMKQLEETQRDSETAADLNAENEQERKKHADEMEELKEQLNRIDE---------------------DNRELTQIREAYEGQIARLSSELENKPNFDAES-------DYNGKEKDEQLAEYEAQVQELERKLEESKAS----------------------------------GPSMDKLQEIREGLAAQIKMDYDDLMEDLKYDHEGQLKRLKVQLDVEYKDNLRDATKAVEDQLEQSIKKVQTEKE--REFVLELQNVRAELQEQYDEKLRQELDKG---RSETPSEEPVAFGMVNEEASQGGGVLTELKKDLMNKLEIEVQDLSEVRDDLLEQLEVATRDQETVREELAAATF--EKNNLSAEVEQLKDELLG-----IHFQADTSTDSPFYDNE--------RDELN-----DKMQVMN--------------SKLQSQEQDHEIEVSGYIAKMEAL-KQEIAKATQVHDQERGELDNKIQELESQLKEKVDDVPSE-------ELQGLG--ERNASLVSEIERMQRDLSDAK-------------SFIEEHGQRAVD-----LDSRNQALEEQVEQLQKQ--LELSGHEMEG----LQEAMTSLREVQMMEMQQLSEE----------KPRLESDLAEANDEIERMKNAQSKDTSEEAT-AELEDKLRELE----------EEKR-------RADELLEKA--VQE--LERMREEVEQSEERIRDLEGEVCR--QADERNELDDKMSTLE--KERDQLLTEK--EELQHQLETEEKERESQVGELESRIASFIDREDDTADADG-VRQERDSALG-TLDSLSSQYRT-MDARN------KELELHVENLEEQLTRQQSMQIVRDVEIMAQ---RETFEEAVANYDRTIGELRAEKEEIRMELERQGTPDKPSHLIPVEEEMIVAG-----TVAPVASVAAAPSPGEG-----EDTSDGRDFTSLPSDLETTVETFR----TENKDLK-----AKLAEAS---EETDFLL--HREHQLK----QELSALKVLLQEGEEEKEALEDLIEEEKE--------------QINELKMVDEDLE-------------RENQQLKEVIDHQP---------------------------------ELLEDQAAVIAGLEDKIGKLKKELQENQELYERENVLLKEALQEE-------------RDTTARLLNEGESPTSRD-----------------ALLQEIIDLKKRLAH----------------KDQTEHE-ILTEKSLQIG------------------LLQETNAKLEE-----EKYD---------LSTKISDLEK----------RMQASEAISQEVQDTFGRQYLELQS-------------------EQSALKD-----QLEKQKTTSNGQPLDEQALREEALRCAKDMLLQKLE--------------------EKE--------AVEQQMLDEK-MELQKQLGNQQSLEELLHEK-----DTLEQELARQKRSLQSEVKELEQKLQDQARKLQN-EKS-----NLECELQQKDFELRKWEASFREQQ-------------------------------------------LEYRNREAFVRRKHDNQVGSMMTDSD-----------------------KEMVA------------------------------------RLEGMR-----------LILE-RQHAEAVSRLRRDLEQ----------------------EFSSREG---------------------------------------------------RQTE------KHL--SDISLLKSRHQSQLEDMK------------------AEFESR--IEGAKEELEEERKKQLGTVKTVHERVMEQ---DQAEHVKTLQASVRT-----------------------------------------------------------------------------------------------------------------------------------------------------------------------------------------------------  
------------------------------------------------------------------------------------------------------------------------------------------------------------------------------------------------------------------------------------------------------------------------------------------------------------------------------------------------------------------------------------------------------------------------------------------------------------------------------------------------------------------------------------------------------------------------------------------------------------------------------------------------------------------------------------------------------------------------------------------------------------------------------------------------------------------------------------------------------------------------------------------------------------------------------------------------------------------------------------------------------------------------------------------------------------------------------------------------------------------------------------------------------------------------------------------------------------------------------------------------------------------------------------------------------------------------------------------------------------------------------------------------------------------------------------------------------------------------------------------------------------------------------------------------------------------------------------------------------------------------------------------------------------------------------------------------------------------------------------------------------------------------------------------------------------------------------------------------------------------------------------------------------------------------------------------------------------------------------------------------------------------------------------------------------------------------------------------------------------------------------------------------------------------------------------------------------------------------------------------------------------------------------------------------------------------------------------------------------------------------------------------------------------------------------------------------------------------------------------------------------------------------------------------------------------------------------------------------------------------------------------------------------------------------------------------------------------------------------------------------------------------------------------------------------------------------------------------------------------------------------------------------------------------------------------------------------------------------------------------------------------------------------------------------------------------------------------------------------------------------------------------------------------------------------------------------------------------------------------------------------------------------------------------------------------------------------------------------------------------------------------------------------------------------------------------------------------------------------------------------------------------------------------------------------------------------------------------------------------------------------------------------------------------------------------------------------------------------------------------------------------------------------------------------------------------------------------------------------------------------------------------------RDRELERQLDKQKMDAQEDE---------LIETQRLVKR-LERENKRIQETQADDDLHRA------------------IDKEL---------HNESGSPQRRR---------EGRVGKTE---RASLDLYKGQLES---------------------VRQRLQLMAVKQQEELS-KIERATMSADSVPS-STVKELRSVDGALVELITELRQVHAALA-----LLSES--------------------------------------------------------------------QP-----PLVTS-TASRINERLLEQN--AEMSS-------------------YVSQLSTEKSELRAALSKLEEEIWQYRQREVQRHRIQRLYGKY-L-RALSFRKALVY-QKKYLLLLL-GGFQDCEQATLSLIAKMG--------AYPSPEDLRDHPSKHGKGF---------------------------------------------------TRFRSAARVVIAV--SRLKFLVRKWRRATRSGS------------------RDLMGSGNAPPSASQPDRTSAAPP-----------SAAPP---STSTNQSPSTQTYPTGAQR----------------------------------------------------------------------------------------------------------------------------------------------------------------------------------------------  
--------------------------------------------------------------------------------------------------------------------------------------------------------------------------MCSVLELVGLQSLSDGLVVSWLPDG--LKVELTKSKLDSLRAEFESQMQRLQEELDTERQHQLGTVKSVHERERSRELQEMKDEHQKEMDALREDMDVKHHDIFDEMLKKVDENHEEEIRQMQIDSEKEQKLHLEALRITMEQTNAARVEVIRAEMDHSKAKALADLRE--SLIETHRNDLAMLTNQLQSEWTDRMDTLTAEHEKEIETLRDEITQRSQQELEELRNNHKKVMEELTKELETKNDALIAEKKNKSETEEEQKSDVELLLEERQKMLEEAEAVQQNLARIHNQEMQEMRVNMNASHQELVDDLVAAKKAHEETNAKHLQTIAELQHKVDQVDIEEHEALLEEVEALRQE----IGRQKKKSEKEHGREMEKLRGFFEEKLEETEKSWAEELDRVKAEHLDDIERQQQDDHGLETEN-FEISALTRHQQYT--FTQISISDAESSPPPSPYKMLESGEHLTQGMDEKLREKYRIDLEKMKTELESEHKAEIYRLKSQMELKYELDIKSC-KDELNNRHEQELSKLQQSHLEQLEAEKQYLTDIHLKE--VTRLRMVSAADAARQVETEVENLRSEMEFSKKEELSEIKDELGHEHRMEVGAMQADFDIQKQVEVKRAQAECQKELDEKIEEMNQRAEDR--LIEEVAHVR--SELAIEN--------AQQMEMMKMNLEMQKSKELELMSKEFEIERERILTEMDEKLKSLEQELAEKAKIDKEDACVTLQQKHENEMK------------------------NLK-KQLDEEKIKYSLLKDSIDRGENPEIALLKERLEKQYDSQVELIKTEITQEYEGIIQT---MQEQATDHLQGHQTLLDKFVEEQETEVMAAKEVHASDLV-------------NLREALQQQHQNEIEMLKGMHCREIENLKVELVQKLNEEKERMDKEKVNE-FDVHKNELEKLKKEHLVELELQEERVKEELTASHMEKFRAVTNELEAAHQ---------------REIEATKEGVKEYIEAEYVT-RIEELEK--------------------------DVADREDILKDFIEKHETEVAELKESYTEKIKAEKMKVRSQYDDRVDAVNMDTQREVDAAKSDLEEVHRQEIIELQSKLEA-EHTDDME-LLERAKRQEIDTLKQKHSDDMEKMKAELEEKLRHT-LEEIEGDYESQKEQEMTAFRESLHSEYRRIKDEMANQTREREQQYVAESDAAFESIKKEHEIEVGRIQK-----ESEQLQQQLEREQEIVAKLKDENEKLQTRLKEEVEKHDTTMNRREREMEESDNLLAMLRSDLDR----LNTERDTTQRINDHLLRVMTESVRVSLATGEKINKKLASILPDSRPDIEGAVALERE---SGV-------------GSDRPDESDSGAAMQGANSDSQEKEGLGSDDGTLLDTSVTSITDEGLEISQLTESMFVGPDLGPEGEEIVLGAGSRLQ--------GSVETLLDVIVTTTQQLHQSQELLTATTET--QEELRIVNEELNERLQAQEKEEHEHHVDQGNCTLCALKLPEVIQFIYLLPGDFYLNVFGSHE---LTDGHSVESLREQLTKKEENEQELVEQLEVTTTRLKQLELAQHQLREEQEQLDQQKMALAETIDTQELVSGETTVSSKISASGETAAGGETAADDETATDN-------ETATDGETAAGGETAADSETAAISETAANSETAASGETVSSGETAADGETAADGETAANSETTVSGKTAADSETAADGETAANIETAAGGETAAGGETAAGGETAA-----GADDETAADDETAAVGEPAADGETAADGETTANTIETAANTIETAANSETAANIETPADGETAADGETAANIGTVAS----------------------GKTAADSETAAGGETAANSETTADVGLLEENDRLCQEKKDLENQFKKDKEDLETRQKLLE------SALEEQTSRCDELVREQRSQTEEYERQFEAMDKQLKSNKLFLEQQATERE---QERDDFQ-KEIHKL-------------QEHIKMKGG-----------LRGDDTLLKEIQDLTQELKDKISAHNEVVKEKQQLVHDLQEKVT-LENQLK--QQIRELENQVEEK--NRVEQDLIEKKSELENELKKKEQIEED--------------------------------------LMQEKEVLQQQLYDNLLQLGALQSK--LDSTKHG-FDEKRSTTTSAASSTDGVSVAQQLEQDKEAIERKDEEITNLVEQLEQFREEIMDKEEEIQ-GLNMQIEVHRKE-------------IDAQKESLHELEKTKEEHEQFLSTLEKTERGDKAEEQTISKFSHKLLEEKNQELDDLNEQLSQAQEEIQHLQQ------------------------------------DIRQREQENKDIEIENLKDQLSKSHDDLEHLQQALEH--RAADTSGEGKDIEIEQLNTQLA------KAHEELEQLRQEIEDREADTGANVEVLANELEQLRLEHETMLDEKEQEISNYKLHLENEKNKDPLINPLVVETLEMTRNHHDEIKQQLEEELY------TLRHLMSEREQEVEHLEEQLARLRTRSDSP-GSSDDERNMQMDQLEAEIQDKDAHIARLKVQVDNMQDAIFKKQQELNNAQRSTSDFNENLER----MKVLHAE--EIAVMRKSQEEEIKQ-VKADAETRYLQKLSTMKSLSPATARDLEQSMYELRAKLTEQHQQHIRELSDKLERDNEIELESLKIKHEQDKREMR---------DEHKKEIEMAIVKTRRDLIQQHRLEIQTLQDEYHRRGDSISSPSPTPSIRELGRRLQHELEHTERLDSELMSQLRTRQDGVGQEGSPISG-------------------ATGDSMSEPGAGGDQVTSRLQALLNRVYNEGLQVLSLSDLVYLRRHSSPR--PKTPEGADLGTLQQAWESEKHTL--LAAIQALKDLISQTTASGVWTAES-RENDWRAELLKAIKYVFDKERETLLAELRTHVVAHGERNLSDIQELERRIKEQEEHQRSALEQIL--AADRASM-LAELRDLRAHLTVVQKERDEQGQQMIQQINTLE-SHDEQRER---QSKRQSIQNDGAIFIG--AGSRDSVQRLVVLIRNLTQTILEMLEYKYQ-QEKVLAEDIKSALDLEKTKTVELSTGLTKEKSIVVELKNEIS--ELQTKLGNITNNFEREQARLEAVT-STLEAEKTENKNLREELE--AERQTAEHLKCTFENENERSMQSSHRDQETIKELRTNLEKEKTKRTEMTNMYERQVLNTNALDRELQSEKSQSKTVI--SAERTKAAEFKASLEVEKARSEELSAALQRER-----DLSSKFRESLERERATLEHTSQRDHRAMADIKGELDAARKKSLDLHTALEQSKNTVATLNAMLESEKSRSLED--LDRERAVAKQLKAS---------------VDSLQSQRQDLTRQLDNERDRGVRLRNERDRLQAQLMSQKDRERENEAHRERER-----QL-ERQKQRERDREKELDKQRQRDRDLDKHLERQKSETAQMEIRELKQKLSLVEQQRDFDR-AREIHRRTMNGSVVETDNEE------------------IQQAF---------ETEISKGAQRAGFDSLSSSKTGITPESS---PSKVAMYKQQLEN---------------------ARQTLQLLVIRHQEELGKRSVRSGDGVSSHDP-YSDVEIQRLQKVLNEISNELKNVHSSLT-----SEEDP-TDGSPAMSNLNARLLKQNADLTTFVSRLSEEKTELRGALAKLEEEVWKYRQKESELDHYTKKSLDSAET-----ILATE-RAAWARERLANQRALQEAES-------------------EIVRLKSEYRHEAMKRETLQQQQEFSQPSETQQAKLQRMYGKY-L-RAESFRKALVY-QKKYLLLLL-GGFHDCEQATLSMIAKMG--------AYPSPADLQRR-SRHPRSY---------------------------------------------------TRFRSAVRVVIAV--SRLKFLVRKWRRATRIGS------------------SAVGGTPTALSVQSVPHTSTPSPSRSRDAHMLSYAAYTPP---VRDRHTRSATQPSPRSSYLSQRSHGASNNDPLTVPSLSASLPLDDSVTS-IDDGTDTSLSNYIQRLESLQTRLERVQRGNTVGGNSRQYSGLYSKR-----------------------------------------------------------------------------------------------------------------  
----------------------------------------------------------------------------------------------------------------------------------------------------------------------------------------------------------------------------------------------------------------------------------------------------------------------------------------------------------------------------------------------------------------------------------------------------------------------------------------------------------------------------------------------------------------------------------------------------------------------------------------------------------------------------------------------------------------------------------------------------------------------------------------------------------------------------------------------------------------------------------------------------------------------------------------------------------------------------------MKSASTANSRSSSTSHLSPRLPASTKSATSKSASSTVASSVRFKLEDEILGSVSSSATSHD---NDDSLEIMEEIETQDEESGSIREEGGDDGTSSVSEKVVPLNVGRPKTAATTAVASGSSKKRQLFDIDDDEPELELG--------GGGGKFDIDELQLSGDQIAVHFQPEEGEEEESEGDFVVRLANKKKLMQQNHDVDSLEQILNDIESGSEG-------------VEAPAPVQKELSPLNDDDVLINNSKVS-------------------------LNALKKLQKQSHVHENNNSGDNSLNTTNDISELARDTESDRVASDRSEGK-MSSSEGRSKSVDGSTGGSESRGGEDKPFASLGALKFEEEGDDRDQEDNSIEEIVMSNPSVVASSSVANEASSF-EENVTG---------------SEFDKSKEVRGQSSPEDLKEKILTSSKSFEEVKSQ--HELEVPSDKLM------EDDKATAVKQMEKVTEFKEALEDISEESEPTLTTSNHSGSKPSTDDEQSRPFVVLEKGKEMRRILDENIFQKQLSIG-STIYEDNKENIPDSLSLNKSIDFNSVVSLNMFQAMQLEMKELREIIANKEA--DSFSRRES-----LKEP-----RPDSLKDNP-----RSDSNSLATNSTEYRPLN---EEPPAQKDHTSNVYR---WVEILAEKLQESLQDRDRLQAEVDKQAEEINQIRKQLTENVEAIRGRPH-------WMRDQEST----GQRISEISIDLVSETDD-ALSDFPDMYEERSNRNSRERQLDLNLDIDYSEQNPLHIPTAPMTKQLEQFRKYLSPDELRLFNMVQGKFDDYINLEMHKLRSTHEDESKILQERLEAEKNEREAEVSRLRQMLTNVKSGSTEIVDLRTELEARHT--------QEMADLRTYFEKKCVELEKQYSEEVFSMHSRRVSANDTVSDISDQEEFPEENGGYQSKHTSPKRKLKEEIYLSPTHHKITPTTIDTAGEGSADEVMEV-VAEVEKDIQMNPDQLNQFYKTKIADLRRKHDADIKLLQERLKYFEDKEASEEFILNTEAPVVSANASHLTTQAKPPPPKTPSTTSTNVVTPTNNNINPTTTADAGTEPSDDLHEIISDYERRLQEQVALARQDVLRELEVQIQALLTDTVVEDSHWPPELVLLREKFTAKSQLEIAQLEIKHEEEMAKVKADFEKQLQWKLKRQSTFDSTR-DLDKIISERDNLRELSSTLRSVLGELVKYVT--------------VCEDDLNATLIGELHKHGIAVASGGDETLDGTLAVDLNETGASTCS-TARKLHKFTPDVSGLASIIEDPSLLHYVSKEETG---------RSSLNLDECLERLRLEAVHLLKLSEKVTRKTD----TKDEDLDKASVKSDSCEEEDGLKRGGNSRKDGASTRSFDENMVREVESVVP--KLQTAT------SSLPTDLTTVQSSGELNIQLHELRNRLLKTEDEKRLLETELADTLTRHNSLVLELNETKQHLLELNSQR---------------VEFSEGYGTNALIPCTHQPSNSFVELLDRSKHVLSSASDNTVDNAAAAN--LLQLVEDFCREGERYMDDGKRE---------------------------------KVDLQSQIEAADKQLKATRMFLEEQAIEREQERDEFVK--EIQRLKTQLREKDKDKVSFERAT-KELEAVELQSKELGAHLAERDDRIRKLEADLKDSIDKGFTLREIITELESQIDGKSVNEHVLDSKIKELEHYINAQNRQNESLHQEMESMKTDLAVRGYDDKIAKLEEELRQSRPSVEQSLVLEALTVQLRDIEETLERKTKNLETLHSNSAASLVCSSPSEDISMNQDSPLHRRKKSNESGTGLSGGGGEVNQPPPVALPVDEVQRIFDKLHRHTRVEEVAIKRINDLEMQIGGVRTSYAVSPLD-----------------------------------------------------------------------------------------------------------------------------------------------------------------------------------------------------------------------------------------------------------------------------------------------------------------------------------------------------------------------------------------------------------------------------------------------------------------------------------------------------------------------------------------------------------------------------------------------------------------------------------------------------------------------------------------------------------------------------------------------------------------------------------------------------------------------------------------------------------------------------------------------------------------------------------------------------------------------------------------------------------------------------------------------------------------------------------------------------------------------------------------------------------------------------------------------------------------------------------------------------------------------------------------------------------------------------------------------------------------------------------------------------------------------------------------------------------------------------------------------------------------------------------------------------------------------------------------------------------------------------------------------------------------------------------------------------------------  
------------------------------------------------------------------------------------------------------------------------------------------------------------------------------------------------------------------------------------------------------------------------------------------------------------------------------------------------------------------------------------------------------------------------------------------------------------------------------------------------------------------------------------------------------------------------------------------------------------------------------------------------------------------------------------------------------------------------------------------------------------------------------------------------------------------------------------------------------------------------------------------------------------------------------------------------------------------------------------------------------------------------------------------------------------------------------------------------------------------------------------------------------------------------------------------------------------------------------------------------------------------------------------------------------------------------------------------------------------------------------------------------------------------------------------------------------------------------------------------------------------------------------------------------------------------------------------------------------------------------------------------------------------------------------------------------------------------------------------------------------------------------------------------------------------------------------------------------------------------------------------------------------------------------------------------------------------------------------------------------------------------------------------------------------------------------------------------------------------------------------------------------------------------------------------------------------------------------------------------------------------------------------------------------------------------------------------------------------------------------------------------------------------------------------------------------------------------------------------------------------------------------------------------------------------------------------------------------------------------------------------------------------------------------------------------------------------------------------------------------------MALPGGE------ASQNQ-VVHPAAEQVEEVIINVVKAQDPRG----------------AQRKAHLLPTEPVSCWGIDVVLPRAS-LDSRVCVWIG-----LPSIQSINTSSVRA-VGPDVKIQKAKHNIND---ER---FTEMEADQALVDTDSTESTRRFEDVG------GYVTRSRFRDATFS-FKQLWIRGHIRH-LLKVER----------------IGNWTHHQTASAGEGKGLRLKIKHTTEKIVAAGG---ELQHERDVLQERMSEQSLKITTLQSKLDEQRMRAEELHRQGTSHLTVRVHDLQEELVN------------------------------------------------------------------LRETLHTRDKQIDNLKNFLENSRQVIERQEKELAMT-----------------------------------------------------------------------------QASNDRSQFEIKLEAELQAKTDEVQQLKHKIQHEMINKVALPDLMETMLADKNDEIDQLREKLN----QLQQT---------PAAALAT--------------------------------------------------------------------------------------KDNDD---NARTLSDIVSITDCDES-----------------------------------ADMVMRRAPEQS------------------------EAGAGFFP-----------------------------PPLP--------------------------------------------------------------------------------------------------------------------------------------------------------------------------------------------------------------------------------------------HSIPMKG--------------------------------------------------------------------------------------------------------------------------------------------------KDHQLCWSEPGFEPR-----------------------------------------------------------------------------------------------------STAYEAEAS-----------------------------------------------------------------------------------------------------------------------------------------------PLG---------------------------------------YVA----R-----------------------------------------------------------------------------------------------SVSRAIVI-------------------------------------------------------------------------------------------------------------------------------------------------------------------------------------------------------------------------------------------------------------------------------------------------------  
------------------------------------------------------------------------------------------------------------------------------------------------------------------------------------------------------------------------------------------------------------------------------------------------------------------------------------------------------------------------------------------------------------------------------------------------------------------------------------------------------------------------------------------------------------------------------------------------------------------------------------------------------------------------------------------------------------------------------------------------------------------------------------------------------------------------------------------------------------------------------------------------------------------------------------------------------------------------------------------------------------------------------------------------------------------------------------------------------------------------------------------------------------------------------------------------------------------------------------------------------------------------------------------------------------------------------------------------------------------------------------------------------------------------------------------------------------------------------------------------------------------------------------------------------------------------------------------------------------------------------------------------------------------------------------------------------------------------------------------------------------------------------------------------------------------------------------------------------------------------------------------------------------------------------------------------------------------------------------------------------------------------------------------------------------------------------------------------------------------------------------------------------------------------------------------------------------------------------------------------------------------------------------------------------------------------------------------------------------------------------------------------------------------------------------------------------------------------------------------------------------------------------------------------------------------------------------------------------------------------------------------------------------------------------------------------------------------------------------------------------------------------------------------------------------------------------------------------------------------------------------------------------------------------------------------------------------------------------------------------------------------------------------------------------------------------------------------------------------------------------------------------------------------------------------------------------------------------------------------------------MM--GCL--VCSGATESTDVSNSLFHSKDPLQTAATTPAGTATSPLLASGAPQLPQPSFFAVPPPAQPNPPTTFDGHFFQDLSAAFPRPTTSTG-----TPEFLPRQINFSLVDSAESR-GRGVFREPAFIEE-IREE-------------------------GEEVKGRLEN--E---LESLKLSLDRVTSER----EGAAKKLQEKVDEIADLQVELGARNKMYEDLLGERKELKEELERVKAALDELEPLTVQLEVKEKELREVVEQLERSEKEVAEVNLLRASLAEELEQHTKESAGLKAEREKSQLLLST--LKQQIESLNKTIGHKDELVSKLEKDILNYSKNEEKYLEQLKSLDAKETELKIVQGNYKDRLREIEMLNEDNRFLNEDINRLKSELAKSSSAGSTSNVSYVQFLKQNCEKFE--EELRETKVLLTEKMLALERVRIELTSSQHDAEELKSQLKQKEMVMQQIGDDGNSLHEALSGIQSKMQD-------KLREEQERSATLQAEIE---------------RLKVQLQRSDNSSSPKPFSVEEIAEQLEKELNYSAQLDSSIMKAIESDDVQSEDD----AQRQPK-----YKQFNQAEDLRQQLQLEVDKATKLQELLEAEKHNSNAIQMQDAEIIEAMRLRLEAAIENEGTLQKLLSDERNKND--------------RLSTLVAGVQRTKSFDNYLLMK-TKSSPHE-SPSRRLNRSNEFESEVVARLESEIKFLTAQNERERERAADSQRVLERERSRFEKEISDRNEHGEQVKRELVRVTKEKERL-EVELDHEQEKLMLAHREIESLEKRIGALQESESMRSIR-RERVSGANSLEFQELRARLDHVEQERNLLQDAVQSLRTEVERRKHREAKLTEALSKENSL--LEAGQGTAVPEEFLTKLKDLNRMLESNARENHQQAETLRLM---------------MDERKALQQRIQELERYSLH---PGQYN-RDDLEERANHLFGKY-L-RSESHRKALVH-QKRYLQIVL-ATNEENELKALQLLTAQ---------GLTPNP-PE--PTSPKNRR----------------------------------------------------SFRAVVTAVIAI--ERMRFIVRKWQGGRR-VC------------------AKAIFS--QQFTPRRT-QSASTNLWARSPNSHFAEY-----SR-------AAAPHSQVYSGHYLRLPEQQQLLMNG-DLREK--------------LEEQYNRSNVNR------------------------------------------------------------------------------------------------------------------------------------------------  
----------------------------------------------------------------------------------------------------------------------------------------------------------------------------------------------------------------------------------------------------------------------------------------------------------------------------------------------------------------------------------------------------------------------------------------------------------------------------------------------------------------------------------------------------------------------------------------------------------------------------------------------------------------------------------------------------------------------------------------------------------------------------------------------------------------------------------------------------------------------------------------------------------------------------------------------------------------------------------MYTVFAQRIICYVPPNLSQFSSPSNLGPNKSPVVSRSEPSVRFKLEEDIIGSLSVSSASASGSNNEDSLEIMEEIDI-PDDES--TQDNDASGSISENLVLSAAPAENVPQRSVDNSKIATDRKKQLFDIDNE---LP--------GESGVRKFDIDDLQLSGDEIAQHFQSFSESTDLKDEDNKVNLAVKKKLLKQNHDVGSLGTLLDNIESSSDVLGSDP-KKEESDLKQKVGESKKELSPLNEDVVLINNSKVS-------------------------LKSLKKLQKQSNVLD--NNGDNSYSTTNDISELARDTESDRIMSDNNDNSSRSDVASKPMDSDKEASLQKLSSLMLRSKSAEIEEKNVESEDKPEFASLGVVK-LCSEQSSEVHDSSVEEDNSI-EEMLMSNASVDILAVNEMVLSENQQLKEDSSDSNLSGDLKEKILTSSKSFEEVKSQ--HELDVPADNLE------GNKPVVVKQMEKV-VDIKEALEDISEESEQTLTNSNHSESRPNTDDEQSKPLAVVEKGKEIRKILEEKLLHKQLNIE-STIYEDNKENIPDSLSVNKSFDLDSVVSLNMFQAMNNEIKQLHEIIATKDMMLEAYSTRDS-----LKET-----RLESLRDP----QRSSDSSSLATNSTEYRPL---NDETVSKKELLQKVADQNQWIVTLAEKLQEINQAREMERSESIKLVAEVNRLTKQLAENADTIKQRPH---WM----RDQEST----GQRISEISIDLVSETDD-ALSDFPDLYEERSNRNSRERQLDLNLDIDYSEQNPFHIPTA-ISKQLEQFRKYLSADELRLFNMVQGKFDDYLQQELDKMKTTSEEELKIIQERLVSEKNEHDAEVNRLRQLLSNVKSGSVEIEDLKSELEARHT--------QEMDDLRTYFQEKVAEFEKQYSEKVLSQHSRQMSN-DTASDISDQEEFPEENGGYHSKHTSPKRKAKEDIYLSPTHRKITPTTIDTT-ESSADEVMEFPMEEEDEA-HLTVDSLREYYQNKLKELKRQHEQDLRELRKTFKQFEEKDIDEKGLPECLPPSAGVILIDKSTTSTTN----------------------TTTVDIGTEPIDDLHEIIADYERRLQEQVALARQDVLRELEVQIQALLSDTISEDYHWPPELILLREKLTAKSQLEIAQLQIKHEEEMAKVKADFEKQLQWKLKRQSTFDSAR-DLDKIISERDNLRELSSTLRSVLCELVRYVS--------------NCEDDLNSTVIDELHKYGIVPGNAEE--VLECSALD--MSVSNISTLSTKKFLKFTPDVSGLISIIEDPTLLEYISKESVN--HDG---ANRSLNLDECLEKLRSEAMHILSLSEKIAKKSS----TKDEDLDKVSVKSDSCEEEDGLKRKV-VKKDTVGSRSFDENMVRHS----R--TMDAS----HIDSSLPMDLTAVQCSGELNVQLHELRN-------EKRQLEMKLADALSKQNSLVVELNETKQHLLELNSQK---------------VEISEGYGTNALLPCAQKQANSFSELQEKAKQILASSP----DSDIDNSTNLLQLVEEFCREGERYMENGKRD---------------------------------KLDLQSQIDAADKQLKATRQFLEEQAAEREQERDEFVK--EIEKLKIQLREKDKDKTNIDRVT-KELETAEQNIKELSAEIAARDDKIRKLENDLKDSIDKGFTLREIITELETQNESKAIEQHVMVTKIKELESYIDAQNRQNESLHQEAD-----MTVRGYDDKIAKLEEELRQTKPSVEHSLVLEALTVQLRDVEETLERKTKNLETLHANSSASLVCSSPSEDISVN--SPSHRKKSAENSSI-----------PP---LPVDEVQRIFDKLHRHTRVEEVAIKRINDLEMQICSVRNEYAELQHERDVLQERMSEQSLKITTLQSKLDEQRLRAEELHRQGTSHLTVKVHDLQNEVVNLK----------ETLQTRDKQITSLKNFLENSQQVIERQEKELAMNQADNDRSQYELKLEAELQAKCDEIQQLKNKIQNDMINKLALPDLMETMLADKNEEIDQLKEKLSQLQSQ-------HHPPASHLSHGKEDDNARTLSDIVSITDCDESDMVMRRLPEQN--EGL--LPTQHSIPMETPSNFFNLKEQHQTASTTHS-TTHLPTSSSNVSPLQH----AMQLPKATLFPLGFEGAFLQDFSAVFPRPTPSTGTSSGGTPEFIPRHINFSLIDDPRSAEGKDRLREPAFIEE-IHEQEEEKVMAQTMTASGGKRLSDISENVSEEDKGKLEN--E---LESLKMQLDRITNEKNVALERMHAEIQEKIENIEDLQVELAARNKLYEDLLREKKDLRDELEKVKHDLDQLESDACNLQKKENELKEVLESMTHKDQEMCDLKGICNKLREEIEQHVRDSETLKSEQQKNHLLIDT--LKQQVESLNKTIGHKDELMSKLEKDILNYSKNEEKYLEQLKVLDAKEIELRLLQGNYKDRMQEIEMLNEDNKFLNEDITRLKNEIAKSSSN-SSSNPSYVQFLKQNCEKIE--EELRETKALLTEKMLALERVKIDLTSCQREAEDVKSLLKEKEMIIQQIGDDGNTLHQALSNIQNQMQETSGNLNKKLREEQDRNTALLAEID---------------RLKTLLQRSDNSSSPKPFSVEEIAEQLEKELNYSAQLDSSILKAIESDDVNSDDE----TNEAIKKSKNGLADAVQLEELTQKLRVEAEKGNKLKEMLEAEKHNSNEIQMQDAEIIEAMRLRLEAAIDNEGTLQKLLSEERSKNE--------------RLSTLMAGVQRTKSFDNYLLMK-NKS-PHD-SPSRRLNRSNEFESEMVARFESEIKFLNAQNERERERAIDLQRALERERNRFEKEIADRNEYGEQVKRELMRVTKEKERL-EVELDNEQEKLLLSHKEIESLERRINALQEAESMRSLR-KDRSLGQNSLEFQDIRLRLENVEQERVQLLETNRSLRTEVERRKQNEAHLMEALSRGNSL--KESG----VPEEFLTKLKDLNRMLEANARENHQQAETLRLM---------------VEERRALQQRIQELERFNVN---SNRSN-RDDLEERANHLFGKY-L-RSESHRKALVH-QKRYLQIVL-TTYEENEAKALQLLNAQNVPM--GIQGLSLAK-PN--ADENHPRR---------------------------------------------------KSFRSVVTAIIAI--ERMKFIVRKWQGGRR-VC------------------AKAIFS--QQFTPRRS-QSATTNVWTRSQNSHFVEYNASPPSRDRAVFQPSVVHQVQNYPSQYLRLPESQ-LVMNA-DLRER--------------LEDQYNQT--NR------------------------------------------------------------------------------------------------------------------------------------------------  
---------------------------------------------------------------------------------------------------------------------------------------------------------------------------------------------------------------------------------------------------------------------------------------------------------------------------------------------------------------------------------------------------------------------------------------------------------------------------------------------------------------------------------------------------------------------------------------------------------------------------------------------------------------------------------------------------------------------------------------------------------------------------------------------------------------------------------------------------------------------------------------------------------------------------------------------------------------------------------------------------SRKESSVRFRLEDELLGSGSELSG----EGSASASISES-LSANDNHDDDSLEIMEEIVVSPVDND--HDDDDDDEQANVAEISSAMIALNRSKKKGSIGSAVADRKRQLFDIGEDEDFLPRSAERPPSGGSGSGLFDIDRLQLSGDEIAQHFQTTVESDRSEKEDSP--FRDRPKSSENEEYEDSFGQDGGEGESSVGERASGKGKSPKRPLELVQVHHAKELSPLKEDDVLINDSKVN-------------------------LNALK--QRNQPIVQ--RGG-------IRLEEKARNQKPPNVMAEESNNNSQDNSLNTTNDISDLAADTESERVGSVELDASSGSAARQGKLSADYRSRSVPDGIGAGKGSGAAGPSGAGFTSL-GPMKMSEAMSDR-------LSNEPSGSSSSGGSGISEDLRERILTTSKSFEEVKAQGVAAIEVLEDKTE------RPGGEPAKPMERVPVDQKEALEDISEESEHTVGGGGVKVTSRQQEGASPVPGNLIERGKEMRSILEEKLLQKQHSVVGSTIYEDNKENIPEGGSLKAGPEFDSVISLKMFQTMENEIRKLHEIIATKDVLLNAYGGRKEGGTAQGKETNSCSSRAESLREQPPCRSHNSDSISMATNSTEYRPVGELADMASAEKDLYGKIMERNQWIELLADKLKESLQERNQLQSEGEKLAAEVTQLKKQLAETVESMKLKAQSSSWAGVRPPDQESTSGGGGQRISEISIDLVSETEDGGLSDFPDLYDDPP-LGATERLLEPPGCSDFSELNPMHIPLK-ISKQLEQFRRYLAPDEVRLFNMVQGKFDDFLTQELDKVRDGGETENRVLREQLQIERAEKEQEVNRLRQMLGSVKAGSIEIVALRTELEARHT--------QEMADLRTYFEKKVVDLEKQYSEEVFSQQSRRMSNDDTASEISGAEEFPEENGGYQSKHTSPRRKHKEGIYLSPTHRKITPTTIDAT-DASADEVLVVEIIEEQEHRHLSAEEVREFYQSKIKELNAQHERVFANLRGKLKAYEAKEQEKHVLVSAYAERPEQVLG--SIRLQTP----------------------TSVGELGSD--SDLQEIIAGYERRLQEQVALARQDVLKELEVQIQALLSDSSFEDSHWPPELVLLREKFTAKSQLEIAQLQLKHEEEMARMRNDFEKQLQRKLKRHTTFDSSR-GLDKIISERDNLRELSSTLRNVVGGLAKYFS--------------ICEEDLNSTVLEELHRYQHEDGQTHEPSAVDCSANESALNVTDVSFLASCKLLKFAPDVSGIISIIEDPSLVEYVSTKAQQQRDDGTEPENISLNLEECLERLRAEALSLLALSERIKQKSSSGTTTKEDESDRVSEKNDSCEEEDGLKRR---GKSLESARSFDENIGRQQDGPDR--LGDAKSNGAHGCRSLPIDLTGLQLNGELNMQLHELKNRLLKSEDERKLLESELAEARSKQSSLVSELSETKQHLLELNSQR---------------VEFSEGYGTNALLPTAQRVNNSFVELQERAKVLLGSTTGAGADQTIEDSSMLLQLVEDFCREGERYLEDGKRD---------------------------------RMDLQLQIEAADKQLKATRQFLEDQAAEREQERDEFVK--EIERLKGAMREKEKDKVNFERAS-KELESMEQQVKELTTQIADRDDRLRKMEADLKDSIDKGFTLREIISELETQIESKTINEHVLETKVKELEKYIDVQNLQNESLHQEMESVRTDLAGRGYDAKIAKLEEELRQRQPSAEQSIVLEALTVQLRDIEETLDRKTKNLETLHSTSGASVGCSSPSEDVSVNQDSPLHRQRRSAGGAV-----------TP---LPVDEVQRIFDKLHRHSRVEDVAIKRINDLEMQISNIRGGYAELQHERDVLQEKMSEQSLKISSLQTKLDEQRLRAEELHRQGTSQLTVKVHDLQNELHNLK----------ETLQTRDKQIVNLKQFLENSQQAIARQEKELAMNQDGAERSQHEQRLEAELKAKEEEIRVLKERIKNEMINKAALPDLMETMLADKNEEIDQLKERLAQMTGKGAAANPRHDELVAAAALAKDDDGGRTLSDVVSITDCDESDMVMRRMPEQHTLEGI--LPAHSIPMVSTGVRCIHDHASSGRECQKDSGTSLLPNHSKQSSVPLGGHILGGSHDSTMLNSIPLRSNFFHDVCSVVPR-IPDQTRSSAPTPECVPRQIDFSLADVEGTPHLNAQLREPAFIEE-IVEGEE---LNDAMVAAHPAQR-----KAPNEVNSNPEG--E---VESLKLSLDRVVTEKNEQIEGLKGELQEKIERIEDLQVELAARNKLYEDLMQERKELRDEVEKVKQELGRLDQQAHDMQQKEAELRVALEQVRQKEIELGEVNSRYEKVRTEAEDATKEASALRGEQQRQKLEVDT--LRQQVESLNKTIGHKDELMSKLEKDLLNYSKNEEKYLEQLRSLDAKETELKIMQGNYKDRLHEIEILNEDNRFLTEDINRLKNEIARSNNS-LSSNSSYVQTLKQNCTKLE--EELQETKVLLTEKMLALERVRIDLTGCQQEMEDLRSTLKEKEMIIQQIGADGNSLHEALSNIQEKMQEKNVTLNGKLREEQERNAQLQSEVE---------------RLKQQLQRSDNSSSPKPFSVEEIAEQLERELNYSAQLDSSILKAIESDDMNTDDDRQGSSSGAAGRKPGPSRKPSDLDELRQKLQLEMDKGKKMHELLEGEKQNSAAIQQQDAEIIEAMRIRLEAALGNETTLQRLLEEERTKNE--------------RLSRMVGGLQRTKSFDNYLLMKGGKGSPQDGSPQRRLNRSNEFETEMMARYESELKFLTAQNARERERTADLQRVLERDRERFEREITERTEHGEQVKKELNRVLKEKETL-ELELDHEQERLELAHKEIESLEKRIGALQEAEALRSAR-RERTSGQNSLEYQELKLRLESVEFERNQLRDTVQSLRSEVDRRRVREAHLTEALSRENSLNAQNQQQQQIVPEEFLNKLKDLNRMLESNARENQQQAESLRFM---------------MEERRALQQRIQELERYNVHGSGVGHYHQREDLEERANHLFGKY-L-RSESHRKALVH-QKRYLQIVL-ATYEENEAKALALLNAQLPPG--QMEVLALASGPRSLDSSGQPRR---------------------------------------------------KSFRSIVTVIVSI--ERMKYLVRKWHGGRR-VC------------------AKAIFS--QPFSPRRS-QSASTNVWARSPGSHFVEYGSG---------------------------------------------------------------------------------------------------------------------------------------------------------------------------------------------------------------------  
---------------------------------------------------------------------------------------------------------------------------------------------------------------------------------------------------------------------------------------------------------------------------------------------------------------------------------------------------------------------------------------------------------------------------------------------------------------------------------------------------------------------------------------------------------------------------------------------------------------------------------------------------------------------------------------------------------------------------------------------------------------------------------------------------------------------------------------------------------------------------------------------------------------------------------------------------------------------------------------MAINIALFTLQITSLLRPHGASVSYKAIEAATLEELPATSVATATSTAATSRATGGAAATCVDVEFIPTLNIIFEKPTSSSEQLQCAEKSEHVAGGVSDKEPADDCDSDNDSTRTYIISRSSWTGVTSSSSTPYAVTSTDTAELLRRQNNLSLTEEDQSVSSFSIEQPTSSITIEMADQRTTTTTTTTATTATTPSSANILATTATTTTTTSSSIEEEIEEAIEISEVEEQLNTPLESLSEAAAYARPKDEQSSAK--------------------SLSSNPAEDEDADEFRIDDAQLSGGQLAQHFLVDEDESEEGSDLPAASKVEVSLSSNDDDFDISLPLEQRKPVHSLHEDDESVDQSLSNQSTTDDVSELVEEPVHEMDDKTDGSAAISASAPQET-QVDESQITDEQDHSMEEIVATNESIEVTYEAEETVNTSQKQDLITDLDEESEDQVHVDNRPTSTLQALKLPALQQPQIVERKAATALTTLRLQADVLEPLELTILEMDDDENEEDDDDEESSLQLMKLRLMAMNQQMIVDNAPKLSPTEAEQTQVTSSSNNLEIKQMERVPLTEFSKDVLEDITEESERLLSMSTTMEEEQDPPSLSLDESKTLLQPGAHKTGGSSSSLVSLNMLKQLEAKVQELHTQLETKDNCLASLNLQLETARRESSAGPASARDSSSLMTNSTEYRTLQEELGGPTLDIYVEMSRRDELIAKLTDSLQQSLNVRDKLQVDADRLGGEVQNLRRQLQETLDAVKRSSAVWPDQECNPGQRISEISMDLISESDDDLDRHFLTDNEERGSRSSKERQLTQLQTNEELGLQASNPEWTPTFSKQIEQFHTYLLPTEQRIFQMVQRKFDDYLNQQITLCRDLGAQELKIARDQWESEKLTSEQNQQAAHAK---------QMEDLRKYFEHKCADLEKQFSDDVFSHKSQHQTGDSSSECSEVDQLPEEGAAAVSSKEPSPRKRKRAELLLSPSHRQMTPCGLDSLGENTGKTEKDNTADIAD---------LKIFYQTHIKELKRAHEDQVRKLSDRIKFYERQQGDDDYKPATESPERTCPDQESAGGVANTSLIIIDEDELNF-----------------NNESQVIQRIIEEYEKRLQEQLALARQDIATELEQQIKSLLSENTVDDQHWPKELILLREKFTAKSQLEITQLNIKHAEEMSRLKLEFEKQLNRKNKRHLTFDAAR-DLEQVICERDGLRELSKSFRSVLCRLAKCVA--------------HCEEDLNATLSEEVQRLLFHSRSQDGGDDLEATISSSLNNTKHMLRVPDVHSLLEVVEDPSLVQFIDSKSNEEPSEDFDLNDCLERLKSEASYLLHLSEDLHKQRTHDESSEHLDEPEKQEHELCCEAEDGLKTTGAVNQQVLSKFLRTNSLNDQQMGVANQRKNSNPEAGKTHSSL--PPDLQEHAGNASELSFQLVQLKNRLIKSEADRQNLQQQLSRTIDRNAELGQELQALRDQLSQLNSLNHTDYNEGYGLGTMKS-----------LQEQGLDQSSASFLALQERARHLLSSSPVKEQPSRDHGNSTVILLQMIEDFCREGDKVVEFSKKDREDLQS--------------------------------------------QIDTADKQLKDTRRFLEDQAAEREQERDEFQR--EIERLKAQLRDKEKEHSSYANASE-EYAQLESQFREVNQQLCESNAKRDKFEVELKASIDKIFVLREIISELETQVQTKALNEEVLAEKAQQLEEYVSLQMRDNDILQQEVHSLKTDIGEG-YQSRIRVLEEKLKQSGPTAEQGVVLSQVAEKLRDIETTLDQKTKALESLHNSNATSNSASLSVTEDVSIHGSKEPTAVGSPSHPSLTVEG----------------VQRVTEKLDRHTRVEEAAIKRIRDLEMQVHQMRAGCVELQHERDTLQGRMEEQTQRISTLQNRLEEQRQRAEQLHRTGTSDLNTRVHELQGEVQN------------------------------------------------------------------LYEQLAARDKQMANMRQQLQRSKEEITRLETEVEVR-----------------------------------------------------------------------------TQP-DRS-LVNKLQAEVQQKGAEIVKLKDKIRTEMINRLAIPDLMETMLADKNDEIDHLRDQLEAKEKELQASQQEASQISSPSGAAGK--------------------------------------------------------------------------------------QEGSGGKLSARTLSDIGSITEFPEPDVERRAAMRSLTALQMSEGAGGFLHQTMETSKEAVANLTHKRTDDLSGFIVPYPVNTFEHPHYFQALGVTAQSTDGLTPGLVPRQINFSNLTEDSKLKT--TSLLMHTPELPRPTTPPEIHQLRVKLSDLQTEKQRQQSELEKKLQDLQKELEQEKEKLSRQAQTLQSYEESEAKYRLRIENLESKVLETAAQAASDRENLRKELNCVSAAH------------EQCENAAAARKRELEKLNSEVKVKADQLHAALRRCADLELQVLTLERDLERLKNSDNSSKQYSVDEIAQQVEKELNYSAQLD---------------SNILKAIESEEENNLDKKLQKGVQTEEETLPGTGNGTDDENFTGERELLNQLEA-------------------------LRAQLAVEREQCEAMSKELLGEKQHSQDIQEQDVIIIEAMRKRLETALDAEDELHKQLDQER--------------------ERCERLQTQLTSLQRAESRRNSSLLLKSPGDSPRKSPRADFESELGDRLRSEIKLLVAQNERERERSADAQRSSERERQRYEKELQERVAYCERLKQEMEKLSRDKESA-ETELEHFNERLTLQASEIESLEARLVTLQEAETRRANT-RTRQHQENVKLQAEIHELKSKLLAAEAARDCLDQKVTQLRFDVSRSGQREAKLAEALAQANDRLAHSTDDNVPAQFMQKMKEINALLAENTQENRQMAET----------------------VQFLVGERIALQKKCEELGGAGNTNVTELEERCRQLIGRY-L-RVESHRKALVY-QKRYLKLTL-EGYQASEQLALQNLRGGAE------------------QPPQRNIK---------------------------------------------------KKFKTVALAIIAI--QRIKYIGRIWHTGKR-IV------------------SKSVFTITQQRSPGLNLNVAPPQSPLPGPNSNLPTNNNNLMGR--LSYAPLSPPMVNFSTVQPIMLPSDFTLQAPTTSLQTT--------------IANNNSENTLPSLARLDWPTMQKPKRAHARHH----------------------------------------------------------------------------------------------------------------------------  
--------------------------------------------------------------------------------------------------------------------------------------------------------------------------------------------------------------------------------------------------------------------------------------------------------------------------------------------------------------------------------------------------------------------------------------------------------------------------------------------------------------------------------------------------------------------------------------------------------------------------------------------------------------------------------------------------------------------------------------------------------------------------------------------------------------------------------------------------------------------------------------------------------------------------------------------------------------------------------------------------------------------------------------------------------------------------------------------------------------------------------------------------------------------------------------------------------------------------------------------------------------------------------------------------------------------------------------------------------------------------------------------------------------------------------------------------------------------------------------------------------------------------------------------------------------------------------------------------------------------------------------------------------------------------------------------------------------------------------------------------------------------------------------------------------------------------------------------------------------------------------------------------------------------------------------------------------------------------------------MEGDSTNREGAPLGDSGSSSLTLSLTPPLSETSTISEHLSSVENDRSLPELLVNFPP--------DERISLEPKPPSLSDSYTSSFVSLNGLERFISTNMEGRTHAQSILEEILSNDQFKSDSDESLNIFANVDLDDKLL----------------------NSYNAGKSVNFDLNDEVCKRVKDFEDLIAVKNTTIAALTSELDSFRELLSNTSSLGTTTTEYKQFQEECHNKLVEYRDAIVHRDEQIEKLTSSLQQAILNREEMKQH-------------FRSEIAQLQEQLE--------------KTSNLLKDHKKSFDFDEICAQFEHSLGPPQLAPFGELKAAFASYVEMSKRALENEISNLKTKFSSDKAQCESEIKSELDAKHS-KEMEELRTYFEKKCADLEKNYS----------------------EEVFSQQSRKMSESSSCSELSSDFVGPGGDTRVDYTRKDLVKLRQSLVQ--LIKIFDGFNLEDLSDSDFERLTSEIGKFEFRNLLKFDLSLMKSELQNKYHAELEILREDYDNRVDELNVEHEKKLKSLN--------------------EEIDDLNRQLRGRIVTTSQQEVANSGEFEIDEVVQSYERRLQEQVTLAKIDIIAALELQIQRLVAS-----------------------------------------DSLDDDWPEELLQLKSRFADKYQTEIAALKQQHSEEIARLKDEHLKNLNAALERARRRSLRDADSLSKGELELLKERDLLKKQTLALRNLLGELIKYFTQCEDELNN-TLVEELMTKNFTQIERELDSLENTSGVKRVHLTPNFTELIDLIDNSPDK--------DLETIDLKSELGSCLEKLKADANAILQLSTSFAKSEELEVPKRKNESLELTRKLINETQIKNELVDQLSEARSIIHSLETDRGALENQLEQ-----LIERQKNLECDLLTARDKIAELIENGHKEIVSEGYGENGDRGVRGLAEAVAALSVLQDEARVLVENRSQVDQNVVKLVEGLARAGDKIVEEARRERED--------------------------------------------------------------LKQQIEAADKKYKITCQFLEDQAAEREQERDDAQKQIDSLKDHLR------------------------------------------------------------DRE--KDRATYERYTTEVEHLEQQLRDMSKLVEVGEKKVKEVEGERQEAVEKIYELRDIIRDLEEQVRVKTSTEEELRSIVSELEIIVKQQVTDEKKDVSENTKEFHQYVGDLENEVQRLRLTSEIAGS-EGALKQIKNQLSDFETTLDKRTKDLEGFHSTVSTTCSSP------SEDMSIQERSIYDECEIPLQQLARLKEKLLRHSRAEDAALKR--------------IRDLEMQIVALKNDLEESQTEREILRKQISDHLVLISNLQIRLDEQRIRAEHIEKQTNTSLEVQNYDLKNTIASLEEKIA--KRDKTIAQLKSNIDETKKRLEEREKEDDLVVQMQEQVEFLRSENAKLKEESKLLNLDEKEVESLNRLLLSGVSIHDLENFEQCRRQMTDSFPISPIPGRKNHETTFLSLPN------KANSTEKHVRFEDDQLKQLEGKIELLQTHLDETEENLKKATQLFEEEQKKSEEQIKLLEAKNGEIEANFRKLLQEEKTKSEEIQARLD---------------QANDEMKKSKELEANLRLQNEEIQARLDQGTEEMKKSEAKLQECSRRLESRDKE----VKTLYKVNNELKNSVKIKEEEIAEIPLLRKRNEEVGEELRHLQHLLLEKEKIIEHMEADSKSLHTNLETIQNKLQETGNVVDLS-------------------RRLREEQKKNAELFEELHTLKAQLLSVDKKSLEDITSQIERELNYSAYLDANLLNAVGEGSLESEDVDGLKLLLKKQKSVNKQLVKAR-EAVETKFELLEQKYENLKKE-LERVQLEDAVLIGQLRTKLTQTMDVEDEALRDLRESRK-YELGLKNEKIID--LESQISAFEHNERLLRADLEAKVNELGACRVLQEQSKAEIRKLKRLLSEHKAKSSDLVPDQIMEKIKELNAVARDNKQMIDLIARLN-----------------NEKLVLEERIVALEEHHNYLPFD--------DPSERANYLFAKY-L-RSESYRKALVW-QKKYLISLI--EMVYVTPVEHSIVPKR-------------------------------------------------------------------------------AKFKHVGIMVVSI--CRMKYLVKRWHSGKR----------------------IVRRKSRETVNFQVGQPVSSQNFVPSALNQPKTDMP----------WVGTSPPTKEPQVLRFQRIATRDALSSPG------------------------LLSKYVERFNQIQDKLGLDTQ-----------------------------------------------------------------------------------------------------------------------------------  
------------------------------------------------------------------------------------------------------------------------------------------------------------------------------------------------------------------------------------------------------------------------------------------------------------------------------------------------------------------------------------------------------------------------------------------------------------------------------------------------------------------------------------------------------------------------------------------------------------------------------------------------------------------------------------------------------------------------------------------------------------------------------------------------------------------------------------------------------------------------------------------------------------------------------------------------------------------------------------------------------------------------------------------------------------------------------------------------------------------------------------------------------------------------------------------------------------------------------------------------------------------------------------------------------------------------------------------------------------------------------------------------------------------------------------------------------------------------------------------------------------------------------------MDNEEKRRSRALEAGREMAYKLKHSNRRFSKSLEQASDEESFRNEAENQSIDLRSVSNNESILSRDITYSSVSMSEGEGDGDLEGLAGRVTELEELLQGKEAVVEALHAEIDHLRADASSPTSSHSQNSNNPYKDVIVTYRAKLQEFDRALNQRDNLIEDLTTSLQQALASRDALLAQISVLNSTQLGNPDRENICDEKISALENVLSIQKELVTQLSSQLEQAEQNVKRLEHEKEVQNTELVDYKDQIENLNKKIQSGSTEIDNSISDAQQVQKQYEKIKKDMEAVINGFKAETSANNAKHASEIKELSLRHEAELQKLRDENHNLETHHAKEMSVFQTQLTNYKRTIESLKLDLVTRKETHAKDKELLTEQIKLHKL----------QLDEMTTKYIATTSVLDSKESIERSLEQALNDAATLRTENESLKFKLDDLSSRYSAAQSLIENNQVHERTLSNKIFTLEKSLSRLSGISFAELDQTAYQTLDEMS---LQYQITKQKLEEKAVMEKELVQKIQDLQDLVSKTRTELESANLAKESYEKQIKDMKNTCDRLKSEMNSTLEHQQQRFS--------------------ADSLEVTRMPSCEFEQEILELKKTIEKRDEETAEYIKKLEELGGINQRLVEESQKLKNGLATAYAQCAVFEEKLDQTLGFGESKLDDTSALMDQTASSNGSTFDLEEVLHKQSNYNKVLLKLDSCRKQLEDFER--------------ERAQMLHKLEELTKEKEDILKEKEELLGNEGLKQLKIEMEEKHAKEMEELRTYFEQKCLQMEKQYSEEVFSQQSKKMSDNDSEIEELNEDLYVGSGPVELSGGGDALGVLEKEIKSDS----------FSDFKDKKQNNVSEEKIAQIAAEYEEKLENLRKQLQEVKEKSGRTILLK--AVNQFCQTEQSSTAAVQESCNELNKLRADYNRQLEEQVGLARIDIINALQEQIEALITVESESDENWPTELLELRNKFTSN--------TKREIQALRERHVQEIKCLREESNKIINGLHDEIKIIKAETRESLQSNLIKERDILHKTCATLKGLVSQLIGYFADCEEELN--------------------------------NTIISEVLKKQSTPRMDSRSVFECEQSAAKIKRVHFAPKSNEIASIVGSDTDLHDLINSEKDVMKILKNELEASLQRLRADSAKILNTSFSDSESWSTGLGDSPRDKLIEVEKLLAAFQEENEHLKVKVIELQQRVIMAENKKEVISEGYGEQDESVLEEVEEDFSQLQDRAKYVVMNGCNDTVYLLQLIDELCRHADKSIDDIKREKEDLQHQIEAADKQLRSTRKFQEEQAVEREAEREEATKQIKQLQERLRELEREKDRDYREYCIDSAEVETLKMQMHDLETKKLKTESELEAAVEKIWELREVIRELEQQVTARVNREDVLSGKIKQLEEVVVAQTKNQEELVQELEVLKSGNDNNQLS------DHIGHLQEELRKHQLSSEQLTANSSALKQLRLEIRELQSQLDRKTRELESMHVCGSSLSLSQPSEDVSIREQIDAARCPTPDDPTSPPILPLDHVLKLKETLLKHFRAEDVALKRLKDLDIQLSSLQRQNEELLAEQEILQQTTSEQLFQIETLRARLEQQ--KQNAPFAQRQATSRLETQLYEINDRLEITERALNDKDIEAKLNNQEKTQTNMPQLIDTMLTDKNNEIDHLREQLMQREKQLEVYFSIDEAQLRELLRHNEHQKNSARTLSDILSINSECEEVEA-IREAPNYTRQNVSNFKIPNSIIHSSGASKRDVVDFSQGIVDT---PKVPMLELDSHSQCSDAIGSNNEISKDMDNLQMHPHEQVSLARVSPT---PDTDAENQNESTASEDHDGFVCPRHGKVNNSTRNVEETVLNETITVQRIQELEAHLHEIKEELGAKSVALNQRDAELLEIQSHL------EQLQENIESLNEDRLYYKSEYEKTKESELKIQRDLEEVENTLKKKTEELEDFKEKIQVNEKILTEEAKRCKRSEATLQEKLKEITNLKEIISEKDITIETLQEKDKVIR-------QMSDDSKSLHRALETIQSKMKESGNVVELRRKLKDERRSNVELTEEIAKLRTEVEMLS-SSRRAEEDNDIAEMVQRELNLSARLDQQLLN------------VIDSEPEDRMKRMKCNDLKQANKEIDELNRFKDDLEIEREMLRSQIAEYENRIMQLKGDAEEESLRIAKMEEELARERHLVRSLQLQLQREKNSAEENKVRDTELISLLRIKLDEALEVRDRLLMEQKSHEVMNTLKDQNRKDIP--------------DFGTEFVDFEKQRLELAKKLEAEQHKYAEAQEIIKKLESEKDRIQKQYELAEAEIEKLTSSLDLADCVKDQMKS-DLRKAREELKAKNKECDWQKSILKTMSEADSKRNQQ-RTSDHSELKNLRRELKNAQEVINDFEADMKTLKEQLSESAEREAQLSRCIETLTDKETELTEQLSAAKAEDKKLRDIIADQQNELQLYLRREIELSEELKRERLSPNKNNSPSKVLQRVKDLNSTIERLSQEKVHLYEKISH-------------LREENERYLDQIRFLETQLSLKNKGYSNAVSRNDTEEKLQHFYGKY-L-RSDSRRKALIY-QKHYLVCII-AGYQLLEENTFAFLAQLT----------QTQRTYARRGMHRHHAR---------------------------------------------------VRFRSAVLVVISL--QRMKWLILRWRTGRRVGA------------------NVVLGNIERPAITQPIRHRSAGAMTGHSPPVKERATTNGTS---AFSHDPYFRRLTDIQQSLHLRVPESSTDNFKSD-------------------------------------------------------------------------------------------------------------------------------------------------------------------------------  
-------------------------------------------------------------------------------------------------------------------------------------------------------------------------------------------------------------------------------------------------------------------------------------------------------------------------------------------------------------------------------------------------------------------------------------------------------------------------------------------------------------------------------------------------------------------------------------------------------------------------------------------------------------------------------------------------------------------------------------------------------------------------------------------------------------------------------------------------------------------------------------------------------------------------------------------------------------------------------------------------------------------------------------------------------------------------------------------------------------------------------------------------------------------------------------------------------------------------------------------------------------------------------------------------------------------------------------------------------------------------------------------------------------------------------------------------------------------------------------------------------------------------------------------------------------------------------------------------------------------------------------------------------------------------------------------------------------------------------------------------------------------------------------------------------------------------------------------------------------------------------------------------------------------------------------------------------------------------------------------------------------------------------------------------------------------------------------------------------------------------------------------------------------------------------------------------------------------------------------------------------------------------------------------------------------------------------------------------------------------------------------------------------------------------------------------------------------------------------------------------------------------------------------------------------------------------------------------------------------------------------------------------------------------------------------------------------------------------------------------------------------------------------------------------------------------------------------------------------------------------------------------------------------------------------------------------------------------------------------------------------------------------------------------------------------------------------------------------------------------------------------------------------------------------------------------------------------------------------------------------------------------------------------------------------------------------------------------------------------------------------------------------------------------------------------------------------------------------MQMNERIIMELNSEN---IKLKKNIEEKEQELEQTRKYTTLLHEKIK-EVQNFRDIILDKDITIETIQTRN------------------------IEIENEN------KQLYE-----------FKTKYQSIKQELLECQTEIQRLTEGLNNRDQIIRRLEEMARR---------TSVSETSSPSNEKDQEIHHLQEYLKEK----------DKMIR-------QMNDDGKNLHKALEIIQNKMKESGNVVELRKKLKDEKKLTAELRDMVDKMSKELSDLKLIAQRSQDDTDIEDMVQRELNLSVHLDKQIMN------------AIES--DTKTHKTECEKQHNS-------------------------APYQD--MELK-----------------------LKLIQIEIEKEHRTMQIEHIRNSELIDFLQNKQKNSLDN------EAKLKNELNLLRQEYKN------------------------------LEMQLSLMKEHG------------------------------------------TKDHLQT-DLRRTKEELKAKEEECEWLQKRIKTMSDAETRRQER-STSEHNDLKASRREINNAREVIMDLEADMKQLKRELTESLEREVKLTETMETLKERESDLIKKLTTAKDEEKNLKDVITELQQDIKTYTIRELELTKELKN-RFS--NENVPAKFLQKIEDLTN----------------------------------INEKYLTEKTILQEKL-----------------MKLQHFYGKF-L-RADSRRKALAY-QKRYLLSIV-GGYQLSEENTLSVLAQLT----------KVQRSYAVIGRNKKSPK---------------------------------------------------VRFRSAVLVVISI--CRMKWLIVRWNTGKRIGV------------------KTLLWNIDQSFL--PIQKT---AMN-HSPPVRDKNTSNGDGGFDGFTLEQYYQRLKNIQQKLGLAMAETGNLQIIPE-------------------------------------------------------------------------------------------------------------------------------------------------------------------------------  
------------------------------------------------------------------------------------------------------------------------------------------------------------------------------------------------------------------------------------------------------------------------------------------------------------------------------------------------------------------------------------------------------------------------------------------------------------------------------------------------------------------------------------------------------------------------------------------------------------------------------------------------------------------------------------------------------------------------------------------------------------------------------------------------------------------------------------------------------------------------------------------------------------------------------------------------------------------------------------------------------------------------------------------------------------------------------------------------------------------------------------------------------------------------------------------------------------------------------------------------------------------------------------------------------------------------------------------------------------------------------------------------------------------------------------------------------------------------------------------------------------------------------------------------------------------------------------------------------------------------------------------------------------------------------------------------------------------------------------------------------------------------------------------------------------------------MSKAIEDDERRRRSLEAGREILEKYKAEKASKVQGTSYNQMDEISDEESFRNDKSIGHRESYVHEGVSSRDVTQSSVSMSEGEADGDLEGLAGRVAQLEELLQ-----------GKEAIVEALNAEIDHLRAEASSPNSSQSQNSSIPSRDVIPLYHTKLQEFEKAVNQRDNLIEELMWSLQQAISARDNFASQLNALNAMEIPCKDN------------------------TSSMNNKSLQEKIDTLEKTLNDQRSMIQKLNSQLAQNLEHVQTLEMERETRVAEINDYKLQINNLN----------------------EQIRVSAADKNLNITETLEQQKQYEARVDKIKQDMQHILKKFTTETN--INTTRHQQELKDLEENKILADRLNKELPDLDTRHAKELSIFQTQLTHYKKTVEALKLELMNRSESQQ---IAQTELNEYKSKFN----------------EFKVQSERARHILNLENQKEKEMLSEQIKLHKLQLEEITSKYIAVTAILESKESIERSLEQALSNAAMLKEENESLKFKLDDLSSRYSAAQSLIENSQTHEKTLSNRIYDLEKSLSRLSGINMSTLSELNETTYQTFDEVAVQYQLTKQKLEEKAELEKLLIQRIEGLEEDIHMKNVCDKYKSELISLKKNELDDQSSLLLEEELKLSNQNMKDTVNDLLHKIEEDQQEIKKFKIILEQKETEFAECIKKMYDLTDKLK-------------KSEQEREQLKNGLATAWAQCAEVEEKLNQTLALNDSKLDISVPSSSYNSALMKQYKLDRIVDDSVSMMHDQS------------------------------------LDKNKTLSEKNENLDSSNKRVSLQGKLTFVLEENERLRKELEHLN---QQTDYEEIKSKLKYYISLSENLTTEKEQKMEENRLRSEKETLRKEIETLIHVHDEQINAIKTETTSEIRKVQSLMLGLKEGTTELNDLKIELEKRHAKEMEELHNDSEIADITEDLYFGGAGDCLNVSNISEHNSRLGSPIGDEQSKYSSKNFNTYNK--SELEYEIKTLQQEFYCVIVCMQHCQTEWDAGALENGELTQLRAAYNHQLEE------QVALAKLDIINALQEQIQVALDLATIYNVLLTIESDVEDNWPIELLELRDKLTGNAKKEMQLLKNTHIEEVQHLKEEHSRTVTKMIDCHREELN--------------------------------------------------------------------------KIKSEYLQNYCGDRKEINNTLFTEITKKQLSDSVNNE-----------------------------------------------------------------KTMQFDETEGLKCNELKMSSKKDSLNLSEMIVRKVHFAPKTTEIVSIINSNVETLPTILEEDDNITEKLKQELNNCILRLKSESAEILNTSSIEGKLSSKDTFWLNKMNEELNLKLHHAETLI-MGYQEEIDHQKMTIFDLQRKLVNAENKKETITEGYGENY------DVGIDTTLQDFSQLQEKVRHVLSNGGGDCTELLQLIDELSRQSDKLMEEAKKEKEDLQQQVPLEPTPTPYIHRVCHRKVNYRLTNTIIKICSIEAADKQLKATRKFLDEQASEREAERDEAAKQIHILQEQLKE-R--EREKERDQRITSEQSTLSPEATSDIPTLQASDIDATVEVLESQMREMSSLMSDTEAKKNETESELKAAIDKIWVLREIITDLEQQLQIKTEKEESLQLQINQLETVIAAQT-------KNQHELVQELDAVKMGSESKQLNEHIIHLQEELRKHKLSSEQFNVNSAALKQMKTELR-DMQNQLDKRIKELESAHMCSSNLSLSQP---------------SEDVSIRDQIDATRCPTP-------------DDPTAPPMLPLDQLLKLKEKMLK-------HARAEEVAFKRIKDLELQVTALRNQNEELQAEQEILQQTASEQLFQIQSMRGRLEQHKQSAPFAQRQATSRLELQLHEAN-----------------TKFQSLERTIADKDLELKDMKNQLDRINQLLQEKQAEIANVVQVESVTIQKLKEHLEAKVGVQEHAQLELPKLIDSMLADKNEEIDHLKEQLSKKEKQLEMYSSLNLDETQLRELARQTEAKNSARTLSDILSIHSECEETAEAIRGINTTQNLPNVSTFKVPFTLKNIDDSIVPLMDSAKMIHPQVPPLDLG-SQSLSATSSQQSGMLDLLHSGLELKTSSSENNLSNDKTDHVTQSRQERQKSPEKHIDE-------------------KNDSNKSCVTSIKYTQTSINETLN---EMEKLENQLQIMKEELNT-KSNSKR-------------------------------------------------------------------------------------------------------------------------------------------------------------------------------------------------------------------------------------------------------------------------------------------------------------------------------------------------------------------------------------------------------------------------------  
-------------------------------------------------------------------------------------------------------------------------------------------------------------------------------------------------------------------------------------------------------------------------------------------------------------------------------------------------------------------------------------------------------------------------------------------------------------------------------------------------------------------------------------------------------------------------------------------------------------------------------------------------------------------------------------------------------------------------------------------------------------------------------------------------------------------------------------------------------------------MENVGKKKILEKTKEKIGNRKKGNEGKKSRTNSVSSNIESSTVDEKLDNIVVVGKSCSVLKPNSCLSESHSRAILHPPIISDNVINKTSGDTSDNLQPSLSCSSFISAKSDLSNSKLCSKEVFDLEDDNSAKLLPGKQTISNDSSKEKDYLVKLKEMEKKIYIKDRKINDLNQKIDECIMDKKKKLTE-NKDLTHKVGVLQRQLQSVFEMMKQKNMNPSINQDEFLNCKKKTNELHALNQELHREKETLKENLILVKNDLHEKEEELERKKQEINDCKNRCSLEMWKFINEIDCLKKSLNAQSQILNKKISEAQHSMDDEIVRITIKLEDQYQKDLKSAVQKLQGNFEKLSNLPVTTIEHLIQMNDIKNIDWIAFQKDLK----------QPENLSRGKSLSLSELNNHEITHSKNFYESKLALLKDQMNILSIRLKEKDENVLNADLIIDKRNLRKMYPTLPWDNIFSISDFYMAEIKSQNKIHKLERVFFENKVKTLTDEILHMAKNEEIYLEELGNVKKLTYLYREKYENLVNKWQERVEVNNEAEENLNFLSLKDIFEQLENFIFLLQKFEKIHGVNLEKIFAQHDSEILLCRESMEAFQNKILELEMFQKNQLILDNTIQELIEHVGNNYVKTVEALVTSHATELNLKQLEAYSNKEKYLDEIAVLKKNYQLEIQEHQNKLLLLEKSKNAVEKELDSFKQCQLHQNPSLLKMKLEETSLDLLKCEN-MLAMKDETELNLNNRIKELEEIINTKNINSQKEIENYLVKLLSNNIIISAKNDLIKELEDKLENSEKLLKKCCLDIEYAHEIFFNLEDRLQFLVEKFLYDERNFFRNNELFKIHSVIQSSRKELKDAFLQLFQKINFIEKKLNFNYENKADACVNTEENITDNDNCEKIGEDLNKGEGEENYEFQGSLKNEKNFFHDILIEETSNAENLSEDDLLDVFLFEKKNEVEENLVETYGVEENAEISENDLELMTEVVTNLELNDKSEKEEILSICFQKMEFLYKEENMTDE--GRNSPSFSPKSYNSSECLKYNGIIEENEKLKNENNKLLADSFLNKSHIRYLEELVSELTKNLNALFNENNEWICSTTILEKENEYLKNEVKREDYDTTGGDLRKEISKISKCIKMISIKLDNMKLNNERLINESRKNYKKNLSLTNELEEAWAQRYEIEDQLTNVRQENFLLNMHKAQLEDTNRRISSLVAEGK-------------PSVISWIKIHETVDNIVDEVLAAKTFVRDSDCSSASYKNLNLSDSQCKFNSVTESLHAHVENKTFLCDELLMQKTTLENKLKDLEELLKKNDAKINNRNKIISETLNMTENTDLIKKQIEKLQSVIEIKKNDFVENANIRKK-------ELEPSCFSEEDCEKEILFFFHEFVEEINGLKSLKIKLNEPDEVVDEITNYIEKIETINNCYKEKKLDIEPKFKKTRNPCEEKLKNATEKEEFEVFDRIKNILSNVELNEVELKEIIHYIIKKHVFKLSVLKNKNKEILNNHTSEIISLNIVLDDLKLNLEKTFREKENNTKKQLENMKIEYENKLKEEKTKYENDMRKIENDSERKIYDLKNLPKNFKNEKKEMKRKNFPIDQIMCDNEGDSCLHIKYIKNLESEISALKSEIAMANTPVCPSGSTQNNFFNLDITTDDDKLEIVRERDTLKNMVNSLRVLVGKLLNFIGQSGKNMGNDVTIRDFNYDDTMYKTLLLNSPGRN-----------------------------------NGDKSSLASNSVMYSLDISNIIQEINDHSLLNASYQIQNELDACVKEMKKDALNLLALSESE--DLKTLSKFLNKMEKDKDDLEDKIIKVEQVQNEIKSELDATKNKLVQWEKNIEVISEGYGEEIVGPKRIIPLSKADDKMMDGNESHEDVNELDEMHKDCHLHEEWKQEKIDLQQQIEVADKQLNSLKKFMEEQATEIEQERESFDKEINDLKECLKAKERGNLFNDQKKQEAYLLLNITNYLVFLTLTLSLIVSTL----DCNRIESLELQIKDLTAQINTSEENKKAVELEFQNAVKKIWSLREVIQDLENQLAEVNERENNLKKKIDNLNSRLEEQFKINKEIKNEFEVLKNENEMKYT----EHIKHLEEELVNLQQIVGKVNPESFAIMKSNLRHMETILMKKTKELEQVHISSANVSCSSPSEDVSIRDRFDATRCSTPDEMCCGNSSENITAPLEELDRLQEKLMRHTRAEEVALKYIQDLKMQIKMSKKSLEELQTERDVLLERSDFFLKENANLKARMEEQRCNAAFIQKQLNSELESHIHRLEIEVEKLTSLCSQKDNQLRDMSEVLENTSKTLKQREMEVMNKSREENVLISTLKMDLANALGDKEKLEESLEKIREKFKNSIKLPVLFDSMVEEKNAEIERLEKEVSRLSNEKSNLLSNLKTVAHSEFKCKKNFSRPDSFGEYISPISSGNRTEMRQCSDSYSDFKNSSFKLKQNESTFSNEEKEVKLLFLEESKLHREINLKNEEISKLKNEISSMENKCDQLQNQLDASQNQIKKINEDLDLSTKEIRDLKTKESKLIETLECIKKEN-LQKMEDLQNKLSFENAKVKKVYEEIKELEFKKIEIEEAFFNLKDDYCEKLNEEQKTVKTLMEKVRNLESKNCDTENKLIFVKNELKNLTKDSEANIKVLNKQVSDLKKENEMLKSEKKTVANAEVQVN---SNCSNLVYDSNLNKTLPKTFAGINLLNNNIKLLKQEFDKMSKSVSEMYQGEESVKYNEIKILFSSIIDNINELIEEFNAHTKKIMESSVDD--------------LAIKVKKELLLSAKLDHDLLTSFQNNDFAAAKNLGDEVSGVLPEYGGGDFPRVQSCPD-KSGGSSQELIVKKENEKIMKLEKEKQELLEKIHDHQKIIKFLEESLLAEKERILAEKERCKEDANIMELLRKNLEKGLRDIKAK-------------------EKDVKKERQLRMALEKELEALKKDAANVADNFNNKIANLKILVKSHEDKLEMLSKSLESEKIKNQELRILLE-QKKLEKISEKEAYKLLESDYNLLQNQKANISSEMNFTKKLLQSRNEEIKELKKKISLQDIDIKTLSVSKDLFSRNPSSDVVLDSDLKNIHVNSFPGNLNLQNLPEEVSESLLFKMKELNDCVRQHIQGGSD--ETNGVFINLNKENMGEQCSDNKYGDETIIPDDELEKKLLNLEKGK--------------LMMDLENLKSDYEEARRESIKINKSN------EILVCCCCCCLSSTDQ-SGQSFSKYLFEFSKQFFCFFVFPEYKFNQNCFFFDLFKTN---------------------------------------------------------------------------------------------------------------------------------------------------------------------------------------------------------------------------------------------------------------------------------------------------------------------------------------------------------------------------------------------  
-----------------------------------------------------------------------------------------------------------------------------------------------------------------------------------------------------------------------------------------------------------------------------------------------------------------------------------------------------------------------------------------------------------------------------------------------------------------------------------------------------------------------------------------------------------------------------------------------------------------------------------------------------------------------------------------------------------------------------------------------------------------------------------------------------------------------------------------------------------------------------------------------------------------------------------------------------------------------------------------------------------------------------------------------------------------------------------------------------------------------------------------------------------------------------------------------------------------------------------------------------------------------------------------------------------------------------------------------------------------------------------------------------------------------------------------------------------------------------------------------------------------------------------------------------------------------------------------------------------------------------------------------------------------------------------------------------------------------------------------------------------------------------------------------------------------------------------------------------------------------------------------------------------------------------------------------------------------------------------------------------------------------------------------------------------------------------------------------------------------------------------------------------------MELESVHDAYKKELERVCEKHQIELDENEQRHRNEIQRLCGENSSEISNLKLNLKSLFETELETIKNNHEREICELKEQFERRLNDLKDCDLDTLTKRIEDDITKTHAKHKKIVSYLVNEIYKLKKTIIS-------------SSFDDVDRFSS---IDSFDPKLFQNNLNGHDADIETSDYVLSQDSIDMQIKDLEDIVQERDDLRSVATTLR--QLSKYLCSFLLTK-----QEELNNSIVERMNGMECGGVLRGSGDRN------VIESTDNCEKPVNEIRSSRRVHFIPNIEEIVSLIDEHSVLADFTTEGTSEEHN--HLELNTCLKKLNEEAAALSDVINNEQNLLCTNSDDICHKRFFDKEIEYYKKELENQKSDYKKLFMETSALEQENISLKEQLE-----------KESSSVNDIKQDNDIDNDNLILLKKKAQELIKNYN----DWPNLQVLFDEFSAEYIKIIESIKNDKDD--------------------------------------------LAQQLVVADRRLKSSQKFIKDQIAEREVEREEFDN--KLETLKCQLKEREREKESLDTEDLR--SHTEQVVTEAEERYIKLENRCLRTEELLNTSKLEILSLKNIILNQETRLEHFIKNESSQKNSINQLTNMLEEEQNNQQDMLTELNRLKNLLEEQIQSDCHEFADIEIVINKIRLQLKDIEKTISKRIKDLDTFWVTEECGSPMSEDISVAERCELPSKISIEKNSSPARIVATLDDVFIR-------------------------------LQERLNRLFKSEDAIFKHESDQQNIVNKLNKQIKSLESEVDMLRTRLAEKSDQLTTAKMKIDELRVLKNDNVHLATSQLQEKLQK----------------------------------------------------------------------VTIENKQCSQVMEEQNHEIKTLKDSTKYFKNMLSAYET-----------------------------------------------------------------------KLAEKENIDPNTINNLESKVVHLAEENINLQIELANKISQIEALSTQINSLQIKELNQNAGNKQNYSVSKSIGTDMHISENEQMRRVIPNESNTLPSLQMSYHHQDVSIREPSIIN--LSLPSQITKENTNIHSSISEDLKSALANTKLELEEKN-------------ALIKVMEKDTERMDQQLKELKESIKMLNLEKIALEDHNHTLVKNETILTDKLNDALTQLDKKQETLAELETLGVKLRAEIQPLVYMKDILEKKISAIKDCNKLQLERIECLENMN-----EELLNECDHFK--KNQKDLEEIKSLQQDNWTLKNELLNTEKFKVEVAEKLEELKNEYDSLKSKNLALVDDIENLKLDKYRLKNELEKNEELHADELVGYQNLLAEEQNKKHQIN--------------------------MELLKTIEELTSQASEQQQLMSGIQENKELEVTCTFSDFGSENFTSNMSNQLKDNVKVLEIDLENERKKTTGYQIIIENK-------------DAEIEHLNAIKKCQEIKIADYMHRIQVESDTNTKLELTLAKLENHIKELENKIEIFKAKELHQTNDNLVEKLENDLVKVLDTNEMLDKELTRILDIKEETDQHLIEAYKKIAELETNKNQISLLLEQLESKNKIIEAFKHQ---------------------LDNAEIAMKDQQARSDKDKRSLELKLLDLKKELKESKQKYQEIVNDFEIMKNYVIKKEKNTEGIQLISEKNDNLVLKLDGAEKTATEWKKKYNDLLNENCEIKHKFES----LDAECSHLQNKITELQNKLTLSDHYKNRLKIDIQDITTPADLELKHSKLILNLETENVSLTRKLTETREKYNKCRAHLED---------------------------IKTMLTSQNLKNIVQENMELCNTRDKLQN---------------------EVMELKCCNQDLNRKLFEKK----------VENNWPVVGNQHITRVEALYHSLVW-QKMYLVKLARG-----------------------------------------------------------------------------------------------KD-------------KLLRFIIQEWPDLDN---------------------SIVKTMMQRICAKRKMSKFK----------------------------------------------------------------------------------------------------------------------------------------------------------------------------------------------------------------------------------------  
------------------------------------------------------------------------------------------------------------------------------------------------------------------------------------------------------------------------------------------------------------------------------------------------------------------------------------------------------------------------------------------------------------------------------------------------------------------------------------------------------------------------------------------------------------------------------------------------------------------------------------------------------------------------------------------------------------------MVPSTEEVDYLRECLKESTTLHSSGGTLVDSDLSQTPTHQQPQLPLQPHQLQPQLPHQLQPQLPQQQPPQQQSSQQPELDQQHQFIYPQTFDQHQCEQQNPSHQRQNPQDQQQQSLLTPDQMSDTFEPPQPQQPPQPQQPQQPQQPQQPHLPQQPQQPQQLQPQQPQKCLNCLAKDEQLKIQSDMILHLKSKLESYEKEPSNIVAVVANDTDLNLKVKLEELNDVIR---------------------------VRDLEIDRLNALVNELNSKIARIKNNLLNNKFKPSRGGEVDDDGGEDGGDNDGYGVSYDSQIKEYAQRLSIIEESDNDDDSGDDNADDDVNNGDDNDVNND-----DDNDVNNDDDDDNNDDDDNVVNNLIVFDMGDEVGDDDDDKNSDRDTGKKKVNDVVDDIADGDRDDDRDDDRDDDRDDDRDNDRDDDRDDDRDNDRDDDRDDDRDDDNPEINDDKIAKNNVDDGDGDKNDDDLDDNSGKNLSVLEEILSVHG--------------------------NDLLTDKLSDEQDPTITSDDDDDEDGDAICDIDEDDDDDGDDRKTDASRGVTEFGNSRDNADDGDDDDGGDIANDDKDDDNKENKDDINVSYKKRVSVELIKLSEENNLKMQRLMDKTDYTHAHD-ENDEDDDDDDDDVGDDDDDDNDESKKVEDDENTNNNNNAKSNSDDGEDNDDDEDDDDDDDGEEEPFLKS-------DRWERLFEDHTSKAAAHTNMSLDFKDEDDDDDVTNDMSHADNEVDSGDLITTNNNVANEVDLMITNDLVEIGNNDDDKGDENDNDDDDDDEASVLSKHILDQLINDVTDSVVIKEDDDDEDDVA--SNIITVDDDGGHCKNDKKDGVLSDDFSGDDVAIATARDEIDDDEGDDAKKRNARFLYDEALSLDDDDGEVDDENTNDGEGDVDDEEEDDDEEEGNDIDDDNSKTLDEKFFKEYKADEDDDVKPPIRDDVIGDNHITKYTEMVAEAVEITSLSSVISSVLIGYDLKITPDNIDLIKEQVKTIAKHIISHDNAERMKLNDDEEEKEEDEDKTKITKNDNDNGDKVVTKTDHFLAHDNKYITSRRDINDEADDSDDDDDRGNKEKLRKSLKLVDELQKEIKELKERLNESSDVILAGDNDAAA--------TATTTTTAAAATAATAATAATTTTTTNYNDDDNADDDGDEIKKLKTSLSSPLDRRVFEDEMRKKIEMIDILQEKVKGQEKLIMTLTDNNVNSNNDDNIDADDISIN----DELTATNNHIISTSALDDDNINNINDNVNLTMPDGDNNNDMNENNTNNNTNDDVIDDIINSKDSIIVDDDENVNVTH---------------------ANNSDSISKQPFNDTLDLMNDTKLKKESLTVTNELLHRILSES-VRVSLESEKTVAEFFNRYKIFTKSINNYNNNNNNNNTNTNNNNNNNNSSNSNDDDGSETRVEDLNNDNSKINNGNGSVATENYQLQQQ----------------RFSCLPSQQQGCSEFESFIRQSLDGNFSEAGSLLSILYDDYNNKNNNDDDINNSTSGKSHQQPPADLTER--LINSILSDRCCNGDSDDGHVHGDHVADEDDDDDGDVTSHHKCGEVLTNSVLRLQTAITYMIGYIEECDSKYQQTAQQLEKLIEDLDVAKAEKEHLNVNLSMKGRT-LIDGLTLEKLALERTIAALEEKHEKLVRSSELVEIELESYKRCHEDLELKQKELEKNREIILNETADFATQELLRENMKLSDD------------CRDMSRRLSRDKQLFEMQLKELEAANEQLSKQVEKLKEEGEVAARDAKMRMDALEKQLKSDKKFIEARDQTNER-----------------------------------EEEAELFQRRIKMMEDALRDSEKLSSNHQRMKKEIES--LEEQLKEKIDGYAN-VLNLKEVVELDLNQQIGTVAELRECIRALENELSEKCRREQELSLQLEEMKIEKERYQSNVNAGNKHSPADDSDDDDVNDGDASKDDEIHHLKEIIDSLEKELKTS-KAAEISLKAEMGKVKEDLKIVESKMQDLKMDHEILGKQSVEQLIQLSNLKHLAEERCLDKYGRALASAGGGGGVSNNVVVNGGSGDLDLKKMIEEKDKQINDLTSRLTDTTQKHVHDKEMMDRLMDEVNEQKLNHTRMTELNKKLSDQTVILKFKNEELSKTIDTLKLATTSSTATTTTKSSPNESSMMLLNEALIDEKNDCID--------ELSAEVESLRSQSRCRSLQTTTTSSATTANNINNSAALSYNTPEILRKSDNSTSVSQLQHIGISHHLGFSTDNLGISKDGIAFSADKFSQVHFENSLKAKAMMDDNNEETVLMVVDDDDDGGDEQIVGRRRNEGRSGIGENGMVEDLGEMLSPINDDYT--HNSDMPITQEDLSRYNVNTFTELANMIEDKESTIKRLNQQLDQQISENQ--SLNLVIDELKLKLEQTISSSDLELRKLKDMLKVKETELNDVIERLSHVVDQRTSDELDATRLSVAQLEHVIEVKDDQIRSLTDGVERLRRELDESTLNVEKLSTTITTKNLDLLNQEEINQKLQQVNKDLLQKNDNLTSHIETLENSVNNTEKLLENSQKLLNDLQKRHETSTDLLRNKRTELEQMKDTCNQILAERNDLKIKLDNLEVKLAGVEQQSKENVENVIQQNAERLQQMNASLISKENELQTYLTQIEHLREQLLESADKYDDVCLSLDRLGSDRKTEIEALKLIHESNVMELSETHEQQIDKFMQQVQDLQQQLQHLQQQLQHQQQLQQQQQQQQQQQQQQLQKNKSSTNIRDVVDAEEEA--LLSVLSHNNTLTTANTTTTTTFDELLSEDLLSILRKLHSEAMRVLSLSDCQFLESLEKKSIEMEELKERWMTEMERMKEEKKVLEERLKIEEE-------------VSTLKMERLSRKYKDARTLVEKLKADLNGCKTVAQVSIAPSQQQQQWQQQTFQQER---DKLVEERNRLQDDLVNLKASIDVERSQNLEFGKKIERLLEDRTKMKSDMKKMFDLLEVYESEKSNLTQELASLQQQLSNSNQ------------------QKDSLQKQLDDIKVKLDNIEQQIKQADRKTSYSAAVASAAAPSSLMSSDKLFLMKQLELLRQKLQLYQILTRDKMFVCSTLFGVALGVQMSMECAGLLNDVLTDFTRTINN----LELTSTAAVANYDIEMLNDKILLHNNQLTQHIQRFCNTNSNDVGDINNNIGNVNNNIGNINNDVIGGNSNTLHLNKHPQEAHGNMNTDNRMLIDENDDDDDDDGVVMTSFARHSDVGLKVNSNAYNELHGPVVADRK------------------KQTSLHLDYGADVEQHTQLNNNE--IHMRVTLTQQIQRLYGKY-L-RSESHRKSLIF-QKNYLLTII-SGYKHAELDTINSIHHITNLARHHHQQHPHHRHHSNFNIASNGDISNSSVCSLVGSDPGDVDGVMAVDLNVNDSGINRTLIRCRRSWVRGQSLSLMSKFRSAAWAVVAI--LRLKFLVLKWQKATSWTR------------------PMIATSQTTSQPTTTSSAAGNLKHQYSLQYLPQQHQQPRHQQQQQQSLHQRQSQSTKSLSHEVRSARSLTTLNNNNNNNINNNRQQRKQLQQQFQHTLPTSSYGYISDININDNNNNNTNNNINVNNNNDDINDVVTYSNMNRRTIQATPPTKEKASAKRKTSNCSINNNNYINANNNYMTILMKRDQFHEDQV----------------------------------------------------------  
----------------------------------------------------------------------------------------------------------------------------------------------------------------------------------------------------------------------------------------------------------------------------------------------------------------------------------------------------------------------------------------------------------------------------------------------------------------------------------------------------------------------------------------------------------------------------------------------------------------------------------------------------------------------------------------------------------------------------------------------------------------------------------------------------------------------------------------------------------------------------------------------------------------------------------------------------------------------------------------------------------------------------------------------------------------------------------------------------------------------------------------------------------------------------------------------------------------------------------------------------------------------------------------------------------------------------------------------------------------------------------------------------------------------------------------------------------------------------------------------------------------------------------------------------------------------------------------------------------------------------------------------------------------------------------------------------------------------------------------------------------------------------------------------------------------------------------------------------------------------------------------------------------------------------------------------------------------------------------------------------------------------------------------------------MIDEFIFIGTNFSQVKNVLSHSVCLQTEPNLTMNDDDQIKNYTVCLEFYHKMILSIIDASTKLKHELTAHINDQNSWMN--------------------NDLLLDYAMKHSEQDRENELKVQLLNDYLNECDHLL--VTNVISDTKYLDHFRTDCKSLTSIFGQLLPEIDRLLTLCKIGCEKLIRSTDTSRLNQVDVDIENCELVRNLREQLLTAH---QLLTEKLPVETKIA------------LLETSQIVETER--IQLQAEMDRRLSEFERNHTASLVELETSRHDLMQQLDEMELINRELKNEIFSVQQKLQ--AKEKFLNEQTEER--ELEREEFRIELNRLKSELEMKKSQMMSYKLLD--------TSDTDSCTTPLNIKETGQNNFVPHWISDLKMGHLCQNDTIKSNIQTLVQS--KSWDIVSWNKPNDSDEDMISGNINNKRSQNQYKYVDQQNDWQPYTIPTISEHSNFVDSVTNTSGNYDIQNYPDEIDTKQVK-------------------VIDDSNAYIQQHQVNLCDACTQVEVITF-----DKFVEATIDSVDMEVQVAIDD--------------------------------------------------------EVKNLETMNSHEQLNLVQSSPKDNTNHSDNTRYSNQPTRTTKTITRSRTWNTTSAAAVLSHIVVKEEEEESIVTVVDESDTRNDSKINNNNNNQKFYQDQIEKCKLEISNLRKQLDRINTCHKEMEQTKQSLEFMQHKQDKGIQTMVFCQNLS----NDIKLKTSLTHHMSSSIHRMHHHSLPALFSPSFSSNNEDTGVEMSQGLLDLESIESISPPTAKSPIIQDLILMGHEKCSSPEDENLQTLMGSPIS-------------DKDDELENEIPEIMLSSEHLDTTPSLCVSENACCKTDSVDSDPQTVCAISNDDVENPGAVVPLSEVSTLIEQLMDSEVLIKTLRNEISG--------------------------------------------------------------------------LTKYQIELQHDYDAVHEMLVERQNDLTR--------------------------------------------------------VTEQLLETENKCK--SLSKQLLERKDVILERDEDLFLLSEDKKSLEIKVTQLELEIEELKTQVKYIDRDKLMSSAFVQTDLMNSRSLFSTKIVQTDDIPISLESQPSVYSDPSKNVNLNILYDRKNFHPNIEQEHIHQIWATSTPKEEESPNSTFLSSENDTLSGAVSSELNN-------------------LSNRLREESVRLATATAIATARQSICDRPGSILINKTNDNE--ETTIKVECNECAPWIIEIRNSYGDLKEAVNEVAKIIHNNPWINEDQYKLEDSEETMRMFMNRLLVSLTKALDTDEKLWISVITS-------SINQTCDILRSRTSSVPNLALQLPTENILCQIIQHLTERVSAFMRREDEFRKCLIDVLHVEEASFKSELKTHVNRSDALVGEMNRLTKVVSSLSEELNHANNRTN----------------EVQGQLHDLQVDLVHTQNELQLKEDEVQRQRTNVEQLRLQLCEENTQTERFRTELETLQSAKVQAENELS--------------------------SSNNLIQELKISLTNEKNRAQSLKIELDQLYDQIRKNTYQTTPTPNNNNNNAIG---------GNMTTTNENKAPNDCSSNLNPRVYEAINLATTQLASTRRDTIKLQNQVKELQATAHSLRLNIAEAELRLMP----------------------------TLTSVLPGTFNLSSINNTSQSSMIKQSAAKKKSLVYGDQ-------------------------------------SALTSAKFTKLKNVCVDLLSRVGVDERDDDDLQKGDDNDDHNSDSHSSDDDSIEENIFIG------------------------------SGIINSLNRDGGYRIDAERDESSLNNGNNNCDILNKGSCRSNKATNRRSTDTLNNSLQSTSNSHVKTVNVLNSNKIHNSVQT--------------------FPIMTTNVISSSPNAHINQTVS---------MEQYFSLYVRF-L-RAQSYRRSLSF-QKHYLLLLL-GNFQYAENVVVAILGCPES-------RVTSQTDSKDRSSQVNLSP--------------------------------------------------LRRFRTIGRVVQVI--HRMKHLVNKWRRLDIPSM------------------ESTPINNKILYMHTNTPTSYYAPPFQHRSFSSTLDRSNQTNRIPNGSLRTPLKELHAEESNNIYSSALQNSSLFNGKQYLSN-------------YPVHTPRLSHIQTSPSGYSSLLENQTISTGSSHRRYVNFC---------------------------------------------------------------------------------------------------------------------  
ICSRYLRAELFSKGYLRAELFSKGYLRAELFSKGYLRAELFSKGYLRAELFSKGYLRAELFSKGYLRAELFSKGYLRAELFSKGYLRAELFSKGYLRAELFSKGYLRAELFSKGFSVAILNDCANKNLQNNTIFSNYFDELFDKTVICVRLFLDPSIMDGNMKSSSSSISDHSNNKKSAEDTISTSSTDPAPFRYVFKHEEDELDVHRNLENTLEEDITTPSEVSQELNFDDSFPITNNSASKVTVSPLYENQSFVLSSPSKNDSKYNYEMNKMTDDNPSKTQMLTAKTKGKPLNLASEDQQNEDSYHQSSPDIEKMVTELKLQITKKDSEIERLYVIEKNFKQNKQILKDTTWALRVKEEELLSIKTEMRENYEKRLRELMQRLNDQEYQMGELQSQNNELIEKENDSILRVDELNQQIYLLTQELEMKN-KLAHVIEKEKELTAGENKSQIKILSQQVQELSFLLEKSEKNYGKESLRLQELELQIENFQTNTAHLETLVEQKDSQLKLLEESKEVIVQNLQDELFKSKSQIKELVLLLEGASTNSDIIPTRQIDETDFNVKHKKLSNFETDGDSFGSVDDIQSEDLLSHIDNDSVKQMNHTIGKYEQQIIDLQNLYDHDISQLFAEKEEVLKELQELKVVLNKEKQSKETSSFEHGQLQDGYEAEIEIMKKNFESTLDQQKTSLSKTISQLQNENEKLQLLLHQVQKKYDDAVAGLEIAQEQTNLTADKYSIDHDSVAEIEQLLQQQINEIKSQYDKQLTSLETDFHREKQIKNNELENNFSSQF-FLLQEENIGLTKKISSLESELDVVQQEYDDLKISLLFNNGINDRETASDNDVLAKSSALHLELIRLRENFNDAKVEVENLRSERNKFASLSDALSQNLQELQIELEFNNNKSLLPDLNSTIKEVDMLRHENSLLVHKLNLSTPLGSLPGYSSEHMIEEVTLQPLLIESKPIDSGYFNNIKEIDNEQEEEITIFDQIENLNFIISEKTEHEKELLQEIEKLNCQIESYQSSSEENKIIELQSQLERSEQLSNHLQEMFSIQLMKLQEENKQLTNDILVFKQKLEKQKKLSEEKKQLELLLESEKVLASESSERLQSLQQKLQEEQAAFMNLNNDLSNANKEIEIERQNNKKALSENMQYQLELAETKEELIKSNNELIKLKATINQKNDKIDQLTEEIKTINLAFKEREDAMKLEFEDTLRSSKEAMSVTNSECLSRLRSELDIDHQQRLKASEDMLHDEYTKRIKALSEIKEAELVNLKSEYEAQLSAFTTSFTAEKEKQIRTLKTVLERNHLKEIQELLVQHDNKIEALQKQYSEETEKLVAKLHSNYTDKITELETALGAA-RVEQFEALKLENNKLIEKELEMLSNSLNEKHSNEIISLEHQHKKEMDDIATKHKKEIDDIATTHKKEMDNIAARYEKEYEIRLSQKTEELVLNYQNQLNFEKKNLNLFFETKLSSELEKAALELNNKHLQSLKEYDSDLQIKHQANINKILLDIQVEHDNYVKARVDEITSQNNMNIQTLEENLASSLAKYKFEMDEKMNDLKTDHSKTLMDLTNFEEKIKTLQLLYDELRLGKDMEILSLTEQVEERYGSSNVQQILLAGNLSVDKKKVEDELRLKIDKMKQKNEFKLNEIKNLHEQNLEHALKDQEITLNKAHENQINNLVTNHNQEIEQLMSAQKEEILTLGKKKELNIIGSLAEMHTKLVLKHAVEIEKFNFDAKNLLDKTTCKYQNQINEMLSTVELLKAENNKLISQQLTVKLDQNTMTEEDLIVVKLKLEITNLQLQLLEATSQFQKDKLEDAKKIKNVFKKTQKDFEIARVSYKQEIDKLKLEYDWQIKKVGKEIICLLKDQLKVISNEKEKEFEMARDDLIEQQQNLLNEFVKHQEYEIAHLQHDHDHEVQELKSKLQAAEEAMSKVAAVITTDISTCTDEDRPHDEEFSMKIAEVERTLTAKFQ------MDNAVIEEKYKNQLFEVELKQISDLREVEESLLNKHLKNLTELKEEMEHKHSQKIQELERIVEEKVEEQHQLLLESQKHHALDIDNDDIGWDEDDEAGSDEDDETGSDDIEQLTLKLIAQEKQAQDLKKENEEYHETIHKKHQVDLMQLRESLEDEHQKERINLENSFDEKIRTENDEIMIIQERFNIEKKQLLDELIKKDNLIKQLHEDQTEKY---ELEHRKIEEKIKLLE------------KDIVHLEREKLQLRQTIEVKGKEIDQLNANVKELISKNELMFERHDRQEQESSNLVAILQNDVNKLTKERLGAAPSQVVAITFNLHSSSHEATAFQNDGKILVDNIPSVAKLASKSTNTTLALQNDITNVEEVSIDPDHAVHSLVPDEEESIIDHADFKVNIQDPEELKEIGERLFMV--VDHLLDMFSSTVNELSDIRIVCSETQNNVNVLSHDLEIKKDQLLNLENENKHITQQIYDYDSQVKDLKLNLSKLNEQNNQAQIELAATIEKNNLIQTDHKNLMEYNSLLENQLKQVQTELNDSDNRVEESSKVILSVQQNNQKLEKESQECKEKLKEKELELILVCEK----------------------------------LTSLQLENKLAETKLSDLQAENKQLMDE-KNAKEIKFANLKMELENE-RYSRKQEEFAFSKKATKAAFVIDEEKENLKQEIESLKEIIKEIMEEKQELRSQMLDSGYSHKEDSLRSDMTKVMNENDELILNNQKLLQQVCQLEGALQETLYEKQKIESTLNEMYQVQLSTKVIKQNQGKSIDFNVEANAIKTPIKDIPSSIVGSFQKGIQSTAVSEQKMPSFTHGSPIPMHQLNHNLNSVRFADEIQTIRPDSSVLTNEDFHSLTNEDVQSLNQMCELVNNINNNLCS-NEGMPLMELTIEPPKNEVVMRLKEMISELFKKISDLSVNHARMAQLSLQQKEDIEKFSDLVSQLSKEKKDLEEIA-------------LVLQDENKRTVENLMEEQATQNATLIKDLEAIIRERDQEIKAASLKHNDFHQQRELYNQQRVYVAELEACLKETQFVLSSKNEELKLMKSWSNEKRIEEEMKSCSDKKSDEEQKPSDVKENVFEMQNLFSNSQLVNETKNDSFWHECTDNQMVNKT--VHQRLMNLLKHQQQLQDETIKNLTDKDVKLLVNDIQNEAEEVLNLVMSIPELNEALSSQNMQHLQNAWLIEKNCLLAVINSLKDLVVKVSSKNAASGKYDGDRRLELVNALA-DLFEKEQEAIS-VECKINGKDKEKQFDNEISPLLTTLLSKDREMLVDEVKRLRS---VTSADNSFVLLNKQIDELKCEITRLNTQLDYLNSNLEAEKLAKKQLSLENSDKFDTLKVLTTQLLQYQQSNNEARLLLEEEVAKSNHLRKLLEDAESNVVSLHDLVKSERESLN-----NLLEQDSCSVNELEKML--DNEMEKRALCEEKLANLKVLLENEKLKVQAFETELNNQRENRFNSEKLVQTQKLKIAELEKQSCELETKLLQNSDHRKSFQKSNDLSESLQNDSGNFNNPKLIKDDYTDNLLKDKILSLEKININLQEQLERGSTTLNTYEKKIDLLKKKLSENESILSVKEAQLEKSYIESSQNNIKFDKIIKLNEFEVRKLNDEITQLRKSEEYFRLALSALEKDKCLVLEKANVDLSSQLESNVFTLNTHEKHIELLKNKLTESESVLSEKEAELEKSYLELQKYKNKLTKSESVLSEKEAELEKSYLELQKYKNKLTESESVLSEKEAELEKSYLELQKYKNKLTKSESVLSEKEAELEKSYLELQK-------------------YKNKLTESESILSVKNAEIKKSYVELHQQRNEFDKIKISNESEVRKLEDIIRQLRKSEDKYNLVSGALEKEWSTKNNALSLRENLLNEKEAELEKLILELEKENNLLLEK-RKHQENTEQESIKKKSEIDRLAEELKEKMNKNSYEINA-DKSSQDFINLERDQLLENIHRLEHKVAFYRQQDVKFQELRDFWEKERDHMRAQFEQIKLQRDKFKEKGHKLSAKIIQLKEKMFFLQKTTDENNQMHLIENNKL--------------QDEITILKERLASAPLEVAGDGLKLL---NAGWTIAKVQRLYLKY-F-RAESFRKALSY-QKTYLLLLL-SEYGNSNQSLVRKMVR----------------------ENSSTPY---------------------------------------------------KKFKVTALVIVSI--ARMKFLKRKYQRSLYKSS------------------ADYPYKDVREKKHDHKDNNQSKLLLDKKFKPEKVSHSGTISSPSYTRTNPVTIDSDKQYFNKTSSDEKMITSQSLNYKSDSHDHTLSKYSVTRLEERPKQSTTDDLESSIENNNLLKSPSNISNTSPCSDSNASNTPRFVEVSVNSIKSSILNQSRTSSGLKHYSDAPRSYPRPSPTQSDFVFRHASPPVRDVDDGNDGKNIYSPQTISINKEFADVYTRGNRDSSTSLNHYIQKLESLQVRLKEQHTQFDK  
-----------------------------------------------------------------------------------------------------------------------------------------------------------------------------------------------------------------------------------------------------------------------------------------------------------------------------------------------------------------------------------------------------------------------------------------------------------------------------------------------------------------------------------------------------------------------------------------------------------------------------------------------------------------------------------------------------------------------------------------------------------------------------------------------------------------------------------------------------------------------------------------------------------------------------------------------------------------------------------------------------------------------------------------------------------------------------------------------------------------------------------------------------------------------------------------------------------------------------------------------------------------------------------------------------------------------------------------------------------------------------------------------------------------------------------------------------------------------------------------------------------------------------------------------------------------------------------------------------------------------------------------------------------------------------------------------------------------------------------------------------------------------------------------------------------------------------------------------------------------------------------------------------------------------------------------------------------------------------------------------------------------------------------------------------------------------------------------------------------------------------------------------------------------------------------------------------------------------------------------------------------------------------------------------------------------------------------------------------------------------------------------------------------------------------------------------------------------------------------------------------------------------------------------------------------------------------------------------------------------------------------------------------------------------------------------------------------------------------------------------------------------------------------------------------------------------------------------------------------------------------------------------------------------------------------------------------------------------------------------------------------------------------------------------------------------------------------------------------------------------------------------------------------------------------------------------------------------------------------------------------------------------------------------------------------------------------------------------------------------------------------------------------------------------------------------------------------------------------------------------------------------------------------------------------------------------------------------------------------------------------------------------------------------------------------------------------------------------------------------------------------------------------------------------------------------------------------------------------------------------------------------------------------------------------------------------------------------------------------------------------------------------------------------------------------------------------------------------------------------------------------------------------------------------------------------------------------------AAE-RAQWAQEKAESQNTIRAANE-------------------EIARLKEDARKAGTERSSDSQATW------PQAKVIQKLYSRY-V-RAESFRKGLVY-QKRYLLLLL-GGFQECEQTTLAMISRMG--------AYPSY---PDVPKRRPPAY---------------------------------------------------TRFRSAVRTVIAI--KRMRFLVAKAQRTS----------------------------------------------------------------------------------------------------------------------------------------------------------------------------------------------------------------------------------------------------------------------------------  
----------------------------------------------------------------------------------------------------------------------------------------------------------------------------------------------------------------------------------------------------------------------------------------------------------------------------------------------------------------------------------------------------------------------------------------------------------------------------------------------------------------------------------------------------------------------------------------------------------------------------------------------------------------------------------------------------------------------------------------------------------------------------MESREQQLERTKQKLQRFRNSRAPGAYSVHSAISSSSTVHGQPLSGLGLVSKSSNGSLKRTDAHSVTSDIEVQPASSIAS-----SSEMSDTRRQIMNLPSTVSLKSLSRQTSRSQLRTDLDHDFTSNYSECESKISATSMVGIDLETDPNLAEIDRLVLYDGANDSIS---------------LSITSNARSSSIRADSPSTQSVMKSKQDILVRSASVSGRRDSIQSSIHESQRLHSQEEQLQKIHGQYISRIEKLEAELDEYRSKYDTELNTLGRHNHNLKAALQTAEIRSAELDMQFERDYGEVEENRMRTRLLEEDNAMLNQQREMLEMQLENALSVNKPISSLVPDNNLSEVELLQNQLLEYQSMTETLKQGAHEAHLQITELISAKHVLEETVESLKGDFDLMPHVDSDSLDTCSKEVVQLQDIITKHKEHVTLLKDQIADLETCLLNKTN---------------------SHRELELEIERLTSLLAESTEALKQVEARLVEVESNSLLTIDGFMQQISSLQTELAAIKTLVHPSASTNVESGIIGEFFNCLDETTGAKDEISQLQAQLHDALLGQKDFSDRLEAANEQRALLQIQLDGMNLDLSHSGADFSSQTQQLHEKIEELSAENSRLQSEIDALYSKTTTESTNAIADSSDVEQYRMLTEHMSQLVETTATSEQEHLLKIENLQNTIEWVQEEKQTLTADFEQQIQVLQCELSTISAERDALIANAKHTNTSYGNTAIDKDESFAR--AMELELEVETLKTDLQSVRDENDTLKRLSQKHNTATTSSKTIQLSPMTKLAEVTQPDILSAQDTDGWDEFPLLSPEVDDLKVIIDDLKTQLQTITIERDHLASCTITTGSASSSGAYDMSSLQNELIAITTESDNLKSKANAARLELETFRKQSIDDQEQLVDTMRNLTERNSEYQILLAELQAKNDALLKRTSTLDTLDASVLTDFSPDFEQLSEALKKITELEAKIASMEADYSDTLVQCDDKIQQLQDQLCAAQSKVVESATELANSSDLINEKKTSDAQLADIEVRLADQTVFAEKLTQKIVEMQDELNYLQDEKHYFEERCDELQSIIESNNVKFENELAK-----LEAAHSNTSNQLDEVGIERKSENESTALHIETINMQLSSLKQERDQLAALLEQAQKDALASRSELDDAIHASQSSSALSEEVERLKEYILVLEDKIATLENELDIMRTSS-QQWEDYAATSLEETNELHAQELACRDENESALTHKIEHLSAQLLQAQQSFSNTEDLVSANEDLTSKNNTLETTLEDLR-------------------------HATELEVLSLTDKLRDVNVKFIEAQDQSSKMESQLKETSSRYDELAHTHDQIKFEKEAFERDVLSLKENIKQLEASISNNESRTSHISQEAEALKHNYENIVAIRDDLQLQLEAADLRSQNLEQQNMEHR-------------SHIESICTSQEALIAERDTLTQQIQQLSQSLDAAKLDIVRLNDFCANVESDKSAFGDVQQRLDEHVLLVQQLQADKETLVNTLDEMR--ASQDQLHVDLDALKTQKNELDITCAENDQY-------VSELTLQIQDAQQAVNEWQAYAQTFETEKSEWESRRIEFEDRLANISNERSSE-MDIIIQELDQVKNEKEQLMAVHNEQSHELATLAHEHSNLSKRMAEIELERSEMEYEIQNMHKSTDASKATQIELEKLQEES--------------RQLRFSMEQSEQHYQQQLEKNAFDFDSLQERIMQLTQEQAELQQEKNALQEELNVLLESQTKSREMEEHYHELK----------------------------------LTNAQIQQEYQQVLLQLQTTDSQLTRFSELEQQLATKTEEIDHSSKEISQLSQELELCKLDHLNAVKSLAERDHELFAVQEKYVQMEHSLEQAQSHKKAEVSDLQYQLSQLSAKVPELESHVTYYLEKIALMEDAAHASNEKLQLAQEQYAALEKELYDCKHELHDNHEAHCVEMDALKASHEDHKISLNANYDEIYSQLTYQAN-EVQRLTMLLEEAQHNVAMAQEQHRDVESELLSIKHVHSEAIGQLNTNLSDQVAEFSTLTAKAQQLEAENTLLKQSVEKYVQEITQLQDSVTKVQDAYDLVKKNLGETELRLSELKAANLLSIDKSTNADIQTKQLQQNIDDLTKKLADAKQSKSE-------------VVVKLEEMNDILLR---------------------------KDGQLETAQAATAAVNAQLASLLQQHKIDLEMHRSNCVQSDTSEQLRYKALEAELADTKNVLAITQNKLLLARPFTNPEQALADGITNLSMTGMHDRGNFSSAELQSLRAEMAQLTSEKQALISALDARSEHHGTKHTSAAVEVIRLQTVVAELQGKLAESDHLLSALQPALDELDSKNREVEKLSHLVRSLGVKMDAN---GTALVLMQDESPKIPAGMRQVSIADHALLEAKACNVDQSMNQLESALALIEENDGEIKTLRNTIEELLNNSFAGMSETPDG-------PDLKLLQRQIDELRKLWSHELSANSILRNLIAKAQADAHQYQQESQCKIASMKEEFDELAHVCEIAQRDIDVVQHEAKTHELNARSIEKQFEDRLNTQYFEHHNQIAVQDELHTKERAALNKLVGSLEAERDR----LVSELARLKSRQQSHSTDSVRDSDFFKQRLAEKEAMLLEAESNLRQVEIKVRDAEFHIRDCEANIKKMDQVHKDVVADLDSRLQHLNLHSQLLETKLKDSEFALAKALADA-----ARASQEAAYFSDRYRSMEDTYKQVPGTHHLQRTQEDHESHRHAWLSERAQLETELRKKHMEILDRDQILMEERQRMRAIADEEIRSAVA---------------AEQRHSRDLLNARIGELR------SKYDTALDELRAQKRKVEEQLDDTEHMLFE-----ERDSLQRRLFDTEERERVQS--AEVIILQERLTRRDKLQSDRQTTESLTERAEKWHCKSLELEMRITQLEQEFDTERRDLEKR-----------------LNDAVVQSRLADQRLTDRDVLWTNERRGFEQEIDRVRQDAAKLASQLRAEDTNGLLKSLQEQKNEIETLLRDREARLIDLETRLQDQIARNTETTENWHRVVAQSQVMESR-LIERDAQLREAQTKVHSLTLQLSQFENKDIVSPIVSEQHHSQMEEAKRQADAQIKFERE---RAKRLALKIEDLKNRNQQLKRQVETHSQPMQLNSQAAAEEIESMRHELSNLRISNGEVLAILREMLINTIGPGAIDIP------------------DLEVNKIRLNLPKLREQCR-------------SLVQELVYVRALVNRLVLWRGDLRY-QKLYLSFKV-DDLLESQKMSLSYLHGMG-----------VIMDKPDFTKSLTPR----------------------------------------------------RKLIRCINAVIAV--HRIKIMAARWQTFLEGNGSFSLDEYHGAEIGYNQTGLVAVSTKNQFSRQWSYGELSSDAQNQHVKDLERNLQQARMGQAAIEERLNEELYEKRAIEMENERLLRALKYDRHNLSHISSPMHFIQDRMSPAPPSAAGSVTNSLYREEQGRHSQHIPHGMMDDRQFTSQLNGSTYPTDQRWSQQPTRFR-----------------------------------------------------------------------------------------------------  
-----------------------------------------------------------------------------------------------------------------------------------------------------------------------------------------------------------------------------------------------------------------------------------------------------------------------------------------------------------------------------------------------------------------------------------------------------------------------------------------------------------------------------------------------------------------------------------------------------------------------------------------------------------------------------------------------------------------------------------------------------------------------------------------------------------------------------------------------------------------------------------------------------------------------------------------------------------------------------------------------------------------------------------------------------------------------------------------------------------------------------------------------------------------------------------------------------------------------------------------------------------------------------------------------------------------------------------------------------------------------------------------------------------------------------------------------------------------------------------------------------------------------------------------------------------------------MYTSSMREQKLAAAKKKLAKYQKQRAAGLPRSTSATPSETAGSPPDSRVVSPESSVENLHADAGGRPRTGSMLSTSSIAADERSASPLGTLTGLRNRTDPPRSGSPVPTFPPNVQRIATPPPPAEPDLIQRVRELASDNQHLARRMRELSNENRELKEKMKQFEVHGVPQEKAEVAADMDGRIAELTAHLEEARSAATEAEAERTELSRELQQLRTRLDETDTLAQQLEALKEENENLRQGRTEQQQHSDEALEMIQQMREDAEIFRKEKLAG-ESKIKQLELELSQLQSAEVPGSVDHDSAQLNADLEMTRSQLDSAQNTIQQQATELEEVRGELERMQAVAG------------QGAATSSEEQTGVIEDLRNQLANAKETTKEQASEITEIRQQLAVLEKAHGDLATENELLASKAATAASSGPSTVPDADLVLERDELTSQVQKLKEELQAIT-----EEYEQCKAEVTTLRDENATLMADRDAHASQMEELSSTQGELQMQVQTLAQDNEAIRRQHVTVVGNMRKLLEDMSGRS----------------------------AAATREKEAMTIRVRELEEEVKRVRNEYPTLLSANAEAESKLQQLESDVEYLRTEREQLLTEMDLMKENIKERELRAAEDTNTLVETMRDLTDQVADLKKQITQLTKEKADAVLEKDALERKVEELS--------------SVDATHKEEL---------HRSQSMMMDELNERQALISDLQSQLDVVRNDATAELAEKARQLQEVSAQLEDLRIRSASESTAKDNDIKSLNQKLQEHAAKEEQLAASAEELT--------------VEVHSLQQQLAEEIQKRESTEDRVRLLEAELQAREELIASITRE-RSLEIEGVLDQRDQSIKELMEAQEQLASVVAARAELEQLLEGMQPEKIAREREIATLTAQKEALVLKLREFATEKDNAARQVTELSAG------------------LADAEAALDMLNEEKEMLQMAMAEADRVLR---AQLAEANERYNHLVSEGGDKAKELKKELDIVTEE----------------------------------------RDRRLAEVNELVAERDGLHDRIARIMEKFDGRTEEIERLHQERDAALQERERMAAECDELQAQIELVEVERKRVHTAMEEGVVKMTELEARVMTLEEEKGALREEVDIREEERAALIRELDSLKETVDTFRQRKDEEEKERSVWKQRIEEVNARVGEIS------EENERLLQSLNARETHIAYLQEELEREQLSVAEISQKLTDLETASNEQVAARQELETVSEIIRKEREDALEKVAALEQMIPELEAQMEQERG---VLSAAQKEAEQRHAVAQSSLEDVGAQVLRITQERETLASERDVLEARTQELQAQVTALEEERHSLMEERDRSTAIVQTLQGQLVEMEETISMLEEDYESR------------------------------------------------------------LDERGKVLIDLQTTKDEFAHRNTVLEAEIELLTKSTEQNGRLLEEERALVQELQKALEDLQTSFEEIRTRMGEQKNAHEEEKSAMIKSFAERDEEEKLKLQDIVNVMKGV--VGAVG--GGEQSPVDWESTPEGSLDELTASRTRLLVRINDLARKMRDMVEMQSLSLASAGEVEQRLHQIRAEHDAVSQAKTKLEQRVKKLEGVETELRGQLDEAIELSASREEESRQLRATVDALEREKEVMEKSHNALVQQSRDEAARLVTELENARQEVQR--------------VSKELYEATQAHSLDSSLPDALEKLREEQDRSSALAMELADVKLKLEGKERALEVLKRDLQRVSESQMSDTSATSPVSHDTLHSKDLSQPPVTAATVDQLRHRVTMLTEERDLMRSEVREKTRMID--------ELHVRTSTLEADVQSLERALGEADEVLRENEIELRRNNLVLEELDDK-RALVVKLEEEITALQSRVLQLEDAAIARDELKKEVDGLREMVEAKSAQIRHMAADFDARLAEEKALYLRSNRDGHTTDTYEADDEEDGLAARGGRVRRPAKRTPSGNVATSSIADMRSLVSHVVGVVGDYQSAVAALVARKDGFLDLLRSTTVG---------------GYERLPKRILDDLNILITKLHDVEDQHVILGKELSSVGALIDTTAKGGLGRPVS------DHEGNYHLTPGEYADLYEKAAAAEQYQRQLENSHALLKDHAMQTQVLKDALQSMTQESTSTQRQNGNEKVLQRQLDELR----------------------RVWTHELEANTALRELITRTQRDAMHAQDKARAAQARMREEFDELVDLLDAATKDAEMARSDVKLLEDKLNN-QFFEHQEAASAQEEMHTAERKALEGLVDALERERDRIVDDFRRVKERLEEKILQGETQIRHLEEVSLEKDDRIRRLDGRVQHLEDARRSAEIQVLRLEKERGRLVDRRHPSDAKTQSPSDAGDEGLRDEVGLLQKELKKIRQSSREMMVVLRETLKNTIGDVSGSGHDVMAVLGEND-----------------DKVAKVDMSRIKEQCRQLIS------------EVLYLRALVDR-----LSTWRADLKY-QKLYLSLTV-QSHR-------ATLAGVG--------LEPS-------GVGSDKPV---------------------------------------------------IKLKRCVDVVRSL--IRMRILARNWKQALSESQQHFFAELCGDTE---PILSRSAVGSWENLLDLEQDDPRMRELEEEVIRLKDAKLALENENERLAKAVASLHHLRVREGEEERGWEVAREPARDSGREMLRERRSLRDAYTREDDRTERDTYEDRQRVSSSGRYYGESTSTYSNESTAYRGSNRSGQLSPRWAPVSRGMNGRLQERRRGGG------------------------------------------------------------------------------------------
