# Supplementary material for: Coiled-Coil Proteins Facilitated the Functional Expansion of the Centrosome
Source: PLoS Comput Biol. 2014 Jun 5;10(6):e1003657. doi: 10.1371/journal.pcbi.1003657 (PMC4046923; doi:10.1371/journal.pcbi.1003657)
Supplement: Dataset S1 — Multiple-sequence alignments. This file contains alignments for the protein families spd-5, AKAP9/PCNT, PCM1, HAUS7 and HAUS8 in FASTA format and as HTML pages with highlighted coiled-coil domains. (ZIP) [file pcbi.1003657.s021.zip › alignments/HAUS7.html]

Multiple Alignment


1  
|

5  
|

10  
|

15  
|

20  
|

25  
|

30  
|

35  
|

40  
|

45  
|

50  
|

55  
|

60  
|

65  
|

70  
|

75  
|

80  
|

85  
|

90  
|

95  
|

100  
|

105  
|

110  
|

115  
|

120  
|

125  
|

130  
|

135  
|

140  
|

145  
|

150  
|

155  
|

160  
|

165  
|

170  
|

175  
|

180  
|

185  
|

190  
|

195  
|

200  
|

205  
|

210  
|

215  
|

220  
|

225  
|

230  
|

235  
|

240  
|

245  
|

250  
|

255  
|

260  
|

265  
|

270  
|

275  
|

280  
|

285  
|

290  
|

295  
|

300  
|

305  
|

310  
|

315  
|

320  
|

325  
|

330  
|

335  
|

340  
|

345  
|

350  
|

355  
|

360  
|

365  
|

370  
|

375  
|

380  
|

385  
|

390  
|

395  
|

400  
|

405  
|

410  
|

415  
|

420  
|

425  
|

430  
|

435  
|

440  
|

445  
|

450  
|

455  
|

460  
|

465  
|

470  
|

475  
|

480  
|

485  
|

490  
|

495  
|

500  
|

505  
|

510  
|

515  
|

520  
|

525  
|

530  
|

535  
|

540  
|

545  
|

550  
|

555  
|

560  
|

565  
|

570  
|

575  
|

580  
|

585  
|

590  
|

595  
|

600  
|

605  
|

610  
|

615  
|

620  
|

625  
|

630  
|

635  
|

640  
|

645  
|

650  
|

655  
|

660  
|

665  
|

670  
|

675  
|

680  
|

685  
|

690  
|

695  
|

700  
|

705  
|

710  
|

715  
|

720  
|

725  
|

730  
|

735  
|

740  
|

745  
|

750  
|

755  
|

760  
|

765  
|

770  
|

775  
|

780  
|

785  
|

790  
|

795  
|

800  
|

805  
|

810  
|

815  
|

820  
|

825  
|

830  
|

835  
|

840  
|

845  
|

850  
|

855  
|

860  
|

865  
|

870  
|

875  
|

880  
|

885  
|

890  
|

895  
|

900  
|

905  
|

910  
|

915  
|

920  
|

925  
|

930  
|

935  
|

940  
|

945  
|

950  
|

955  
|

960  
|

965  
|

970  
|

975  
|

980  
|

985  
|

990  
|

995  
|

1000  
|

1005  
|

1010  
|

1015  
|

1020  
|

1025  
|

1030  
|

1035  
|

1040  
|

1045  
|

1050  
|

9606.ENSP00000359239  
10116.ENSRNOP00000023305  
10090.ENSMUSP00000033737  
9615.ENSCAFP00000028296  
13616.ENSMODP00000009351  
13616.ENSMODP00000025890  
9258.ENSOANP00000001067  
28377.ENSACAP00000009587  
8364.ENSXETP00000039812  
7955.ENSDARP00000054478  
8090.ENSORLP00000002507  
99883.ENSTNIP00000005866  
51511.ENSCSAVP00000010979  
7719.ENSCINP00000014740  
7739.JGI131050  
10224.XP\_002734645  
7668.XP\_001191643  
7668.XP\_786419  
6412.190775  
283909.jgi|Capca1|224194|estExt\_fgenesh1\_pg.C\_390005  
225164.jgi|Lotgi1|234008|estExt\_fgenesh2\_pg.C\_sca\_460030  
45351.JGI206981  
10228.JGI56918  
5762.jgi|Naegr1|70112|fgeneshNG\_pg.scaffold\_38000089  
3218.JGI91882  
39947.LOC\_Os01g64920.1  
3702.AT5G17620.1-P

Homo sapiens  
Rattus norvegicus  
Mus musculus  
Canis lupus familiaris  
Monodelphis domestica  
Monodelphis domestica  
Ornithorhynchus anatinus  
Anolis carolinensis  
Xenopus (Silurana) tropicalis  
Danio rerio  
Oryzias latipes  
Tetraodon nigroviridis  
Ciona savignyi  
Ciona intestinalis  
Branchiostoma floridae  
Saccoglossus kowalevskii  
Strongylocentrotus purpuratus  
Strongylocentrotus purpuratus  
Helobdella robusta  
Capitella teleta  
Lottia gigantea  
Nematostella vectensis  
Trichoplax adhaerens  
Naegleria gruberi  
Physcomitrella patens  
Oryza sativa Japonica Group  
Arabidopsis thaliana

MGG------------------------------------------------------------------------------------------------------------------------------------------------------------------------------------------------------ARLGARNMA--GQDAGCGRGGDD-YSEDEGDSSVSRAAVEVFGKLKDLNCPFLEGLYITEPKTIQELLCSPSEYR----LEILEWMCTRVWPSLQDR--FSSLK----GV-PTEVKI---------QEMTKLGHE-LMLCAPDDQELLK-G-CACAQKQLHFMDQLLDTIRSLTIGCSS-----CS----SLMEHFEDTREKNEALLGELFSS---PHL-QMLLNPECDPWPLDMQP--LLNKQSDDWQW----------------ASASAKSEEEEKLAELARQLQESAAKLHALRTEYFAQHE--------QGAAAGAADISTLDQ--KLRLVTSDFHQLILAFLQVYDDELGECCQ----RPGPDLHPCGPIIQATHQNLTSYSQLLQVVM------------AVADTSAKAVETVKKQQG---EQICWGGSS---------------------------------------------------------------------------------------------------------SVMSLATK-MNELMEK---------------------------------------------------------------------------------------------------------------------------------------------------------------------------------------------------------------------------------------------------------------------------------------------------------  
----------------------------------------------------------------------------------------------------------------------------------------------------------------------------------------------------------------MAEKGAGGGAGGGSDDSYYEDVGDDCVVKAAVEVFEKLKGISCPFLDGLYITEPKTIMELLCRPSKYR----LDILEWMCIRVCPSLQDK--FSLLK----GN-AVDMKI---------QEMVKLGHD-LMLCAPDDQDLLM-G-RECPQKQLQFMDKLLDMMQSLATGCSS-----SS----SLKEHLEGATEKNDALLGELFSS---PNL-WAILKPESDPWPLDMQS--SLNKQCDDLPK----------------AGPSAQ-SEGEKVADLARQLQESATKLQTLRDQCFAQHK--------AG-----TDTSTVDQ--KLRLVISDFYQLILAFLQVYDDELGECCQ----RPVPSLHPSGPIIQAVYQTLASCGQLLKAVM------------EIADTSAEAMEAARKQEG---EPNCWSSNN---------------------------------------------------------------------------------------------------------SGISLAAR-IDEVTQKYKILTDRFHRGTR--------------------------------------------------------------------------------------------------------------------------------------------------------------------------------------------------------------------------------------------------------------------------------------------  
----------------------------------------------------------------------------------------------------------------------------------------------------------------------------------------------------------------MAEE----GAGAGSDDSYYKDVGDDCVVKAAVEVFEKLKGVNCPFLDGLYITEPKTIMELLCRPSKYR----LDILEWMCIRVCPSLQDK--FISLK----GN-ALDLKI---------QEMLKLGHE-LMFCAPDDQDLLM-G-RECPQKQLQFMDKILDMMQSLATGCSS-----SS----SLKEHLEDTTEKNEALLGELFSS---PNL-RAILKPESDPWPLDMQS--SLNKQSDDLPK----------------ADPSAQ-SEGEKVADLARQLQESATKLQTLRDQCFAQHK--------AG-----TDTSTIDQ--KLRLVISDFYQLILAFLQVYDDELRECCQ----RPVPSLHPSGPIIQAVYQTLASCGQLLRAVM------------EIADTSAEAMKAARKQEG---EPNCWSSNN---------------------------------------------------------------------------------------------------------SGISLAAR-IDEVTQKYKILTDRFHRGTR--------------------------------------------------------------------------------------------------------------------------------------------------------------------------------------------------------------------------------------------------------------------------------------------  
----------------------------------------------------------------------------------------------------------------------------------------------------------------------------------------------------------------MA--GLGGGGGHKAGD-Y---EG---VLKAAVEVFGKLKDLKCPVLEGLYITEPKTIQELLCTPSKYR----LEILEWMCVRVCPSWQEK--SSSLK----GA-PVEVKI---------QEMVKLGHE-LMLCGLADQELLK-G-RACAQKQLQLMDQLLDAVRSLNVGGAS-----CS----SAKEHLEDTREKNEALLGEVLSS---PCL-QTLLSPECDPWPVDVQP--LLEQQSDDWQR----------------ACPSVE-LEEEKVMELARQLQESVAKLQTLRVECFAQQK--------QGAAVSGADTSTLDQ--KLRLVISDFHQLVVAFLQVYDDELGECCQ----RPGPYLHPCGPIIQAVYQTLTSCSQLLKAVT------------EVTDTSANAVQMVEQQQG---EQMCWGSSN---------------------------------------------------------------------------------------------------------SIMSLASK-MEELTQKYKVFRDSLQKGVE--------------------------------------------------------------------------------------------------------------------------------------------------------------------------------------------------------------------------------------------------------------------------------------------  
--------------------------------------------------------------------------------------------------------------------------------------------------------------------------------------------------------------------Q----------------------------------DIDCPFLEGFYITGPKTIKEFLCTPSIYR----LDILEWLFIRLYPPFGDS--IATVP----DP-EAKEKI---------RELTKLGNE-LMLCGPNDQDLIK-G-CTEVKDQLCFFNKLIDLVTNLGPEYASN----FS----SVEENLQSLPKDYKMLLKKAFD----PSLRKEVLDPKLNPLPADTDA--SRRRTKEDLHR-------------------TAMESKKSKVEELSDKLTKLTEMLQTHKEESLVLAL----QSSPHHGKMSRQGNGPLG------LILSDFHQLVTAFIHVFENELQDHCH----RPAPHISPCGLLFQSVYETLTLFLQELKAVN------------EVTNTSEEVE--TKAEKPR-REKAYLGGDT---------------------------------------------------------------------------------------------------------CMVTLG-------MQPHLVPQ-------T--------------------------------------------------------------------------------------------------------------------------------------------------------------------------------------------------------------------------------------------------------------------------------------------  
--------------------------------------------------------------------------------------------------------------------------------------------------------------------------------------------------------------------MSGDRIGGNRIQAKYKDYLFNSIVEAVLDVFKKLVDIKCPFLEGLHITELKTKKQLLCSPSM-R----LTILEWLFVRLYPPSEEL--FATFK----DY-EAEEKI---------LELVRLGHE-LMLCGPDDQNLIK-G-YSNVKEQLCFFKQLLHLVSNLDAGHADF----FS----SA-ENFHNLVMNNEMLLHKLFS----SNV-QQILNPKLSAFPLDIEC--HFKIKKK-------------------------VKSSKNKVKELSQKLNEFTEMLEELKEYAV---L----QV-----KMPSKGSNNLSQ--AFRVTLSDLHQLIVAFFHVNEIEWREHSN----RPAPNINPCGPQFHSVCEILTLYNQELKAVS------------EVINTSKNVEKIVKGQKP---EKVYVAREN---------------------------------------------------------------------------------------------------------YMTTLASK-MEKLRQKHKLFQDPLQKSSE--------------------------------------------------------------------------------------------------------------------------------------------------------------------------------------------------------------------------------------------------------------------------------------------  
L--------------------------------------------------------------------------------------------------------------------------------------------------------------------------------------------------------------------------------------------------------------------------------------------------------------------------------------AEMVKLGHD-LMLCQPDDLDLIK-G-RE-EEKQLAFMDQCAGCNIILKKGPTSEADLGCA----YRVENFQDYAKKTEAFLTELFSS---PHL-QAALDPEINPWPLDIMP--LLADEKALHKR----------------TFVSAK-SSENVLEELSKMLKTTTADLEKLKEECCFLNR--------EAADPNNDSGNTVVQ--TLKVVVSDFHHLVTAFTQIYENELREHCN----RPAPEINQSGPLFQSVHQSLTLCHQELKAIA------------EVSDTSKKIMETVERQQH---ERRCWDSSH----------------------------------------------------------------------------------------------------------LVTLSTK-MEELRRKHELFHDTMQKLTA--------------------------------------------------------------------------------------------------------------------------------------------------------------------------------------------------------------------------------------------------------------------------------------------  
-------------------------------------------------------------------------------------------------------------------------------------------------------------------------------------------------------------------------------------------------------ELGCPALEGVFLSEAEDIQKLLCTPSSHR----LDILEWICTSVYPPLKEQ--FSSLK----ES-ESDLKI---------KGMAKLGYE-LTLCHANDLDLIQ-G-KASAQKQLRFLEQLVAVIPAVS-DSST-----CS----SPEESLLEMVCKNGEFMKQVFCS---PDL-QAVLNPQCHPWSSDIKH--LL-------------------------------------------------------------------------------------------------------------------------------------------------------------------------------------------------------------------------------------------------------------------------------------------------------------------------------------------------------------------------------------------------------------------------------------------------------------------------------------------------------------------------------------------------------------------------------------------------------------------------------------  
-----------------------------------------------------------------------------------------------------------------------------------------------------------------------------------------------------------------------------------------------------LQKLSCPCLEGVYLTDPQSIHELLCTPSSHR----LDVLQWLCSS------QP--MTKCK----KK-NHEIQKAVFTPNDSNTEIAKLCFD-LMLCHFDDLDLIK-G-HASPLKQISFIEQLLDVIQFPDIISNN-----VT------LESLSHSIRENEEFLKELFSS---PHF-YATLSPECNPWPADFKP--IL--------T----------------AEESLQKRFLLPLTDLSIRLC-NVTHCKNATISCSAQGNLQEQYSECVDLCSSVADGDKVIQ--TLRLALTDFHQLTVAFNQVYVNEFQEHCG----HPTPQMSPAGPFFQSVHQSLSTCCKELESIA------------QFTETSEKIVNVVNERYQ---SKEKWSGSS---------------------------------------------------------------------------------------------------------ISTLFKGE-RGLMTWWCQFVSQHPA------------------------------------------------------------------------------------------------------------------------------------------------------------------------------------------------------------------------------------------------------------------------------------------------  
-----------------------------------------------------------------------------------------------------------------------------------------------------------------------------------------------------------------------SVTDAQTHCRNNNMAGISKEHQLSLRVYNTLQSLGCPLVDGLYLREADSVQELLCTPSFHR----TDILKWICASICPSLKEK--FSSIK----ST-PNEESI---------EELTRFGYE-MMLCKANDKDLIK-G-LAPPLRQLVFLEQLLRVIQA---DSSL-----CS----GQNSSSGDEGVKSNDLLEELISQDHLPDL-YMLLDPACNPWPAHIRE--HLIRTHSAHNKINGNHSKFSDFVSRSDQDSRLVSHGEESLTEAMALLKSTQSTLDELHKECEFLQS--------------HSSGSAVLSPCALKLAISDMSQLMTAFGHIYNTDFKGYCQ----RSPPTLSSEMTVFQSVHQLLHTCNTELEAVK------------QLSETSTSLTHTLQQLQT---DRRYWSKGE----------------------------------------------------------------------------------------------------------KHTLPKQ-LEELKNRYMAFLSLHQS-----------------------------------------------------------------------------------------------------------------------------------------------------------------------------------------------------------------------------------------------------------------------------------------------  
--------------------------------------------------------------------------------------------------------------------------------------------------------------------------------------------------------------------------------RVWKMAGPSTH-LLARRVYDSLQAASCPLLPGLDLREADTMLQLLCSPSELR----SSILAWIFSSIVPSFVSK----AVS----VK-NPDVLT---------KEMAAVGEE-LMLCRKDDFDLIR-G-NTSPLRQLCFLEQLLTLISDTAKPCGS-----------------------GEALLSELFADENLPHL-RQMLEPALNPWPVNIRSVRVPSVQHSSSSRLQGFRSADRCLSMALHKSSRSPYKPRKEAGDVAALLQKTQSELEQLQSKCDFLHS---------------QQKSPSFCASSLRLAAGDLQQLMTTFCPVYESNLKAHCS----REPPSFSTGTDIFQRVLQLLQACNTELEMLS------------QVPEASACLEEEVKQLQT---QPRYWSHGE----------------------------------------------------------------------------------------------------------KLTLADQ-LKEFNRRIGVLLYQISPEQAPPSPDKTD---------KQV------------------------------------------------------------------------------------------------------------------------------------------------------------------------------------------------------------------------------------------------------------------------  
------------------------------------------------------------------------------------------------------------------------------------------------------------------------------------------------------------------------------------MARALTEDQLARDVFTSLQAVSCPLVEGVKLQDSESILQLLCAPSQHR----TDILTWICCRIKPNFCSSNTKACLR----TK-EPETLW---------KEMALLGQE-LMLCRAADQDLIREA-STSVQWQLSLLQQLLTLVPGSKESSGS--------------------RTDTEALLNELCASENVSQL-SHMLTPTLDPWPSHIKT----------------FQTGNRASCGA----------SREEAADVSGLL------------------------------------PSPPVCCKLLQVTSGS------------------------------------------------------------------------------------------------------------------------------------------------------------------------------------------------------------------------------------------------------------------------------------------------------------------------------------------------------------------------------------------------------------------------------------------------------------------------------------------------------------------------------------  
MDV--------------------------------------------------------------------------------------------------------------------------------------------------------------------------------------------------------------------------------------NMD-FATSLLEMFEVLDCPFTENI---DASYLIQFLLNPSPQR----MQLLAWVISCIDNELSETIHNYESKRLNASKLEIDGRV---------QLLTEISSQHLCICGPKDFDLIR-G-KAPEKIHLDFWKNILSIATAVRVGGAD---------------SINEEFHREESDQENMLRA---LNA--DCFSETLNRLPITIERDLQISKKSSKTRE---------------AKPNLSLENLSQKIEDLETQLDKEKRRLECILNEYPEHKN----------VVRNDVLVNQLTC--SMHLLLGDLRQLQTTYNQTHDNNLQAYCK----RDKVEYGKLGLTIHHLQRKIEAFLKVMDELQ------------GMRTSHESVAVSSTNFAP---------------------------------------------------------------------------------------------------------------------------NQLLKSLNEVNQSISC---------------------------------------------------------------------------------------------------------------------------------------------------------------------------------------------------------------------------------------------------------------------------------------------------  
MGF--------------------------------------------------------------------------------------------------------------------------------------------------------------------------------------------------------------------------------------NKETFMLDIKRTFEILECPFTINM---ELSYMEELLLHQSPQR----MRVLAWVLSLIDEEITEKIENYESNRVNPSKLNIDGRV---------QLLTEISSDILGLCHEKDYDLIR-G-KASDETQLEFWKNLLGIVLTVNEEKTD---------------PNTAKFEEGNIILEELLMN---CNL--NEF-EYLTNLPLSV--DCKDSKLNGKT---------------------TSTEEIEKTVVTLETQLEEHKALLNKYIAENPNHLE----------NNQCGSLMKKLTC--SVQVLLGDLAQLQTTYQQTHDINYETYCR----KSTLKFTRLGDTMHHLQRKVETFLKMMSEMK------------EMQRKHENVT-----------------------------------------------------------------------------------------------------------------------------------TELLPATN------VT---------------------------------------------------------------------------------------------------------------------------------------------------------------------------------------------------------------------------------------------------------------------------------------------------  
MAA---------------------------------------------------------------------------------------------------------------------------------------------------------------------------ISRK---KSEL-----------------------------------------------------ATSFKSRLESLDCPYIEGV---EESWITQLIFTPGEHR----LRLLQWLFSRIDPKLCDIVEGYHST----QE-----RE---------QRLLFSASI-LGLCKREDLDVIR-G-TCSFPGQASFMDQLIDMVCIINSSQDATQRAMSSPGHISESRPLVEQAEADSHLMDRLSRQ---YTF-QSMFSHKLALLPPDIKH--SVEKGWMDAGN--------QKGSGPDPP---DVGTLLEAAAGLSRQLEISTAQLHHLQVKHTYGEP-------------DHLSVSRVSQ--TLGLVLSELEQLLTGFSYCYEQELRSWCN----RTPPTLSELGPAFKRVHSLLTNFTQLLTSLD------------TLKTSYSSICQGTNPQQDQ-VSDRHNGVFN---------------------------------------------------------------------------------------------------------SLALLGQSAALKMQDCISVLDQSVHRTSALQDSTLLASTAL-----S--------------------------------------------------------------------------------------------------------------------------------------------------------------------------------------------------------------------------------------------------------------------------  
MAA---------------------------------------------------------------------------------------------------------------------------------------------------------------------------ISRR-EKFVAI-----------------------------------------------------SQSFRSRLEEVNCPFVEDV---DDSWIMEHIFTPGEQR----IRLLQWLFSRFDPHLTELFDSQCIP----SDTKIDSRL---------QRLLYVSSI-LGLCNPNDVELVR-G-NTSKSKQIDFIDQLIDMVYISETSEDVSKRAMSSPGLIDESVSLPEQVNHDCNLLETIVRQ---ENM-NALFSVKTSLLPPDLTK--NMRTSMKELGY--------GPDQRYELPQTYKLECLSEE---LAKQLIRSTAHLKELHACPEYDE----------------SVVKKVIK--TTELVSSELAQLVTGFTFTFENEMRPWCG----RSPPILSDIGVSFKRVYTLLQNFMQTLQSFS------------MIKTSVNSI-----LKSQK-DVD-----------------------------------------------------------------------------------------------------------------IPQEAEM-IDRFKECVQILND------------LSSSQQE-----KRY------------------------------------------------------------------------------------------------------------------------------------------------------------------------------------------------------------------------------------------------------------------------  
-------------------------------------------------------------------------------------------------------------------------------------------------------------------------------------------------------------------------------------------------------ALDCPYVDGV---DETWISELLYQPGEAR----IRLLQWLLSRYDVKFAEILDSQYWT----SEAKMDTRI---------QAMTKLCSC-LGLCRPGDVDLIR-GVSTSHSKQASFWDKLLDIVTISDASEETHRDVTSSPGIVSESLPLWDQFCHDCQFVSSLAHG---RDL-QEALSPKVNLFPPDITR--ILAKRAADE-E--------QRAA---PP---SIDALLEMSTHLSMELQQANQHLKDLQKAYPYPTP-------------DPKALNKVCQ--TMKLVLSELVQLVTSFTFMFESEMRQFCN----KAPPQLTQLGPAIKRVHTLLQQFSGNEIKLEVTLSVIVDKNENELEVILSVIVDKNEIKLEV--------------------------------------------------------------------------------------------------------------------TLSVIVDKNENELEVILSVIVDKNEIKLEVTLSVIVDKNEN-----E--------------------------------------------------------------------------------------------------------------------------------------------------------------------------------------------------------------------------------------------------------------------------  
-------------------------------------------------------------------------------------------------------------------------------------------------------------------------------------------------------------------------------------------------------ALDCPYVDGV---DETWISELLYQPGEAR----IRLLQWLLSRYDVKFAEILDSQYWT----SEAKMDTRI---------QAMTKLCSC-LGLCRPGDVDLIR-GVSTSHSKQASFWDKLLDIVTISDASEETHRDVTSSPGIVSESLPLWDQFCHDCQFVSSLAHG---RDL-QEALSPKVNLFPPDITR--ILAKRAADE-E--------QRAA---PP---SIDALLEMSTHLSMELQQANQHLKDLQKAYPYPTP-------------DPKALNKVCQ--TMKLVLSELVQLVTSFTFMFESEMRQFCN----KAPPQLTQLGPAIKRVHTLLQQFSGLLTSLQ------------SMHTSYYSIVNTKGIQSE------------------------------------------------------------------------------------------------------------------------------ESRPADLM--------------------------------------------------------------------------------------------------------------------------------------------------------------------------------------------------------------------------------------------------------------------------------------------------------  
MEG-------------------------------------------------------------------------------------------------------------------------------------------------------------------------------------------------------------------------------------ANVTELIEDIKLKLVKLSCPYVENV---ADSWLQNLIFTPGTSR----INLLKWLLSKCDIGLSEILHSYNAT----DLNHFESDA---------QNILSMVSS-LGLCFPGDIALVK-G-TTTGQSQMIFTKTLVDLASELNNPDEFDSCT----------------YTKGNSLSDKIANR---QPI-TLSYCNKSNFLPADLQH--ILDIRMADRSA--------KNSV---IP---DVNELQSNLKQLDSRIEALTSKMK-LNSNQPEQEA-------------IKANSDK--N--EMKLVLSDLNMMMASFAHSYKDVMH-LCQADNTKEYHQLND---------GLLQDFSEKIEKLN------------NVLSSYGPIKCDIESTFNQ-LTKSTTDDKD---------------------------------------------------------------------------------------------------------DL----KKALESLTEYNKTLERSLLYSKYT----------------HND------------------------------------------------------------------------------------------------------------------------------------------------------------------------------------------------------------------------------------------------------------------------  
MASRRTLSLFGSEPPAIRYSSAENSDSPTYVLQEPIAAIPDLPMPLGSCGDREKLKSLEPKAQKWDLKRVLSLNNRGLLEHIETEVETRELNNRLREDVFGMPRGNCFRPLGTRNNNVGHNLPPKTQNPFLRRDSGEFVATGTYWSPIGAPSGRISVNSRPTSRTSQISSTQLNVQKKLTTKSHVNSIPRHRGQKEEEPKYADPLINAPPSLNQRVSEMASLEADSLRWEKARKFKRKSKSYAMMLKLLQCPYTEAV---DDAWIAELLFTAHEPR----MRLLQWLFSRFHARLNEVVDPSFYS----SESRMDSVL---------HRLLFTAST-LGLCGPSEVEVIK-G-SAPVQRQAAFWDQLLDLVCVIDASHDPQKNIMQSPGFIGEGTGLHQQFLADCQLVDDLVDQ---ENV-DQVFQIEAKVLPPDISR--MIEQKTKPEER---------------IP---DSAQLTEMLQELKEDLRRQEDMLHELKQDHPSST---------------SSSDDKLQR--TLQLVFSELSQLISGFSCSYDNDIQQWCN----KTPPQLSQLGQSFKKVHALSQLFSTMLNQLK------------KIRGNYSQISSDMKSSIHR-LRDQKLSLGS---------------------------------------------------------------------------------------------------------DL----HSALANFQISAEILEDSICRQGEEVTSRNTTLESLKTPVVKFN------------------------------------------------------------------------------------------------------------------------------------------------------------------------------------------------------------------------------------------------------------------------  
MAD---------------------------------------------------------------------------------------------------------------------------------------------------------------------------ISKQLTKTSKF-----------------------------------------------------SHSLKERLDTLGCPYTEGV---DESWVLELISKPGEPR----IRLLQWLFSKLDSKLNELLDPHNAP----IESKMDSRI---------QRLLYVSST-LGLCKYNDVDLIK-G-ITTGSKQCSFVDQLLDLVCIVDVSEDPRAKIFQSPGVVGESSGLSEQYIADCNYVDQLVID---ENM-STLFNSKVNLLRPDIYK--NIEMKWVEKGF--------SKG----IPPSVDINQLKEKAYTVANNLDRQTVLLQDLTRSCTYSEN-------------GSAIVSTTTR--TLQVVLSELSQLVVSFTYCYENEMRLWCN----KTPPVLTDLGLAFKRVYNVLEQFVEFLKHLD------------SIHKSYSELGQDVKIKVTS-KTN-----------------------------------------------------------------------------------------------------------------LVLTSQMALENFQECLTILDESIQRTTGPEASCISTITSE-----QFC------------------------------------------------------------------------------------------------------------------------------------------------------------------------------------------------------------------------------------------------------------------------  
MAA-------------------------------------------------------------------------------------------------------------------------------------------------------------------------------------------------------------------------------------GKHRISISLFRRKLEELDCPYIEGV---DDSWMEELLYKPGEPR----MRLMQWLLGKFDPTINEMLEGQH-K----MRSRNDSRL---------QRLLFAAHL-IGLCHCDDIALIK-G-ETSHSKQAVFFENLIDMVSHVDAVESKARQQRIPPAI-----SLAEQFESDCRLLDTLCRQ---EDL-SEVFKMRIQLSPPDLVK--ANKINDNKV-----------P----------DSKHLAGVADNLSEELQTQTQHLEELQQKWPFRQS-------------DHQEVDKVSK--TLELTLSTLSQQVAGFVHCYESEMRPWCS----RSQPVLSELGPAFKRVYTLLQKLVHLLGSLE------------LVKKSHESICGETKSRLEE-VKA------S---------------------------------------------------------------------------------------------------------GLSSSTKDFLENLPDCVAILEGALERYQQTW---------------HKG------------------------------------------------------------------------------------------------------------------------------------------------------------------------------------------------------------------------------------------------------------------------  
MAG--------------------------------------------------------------------------------------------------------------------------------------------------------------------------------------------------------------------------------------------STWVQRLESLDCPYVEDV---DESWVTELIFKPGEAR----IRLLQWLFARLEPSIEEILDNQTIQ----ASGRGDSRL---------QTLLFIASS-LGLCATHDIELIK-G-TCPASKQKIFMNTLIDVVCAID------QSQKMGSG------HLEKKFTSNCLLMDKICHQ---ENL-QQVFLTNINLFPYDLTN--ITRGSESASVT--------EK----------DYKDLLDSASSIADQLVERQNQLADLKSSYSPCEY-------------DMNKIRPILR--RLKLTLSTLAQIMAGFSHCYETEIKTWCQ----KPSPRLSALGLVVKRVDSLLAHFSQTLQGIA------------TIKGSYEKLGNDFMTQTDE-ALS------E---------------------------------------------------------------------------------------------------------EQIATVKT-LDGVRRSVDILEKSVSRLLSN-------------------------------------------------------------------------------------------------------------------------------------------------------------------------------------------------------------------------------------------------------------------------------------------  
MYM-----------------------------------------------------------------------------------------------------------------------------------------------------------------------------------------------------------------------------------------------------LNKYFVPSYFPDEVNNALRLPLDSNDHKEQSILRAFSW---------------------------------------------------LGIIEFCDMEIVR-G-ECTLERNIKFIMDILDLIYSVKGFNNT--------------EELKQQFTKDHNFINHICHNK------DSVLANDMKLFSNEMSS--KFKRKKLPDAA----------------SFKTDVEKANLHIQRYQDIIEELQTNIDEQHRGQSVVLNEQTLHTGGNYYIMSNLSVEHNEKIQLLGEAIGRFSTLVDQFMHVYHEEFTQWINPKPVEEKSQLEELSVSIPEVLTTLETEKLLLMNIK----VIRYELHHQTDDSKPFVLYTIQIENRRKRKQLGVSSSHLENAELSGDVVPLNYSSPFDREYKYSYVEEFDDDGISTSSHSETTSNGGGGGASPNQHHHHHHHHNHNNQHHHESPRETCGFMNHSDWTVTKRYSELYDYHQRFIEITTTTTTSLQNSKDMKSLFPKKLIIGNLKKENIEKRVEQFNEYFKKMVDIILKEDNSELQKLVTHFFSAKPPLVLSESNPSKHPNKNIDWMIHKPNSRNNWFFSAPLGFEKFSTSFDRRAEYISPIFQNISIETLINLWEKFISKLEKVELVDSNHDICLYTYMQKASFINDFITVQFVQEIPPKSPETNTETTAEVTSPNKNEELDDTLSNMSPPNFESNVGDILKRFSIRRTTDFDIRDLLSDTSNNLYDDTQSTEEVRKPKFSIVIYSRSKFDLKDTNEKRVKSWLNDFKKFVLTQAPTVQLCNSFQWE  
----------------------------------------------------------------------------------------------------------------------------------------------------------------------------------------------------------------------------------------------MDDVHHKLSLLNYP--------RSSAPSQSLLYAGLER----YALLDWLFFRLLGD-KSPFTQQDLLGEGADRDEEAARI---------QYLAEIAKY-LGLTPTVDPDAIQ-G-KGSYESRAEMLHLIVELVEASSVTDNP-------------EWSVDDQVAKDILLVDAIAEKQ------TQVFSDECKLFPADVQI--------------------------MSNIAMHDITDLERQLSEQVKSVERLQSSVAELSAKQSYNPN-----------------EDYVDAEIELRARLATFLDSAKSFNIIYAKEIRPWTH---MMDVPQLHGLGPAANRLLDSYSQLLKLLRNLQ------------KMRDSYSAVAAGSDVANDAISASTEESSLV---------------------------------------------------------------------------------------------------------KLVAECEEALLLLNNGLSILSLSLERDYIGGIHTDKPASPL--------------------------------------------------------------------------------------------------------------------------------------------------------------------------------------------------------------------------------------------------------------------------------  
MAS-----------------------------------------------------------------------------------------------------------------------------------------------------------------------------------------------------------------------------------------KQMEEIQRKLAVLAYP--------RANAPAQSLLFAGVER----YRLLEWLFFRLLGD-RSPFTQQNWQGDSLDRDEENSRI---------QHLAEIANF-LGITPSVDTEAIQ-G-RGSYDERVELLCLIVDLVEASCYADNP-------------EWSVDEQLAKDVLLVDSIAEKQ------AQIFSEECKLFPADVQI--------------------------QSIYPLPDITELELKLSEYTKKMSNLQLMVQELASKYDYNPN-----------------EDYAETELKLREHLQSFLETVKSFNMIYTKEIHPWTH---MMEVPQLHGFGPAANRLLEAYNTLLKFLSNLR------------SLRDSYAAMAAGS-----L-SASNEPSSVT---------------------------------------------------------------------------------------------------------KIISDCESALTFLNNSLSILSTSVARE---------QGETLNSQ-----------------------------------------------------------------------------------------------------------------------------------------------------------------------------------------------------------------------------------------------------------------------------  
MAA-----------------------------------------------------------------------------------------------------------------------------------------------------------------------------------------------------------------------------------------KQMEEIQKKLRLLSYP--------RANAPAQSLLFAGMER----YALLEWLFFKLLGD-KSPFSQQNLQGDAGVRDEETVRI---------QYLAEIAKF-LGITPTVDIEAIQ-G-HGTYEDRMEMLRNIVDLVEASLFSDNQ-------------EWSIDEQVAKDIQLIDAIAERQ------SLIFSEECKLFPADVQI--------------------------QSIYPLPDVSELETKLSEQAKILSNLQQKVDDLAAKHAYNPD-----------------EEYTEVESQLRARLESFLETARAFNTIYTKEIRPWTH---MMEVPQLHGFGPAANRLLEAYNMLLKFLGNLK------------NLRDSHAALSIGS-----SGTVAGEPSSVT---------------------------------------------------------------------------------------------------------RIVSDCEAALTVLNRDLGILSASIARE---------QGERL--------------------------------------------------------------------------------------------------------------------------------------------------------------------------------------------------------------------------------------------------------------------------------
