# Supplementary material for: Coiled-Coil Proteins Facilitated the Functional Expansion of the Centrosome
Source: PLoS Comput Biol. 2014 Jun 5;10(6):e1003657. doi: 10.1371/journal.pcbi.1003657 (PMC4046923; doi:10.1371/journal.pcbi.1003657)
Supplement: Dataset S1 — Multiple-sequence alignments. This file contains alignments for the protein families spd-5, AKAP9/PCNT, PCM1, HAUS7 and HAUS8 in FASTA format and as HTML pages with highlighted coiled-coil domains. (ZIP) [file pcbi.1003657.s021.zip › alignments/HAUS8.html]

Multiple Alignment


1  
|

5  
|

10  
|

15  
|

20  
|

25  
|

30  
|

35  
|

40  
|

45  
|

50  
|

55  
|

60  
|

65  
|

70  
|

75  
|

80  
|

85  
|

90  
|

95  
|

100  
|

105  
|

110  
|

115  
|

120  
|

125  
|

130  
|

135  
|

140  
|

145  
|

150  
|

155  
|

160  
|

165  
|

170  
|

175  
|

180  
|

185  
|

190  
|

195  
|

200  
|

205  
|

210  
|

215  
|

220  
|

225  
|

230  
|

235  
|

240  
|

245  
|

250  
|

255  
|

260  
|

265  
|

270  
|

275  
|

280  
|

285  
|

290  
|

295  
|

300  
|

305  
|

310  
|

315  
|

320  
|

325  
|

330  
|

335  
|

340  
|

345  
|

350  
|

355  
|

360  
|

365  
|

370  
|

375  
|

380  
|

385  
|

390  
|

395  
|

400  
|

405  
|

410  
|

415  
|

420  
|

425  
|

430  
|

435  
|

440  
|

445  
|

450  
|

455  
|

460  
|

465  
|

470  
|

475  
|

480  
|

485  
|

490  
|

495  
|

500  
|

505  
|

510  
|

515  
|

520  
|

525  
|

530  
|

535  
|

540  
|

545  
|

550  
|

555  
|

560  
|

565  
|

570  
|

575  
|

580  
|

585  
|

590  
|

595  
|

600  
|

605  
|

610  
|

615  
|

620  
|

625  
|

630  
|

635  
|

640  
|

645  
|

650  
|

655  
|

660  
|

665  
|

670  
|

675  
|

680  
|

685  
|

690  
|

695  
|

700  
|

705  
|

710  
|

715  
|

720  
|

725  
|

730  
|

735  
|

740  
|

745  
|

750  
|

755  
|

760  
|

765  
|

770  
|

775  
|

780  
|

785  
|

790  
|

795  
|

800  
|

805  
|

810  
|

815  
|

820  
|

825  
|

830  
|

835  
|

840  
|

845  
|

850  
|

855  
|

860  
|

865  
|

870  
|

875  
|

880  
|

885  
|

890  
|

895  
|

900  
|

905  
|

910  
|

915  
|

920  
|

925  
|

930  
|

935  
|

940  
|

945  
|

950  
|

955  
|

960  
|

965  
|

970  
|

975  
|

980  
|

985  
|

990  
|

995  
|

1000  
|

1005  
|

1010  
|

1015  
|

1020  
|

1025  
|

1030  
|

1035  
|

1040  
|

1045  
|

1050  
|

1055  
|

1060  
|

1065  
|

1070  
|

1075  
|

1080  
|

1085  
|

1090  
|

1095  
|

1100  
|

1105  
|

1110  
|

1115  
|

1120  
|

1125  
|

1130  
|

1135  
|

1140  
|

1145  
|

1150  
|

1155  
|

1160  
|

1165  
|

1170  
|

1175  
|

1180  
|

1185  
|

1190  
|

1195  
|

1200  
|

1205  
|

1210  
|

1215  
|

1220  
|

1225  
|

1230  
|

1235  
|

1240  
|

1245  
|

1250  
|

1255  
|

1260  
|

1265  
|

1270  
|

1275  
|

1280  
|

1285  
|

1290  
|

1295  
|

1300  
|

1305  
|

1310  
|

1315  
|

1320  
|

1325  
|

1330  
|

1335  
|

1340  
|

1345  
|

1350  
|

1355  
|

1360  
|

1365  
|

1370  
|

1375  
|

1380  
|

1385  
|

1390  
|

1395  
|

1400  
|

1405  
|

1410  
|

1415  
|

1420  
|

1425  
|

1430  
|

1435  
|

1440  
|

1445  
|

1450  
|

1455  
|

1460  
|

1465  
|

1470  
|

1475  
|

1480  
|

1485  
|

1490  
|

1495  
|

1500  
|

1505  
|

1510  
|

1515  
|

1520  
|

1525  
|

1530  
|

1535  
|

1540  
|

1545  
|

1550  
|

1555  
|

1560  
|

1565  
|

1570  
|

1575  
|

1580  
|

1585  
|

1590  
|

1595  
|

1600  
|

1605  
|

1610  
|

1615  
|

1620  
|

1625  
|

1630  
|

9606.ENSP00000253669  
10090.ENSMUSP00000040802  
10116.ENSRNOP00000043408  
10116.ENSRNOP00000038415  
9615.ENSCAFP00000022866  
13616.ENSMODP00000018784  
9258.ENSOANP00000025246  
59729.ENSTGUP00000000475  
9031.ENSGALP00000005905  
69293.ENSGACP00000015326  
7739.JGI69118  
7668.XP\_001186511  
283909.jgi|Capca1|224384|estExt\_fgenesh1\_pg.C\_2150004  
225164.jgi|Lotgi1|231693|estExt\_fgenesh2\_pg.C\_sca\_210089  
45351.JGI242019  
400682.Aqu1.224157  
10228.JGI60793  
595528.CAOG\_05450T0  
645134.SPPG\_04048T0  
3702.AT4G25190.2-P  
3702.AT2G20815.1-P  
3702.AT2G44190.1-P  
3702.AT3G60000.2-P  
3702.AT2G24070.1-P  
3702.AT4G30710.2-P  
3702.AT5G43160.1-P  
3702.AT1G49890.1-P  
3702.AT3G19570.2-P  
39947.LOC\_Os03g38480.1  
39947.LOC\_Os09g11440.2  
39947.LOC\_Os10g40620.1  
39947.LOC\_Os07g02470.1  
39947.LOC\_Os03g55470.1  
39947.LOC\_Os09g25500.1  
39947.LOC\_Os08g34320.1  
39947.LOC\_Os02g55110.1  
39947.LOC\_Os03g10820.1  
39947.LOC\_Os10g20510.1  
3218.JGI172159  
3218.JGI172191  
3218.JGI100775  
3218.JGI82152

Homo sapiens  
Mus musculus  
Rattus norvegicus  
Rattus norvegicus  
Canis lupus familiaris  
Monodelphis domestica  
Ornithorhynchus anatinus  
Taeniopygia guttata  
Gallus gallus  
Gasterosteus aculeatus  
Branchiostoma floridae  
Strongylocentrotus purpuratus  
Capitella teleta  
Lottia gigantea  
Nematostella vectensis  
Amphimedon queenslandica  
Trichoplax adhaerens  
Capsaspora owczarzaki ATCC 30864  
Spizellomyces punctatus DAOM BR117  
Arabidopsis thaliana  
Arabidopsis thaliana  
Arabidopsis thaliana  
Arabidopsis thaliana  
Arabidopsis thaliana  
Arabidopsis thaliana  
Arabidopsis thaliana  
Arabidopsis thaliana  
Arabidopsis thaliana  
Oryza sativa Japonica Group  
Oryza sativa Japonica Group  
Oryza sativa Japonica Group  
Oryza sativa Japonica Group  
Oryza sativa Japonica Group  
Oryza sativa Japonica Group  
Oryza sativa Japonica Group  
Oryza sativa Japonica Group  
Oryza sativa Japonica Group  
Oryza sativa Japonica Group  
Physcomitrella patens  
Physcomitrella patens  
Physcomitrella patens  
Physcomitrella patens

-------------------------------------------------------------------------------------------------------------------------------------------------------------------------------MADSSGRGAGKPATGPTNSSSAKKKDKRVQGGRVIE----SRYLQ----------------------------------------------------------------------------------------------------------------------------------------------------------------------------------------------------------------------------------------------------------------------------------------------------------------------------------------------------------------------------------------------------------------------------------------------------------------------------------------------------------------------------------------------------------YEKKTTQKAPAGDGSQTRGKMSEGGRKSSLLQKSKADSS-----------------------------------------------------------------------------------------------GVGKGDLQ----------------------------------------------------------------------------------------------------------------------------------------------------------------------------------------------------------------------------------------------------------------------------------------------------------------------------------------------ST--------LLEGHGTAPPDL-----------DLSAINDKSIVKKTPQLA--------------KTISKKPESTSF--SAPRKKSPD-----------LSEA-----------MEMMESQTLLLTLLSVKMENNLAEFERRAEKNLLIMCKEKEKLQKKAHELKRRLLLSQRKRELADVLDAQIEMLSPFEAVATRFKEQYRTFATALDTTRHELPVRSIHLEGDGQQLLD--ALQHELVTTQRLLGELDVGDSEENVQVLD--------------LLSELKDVTAKKDLELRRSFAQVLELSAEAS----KEAALANQEVWEETQGMAPPSRWYFNQDSACRESGGAPKNTPLSEDDNPGASSAPAQATFISPSEDFSSSSQAEVPPSLSRSGRDLS  
--------------------------------------------------------------------------------------------------------------------------------------------------------------------------------MADSSERDAGKSAAAGACAVPKTKGRRVQGRRVVE------------------------------------------------------------------------------------------------------------------------------------------------------------------------------------------------------------------------------------------------------------------------------------------------------------------------------------------------------------------------------------------------------------------------------------------------------------------------------------------------------------------------------------------------------------------SRYLQYDKKAKKVSGAAKEEKPPEGRKASTVPRSREESQ------------------------------------------------------------------------------------------------VMGTGNLQ----------------------------------------------------------------------------------------------------------------------------------------------------------------------------------------------------------------------------------------------------------------------------------------------------------------------------------------------ST--------MLEGHGMNPPDL-----------DLSAIDDKILSRKASWPD-----------REMTDKAKSTSFISC--DKKRILRKK--------RRDLQET-----------MDMMESQTLLMTLLSVKMENNLALLEERAEKDLAAMCHEKERLQRQALELRRQLLLRQKHQELAATLDAQIEVLSPLQPVLERFKEEYMTLGRALDTTRHELPMQAVHMEGSGQELLD--DLEPALRITLQLLGDLSICSPYATAQVQGASAQEPGASTQLSCLLKELKGLVTEKDLELRRLVSQVVELSSQAS----KEAALMNQEVWEEAEGALTSSQWYFSPDACRDDSPS---------------------------------------------------  
--------------------------------------------------------------------------------------------------------------------------------------------------------------------------------TVHFSSLSCRKPAASGAGVVPKTKGSKVKRGRVVE------------------------------------------------------------------------------------------------------------------------------------------------------------------------------------------------------------------------------------------------------------------------------------------------------------------------------------------------------------------------------------------------------------------------------------------------------------------------------------------------------------------------------------------------------------------SQYLQYDKKTKAVVSVPVKGEKPPEGRKSSTVPRSAEESK-----------------------------------------------------------------------------------------------VMGTGNLQ----------------------------------------------------------------------------------------------------------------------------------------------------------------------------------------------------------------------------------------------------------------------------------------------------------------------------------------------SI--------MLEGHDLNPPDL-----------GLSDIDEESMSRKAPQLE-----------RKVAGNDKPTSLLRP--DKQDILKEE-------MGQDLQET-----------LDMMESQMLLMTLLSVKVENNLALLEEKAEKDLAAMCHEKERLQRQALELRCQLLLRQKHQELAAALDAQIEVLSPLQPVLLPRGIQYSGQSPGHHPT-------------------------------------------------------------------------------------------------------------------------------------------------------------------------------------------  
--------------------------------------------------------------------------------------------------------------------------------------------------------------------------------MAGASERDAGKPAASGAGAVPKTKGRKVQGGRVVE------------------------------------------------------------------------------------------------------------------------------------------------------------------------------------------------------------------------------------------------------------------------------------------------------------------------------------------------------------------------------------------------------------------------------------------------------------------------------------------------------------------------------------------------------------------SRYLQYDKKTKKVSVAAKGEKPPTEGRKASTVPRSREESK-----------------------------------------------------------------------------------------------VMGTSNLQ----------------------------------------------------------------------------------------------------------------------------------------------------------------------------------------------------------------------------------------------------------------------------------------------------------------------------------------------ST--------MLEGHGLNPPDL-----------DLSAIDDKSMSRKAPQLE-----------RSVAGTDKSTSLLRP--DQKRTLRKK--------RRDLQET-----------MDMMESQTLLMTLLSVKVENNLALLEEKAEKDLAAMCHEKERLQRQALELRRQLLLRQKHQELAAALDAQTEVLSPLPPVLERFKEEYKTLGRALDTTRHELSMQAVHMEGSGQELLD--DLEPALRTTLQLLGDLSICSPEDSAQVQGASTQQPGASAQLNCLLKELKGLVAEKDLELCRLVSQVVELSSQAS----KEAALTNQEVWEEAQGTLTSSQGYFSPDVRKDHSPTQDRTNSSSLDP----------------------------------------  
-----------------------------------------------------------------------------------------------------------------------------------------------------------------------------MADSSGRGAGKPSSGGPSTPGGGAKVKGRRPQGGRVVE----SRYLQ----------------------------------------------------------------------------------------------------------------------------------------------------------------------------------------------------------------------------------------------------------------------------------------------------------------------------------------------------------------------------------------------------------------------------------------------------------------------------------------------------------------------------------------------------------YEKKTTKKASAADTLKTIGKMPEGGRKVHLLQRSRGPTD-SS--------------------------------------------------------------------------------------------GIGKGDLQ----------------------------------------------------------------------------------------------------------------------------------------------------------------------------------------------------------------------------------------------------------------------------------------------------------------------------------------------ST--------LLEGHGTAPPDL-----------DLSAINDKSMLRKTPQLE--------------KTMSKKTESMSF--SASQRKSPD-----------LSEA-----------MEMMESQTLLLTLLTVKMENGLAQFEEKAERNLLIMCKETEKLQKKAHELKRKLLLCQRKRELADVLDAQIEMLSPYEPVAERFREQYKTFATALDTTRHELPVKSVHLDGNGQQFLD--DLQRELTTTCHLLGELGISSLEENVKALD--------------LLSEIREMTQKKDLELRRSFAQVLELSAEAS----KEAALVNQEVWEDAQGLEASSQWYFNQEGACGEASGEVRTPLLLGTGEPHAV-----------------------------------  
-------------------------------------------------------------------------------------------------------------------------------------------------------------------------------------------------SSSERKDKQIESGRIIES-----RYLQ-----------------------------------------------------------------------------------------------------------------------------------------------------------------------------------------------------------------------------------------------------------------------------------------------------------------------------------------------------------------------------------------------------------------------------------------------------------------------------------------------------------------------------------------------------------CEKKKVVKGKAGSANRKGKQNDGGVKSSLQVKLERDNS-----------------------------------------------------------------------------------------------QVEKDDLQ----------------------------------------------------------------------------------------------------------------------------------------------------------------------------------------------------------------------------------------------------------------------------------------------------------------------------------------------ST--------LLDGHRTAPPDL-----------DLSAINENSILKNSCSLG-------------------PSSFVPL--AITIWHPRK--------KPESIPE-----------IIRMESQTLLLTLLTVKMKKRLAVIEEKAEGNLIMLCEEKDRLQKKVQELKRKLIFHQKEQELMDILATQSELLTPFVTMSECFKKEYKTFATAVDSTRHELPVKSIHMEGDRDMFLD--NLQHQLRITQSLLAEVTTDHLGQNAKVLD--------------VLGEFKEVVRKKDLELKRSFAQVLNLSSEVS----KEAALTNQEVWEDTQGTQNLSQWYFNQE-----------------------------------------------------------  
------------------------------------------------------------------------------------------------------------------------------------------------------------------------------------------MEDGPSIPDNPKTQAKKTQGRLVKS-----RYM------------------------------------------------------------------------------------------------------------------------------------------------------------------------------------------------------------------------------------------------------------------------------------------------------------------------------------------------------------------------------------------------------------------------------------------------------------------------------------------------------------------------------------------------------------QYEKKAVGKTSVADQSTNCSGKSFSNAKIPTRKCKTTS-E---------------------------------------------------------------------------------------------STTKNNLQ----------------------------------------------------------------------------------------------------------------------------------------------------------------------------------------------------------------------------------------------------------------------------------------------------------------------------------------------ST--------LLEGHSIAHPDL-----------DLSAINDKSLFRGKVP---------------DVKHINTSKPLQT--DQNVGPKTL----KKEQTSTPEDI-----------IRMIESQTLLLNFLSLKMEKNLTRLEEKAEKNLLIICEEKDKFQQKVYEMKRRLQLRQRDQQLAEIV------------------------------------------------------------------------------------------------------------------------------T----KQVSLI---------------------------------------------------------------------------------  
-------------------------------------------------------------------------------------------------------------------------------------------------------------------------------------------------------------------------------------------------------------------------------------------------------------------------------------------------------------------------------------------------------------------------------------------------------------------------------------------------------------------------------------------------------------------------------------------------------------------------------------------------------------------------------------------------------------------------------------------------------------------------------------------------------------------------------------------------------------------------------------------------------------------------------------------------------------------------------------------------------------------------------------------------------------------------------------------------------------------------------------------------------------------------------------------------------------------------------------------------------------------------------------------------------------------------------------------------------------------------------------------------------LESETLLLTFLRLKIEKKVAKMEEKAEENLLMLCEEKRKEQEKLWELKREILLEEREQKLNETLEKQIEVLSPLIAVCEKFKEQYKSFAASLDATRHELPIKNIHIEGDKQTYLD--ELGKQLMITQELLTEVMPNHSEDSAKALG--------------ALKELQEVSQQLSKELQRSFTDVQNLSFEAS----KEVSLHNQYVCEENHGVDDVKRWYFN-------------------------------------------------------------  
----------------------------------------------------------------------------------------------------------------------------------------------------------------------------------------------MSALADGETSQNKRKGRRVVK------------------------------------------------------------------------------------------------------------------------------------------------------------------------------------------------------------------------------------------------------------------------------------------------------------------------------------------------------------------------------------------------------------------------------------------------------------------------------------------------------------------------------------------------------------------SRYLQYDKKGSSKNSSTKSFLSSTSSSSSTARTSSLLS-------------------------------------------------------------------------------------------------QKSETAGL----------------------------------------------------------------------------------------------------------------------------------------------------------------------------------------------------------------------------------------------------------------------------------------------------------------------------------------------AS------------------GLLNQSSFEKGDLQSTLLDGDKITRPDLDLS--------AINDKAVRQKATSPKASC--KTDKGTCVN----KQKSKNNPVVV-----------GAELGSQELILTYLGVKQRKDVAQLEEKAEENLLMLCEEKERQQEKLYELKREILLKEREEKLDEELVKQMELLSPLVPLLGRFKEQYKSFAVALDATRHKLPIKNIHIDGDMPTYLD--KLQEQLTVTQELLAEVMPDSSEESAKAFS--------------ELQDTEDVFKKLEKELQRSFTQVQNLSYEAS----KEVSLHNQRICEDNHGVEVVKHWYFN-------------------------------------------------------------  
-------------------------------------------------------------------------------------------------------------------------------------------------------------------------------MQAAEKTSLSKSNSLTNESIAAPPRPSSPKPSGVKP---------------------------------------------------------------------------------------------------------------------------------------------------------------------------------------------------------------------------------------------------------------------------------------------------------------------------------------------------------------------------------------------------------------------------------------------------------------------------------------------------------------------------------------------------------------RVGTPPRRSMAHQTPGTSMMSRENEPSLLGKSILQS------------------------------------------------------------------------------------------------------TFSDGHYF----------------------------------------------------------------------------------------------------------------------------------------------------------------------------------------------------------------------------------------------------------------------------------------------------------------------------------------------RP-----------------------------NFDISVIKEKTIVENAV-------------------------------EHDRNPENT--------------------------KRIVEMQTFLLAYITAKMESNTAKAEAEAEARIMQEMEEEEALHNEVQEKKRQYLLMEQDRLLNELLDLQIAALTPVVETAKQFTKDYKSFATAVDTTRHELPVQNFYIDGDRMEFLD--KAEACLKESEMLLLQCTDGDLKDNSTSLE--------------SLRDMKTTSKTISQQLSGTFSELLELSSLVC----RHTIHVQQATEEEQLGTARTHELFCPKR-----------------------------------------------------------  
----------------------------------------------------------------------------------------------------------------------------------------------------------------------------------MASKRSLYKPAPEESSAGAKHRRPLAQGRLATA-----EPATAVPNVPQEQLAQGSRNTADLTRQALSYRQSGTDDTISREDIFSTPRRNPPRGSMSPARPKPTMTDELAVSD-------------------------------------------------------------------------------------------------------------LESSIGENGSTQSKDSAPVLYV--------------------------DASRGPKSPENTEVDIVTSVVQS---PIKVVCHTPAAVQSDIRNTGDPRQTAKVNVDAVPSVLLEGDTDHTMGMTDLQQSVPRH------------------------KMPLVIRE---------------------------------KDIAGGAVDPFSSSRPVEVPEADTDEVPV------------------------------------------------------------------R-------------------------------------------------------------DSDSLSVRPKIRQQKQHGIIVESRYMQKAKAAVLPDKPKIGKS-T---------------------------------------------------------------------------------------------QSSAVQTP----------------------------------------------------------------------------------------------------------------------------------------------------------------------------------------------------------------------------------------------------------------------------------------------------------------------------------------------KP----------------APSRTTSTAKRKVAQSSSFSRDKSVRKESQHGS--------TPILAGNKRPTSTPVVAG--SKRAASTPM--GGTHPHTPGSMTL-----------GDISAIPTADSILVAPSTATSASKGKPKKKQYCYALWEKNEDLRKKITEGKQRIHCLKYQNLLDRMLKMQEAALQPLVDSAPKMQQDYSEFAHALDTTRHHLATISVSDDVDNEDYIK--KLYESVNEIEQLLGQLSVMTSRQLPKVTK-----------FSKELEELQKTTSAEGVLLKRMEELVAAAETIMT----QEVSLISQEVQLNRAGESSSDLTSQGLL-----------------------------------------------------------  
------------------------------------------------------------------------------------------------------------------------------------------------------------------------------------------MSVDDHRGSESTKKKKKVKGKFVQS-----RYMQ-----------------------------------SQAKPAASEPNVSMTGARPNVSGTRSNISGGTGA---------------------------------------------------------------------------------------------------------------RSNISGSRSNTSGAGARPASVS--------------------------------------------------------------------------------------------------------------------------------------------------------------------------------------------------------------------------------------------------------------------------------------------------------------------------------------------TTGVKLTRPGAVGTSRFKSSSSRTNNISVKRTDRSKSTSDKAV-----------------------------------------------------------------------------------------------PMRATRSF----------------------------------------------------------------------------------------------------------------------------------------------------------------------------------------------------------------------------------------------------------------------------------------------------------------------------------------------AP--------APSTTTTGPAVASSHPRQHPSTGGDSQFSTPVTAKKGKRFA----------STPAVNTSMLPGASHI--NASAIGAST-----SILGANMSAI-----------AHVDSSKLMPDKMRDSHDIRDRSRRERGMSGDMTGPYSGLS-VSPNHDSAYSSLGHKMKKIVADKEKETRETGLGPVTANLAQFKQEHSTLAHALDTTRHQVEMTDILLPQDKDAYHD--ALERVLSESEHLLGEISMATRNKQPRVEA-----------FSSAISMLENTVASESEELRRCQELLAATSSLAT----HETSLRVQDLEQRSES-----------------------------------------------------------------------  
--------------------------------------------------------------------------------------------------------------------------------------------------------------------------------METLKEFRSTVDGKKPELARALFSGSMNESREVSI----------------------------------------------------------DSKRHSIDDESRVSESPAKSID-------------------------------------------------------------------------------------------------------------CSSEVKKKKKKAVRLIASRYMS----------------------------------------------------------------------------------------------------STSRPSAPTS------------------------HSTSNHRQPMRATDRDILKK----------------------------------------------------------------------------------------------------------------------------------------------------------------------------------SVPCKRPQPNVLAPKRTGGSARKLAPKVTRHRSVERTPDVSKP-------------------------------------------------------------------------------------------------PGPKAS----------------------------------------------------------------------------------------------------------------------------------------------------------------------------------------------------------------------------------------------------------------------------------------------------------------------------------------------TP--------EIEMSSHLAVDISAIDSHHSHHSHSSHLSTPGAPPQTNCKS---------------NRSNSTSMKKA--DESSLPSSK------TEEFSQTH------------VDILYARYLQTVYLEARAKHDFKLQEKHSMSQLHGLWLQNEQLQKRSAELKLQLSKLKHLNSLDTAVDDQLRGLSPVLPHLDVVQQQYSALAGALDTTRHQLSTKDVYLPDDELSFHE--NLTAALLESERLLGELSVMIRTKTPEISR-----------FAQTLSSCTETVMKQLRHIDTCSDLVSSAHKLST----QECSLKIQDMQLKDIH-----------------------------------------------------------------------  
------------------------------------------------------------------------------------------------------------------------------------------------------------------------------------MASKGKRDLYKPAQKSVPKRNRKPLAQG--------RLVTEPVCNIVKKTLSGAQVSDDIEDHDTDTLSVTPSKLTPSYHDQVSPQPTSSLDLSDIDTEVIDDINKSVIVM-------------------------------------------------------------------------------------------------------------DATENLLNPENSAEVSVNIGMK-------------------------------------------KSPVRV---VCHLDTDALRERNDNVVVEKKSRSVTKVKQSSTLQAISPLHKDRPKSASKGRTPVRKI------------------------AGTPSKIKPNYPQPNFNDSDESGSMKGDIEDDLQSIISETDTVCSIDEYIPVIKSKALQNQKTSSKGPKM------------------------------------------------------------KMKTAPT-------------------------------------------------------------IIPSRYLQVSSKSSASFNEKPTSRVKKSSNKSLPVTKSASNSR-T--------------------------------TPTLPR-------------------------------------------------------KNLASTTV----------------------------------------------------------------------------------------------------------------------------------------------------------------------------------------------------------------------------------------------------------------------------------------------------------------------------------------------SN--------NQQQTRAVTPSQLSSQAGGGKTSTPCPDNLPNQTQDIDASAIHAEISAISADLSAMNSTRKVNSPYL--NRKQRPNKK-------DIKSSQQR-----------LEVLYSRYLQWLYLDSKSKMVFKEQEKTAMSQLNALWEEVESLREKESALRLDLVRLQHLNQLDDQLEIQKNGLGPVMTNLPVIDKDYNKLAESLDTTRHQISTRGIYIPEDEDTFQA--ALESSLKESEYLLGEISCLVRQEVPKTSL-----------FSKSLQTTQKLVDTECTELKKCTELLSAIQSLTV----QESSLKIQAIQSS--------------------------------------------------------------------------  
--------------------------------------------------------------------------------------------------------------------------------------------------------------MAADKRSLYQLAPTQTPSPHVRKTLVQGRLESQISSPVRLQSPRSFLGYPQEAYDPIAQVSRQSEMPGTESIGIHSVIPNIRMSLGLNNTSQQNPKISQSTQQSLLSHPIPVVSPSALQPSSSLSLSDNDDEN-------------------------------------------------------------------------------------------------------------LAQPLPTTPPKSPKQGTSQPVI------------------------LTDASSRHKTPEKREVPVVTSVVTS---PVRVVCHSPRRDKGVTSNGTSVTGHTESARKVEVAGVQRINVERRKEANNWAAPLNIS------------------------QDSLDSDDDERHIPTIAGSPVDPHSKAIRVALDKND----KKNGVSDPPYDPQQYKSFMGEVDENQGGPV----------------------------------------------------------LAGFNNVKQ-------------------------------------------------------------NLFDYSVADVSIASDTSKVSETGEKKKKKSKTRIVPSRYMSTS-A--------------------CQSSAMKVASKKTTSVQRKQKVSFASTSKTKN-RSLHDSSKLDITAPL-------------------------QNPNARGN----------------------------------------------------------------------------------------------------------------------------------------------------------------------------------------------------------------------------------------------------------------------------------------------------------------------------------------------NI--------MTSTPAVGSIGIIPGHPGGDLAAPTPILPPQSVYNTGGLGY--------KDKTKKTQKIRAQPFDNI--AAEGTHPQE---APAGQGEVSQRQ-----------LELQYARLVQWIFLESKAKSSFAAQEKQAQSQLYSMWQENEKLRRGIAELELEVEAQQKMEELDKQLNLQQSGLEAVDVNLPKLKQEYPKLSNALDTTRHYMPVNGVLIPDSHG------ELLGALDESENLLGEIEALTRHQTNKVSN-----------FSKEISTLDKITGTEVEEHSRCQELLAATLTLAT----QERSLQAQIIQSS--------------------------------------------------------------------------  
-------------------------------------------------------------------------------------------------------------------------------------------------------------------------MSSSFNKLSLYKPAAVSERFMSPELVNPRNKEPMPSPSLHST--------------------------PPPPTQSLSLSDNEHSLLSNEAPLNSTLQNSRISSASAGDDKTMLMVQTIESQT-------------------------------------------------------------------------------------------------------------YLTSPEVQVNTIETPSLARVVR--------------------------------HSPPPLPPPSLPVSNSR---QHRFSSNFVPLSSFKEPLSASKDGGT----------------------SVREKPSLSL------------------------LQHIEEGNTDLSDEEVDGAG---------------------TNLVSATSSSSTLKELLGDKEHQNNGGHE--------------------------------------------------------------------------------------------------------------------------------EIKPTPVESLQEQASTDVKENEGKRPKKKSSRVVASRYMSGIS-S---------------------------------------------------Y-KKNQFPP---------------------------------SNTSSVIT----------------------------------------------------------------------------------------------------------------------------------------------------------------------------------------------------------------------------------------------------------------------------------------------------------------------------------------------KP---VKRGTLSQSVSNVSFNPTHRHTSTPAVKPAPVLKTNELSKRTATTN--------QTNQMKKRQQQQQPLQHS--NRTANPAIR----VSGKPLDPTSK-----------QGLLNARLLQMTYLRVMALKNKRAEESQAQLQLYDLWEATEEKRREVTELKKEIEYCARTQAIDESLSYQLKGISELEEKLAAIEPDHKTLSESLDATLHELPTKDILIPNED-------ELVHGISEAESSSKEIHLRIGDSALTVDT-----------LAENVESLAKIVKKEQNETPGALA-----------------------------------------------------------------------------------------------------  
------------------------------------------------------------------------------------------------------------------------------------------------------------------------------MADQARKKQLYRPAVLHQHIPSSPTRKTLSQGYLIAQISNGDNAFPEDQNKTETKRSLFQSVNTSVQPIVKGNDNYQDDVSLFSDEEESQQDPKDLTVIEGTALSKDAMRSNIQSNI-------------------------------------------------------------------------------------------------------------MANQNANDERYRDTSSDDANFS-----------------------------------KQTRAALSSLMANS---PTPKVGNSPVLIVDALGRTADPQATEVAVVTTVAKSAA----------KVVCHSPSEK------------------------NSNKDDSVHVTAVPRVRIKIADRIAEEERKHEEVVD---NVLALESASSVDNTKVKINDIEAQARYVASR--------------------------------------------------------------------------------------------------------------------------------YLSGISTNRTKTTTVGKSHSKAEKGPMIGRHDLHATRTTPKTS-N---------------------------------------------------------------------------------------------TGITAKSF----------------------------------------------------------------------------------------------------------------------------------------------------------------------------------------------------------------------------------------------------------------------------------------------------------------------------------------------SR------------LGKKQDVMASTPSDRQQASKLRTVRTPQDFSIRRSRN--------TKTPIASASKRKPPIKVL--PTDKKETANTTVDNKAETRIQMEKKKGSIEVTQSELDLVYARLMQWVFAYQKLKHTVNTQEKEAMNQIFSMWKYLHQQNRKLTQLKLELQLAKHTSLLDDVLDMQKSELQVIGDLLPRVKSDYSNLAKGLDAVSHVLRTKGILYMDDHD------EVISALGESEELLGEIISITRHNQENVTA-----------VSQSMCGLADKATVEGKELHRCEELLGALSTLVV----QESSLRVQEIEESRADSNEIDNLSLHSMHF---------------------------------------------------------  
----------------------------------------------------------------------------------------------------------------------------------------------------------------------------------MSGRQAPASSSAAAAAAIAARYSYTGGSSGSSSGASTTRTKPVDPGSARKPRLSREALAERLAASRAALLANRANAAPAPAPAPDDPPPSWIASSSSTNGTTLAASSALPPPS-------------------------------------------------------------------------------------------------------------ATKSKTQASSAPTTQLSAATVA--------------------ALARARLSVQANESSSSRTLPTSSRAPRP---TSPAASEASDVSNVSSMSFPSNSNAASSSATAQINQRGREIAAKYLARSAAGSGTSAP------------------------SASSANRPPAGAPPGSASRSRQLFMQPTQASQASQG---PITSSSSGLKTPQSARKSLGAVNAGSVSLAV------------------------------------------------------------------P-------------------------------------------------------------STPQSARKTSTVSSTPQSVSSRSSAAILGARAAPATGGKAVLP-P------------LSVSRPAAVAKTPSSAAQPSLSRPRSSSISSQSYSLPTAS-SSLSHPAKTPTLASRSGLGSAATAKTPKT-----------PNLDESSM----------------------------------------------------------------------------------------------------------------------------------------------------------------------------------------------------------------------------------------------------------------------------------------------------------------------------------------------AN--------APRSASRRPALPVNRALFAGSQTPTSNKAPGAGARATSSSA--------AAAGSARPSSAASRSSHA--SLPVAPMPT-TTSSGKLLITLKNE-----------IELLQCELYQWSYIASASQQAFAAREVQAEQQIYALWQRVEQLRHDVSLLRQQLWSIEHTQRAEELVMSEHDLLIQLEAELSDFVSAYTALANSISATTHTIPTRGIHLGNQDDIAIE-------LRQSAALLSELTQISAVDQQPIHE-----------LAIQSAVLHDTITAEADSLRKCREVMAALEYLAA----EEVSLRLSEVKFTAS------------------------------------------------------------------------  
--------------------------------------------------------------------------------------------------------------------------------------------------------------------------------------MDPRRRAGPMQPHLRVASGLGSAGGKVSK-----GVIQKP-----------------------------KQPKPRSTETPPSSHPSPSSPHTSSSPDTPSPTLRLQRSR-------------------------------------------------------------------------------------------------------------MSSPSGTPRYMSSTQSFAAKVD------------------------------------------------------------------SSPRPGSPRGG-----------------------SPHASSPSRS------------------------SRNTTPRAIVRDSPASVDLQVDVSPVSSVADSVRGE-----GRKRKVRYVASRYMASTTSAVSKPAPQPV----------------------------------------------------------------------------------------------------------------------------------HRPVSKPVTVRSTLTKSVVESRPERPNVRKVLGEQNLLRER-N------------------------------------------------------VMATPG---------------------------------VELKRKSM----------------------------------------------------------------------------------------------------------------------------------------------------------------------------------------------------------------------------------------------------------------------------------------------------------------------------------------------GP------KSVVKGGLRASELDAPRSSLRRTMAETRPSKSSSQMLAKAAAV--------PLPASPVPSLAPPSARKQ--SSLTQSNPS----RTNSSTPIEDT-----------ILALENRLLQWTFLRANAQKAFEEKKRQAEAEIYTFWQRVTELREEVHGEEETVRQMEELVKVIGPLREQ-------EKAITEAAQALEAFSKGYDTFMNGLRRSVDWLPMKGVMISDAEELRNVLKDTTEEMRHVLQDENGHIEQIAM-----------LSNRLAALRDAIQQTTTEMDECVKLAQELARMEL----VEKSFVIGRLQMNEKKREGWAWEGD--------------------------------------------------------------  
----------------------------------------------------------------------------------------------------------------------------------------------------------------------------------------MATTGRRLRPPSPNNNRSRTISSSISL-------------------------------------------------------PVSLNASLSSSTSSSSSSSPSNSSK-------------------------------------------------------------------------------------------------------------RVMITRSQSTTRSSRPIGSSDS----------------------------------------------------------------------------------------------------KSGENIIPAR------------------------------------------------------------------------------------------------------------------------------------------------------------------------------------------------------------------------------NSASRSQEINNGRSRESFARYLEQRTRGSPRSNASSRGVKPGA-----------------------------------------------------------------------------------------------SSPSAWAL----------------------------------------------------------------------------------------------------------------------------------------------------------------------------------------------------------------------------------------------------------------------------------------------------------------------------------------------SP---------GRLSTMKTPLSSSAPTTSMCMTPPESPVSKAKIRSGGGGA------------------VAGVLKYF--MAQKKVSPV--------QEEDYHR-----------FRIFQNRLLQWRFVNARTEATMANLKINVEDQLFWVWLRIYKMRNYVVENLIEIQRLRQDIKVREVLSLQ-------MPLLNEWSKIDAKNSEALSKLTRKLHALSVRLPLVHGATIDMVSIHEEMVIAIEVMDEIEDVIIKFLPRQVE----------IILYELTELIGMFNQELLYFEEMDESLLSIPLFTA----KESSLRVHILQKTEEQR----------------------------------------------------------------------  
----------------------------------------------------------------------------------------------------------------------------------------------------------------------------------------------MKSCEHELLKTRRGKSREVSS-----RFLS---------------------------------------------SPSASSSPNRRNSTSNSSRDDQNNNG-------------------------------------------------------------------------------------------------------------VKGHLGLKKHDRMSDGTRVCFG---------------------------------LPNQSSIEVDTKENRM---PSPWI-------------------------------------------NDEDNVILPG------------------------RFSVDECALYRASSRRNSCS-----------------------LLYESFNDETDSELSDVSCASSLSTNR----------------------------------------------------------------------------------------------------------------------------------SSWNHKPGIKVSSKYLHDLTAKPSKGNNKTKLRSQDDSQ-------------------------------------------------------------------------------------------------RTNSSKGI----------------------------------------------------------------------------------------------------------------------------------------------------------------------------------------------------------------------------------------------------------------------------------------------------------------------------------------------EN----------RLQRNNSVSRYGSSMSQWALSPGRSLDTQAVTVPSSKLK--------PPRGKGVGKLINLGFDFF-RSKNKSSPFTSPLKPKTCDTESAHQ-----------LKLMNNRLLQWRFVNARACDVNKNVASQEKNQLLCAWDTLIKLNNLVLQERIKLQKKNLEMKLNYVFLSQ-------VKHLEAWEDMEIQHLSSLSIIRDSLHSVLSRLPLKEGAKVNLESAVSIIKNAEAVTDAIISTVDDYAPTMEG-----------IVPLASQLAEVVVQEKLMLEKCHDLLRMISELEM----QERSLKCCFLIQHKQTFDTNLLKH---------------------------------------------------------------  
-----------------------------------------------------------------------------------------------------------------------------------------------------------------------------MEARIGRSMEHPSTPAINAPAPVPPPSTRRPRVREVSS-----RFMS----------------------------------------------PISSSSSSSSSSSAGDLHQLTSNSP-------------------------------------------------------------------------------------------------------------RHHHQHQNQRSTSAQRMRRQLK-------------------------------MQEGDENRPSETARSLDS---PFPLQ---------QVDGGKNPKQ------------------------HIRSKPLKEN------------------------GHRLDTPTTAMLPPPSRSRL----------NQQRLL--------------------------------------------------------------------------------------------------------------------------------------------------------------------TASAATRLLRSSGISLSSSTDGEEDNNNREIFKSNGPDLLP-T---------------------------------------------------------------------------------------------IRTQAKAF----------------------------------------------------------------------------------------------------------------------------------------------------------------------------------------------------------------------------------------------------------------------------------------------------------------------------------------------NT---------------PTASPLSRSLSSDDASMFRDVRASLSLKNGVGLS--------LPP--------VAPNSKI--QADTKKQKK-----ALGQQADVHS-----------LKLLHNRYLQWRFANANAEVKTQSQKAQAERMFYSLGLKMSELSDSVQRKRIELQHLQRVKAVTEIVESQ-------TPSLEQWAVLEDEFSTSLLETTEALLNASLRLPLDSKIKVETKELAEALVVASKSMEGIVQNIGNLVPKTQE-----------METLMSELARVSGIEKASVEDCRVALLKTHSSQM----EECYLRSQLIQHQKKCHQQECTTSV--------------------------------------------------------------  
-----------------------------------------------------------------------------------------------------------------------------------------------------------------------------------MEAKTSGPKQIQPSTPAPPPSTRRPRVREVSS-----RFMS---------------------------------------PVTSSSSSAGDLHSLTCNSPKQHHLQHHQIQR-------------------------------------------------------------------------------------------------------------SVSAQRLRRQLKMADGDENRSS----------------------------------------ETAARSLDS---PFTLS-------------------------------------------QSRKSSKPSH------------------------LKPLNENSHRLETPT-----------------------------------------------------------------------------------------------------------------------------------------------------------------------------------------PMVPPPPSRSRLSQQRLPTATRLLQLSGISACYEKEGIINI-Q---------------------------------------------------------------------------------------------EKPKSNGS----------------------------------------------------------------------------------------------------------------------------------------------------------------------------------------------------------------------------------------------------------------------------------------------------------------------------------------------DQ--------FPTLSCRTHLKVFNNPVPSSLNRSVSSPSSSCNVRESSSFS-------------RLGLPLPPMAPKV--PADTKKQRK-----VTEQLEDVHS-----------LKLLHNRYLQWRFANANAQVKTQTHKTQTETMIHSFGSKISELHDSVQRKRIELQRLLKTKALLAITESQ-------TPCLEQWSAIEEEYSTSVSQTIQAFSNASLRLPLDGDIMVDSKQLGDGLVAASKIVDGITQNVGNYMPKAKE-----------MESLLSELTRVARSERSLTENCVVALLKTQASQI----EECSMRSQLIQQTTKEESSSK------------------------------------------------------------------  
------------------------------------------------------------------------------------------------------------------------------------------------------------------MQVGSKKASMGKQQQSVSDATSPRPPLAPSEKNNVGSVTRRARTMEVSS-----RYRS-------------------------------------PTPTKTRRCPSPIVTRTAPSSSPESFLKRAVSAE-------------------------------------------------------------------------------------------------------------RNRGPSTPTTPVSDVLVDLPVS--------------------------SRRLSTGRLPESLWPSTMRSLSV---SFQSDSVSVPVSKKEKPLVTSSTDRTLRPSSSNIAHKQQSETT-----SVTRKQTPER------------------------KRSPLKGKNVSPGQSENSKPMDGSHSMLIPPQHRWS---GRIRGNRSFDLGDKAVRRVSLPLSNKSSRHK------------------------------------------------------------------K-------------------------------------------------------------SSSDITRLFSCYDNGRLEVSSSTTSEDSSSTESLKHFSTSSLP-R---------------------------------------------------L-HPMSAPGSRTASPSRSSFSSS-------------------SSSNSRGM----------------------------------------------------------------------------------------------------------------------------------------------------------------------------------------------------------------------------------------------------------------------------------------------------------------------------------------------SP--------SRGVSPMRGLSPVGNRSLVRSSTPPSRGVSPSRIRQTAQSS-----------------STNTSVLSF--IADVKKGKK------ATYIEDVHQ-----------LRLLYNRYSQWRFANARAEGVSYVQSLIAKETLYNVWHAISDLRDLVTTQRICLQQLKLEIKLRSILNDQ-------MVCLEDWAMVEREHISSLAGAIGDLEANTLRLPLAGGTKADLGSLKLAMSSALDVMQSMGSSIWSLHSQMEE-----------MNKLVSDLAVIAKTENFLLDKCENLLASTAVMEI----EERSLKTHLIQKKQEEEVRDDAESSPLLPLSKFQWP---------------------------------------------------  
---------------------------------------------------------------------------------------------------------------------------------------------------------------------------------MDVATDTTRRRLLPSDKNNAVVATRRPRTMEVSS-----RYRS-------------------------------------PTPTKNGRCPSPSVTRPTVSSSSQSVAAKRAVSA-------------------------------------------------------------------------------------------------------------ERKRPSTPPSPTSPSTPIRDLS--------------------IDLPASSRRLSTGRLPESLWPSTMRSLSV---SFQSDSVSVPVSKKERPVSSSSGDRTLRPSSNIAQKHKAETT------SVSRKPTPER------------------------KRSPLKGKNNVSDLSENSKPVDGPHSRLI-EQHRWP------SRIGGKITSNSLNRSLDLGDKASRGIPT-----------------------------------------------------SGPGMGPSLRRMSL-------------------------------------------------------------PLSSSSRPLHKTSSNTSSYGGLVSPTKSEDNNIARTSGAQRLL-S-----------------------AGSLDRATLATAVAR-------------L-HPLPAPGSRPASPSRTSFLSSSSISR--------------GMSTSRGV----------------------------------------------------------------------------------------------------------------------------------------------------------------------------------------------------------------------------------------------------------------------------------------------------------------------------------------------SP--------SRGLSPSRGLSPTRGLSPSRGLSPSRGTNTSCFARPSTPPSRGISPSRIRQTTTSTQSSTTTSVLSF--ITDVKKGKK------ASYIEDVHQ-----------LRLLHNRYLQWRFAIARAESVMYIQRLTSEETLFNVWHAISELQDHVTRQRIGLQQLKLEIKLNSLLNDQ-------MVSLEDWATLERDHVSSLVGAISDLEANTLRLPATGGTKADTESLKAAMSSALDVMQAMGSSIWSLLSKVRP-----------MNIMVTELAVVVTKESSMQGKCEDLLASTAIMQI----EECSLRTHLIQTRREEGEDAETPPPLLPLSKFPWP----------------------------------------------------  
----------------------------------------------------------------------------------------------------------------------------------------------------------------------------------------------------------------------------------------------------------------------------------------------------------------------------------------------------------------------------------------------------------------------------------------------------------------------------------------------------------------------------------------------------------------------------------------------------------------------------------------------------------------------------------------------------------------------------------------------------------------------------------------------------MNAALSSPRGTSIARGLSPSREVVP---------------------------------------------------------------------------------------------------PRGV----------------------------------------------------------------------------------------------------------------------------------------------------------------------------------------------------------------------------------------------------------------------------------------------------------------------------------------------SP---------------------------------SDRMSPLRVRSSLSKN-------------------TPLIPHF--AVDGKEKIR------DNGVADAHL-----------LRLLHSRLLQWQFANARANAVISSQKMREERRLYNAWRSISNLYNSVSMKRIEMQHLKQNLKLISILNMQ-------MGHLEEWLVIDRNYMGSLVGAAEALKGSTLCLPVDCGAMVNVQSVKDAICSAVDVMQAMASSICLLLPKVGK-----------ISSLAAELGRVNAKDEGMLDVCRDLLNTISALQV----TECSLRTQVTQLQ--------------------------------------------------------------------------  
-----------------------------------------------------------------------------------------------------------------------------------------------------------------------------MVAAAISTTDPRNPPRDRPQSLTNNGGQRRPRGKQVPS-----RYLS-----PSPSHSVSSTTTTTTTTTTTTSSSSSSSSSAILRTSKRYPSPSPLLSRSTTNSASNSIKTPSLLPK-------------------------------------------------------------------------------------------------------------RSQSVDRRRPSAVSVTVGTEMS----------------------------------AATKMLITSTRSLSV---SFQGEAFSLPISKKKETTSTP---------------------------VSHRKSTPER------------------------RRSTPVRDQRENSKPV--------------DQQRWP--GASRRGNSESVVPNSLSRSLDCGSDRGKLGSG---------------------------------FVGRSMLHNSMIDESPRVSVNGRLSLDLGGRDEY-------------------------------------------------------------LDIGDDIQRRPNNGLTSSVSCDFTASDTDSVSSGSTNGVQECG-S---------GVNGEISKSKSLPRNIMASARFWQETNSR-------------L-RRLQDPGSPLSSSPGLKTSSISSKFGLSKRFSSDAV----PLSSPRGM----------------------------------------------------------------------------------------------------------------------------------------------------------------------------------------------------------------------------------------------------------------------------------------------------------------------------------------------AS--------PVRGSAIRSASP-SKLWATTTSSPARALSSPSRARNGVSDQ-----------MNAYNRNNTPSILSF--SADIRRGKI-----GEDRVMDAHL-----------LRLLYNRDLQWRFVNARADSTVMVQRLNAEKNLWNAWVSISELRHSVTLKRIKLLLLRQKLKLASILRGQ-------MGFLEEWSLLDRDHSSSLSGATESLKASTLRLPIVGKTVVDIQDLKHAVSSAVDVMQAMSSSIFSLTSKVDE-----------MNSVMVETVNVTAKEKVLLERCQGCLSRVAAMQV----TDCSMKTHIIQLSRIPITSSLTPQL--------------------------------------------------------------  
----------------------------------------------------------------------------------------------------------------------------------------------------------------------------------MVAAIPQGAAISNTDSKNPPPRDRQDKPQLTANNGGLQRRPRAAKNVPSRYLSPSPSHSTTTTTTTATSTSTSSSSSVILRSSKRYPSPLLSR---TTNSASNLVYTPSSLPK-------------------------------------------------------------------------------------------------------------RSQSVDRRRPSAVSDTRTEMSA-----------------------------------ATKMLITSTRSLSV---SFQGEAFSFPISKKKETATP----------------------------VSHRKCTPER------------------------RRATPVRD-----QRENSKPV---------DQQLWP--GASRRGSSESVVPNSLSRSVDSDSDDGRKLGS---------------------------------GFVGRSMLQHSQSSRVSGDGRLNLGFVGGDGMLE-------------------------------------------------------------MRDENKARQSTHPRLASSVSCDFTASDTDSVSSGSTNGAHECG-S------------GEVSKTRSLPRNGMASTKFWQETNSR-------------L-RRMQDPGSPQCSSPSSRISSISSKFSQSKRFSSDSP----LTSSPRGM----------------------------------------------------------------------------------------------------------------------------------------------------------------------------------------------------------------------------------------------------------------------------------------------------------------------------------------------TS----------PIRGATRPASPSKLWATATSAPARTSSSPSRVRNGVSEQ------------MNAYNRTLPSILCF--SADIRRGKI-----GEDRVMDAHL-----------LRLLYNRDLQWRFANARADSTLMVQRLSAEKILWNAWVSISELRHSVTLKRIKLLLMRQKLKLASILKEQ-------MCYLEEWSLLDRNHSNSLSGATEALKASTLRLPVSGKAVVDIQDLKHAVSSAVDVMHAMVSSIFSLTSKVEE-----------MNSVMAEMVNITGKEEVLLEQCQGFLTRVAAMQV----TDCSMKTHIIQLSRL------------------------------------------------------------------------  
-----------------------------------------------------------------------------------------------------------------------------------------------------------------------------------MSSSSSSRRWASASVTDLSSAGRSPLPAAALS---------------------------------------PVRPSARRSPAVSRPDPAPSIARTIWPSSSSSNSGNTSTRK-------------------------------------------------------------------------------------------------------------ASPSPSSPAPAASTPSSSSSVA--------------------------------TTLADHLAEDSLDAPPA---ALSRQRSCTELPRFADADAEARKVVVARS-------------------GGHASAIGRS------------------------MRLLPSTRPAGVTLTPGR-------------------------------VAPSDLRRLDAGADVAS--------------------------------------------------------------------------------------------------------------------------------------SGSECSDASRGGGGSTPRTTTKLPKPPPSPLIARTNSTRLL-G---------------------------------------------------------------------------------------------SSNTQWAL----------------------------------------------------------------------------------------------------------------------------------------------------------------------------------------------------------------------------------------------------------------------------------------------------------------------------------------------SP-----------GRRSGSPLKTTLATVPELKGKTKSLIGLGWGHLFSRRK--------AAAAETATGAQATATLSS--PASRRSGGG-------GNREIGHQ-----------MKMMHCRLLQWRFANAKAEAVSKNKLSIFEPKL-SASNTCSSVNSEI--LKPPLEIKHQFIKWWNSWVHGLGYQSCKMRGLESWGQLESKHAVALDSTVVCTQAAICKLPLTNGAKISLPSMGIILQQALDLTMTTKTIVRSFTPMAHD-----------TTLLITELVAVAREEHALLQECLELLGRVSALQTIVALEINELVNDIIVSYRRGHSALALPPPPDLTYRRRR-----------------------------------------------------  
----------------------------------------------------------------------------------------------------------------------------------------------------------------------------------MAIAAPPPPPPPPQRTDMPAAADLPPPPPPLAI----------------------------------------PLPETTRRPRRRTREVSSRYLSSTTPGPVPSSPRLSTSSS-------------------------------------------------------------------------------------------------------------RTPSPRAHRPRAATPFANENHP--------------------------------PPPPPPSTASRRRAVLK---LFDDGSGGANPRASAAAAAGTPR-------------------------ALHRSTSGPA------------------------AAAASTARRGYPRMPTPAR-------------------------------------------------------------------------------------------------------------------------------------------------------------------------------------AASCPSSSSAAAADDAASCCSSDTGSTFTDLSEVDGIALPA-A---------------------------------------------------------------------------------------------PCESPPLL----------------------------------------------------------------------------------------------------------------------------------------------------------------------------------------------------------------------------------------------------------------------------------------------------------------------------------------------GP-------ASCRGGRLSSELRSSVPESGGSVRALNPLCYRSLNSALSGCP--------APAGKAAVNAARPPQPHGVKAAESKKVAMIGGRKVPGKQEDVHQ-----------LRMLENSYLQYRFMNARAEAVARAKASVAEKSLFGLEERITALRVSVAEKKMEVERMRREQTLRSVVDAQ-------VPHLDQWCDLEGDHSSSLIGLTSALYNSSLRLPVIGNVRANSEEITEVLNSSVQLLEPVSSCVKNFLPKVQE-----------VDDVAAKLAQVIASERVAIEECGNLLYQAHNLQM----REYSLRSQVMQLKQQDEPK--------------------------------------------------------------------  
----------------------------------------------------------------------------------------------------------------------------------------------------------------------------------------MESNGVGGGAARPLTASRRLLARSAST------------------------------------------------------------TASRAGGAGAFVYDGMRPAP-------------------------------------------------------------------------------------------------------------LF------------------------------------------------------------------------------------------------------------------------------------------------------------------------------------------------------------------------------------------------------------------------------------------------------------------------------------------------------------------SSTNFARSLRKAASFGGGGKKQYSADDDGAVAVKAAAPPRR-A---------------------------------------------------------------------------------------------LSSKENTV----------------------------------------------------------------------------------------------------------------------------------------------------------------------------------------------------------------------------------------------------------------------------------------------------------------------------------------------HE--------------------------LGTAAARGPWEPARRPRRSSSGG--------SSSPENAGSTRGSAVLRD--MMTRRKEEP-------EKEEAAHR-----------ARMLAARLLQWRFANARMEKAMARATAAAENKLFYTWLRVAELRNIQAAKRIVAQRRRQKLKLARLLRPQ-------LSLLASWDSLAKPHADAVDDLGAVLAAACTALPLADGAQGDMESLHEAMFACVGTVNDIEANADMFFATAGV-----------TSSTLEELSTTIKQEVEGLQEAMKLARIVTSLQV----QEVSLRANLIQIQAKQKVDMGASVPAIATSGWCF-----------------------------------------------------  
------------------------------------------------------------------------------------------------------------------------------------------------------------------------------------MAADAAARAVSVAFQDASYCLDGGKAKQVPH------------------------------------------------------APSPEKKRASFAAGAAAVRAKVCDAR-------------------------------------------------------------------------------------------------------------WPASAAAANSAPYGFRGGVATR----------------------------------------------------------------------------------------------------SVAFDEMTPR------------------------RASVDVPNPLRAALSSDDTE-----------------------------SATSSAGSPDGDAD-----------------------------------------------------------------------------------------------------------------------------------------ADAKLAARARPSPRSIMASPARFSRDAMGSRSERFADHSTP-F---------------------------------------------------------------------------------------------MSRTPRFL----------------------------------------------------------------------------------------------------------------------------------------------------------------------------------------------------------------------------------------------------------------------------------------------------------------------------------------------AS--------PSPKTTPTAPPPPTTTKKKSVKSLFNGLLSSPFTRPSPKQP--------PPTKPAAISPASPSPARC--SATAAASAVPGRLQAQGKAEEEHQ-----------LRLLHNRHLQWRLANAVAGAAISAQELNAEKQLCGAWVSILGMSKSIALKKLELQLLRQNCKVMNTLKGQIYLPVQMMAYLEEWSLLENKYANSLSGTVEALNATVLRLPVSDGAVADFQSVKNAVGSAVDVMQTMRNSMSYLLPKLAR-----------TNVLVSQLSIITRQEQVLMAQCRELLSTLALMHV----KYSSLQGQMIQLSDLKRAKSVSSSEYPY-----------------------------------------------------------  
------------------------------------------------------------------------------------------------------------------------------------------------------------------------------MVAAGAAAAAAAAPRVNPSPSPHRRRASSALSPSKSA-----NSNANANADAARGGGGGGGKPKGKAVPSRYLLAPSSKSTSTSSSSTTTTNSSATSNSTSTSASTPSRRFASPLPR-------------------------------------------------------------------------------------------------------------RSSSVDRPRPTSNAAAAGGDAL----------------------------------GPNGATTTTTRSLSV---AFQGRAYFL-----ETSKAKPATSP-----------------------SPVRRPVAAA------------------------STTPERRRPSMGTVPERGKVFEGGH-----SQQRWP----MSARAAHGFEGNPLTKSLDCSLDKRGAAVL----------------------------------------------------------AAVRSLRQS-------------------------------------------------------------MVFEEGVRRASFDSGDYLMSSDTESVSSGSNSGSQDAGMGRAR-S--------------------SPKGMSVPARFLQDAAASR------------P-NRLADPSTPFM-----------------------------THSSGFAS----------------------------------------------------------------------------------------------------------------------------------------------------------------------------------------------------------------------------------------------------------------------------------------------------------------------------------------------SPRTAPVKKSLLNGFVSSPLNRPIRQPSPSKLVGSRRMSSPSQPRGSVGVS--------ASYGDQHGRSSSGYGLDS--QVKRRWLGC-------SKVDCEHL-----------LRILCNRHLQWRCVNAQADAALAAQKMTAEKYLSDAWITTLGMRKSVALKRFQLQLFRNNWKLMTVLKGQ-------MDFLEEWSFLERDHANSLSGIVEALTATILCLPVTDGAKADIQDVKNAVGSAVDIMQTIGSSICTLLAKLSG-----------TSILVSDLAKIATQERTLMDQSRELLSTLASMHV----KYCSLQGQRVQTTTHRRRVRS------------------------------------------------------------------  
--------------------------------------------------------------------------------------------------------------------------------------------------------------------------MDVLKSDVKKTGILNETLRPPLVPSEKHNASPVNRGRDVAS-----RYKN--------------GLSAHSAATTARRCTSPSPGRTSANECTPEPKRAQSADRRRPSTPSSRVSTPSTPAS-------------------------------------------------------------------------------------------------------------RSVTPVRNTVTEGHKSSRRITS--------------------------------TRNTDGLWPA-MRNLSS---SFQSESVVTPGNKKDKVVPSGSLDQTKGQASVI---------------AERKRSPLRR------------------------KNIGEQCENAQPSEDQPRRVI---------EQHRWP------AMQSGRVASNILSRSIDMSDKAGRSVPS---------------------------------------------------TNISRGVSPRKTLASE-------------------------------------------------------------GTGKGFNKSLDEVARRLAIHAGGRDDKVDSRCHAYSQSTERCK-S---------------------------------------------------------------------------------------------VSRPSRAV----------------------------------------------------------------------------------------------------------------------------------------------------------------------------------------------------------------------------------------------------------------------------------------------------------------------------------------------TL-----------PVPVLHRSSSPSKASSVTSSISRSFQSPSRTRPSTPSR--------SQSAGSIQSGVASPIISY--MVDAKKGKK-----NSSQIENIHQ-----------LHLSYNRYLQWIFVNAYAEDTMSFQKVTAESIIYNVLRNTSNLRDVVNMRRIMVQCIQQELKLHGILKEQ-------IDYLEQWPALEKENSISLFHATEALKASTLRLPVTSGAKADVVALKNAVSSAVDIMQGLGSAVRCMLPKVED-----------RTYLVSELSVIARQEKAMLDECRELLAMAAKLQV----QESSLRTHLTQLRPGIAHMI-------------------------------------------------------------------  
----------------------------------------------------------------------------------------------------------------------------------------------------------------------MDAVKTEGRKAAGTVDCALRQPLVPPEKNIAAPAGRRR---EVAS-----RFKSGGTPAPQAAASSVRRCTSPSLSRAS---------AAEGTASTNRAQSAERRRSSTPSSSSTPSGGGGGAAS-------------------------------------------------------------------------------------------------------------RPRTPVRVAAATEVHGISRRAA-------------------------------STKPPDGLWAS-ARSVPP---LLQKESMAMATSAKKRHKLADGSSSDQTKVQARNVTE-----------TERKRSPLRG------------------------RNIGNQCENARPSETPNKRVV---------EQHRWP-----AAMMGGRGSAVLTSRSNGVANTPIRSVTP---------------------------------------------------------SNPTRGLSPR-------------------------------------------------------------RICPAEVKANGLNQPLNGLAKRLATHESRREDKTESGSDVSSQ-T---------------------------------------------------------------------------------------------SENSKAAT----------------------------------------------------------------------------------------------------------------------------------------------------------------------------------------------------------------------------------------------------------------------------------------------------------------------------------------------RP-------SRTLSSPVLHRSSSPNKVLSAASPASTAFQSPLRTRPSAPCR--------SRCCSTSQSGVAPLVFNY--IVDARKGKK-----SASQFENIHQ-----------LRLLYNRCLQWQFVNARSEDTLTFQKSSIESILYSVWKSIVQLRDSVTVRRIDVQLLQQELKLYYVLKEQ-------IAYLQHWPKLEGENGSTLIGAIEALQACTLRLPVTSGAQADAVAVKNSISSAVDVMQALSSSILYLLSKVEG-----------RTSLVSELSDMARQEKVALGECRELLATAAKLQV----QESSLRTHLMQLK-EGVLG--------------------------------------------------------------------  
---------------------------------------------------------------------------------------------------------------------------------------------MDACGIRAPGDVLLRKSELSSAAAAAKNYGNGHDDAAVRRKAAAGSPATPRRHPSPNAGRSSAAAAEAAGSQARRSQSTERRPATPSRLSPGGSRAAAPSSRISAPTSPSSAPSSPSSSSSSSSTPVRDAVAAESQSAPRRLAGGRAPPDGLWPSMRSLSSSLQLEAKGKRSNGGSADQAKARDAGDRKRSPSRGRSAAEQQPENPHAKVIDHHRWPAMMGGRVSVSAMSRSVDLTDKISRPALSSIPSRGVSPKKATMASTTNALARSIDLADKIDRLVSLSVSSPRTPTASNGAADESKSMSVSKGTKPAAVAIPSRVSAIITATSGGIRALSKSMDLTEKDIGTLSSAASSPGISPSVSVSSMSNATSQTTAKSTRGLSPRRTSTSIGSGALSRNIDLPENDKRPASSSASLRGNSPRRRLASDSVNAVVKNIDFAEKDSRAAISSTSSRGFSPRRRLASDGLDAISRSTDFSDKDSRPSTSSSSAQRGISPLRRLTISKGTDFTDKSYRPSTSSAASRGVSPRTRLASDSAGNILKSMDLADRDNKPSTSSASLRGMSPRRRLASDGISKNITFTEKDDRTMPSSVASQEISTIRRLPSDGADSISKNIDLPEKVTRPATSSAASRGLSPRRRLASDGVNAISKSIDLADKDTGPARSTAALRGVSPRRQLASDRVDSISKNTDFTEKDKDTRPSTSSGASRGISPRRRLASDGVDDLSKGINFSQKSIRPSTSSMASRGTSPRRRLASDGVNALLKSTDFTDKDHRPSTSSAALRGMSPRNRVTSKSIDAKSLDFSDKDSRPFTPSGASQGTLQEVALASDGINALSEAVDTAVIGSLQSTSSVESGETSDARLNNGSGTVVNRIDFAQEVNIATPDGCNGHISESMDSHDIGTSAPSMSITSQEQSPSRTVSNGPKTLSEDINATKKNNRAMTVKIPSRGASPRRRLASEGFGTIYKSMDFSEKDRTSINMATPSRGMSPRRTARSGIVDMSKSMDFSEKCNGPISSIAPSHVVSARRILGPDGANAMSRSMDLTDKIRQPISSTVRKMSLADSRAKAPDLLSGDIESPGSANGNESQEENAGSSLDAPSNDSEKSAPPKRLARTLSSPSPTKASSISSFTPRRMPSPSRNRPSTPVS----------PCSSTRSDSASSILSY--MGDVTRGKR-----SPSHMEDAHQ-----------LRLLYNRSLQWRFTNAYVDEMQSVQKMSAETMLYSVWDANSSLCDSMVMKRSYVQRLRQEVKLGVVLKEQ-------MDYLTHWAALETEHSTSLSSAIEALRASTLRLPVTGGAKADVFTVKNAVSSAVDIMQAMGSSVCYLLSKLQA-----------THSLVTELSAVAANESSMLNEYRELLGTAAALQV----LESSLRTQLIQETE-------------------------------------------------------------------------  
-------------------------------------------------------------------------------------------------------------------------------------------------------------------------MRSLSVSFQGESFFYQTSRAPRAASPSSPGGRRGPTPERRKS-------------------------------------------------VSSVPEAENTRPQHRWPAAKPKASDPLARSL-------------------------------------------------------------------------------------------------------------DCSLDRKDSILAAVHLLRRSMA--------------------------------------------------------------------------------------------------------------------------------------------------------------------------------------------------------------------------------------------------------------------------------------------------------------------------------------------FDSTTSLSPSDPAAAAAAAHDLSASSDTDSVSSGSNSGAGDPP-R----------------------RGISVPARFWQETNSR-------------L-RRLPEPGL--------------------------------PLPSSSGR----------------------------------------------------------------------------------------------------------------------------------------------------------------------------------------------------------------------------------------------------------------------------------------------------------------------------------------------RS------------FSDSQMSPRLPGRSPSPSRGSRGMASPARGRSGEAS---------PNGHTMQAPANAPSIISF--AAEVRRAKK-----GENRIEEAHR-----------LRLLDNRHLQWRCINARTDAALLVQSFNAEKTLHSAWKEISKLRDNVSSKRSKLQLLKQKLKLFAILRRQ-------IYYLDEWSHIEKHHSSALSAAIEALKASTLRLPVVGGAKADAQGVKEAVNSAVDVMHTMASSMCTLLSKVEG-----------TSSVVSELAKLATQEQMLLDQSRDLLSMVAAIHV----KQCSLQAHMLQRKQKQSQTRV------------------------------------------------------------------  
----------------------------------------------------------------------------------------------------------------------------------------------------------------------------------MAATLAQEPRHHPSRPPLAPAAAHAPNSAAAAC-------------------------------------------------------STPRRGKTSPHASSRHASSSSSSSS-------------------------------------------------------------------------------------------------------------LPSCSAARVAVTPAPHATATAP---------------------------------------VTMRMRSLSV---SFQGESFVYETPRAAAPRRAPAAA------------------------AARPRPTTRR------------------------RGEAENERPSPPPASKAT---------------------------------DALARSLDCSLHRKE--------------------------------------------------------------------------------------------------------------------------------------SILAAVRLLRSSISPGNAAAAAAPDADAATDTDTDAAPPSI-P---------------------------------------------------------------------------------------------TQTRFWQE----------------------------------------------------------------------------------------------------------------------------------------------------------------------------------------------------------------------------------------------------------------------------------------------------------------------------------------------TN---------------------SRLRRLPESGLPHPISTSRKPFLDGPIS--------PTLLETSPA-NAPSIISF--ATAVRRANK-----GEDKIEEAHR-----------LRLLDNRQLQWRCLNAHADAAAVARSCAAEKALHSAWKDISTLRDNVSFKRSKLQLQKQKLKLFGILKGQ-------ISYLEEWSDVENNHSSSMSEAIKALEASTIRLPIVCGAKADAQGVKKVVSSALTKMDTMASSMWSLLSKVEG-----------MSSMVFELAKVVSQEQMLLDQSRDLFSAVAVMHV----KLCSLQACILQRN--------------------------------------------------------------------------  
------------------------------------------------------------------------------------------------------------MCTIRTDGAEWQSSILFVWPGPVKRERETTGKAERGELSVGWLVLELELEDVVHHEMMFERSSSGDVKRRMVAAVASSSPSAPVDNVLEAQIERKKEGKNVQNYGGLPPAEKVKVVRKARTMEITSRYKTATTALHTPVTPASRRPSPIRQ---SSPSRQPSPVRNRSCSSNSSIVGASECLL-------------------------------------------------------------------------------------------------------------RHPSPNLGRSNNVPDVTKRALSAERTRRPWPAAISSESKSTSMSSTAVGLASSIKSRPHFRGPELWPSMSASKQGADSNGDSASDRSDCLSEGGSEKEAISKTLNNAPRPAADGNGIGKISGSTSRPGSPMR------------------------RQSIDQSDTARSTENSHSKP----------DHQRWP------GMSTVKVSSGAMNRSVDVGVDRERPLAR--STTTTTPTRSGTPSSRIKAAISRSYIRSGCSDGSVAPSSQVAPRRNPTPPRGRTGTTESVAQGTL-------------------------------------------------------------ANSNGNDDSAHPSGNGTGGGDHASHARRLSQESVATADSLSRPVDNVSDTESESVISAGSAPGSRVVRGTTVPARVWQDMNNRLRRFSEGDQNRTSGASDLPAVAIAPVKTVRRSKVVPHQSAVSTLMNQSMNQSMN-GSTSAWAF----------------------------------------------------------------------------------------------------------------------------------------------------------------------------------------------------------------------------------------------------------------------------------------------------------------------------------------------SP------GRLPVNSSSSTPHPPSSPSHSKGTSPLRGLPSPQRSRPVPGAA---------VALAGSARNLGSTTVNF--GIDGRNKGK----KVLTQQEEAQL-----------LRILHNRWLQWRFVNARAEAVMSAQKAAAERQLYNVWLRTSELRTSVAMQRIKLQQARQAHKLRSILSVH-------AIHLENWETLEEEHSNALTGCMEALESAILRVPVTGGARADVHAVKEALNAAVDVLNAVEGSVHVLLPKTQA-----------MEALLLQLAETAAQERALLEECGDLLSIAASLEV----EERSLRTHLIQLENERQSLFKA-----------------------------------------------------------------  
MCTILSARVAVWTHPRHDIVDVSDQQQQPGCALQLDEVGGHEPGNASITRSPLKSSGVMPVELQHPPLGALRLHTDCGGDVRCEGEVIDVEALEGEVEGSGRPRRRRGGNVEGDDERRSGAGGVLEWHQERVGGKARLRLRLRLGGGNWRCCIDVRQIIERLCEVVMTLRIRDCGLGESSSAVVSSSPPSSVANVQEAKTGKKKEPVNVQNYGGLPPTEKVKLVHKPRTMEITSRYKTATIASPTLTTPASRRPSPLRQSSPSSRQSSPARNRSSSSGSSNGTKGGLESHL-------------------------------------------------------------------------------------------------------------RHPSPNTGRSTNAPELTKRAFSTERTRRPWPAVIPSEIKPVSTVSSSVGLASLTKARPQSRGPELWPSMSVVKQSVDTNSESASDRSDCQSEGGKEKDLTNKVSKHTPKSTGSGTVNGNISGSPSRKGSPMR------------------------RQSIVQSENARPTENSHSKP----------DQQRWP------GMSTGKILNTSMNRNSDLGVEKERPLARSVAMTTQSRPGTPSSRIKATLSRSYSRSGDSEGPVAPSGQVALRRNPTPTRGGAGTTGSAAREPVIN-------------------------------------------------------------NNCSGNADAAHVSGNGTGATDHATHARRLSQESVATVDSLSTP-A-ETMSDVESVSSAGSVPGSRNVRGTTVPARVWQDTNTRLRRLSEGERNRTSDAADLPAVAIAPVKTFRRMKVLPHQSAVSLLMNQSMNQSMNGSTTSAWAL----------------------------------------------------------------------------------------------------------------------------------------------------------------------------------------------------------------------------------------------------------------------------------------------------------------------------------------------SP-------GRMSGSVPSTPHPPSSPSHSKGTSPLRRLPSPQRSRPVNVAA---------AALAGTARSLGSTTLNF--GIDGRSRGK----KALTQQEEVQL-----------LRILHNRWLQWRFVNSRAEAVMSSQKAAAERQLFNVWVKTSELRTSVAMQRIKLQQARQAHKLRSILSTH-------ATYLENWKTLEEEHSNALTGCMEALESAILRVPMTGGARADVQAIKEALNSAVDVLNAIEGSVHFLLPKTQN-----------MDALLSQLAETAAQERALLEECGDLLSVAASLEV----EERSLRTHLIQLENERLRAS-------------------------------------------------------------------  
-----------------------------------------------------------------------------------------------------------------------------------------------------------------------------------MQTEKKREAGNVHNYGSHPPVEKVKVVRKSRTMEITSRYKSSIASPSPLVTPAGNRRNPSPLSL---------NPSPARQPPSAPSSRQSSPVRKDSIVPSSCAKGPSESVV-------------------------------------------------------------------------------------------------------------RHPSPNLGRSSNASEVLKRSYSTERRRPWPAVSPPSEKKTTSLSSTTEGLTSSVKAPPLSKGPELWPSMSA---AKQSADSNEDSVSSCQAKRGREKETTGKHPNHTLKPAGNGNGTAHVPGTPCRKGSPMR------------------------RQCADQAEIARPTENSHSKL----------DHQRWP------GMRRGKNFGGNMTRSTDLTVERKLSLVR-----SPTMTGQSRPVTPLSRTKSISSRSFNRSANDGPIATPGQVALRRNPSPSRSRTGTTESAAQV-------------------------------------------------------------SVASSDSDDVHPCSNTTFQGDHATHARRLSQESVATVDSLSVP-L-ENMSDTESVSSGSSAPEVRVGRGATIPARVWQDMSNRLRRFSEGGRNRSSG-VDLPAVAIVPVKTIRRTKVLPHQSAASMLMNQSMN-----GSTSSWAL----------------------------------------------------------------------------------------------------------------------------------------------------------------------------------------------------------------------------------------------------------------------------------------------------------------------------------------------SP------GRVSGNSAPSTPHPPSSPSHSKGTSPSRGLPSPQRSRPVNGSA---------AALAGTARNFAGTTLNF--GIDGRSRGK----KALTQQEEAQL-----------LRILHNRWLQWRFVNARAEAVTRAQKATAERQLYYVWVKTSELRTSVAMQRIKLQQARQAHKLRSILSTH-------ATHLEDWETVEEEHSNALTGCMEALESAILRVPVTGGARADVHAVKEALYLAVDVLTAIEGSVYVLLPKTES-----------MKALLSQLAETAAQERALLEECGDLLSVAASLEV----EERSLRTHLIQLERERQSSYKADPPNGTSSMSASSHLFTP-----------------------------------------------  
---------------------------------------------------------------------------------------------------------------MRCAGTGPEEECCWSGLKSGQDELRSFCGRSLSLEAVLVGRAGDATADVKQPGKFTRKGREQADIRSWSPRWRLPPPQLLQNYGGLPPAEKVKVVRKTRTMEITSRYKSAITAPSPPVAPAGSRRNPSPLSRNPSPLSRNPSPARQPSPASSSRQPSPVRNRST--SSSSSIKGASESLV-------------------------------------------------------------------------------------------------------------RHPSPTLGRSINAPEVLKRSYSTERRRPWPAVTPSSESKSTPTSSTTDGLASSTKVRPQTRGPELWPSMSA---AKQSADSNDDNVSDCLSEGGTEKEAPSKIPKPALNSAGNGNGNGHVLGSPSRKGSPMR------------------------RQSIDQAETTRPTENSHSKP----------DQQRWP------GMSTGKVSGGTMTRSMDLNVDRERPLAR-----SSTMTAQSRPGTPSSRIKATPSRSFNRSVNEAPVATPGQTLSRRNPTPTRGRTGTTENAARE-------------------------------------------------------------PGPSTNGNGAHPSSNATGPGEHATHARRLSQESVATVDSLSTP-Q-ENMSDTESVSSGGSIPGSRAGRGTTVPARVWQDMNGRIRRFSEGDRSRSSD-VDLPAVAIAPVKTVRRTKVLPYQPAASMLMNQSMNG----STTSAWAL----------------------------------------------------------------------------------------------------------------------------------------------------------------------------------------------------------------------------------------------------------------------------------------------------------------------------------------------SP------GRASGNSAPSTPHPPSSPSHSKGTSPLRGLPSPQRSRPVHGSA---------AALAGTARNFAGTTLSF--GIDGRSRGK----KTLTQQEEAQL-----------LRILHNRWLQWRFVNARAEAVMSAQKAAAERQLYNVWLKTSELRTSVAMQRIKLQQARQAHKLRSILSTH-------ATHLEDWETLEEEHSNALTGCMEALESAILRVPVTGGARADVQAVKEALNSAVDVLNAVEASVHFLLPKTDS-----------MEALLSQLAETAAQERALLEECGDLLSVAAALEV----EERSLRTHLIQLENERQRLFKAESLNGTLNASSSYLSTP------------------------------------------------
