# Supplementary material for: Coiled-Coil Proteins Facilitated the Functional Expansion of the Centrosome
Source: PLoS Comput Biol. 2014 Jun 5;10(6):e1003657. doi: 10.1371/journal.pcbi.1003657 (PMC4046923; doi:10.1371/journal.pcbi.1003657)
Supplement: Dataset S1 — Multiple-sequence alignments. This file contains alignments for the protein families spd-5, AKAP9/PCNT, PCM1, HAUS7 and HAUS8 in FASTA format and as HTML pages with highlighted coiled-coil domains. (ZIP) [file pcbi.1003657.s021.zip › alignments/PCM1.html]

Multiple Alignment


1  
|

5  
|

10  
|

15  
|

20  
|

25  
|

30  
|

35  
|

40  
|

45  
|

50  
|

55  
|

60  
|

65  
|

70  
|

75  
|

80  
|

85  
|

90  
|

95  
|

100  
|

105  
|

110  
|

115  
|

120  
|

125  
|

130  
|

135  
|

140  
|

145  
|

150  
|

155  
|

160  
|

165  
|

170  
|

175  
|

180  
|

185  
|

190  
|

195  
|

200  
|

205  
|

210  
|

215  
|

220  
|

225  
|

230  
|

235  
|

240  
|

245  
|

250  
|

255  
|

260  
|

265  
|

270  
|

275  
|

280  
|

285  
|

290  
|

295  
|

300  
|

305  
|

310  
|

315  
|

320  
|

325  
|

330  
|

335  
|

340  
|

345  
|

350  
|

355  
|

360  
|

365  
|

370  
|

375  
|

380  
|

385  
|

390  
|

395  
|

400  
|

405  
|

410  
|

415  
|

420  
|

425  
|

430  
|

435  
|

440  
|

445  
|

450  
|

455  
|

460  
|

465  
|

470  
|

475  
|

480  
|

485  
|

490  
|

495  
|

500  
|

505  
|

510  
|

515  
|

520  
|

525  
|

530  
|

535  
|

540  
|

545  
|

550  
|

555  
|

560  
|

565  
|

570  
|

575  
|

580  
|

585  
|

590  
|

595  
|

600  
|

605  
|

610  
|

615  
|

620  
|

625  
|

630  
|

635  
|

640  
|

645  
|

650  
|

655  
|

660  
|

665  
|

670  
|

675  
|

680  
|

685  
|

690  
|

695  
|

700  
|

705  
|

710  
|

715  
|

720  
|

725  
|

730  
|

735  
|

740  
|

745  
|

750  
|

755  
|

760  
|

765  
|

770  
|

775  
|

780  
|

785  
|

790  
|

795  
|

800  
|

805  
|

810  
|

815  
|

820  
|

825  
|

830  
|

835  
|

840  
|

845  
|

850  
|

855  
|

860  
|

865  
|

870  
|

875  
|

880  
|

885  
|

890  
|

895  
|

900  
|

905  
|

910  
|

915  
|

920  
|

925  
|

930  
|

935  
|

940  
|

945  
|

950  
|

955  
|

960  
|

965  
|

970  
|

975  
|

980  
|

985  
|

990  
|

995  
|

1000  
|

1005  
|

1010  
|

1015  
|

1020  
|

1025  
|

1030  
|

1035  
|

1040  
|

1045  
|

1050  
|

1055  
|

1060  
|

1065  
|

1070  
|

1075  
|

1080  
|

1085  
|

1090  
|

1095  
|

1100  
|

1105  
|

1110  
|

1115  
|

1120  
|

1125  
|

1130  
|

1135  
|

1140  
|

1145  
|

1150  
|

1155  
|

1160  
|

1165  
|

1170  
|

1175  
|

1180  
|

1185  
|

1190  
|

1195  
|

1200  
|

1205  
|

1210  
|

1215  
|

1220  
|

1225  
|

1230  
|

1235  
|

1240  
|

1245  
|

1250  
|

1255  
|

1260  
|

1265  
|

1270  
|

1275  
|

1280  
|

1285  
|

1290  
|

1295  
|

1300  
|

1305  
|

1310  
|

1315  
|

1320  
|

1325  
|

1330  
|

1335  
|

1340  
|

1345  
|

1350  
|

1355  
|

1360  
|

1365  
|

1370  
|

1375  
|

1380  
|

1385  
|

1390  
|

1395  
|

1400  
|

1405  
|

1410  
|

1415  
|

1420  
|

1425  
|

1430  
|

1435  
|

1440  
|

1445  
|

1450  
|

1455  
|

1460  
|

1465  
|

1470  
|

1475  
|

1480  
|

1485  
|

1490  
|

1495  
|

1500  
|

1505  
|

1510  
|

1515  
|

1520  
|

1525  
|

1530  
|

1535  
|

1540  
|

1545  
|

1550  
|

1555  
|

1560  
|

1565  
|

1570  
|

1575  
|

1580  
|

1585  
|

1590  
|

1595  
|

1600  
|

1605  
|

1610  
|

1615  
|

1620  
|

1625  
|

1630  
|

1635  
|

1640  
|

1645  
|

1650  
|

1655  
|

1660  
|

1665  
|

1670  
|

1675  
|

1680  
|

1685  
|

1690  
|

1695  
|

1700  
|

1705  
|

1710  
|

1715  
|

1720  
|

1725  
|

1730  
|

1735  
|

1740  
|

1745  
|

1750  
|

1755  
|

1760  
|

1765  
|

1770  
|

1775  
|

1780  
|

1785  
|

1790  
|

1795  
|

1800  
|

1805  
|

1810  
|

1815  
|

1820  
|

1825  
|

1830  
|

1835  
|

1840  
|

1845  
|

1850  
|

1855  
|

1860  
|

1865  
|

1870  
|

1875  
|

1880  
|

1885  
|

1890  
|

1895  
|

1900  
|

1905  
|

1910  
|

1915  
|

1920  
|

1925  
|

1930  
|

1935  
|

1940  
|

1945  
|

1950  
|

1955  
|

1960  
|

1965  
|

1970  
|

1975  
|

1980  
|

1985  
|

1990  
|

1995  
|

2000  
|

2005  
|

2010  
|

2015  
|

2020  
|

2025  
|

2030  
|

2035  
|

2040  
|

2045  
|

2050  
|

2055  
|

2060  
|

2065  
|

2070  
|

2075  
|

2080  
|

2085  
|

2090  
|

2095  
|

2100  
|

2105  
|

2110  
|

2115  
|

2120  
|

2125  
|

2130  
|

2135  
|

2140  
|

2145  
|

2150  
|

2155  
|

2160  
|

2165  
|

2170  
|

2175  
|

2180  
|

2185  
|

2190  
|

2195  
|

2200  
|

2205  
|

2210  
|

2215  
|

2220  
|

2225  
|

2230  
|

2235  
|

2240  
|

2245  
|

2250  
|

2255  
|

2260  
|

2265  
|

2270  
|

2275  
|

2280  
|

2285  
|

2290  
|

2295  
|

2300  
|

2305  
|

2310  
|

2315  
|

2320  
|

2325  
|

2330  
|

2335  
|

2340  
|

2345  
|

2350  
|

2355  
|

2360  
|

2365  
|

2370  
|

2375  
|

2380  
|

2385  
|

2390  
|

2395  
|

2400  
|

2405  
|

2410  
|

2415  
|

2420  
|

2425  
|

2430  
|

2435  
|

2440  
|

2445  
|

2450  
|

2455  
|

2460  
|

2465  
|

2470  
|

2475  
|

2480  
|

2485  
|

2490  
|

2495  
|

2500  
|

2505  
|

2510  
|

2515  
|

2520  
|

2525  
|

2530  
|

2535  
|

2540  
|

2545  
|

2550  
|

2555  
|

2560  
|

2565  
|

2570  
|

2575  
|

2580  
|

2585  
|

2590  
|

2595  
|

2600  
|

2605  
|

2610  
|

2615  
|

2620  
|

2625  
|

2630  
|

2635  
|

2640  
|

2645  
|

2650  
|

2655  
|

2660  
|

2665  
|

2670  
|

2675  
|

2680  
|

2685  
|

2690  
|

2695  
|

2700  
|

2705  
|

9606.ENSP00000327077  
10116.ENSRNOP00000039753  
10090.ENSMUSP00000039056  
9615.ENSCAFP00000010585  
13616.ENSMODP00000029799  
13616.ENSMODP00000023765  
9258.ENSOANP00000028445  
59729.ENSTGUP00000007334  
9103.XP\_003205832  
9031.ENSGALP00000036784  
28377.ENSACAP00000006610  
28377.ENSACAP00000006522  
8364.ENSXETP00000027245  
69293.ENSGACP00000022389  
8090.ENSORLP00000010144  
99883.ENSTNIP00000009932  
31033.ENSTRUP00000040906  
7955.ENSDARP00000084087  
7955.ENSDARP00000084104  
7739.JGI117108  
7739.JGI117115  
7668.XP\_786223  
10224.XP\_002737891  
7159.AAEL009976-PA  
7159.AAEL010873-PA  
7176.CPIJ000824-PA  
7070.D2A1X2  
121224.XP\_002423829  
225164.jgi|Lotgi1|153121|fgene  
283909.jgi|Capca1|228845|estEx  
6085.XP\_002169154  
45351.JGI228836  
10228.JGI62202  
400682.Aqu1.200968  
400682.Aqu1.222753  
81824.JGI33155  
109871.jgi|Batde5|86359|GP3.01  
645134.SPPG\_06502T0

Homo sapiens  
Rattus norvegicus  
Mus musculus  
Canis lupus familiaris  
Monodelphis domestica  
Monodelphis domestica  
Ornithorhynchus anatinus  
Taeniopygia guttata  
Meleagris gallopavo  
Gallus gallus  
Anolis carolinensis  
Anolis carolinensis  
Xenopus (Silurana) tropicalis  
Gasterosteus aculeatus  
Oryzias latipes  
Tetraodon nigroviridis  
Takifugu rubripes  
Danio rerio  
Danio rerio  
Branchiostoma floridae  
Branchiostoma floridae  
Strongylocentrotus purpuratus  
Saccoglossus kowalevskii  
Aedes aegypti  
Aedes aegypti  
Culex quinquefasciatus  
Tribolium castaneum  
Pediculus humanus corporis  
Lottia gigantea  
Capitella teleta  
Hydra magnipapillata  
Nematostella vectensis  
Trichoplax adhaerens  
Amphimedon queenslandica  
Amphimedon queenslandica  
Monosiga brevicollis  
Batrachochytrium dendrobatidis  
Spizellomyces punctatus DAOM BR117

MATGGG---------------------PFEDGMNDQDLPNW--SNENVDDRLNNMDWGAQQKKANRSSEKNKK--------KFGVESDKRVTNDISPESSPGVGRRRT---------------KTPHTFPHSRYMSQMSVPEQAELEKLKQRINFSDLDQRSIGSDSQGRATAANNKRQLS-ENRKPFNFLPMQINTNKSKDASTNPPNRETIGSAQCKELFASALSNDLLQN-----------------CQV-SEEDGRGEPAMESSQIVSRLVQIRDYITKASSMREDLVEKNERS----------------------------------ANVERLTHLIDHLKEQEKSYMKFLKKILAR-------------------------------------------------------------------------------------------------------------------------------------------------------------------------------DPQQEPMEEIENLKKQHDLLKRMLQQQEQLRALQGRQAALLALQ-----------HKAEQAIAVMDDSVVAETAGSLSGVSITS-----------------ELNEELNDLIQRFHNQLRDSQPPAVPDNRRQAESLSLTREVSQSRKP-------------------SASERLPDEKVELFSKMRVLQEKKQKMDKLLGELHTLR--DQHLNNS-----S----SSPQRSVD-QRS-TSAPSASVGLAPVVNGESNSLTSSVPYPTASLVSQNESENEGHLNPSEKLQKLNEVRKRLNELRELVHYYEQTSDMMTDAVNENRKDE-E--------TEESE-----YDSEHENSEPVTNIRN---PQVASTWNEVNSHSN----AQCVSNNRDGRTVNSNCEINNRSAANIRALNMPPSLDCRYNR---EGEQEIHVAQGEDDEEEEEEAEEEGV---------SGASLSSHRSSLVDEHPEDAEFEQKINRLMAAKQKLRQLQDLVAMVQDDDAA-QGVISASASNLDDF-------------YPAEEDT-KQNSNNTR--------GNANKTQKDTGVNEKA---------------REKFYEAKLQQQQRELKQLQEERKKLIDIQEKIQALQTA---CPDLQLSAASVGNCP---TKKYMPAVTSTPTVNQH-ETSTSK---SVFEPED-----SSIVDNELWSEMRRHEMLREELRQRRKQLEALMAEHQRRQGLAETASPVAVSLRSDGSENLCT--PQQSRT-E-KTMATWGGSTQCALDEEGDE----------DGYLSEGIVR----TDEEEEEE---------QDASSNDNFSVCPSNSVNHNSYNGKETKNRWKN-----------NCPFSADENYRPLAKTRQQ-NISMQRQENLRWVSELSYVEEKE---QWQEQINQ--LKKQLDFSVSICQTLMQDQQTLSCLLQ------TLLTGPYSVMPS-----------------------NVASPQVHFIMHQLNQCYTQLTWQQNNVQRLKQMLNELMRQQNQHP--EKPGGKERGSSASHP-----PSPSLFCPFSFPTQPVNLFNIPGFTNFSSFAPGMNFSPLFPSNFGDFSQNISTPSEQ-Q---QPLAQNSSGKTEYMAFPKPFESSSSIGAEKPR-NKKLPEEEVESSRTPWLYE-QEGEVEKPFIKTG-FSVSVEKSTSSNRKNQLD---------TNGRRRQFDEESLESFSSMPDPVDPTTVTKTFKT-RKASAQASLASKDKTPK-SKSKKRNSTQLKSR-VKNIRYESASMSSTCEPCKSR---NRHSAQTEEPVQAKVFSRKNHEQLEKIIK-CNRSTEISS----------------------------ETGSDFSMFEALRDTIYSEVATLISQNESRPHFLIELFHELQLLNTDYLRQRALYALQD---IVSRHISESH-----EK-GENV-KSVNSGTW-IA-SNSELTPSESLATTDDETF-----EKNFERET---------HKISEQNDADNASVLSVSSNF---------EPFATDDL--GNTVIHLDQA-----LARMREYERMKTEAESNSNMR--CTCRIIEDGDGAGAGTTVNNLEETPVIENRSSQQPVSEVSTI--------PCPRIDTQQLDR-----QIKAIMKEVIPFLKEHMDEVCSSQLLTS---VRRMVLTLTQQ--------NDESK---EFVKFFHKQLGSIL-QDSLAKFAGRKLKDCGEDLLVEISEV-LFN-ELAFFKLMQDLDNN---SITVKQRCKRKIEATGVIQSCAKEAKRILE-DHGSPAGEIDDED---KDKDETETVKQTQ----TSEVYDG---PKNVRSDISDQEEDEES-EGCPVSINLSKAE-TQALTNYGSGEDENEDEEMEEFEEGPVDVQTSLQ-ANTEATEENEHDEQVLQRDFKKTAESKNVPLEREAT-SKND--------QNNCPVKPCYLNILEDEQPLNSAAHKESPPTV-DSTQQPNPLPLRLPEMEPLVPRVKEVKSAQETPESSLAGSPDTESPVLVNDYEAESGNISQKSDEEDFVKVEDLPLK--LTI-YSEADLRKKMVEEEQKNHLSGEI-CEMQT-----EELAGNSETLKEPETVG-AQSI--------------------  
MATGGG---------------------PFEEVMHDQDLPNW--SNDSVDDRLNNMEWGGQQKKANRSSEKNKK--------KFGVASDKRVTNDISPESSPGVGRRRT---------------KVPHTFPHSRYMTQMSVPEQAELEKLKQRINFSDLDQRSIGSDSQGRATAANNKRQLG-ENRKPFNFLPMQINTNKSKDATASLPKRETTTSAQCKELFASALSNDLLQN-----------------CQV-SEEDGRGEPAMESSQIVSRLVQIRDYITKASSMREDLVEKNERS----------------------------------ANVERLTHLIEHLKEQEKSYMKFLQKILAREN-----------------------------------------EEED---VRTIDSAVGSGSVAESTSLNVDVQSEASDTTA--------------------------------------------------------------------------------------------RDPQQEPMEETENLKKQHDLLKRMLQQQEQLRALQGRQAALLALQ-----------HKAEQAIAVMDDSVVTETTGSLSGVSITS-----------------ELNEELNDLIQRFHNQLRDSQPPAVPDNRRQAESLSLTREISQSRNP-------------------SVSEHLPDEKVQLFSKMRVLQEKKQKMDKLLGELHTLR--DQHLNNSSFVP-S----TSLPRSGD-KRSSTVALSAPVGFAPAVNGESNSLISSVPCPAASLVSQNESENEGHLNPAEKLQKLNEVQKRLNELRELVHYYEQTSDMMTDAVNENTKDE-E--------TEESE-----YDSEHEHSEPVTNIRN---PQVASTWNEVNTNSN----TQRGSNNREGRSINSSCEINNRSAANIRPLTTPL-LDCRYNR---EGEQRLRVARGED---EEEEVEEEGV---------SGASLSSRRSSLVDEAPEDEEFEQKISRLMAAKEKLKQLQDLVAMVQDDDAT--QVLVPAASNLDDF-------------YVAEEDT-KQNSNNAR--------ENSNK--VDTGVNEKT---------------REKFYEAKLQQQQRELKQLQEERKKLIEIQEKIQAVQKA---CPDLQLSATSISSGP---TKKYLPAITSTPTVNEN-ETSTSK---CDIEPED-----SSVVDNELWSDMRRHEMLREELRQRRKQLEALMAEHQRRQGLAETASPVAVSLRSDGSENLGT--PQQSRT-E-KTMATWGGSTQCALDEDGDE----------DGYLSEGIVR----TDEEEEEE---------QDASSNDNFPMYHP-SMNQNSYNVKETKNRWKN-----------HRPVSADGNYRPLAKTRQQ-NISMQRQENLRWVSELSYIEEKE---QWQEQINQ--LKKQLDFSVNICQTLMQDQQTLSCLLQ------TLLTGPYSVLPS-----------------------NVASPQVHLIMHQLNQCYTQLTWQQNNVQRLKQMLTELMRQQNQHP--EKPRSKERGSSASHP-----SSPSLFCPFSFPTQPVNLLNLPGFTNFPSFAPGMNFSPLFPSNFGDFSQNVSTPTEQ-Q---QPLAQNPSGKTEYMAFPKPFESSSSLGAEKQR-NQKQPEEETENTKTPWLYD-QEGGVEKPFFKTG-FAVSVEKATNSNRKRQPD---------TSRRGRQFDEESLESFSSMPDPIDPTTVTKTFKT-RKASAQASLASKDKTPK-SKSKKRNSTQLKSR-VKTIGYESASVSSTCEPCKSR---NRHSAQTEEPVQAKLFSRKNHEQLEKIIK-YSRSAEISS----------------------------ETGSDFSMFEALRDTIYSEVATLISQNESRPHFLIELFHELQLLNTDYLRQRALYALQD---IVSRHISESD-----EKEGENI-KSVNSGTW-VA-SNSELTPSESLVTTDDETF-----EKNFERET---------HKITEQNDADNVSVMSVSSNF---------EPFATDDL--GNTVIHLDQA-----LARMREYERMKTETESNSNMR--CTCRVIEDEDGAAATTTVSNAEETPIIENHSSPQPTSDVSAV--------PCPRIDTQQLDR-----QIKAIMKEVIPFLKEHMDEVCSSQLLTS---VRRMVLTLTQQ--------NDESK---EFVKFFHKQLGSIL-QDSLAKFAGRKLKDCGEDLLVEISEV-LFN-ELAFFKLMQDLDNN---SIAVKQRCKRKIEAAGVIQSYAKEAKRILEGDRGSPAGEIDDED---KDKDETETVKQTQ----TSEVYDAKG-PKNVRSDVSDQEEDEES-ERCPVSINLSKAE-SQALTNYGSGEDENEDEEMEDFEESPVDVQTSLQ-ANTETTEENEHDNQVLQHDLEKTQESTNVPSDQEGT-SKND--------QDSSPVKPCYLNILENEQHLNSATHKDSLTTTTDSSKQPDPMPLPLTASETLVPRVKEVKSAQETPESSLAGSPDTESPVLVNDYEAESGNISQKSDEEDFVKVEDLPLK--LTV-CSEAELRKKMVEEEQKNHLAGEI-CEMQT-----EELAGNPQILKEPETVG-AQSV--------------------  
MATGGG---------------------PFEEVMHDQDLPNW--SNDSVDDRLNNMEWGGQQKKANRSSEKNKK--------KFGVASDKRVTNAISPESSPGVGRRRT---------------KIPHTFPHSRYMTQMSVPEQAELEKLKQRINFSDLDQRSIGSDSQGRATAANNKRQLS-ENRKPFNFLPMQINTNKSKDATASLPKREMTTSAQCKELFASALSNDLLQN-----------------CQV-SEEDGRGEPAMESSQIVSRLVQIRDYITKASSMREDLVEKNERS----------------------------------ANVERLTHLIEHLKEQEKSYMKFLQKILAREN-----------------------------------------EEED---VRTVDSAVGSGSVAESTSLNADVQSEASDTTA--------------------------------------------------------------------------------------------RDPQQEPMEETENLKKQHDLLKRMLQQQEQLRALQGRQAALLALQ-----------HKAEQAIAVMDDSVVTETTGSLSGVSITS-----------------ELNEELNDLIQRFHNQLRDSQPPAVPDNRRQAESLSLTREISQSRNP-------------------SVSEHLPDEKVQLFSKMRVLQEKKQKMDKLLGELHNLR--DQHLNNSSFVP-S----TSLQRSGD-KRSSTVALSAPVGFASAVNGEANSLISSVPCPATSLVSQNESENEGHLNPAEKLQKLNEVQKRLNELRELVHYYEQTSDMMTDAVNENTKDE-E--------TEESE-----YDSEHENSEPVTNIRN---PQVASTWNEVNTNSN----TQCGSNNRDGRPVNSNCEINNRSAANIRALNMPP-LDCRYNR---EGEQRLHVAHGED---EEEEVEEEGV---------SGASLSSRRSSLVDEAPEDEEFEQKISRLMAAKEKLKQLQDLVAMVQDDDAT--QVVVPAASNLDDF-------------YAAEEDI-KQNSNNAR--------ENSNK--IDTGVNEKT---------------REKFYEAKLQQQQRELKQLQEERKKLIEIQEKIQAVQKA---CPDLQLSATSISSGP---TKKYLPAITSTPTVNEN-DSSTSK---CVIDPED-----SSVVDNELWSDMRRHEMLREELRQRRKQLEALMAEHQRRQGLAETSSPVAISLRSDGSENLCT--PQQSRT-E-KTMATWGGSTQCALDEEGDE----------DGYLSEGIVR----TDEEEEEE---------QDASSNDNFPIYPP-SMNQNSYNVKETKTRWKS-----------NRPVSADGNYRPLAKTRQQ-NISMQRQENLRWVSELSYIEEKE---QWQEQINQ--LKKQLDFSVNICQTLMQDQQTLSCLLQ------TLLTGPYSVLPS-----------------------NVASPQVHLIMHQLNQCYTQLTWQQNNVQRLKQMLTELMRQQNQHP--EKPRSKERGSSASHP-----SSPNLFCPFSFPTQPVNLFNLPGFTNFPSFAPGMNFSPLFPSNFGDFSQNVSTPTEQ-Q---QPLAQNPSGKTEYMAFPKPFESSSSLGAEKQR-NQKQPEEEAENTKTPWLYD-QEGGVEKPFFKTG-FTESVEKATNSNRKNQPD---------TSRRRRQFDEESLESFSSMPDPIDPTTVTKTFKT-RKASAQASLASKDKTPK-SKSKKRNSTQLKSR-VKNIGYESASVSSTCEPCKNR---NRHSAQTEEPVQAKLFSRKNHEQLEKIIK-YSRSAEISS----------------------------ETGSDFSMFEALRDTIYSEVATLISQNESRPHFLIELFHELQLLNTDYLRQRALYALQD---IVSRHISESD-----EREGENV-KPVNSGTW-VA-SNSELTPSESLVTTDDETF-----EKNFERET---------HKVSEQNDADNVSVMSVSSNF---------EPFATDDL--GNTVIHLDQA-----LARMREYERMKTETESHSNMR--CTCRVIEDEDGAAAAATVSNSEETPIIENHNSPQPISDVSAV--------PCPRIDTQQLDR-----QIKAIMKEVIPFLKEHMDEVCSSQLLTS---VRRMVLTLTQQ--------NDESK---EFVKFFHKQLGSIL-QDSLAKFAGRKLKDCGEDLLVEISEV-LFN-ELAFFKLMQDLDNN---SIAVKQRCKRKIEAAGVRQSYAKEAKRILEGDHGSPAGEIDDED---KDKDETETVKQTQ----TSEVYDAKG-PKNVRSDVSDQEEDEES-ERCPVSINLSKAE-SQALTNYGSGEDENEDEEMEDFEESPVDIQTSLQ-ANTETTEENEHDSQILQHDLEKTPESTNVPSDQEPT-SKND--------QDSSPVKPCYLNILENEQQLNSATHKDSLTTT-DSSKQPEPLPLPLAASETLVPRVKEVKSAQETPESSLAGSPDTESPVLVNDYEAESGNISQKSDEEDFVKVEDLPLK--LTV-YSEEELRKKMIEEEQKNHLSGEI-CEMQT-----EELAGNSQILKEPETVG-AQSI--------------------  
MATGGG---------------------PFEEGINDQDLPNW--SNEGVDDRLNNMDWGGQQKKANKSSEKNKK--------KFGVESDKRVTNDISPESSPGVGRRRT---------------KTPHSFPHSRYVTQMSVPEQAELEKLKQRINFSDLDQRSIGSDSQGRATAANNKRQLS-ENRKPFNFLPMQINTNKSKDAAISPPKREMIGSTQCKELFASALSNDLLQN-----------------CQV-SEEDGRGEPAMESSQIVSRLVQIRDYITKASSMREDLVEKNERS----------------------------------ANVERLTHLIDHLKEQEKSYMKFLQKILAREN-----------------------------------------EEED---VRTIDSAVGSGSVAESTSLNIDVQSEASDTTA--------------------------------------------------------------------------------------------RDPQQEPMEEIENLKKQHDLLKRMLQQQEQLRALQGRQAALLALQ-----------HKAEQAIAVMDDSVVTETTGSVSGVSITS-----------------ELNEELNDLIQRFHNQLRDSQPPTVPDNRRQAESLSLTREVSQSRNP-------------------SVSEHLPDEKVQLFSKMRVLQEKKQKMDKLLGELHTLR--DQHLNNS-----S----SSPQRSVD-QRSTTSAPSAPIGLAPVVNGESNSFTSSVPYPVASLVSQNESENEGHLNPTEKLQKLNEVRKRLNELRELVHYYEQTSDMMTDAVNENTKDE-E--------TEESE-----YDSEHENPEPVTNIRN---PQVAATWNEVNSNSN----AQCVSNNREGRSVNSNCEINNRSAANIRTLNMPPSLDCHYNR---EGEQGIHGAQGEDDEEEEE-AEDEGV---------SGASLTSHRSSLVDEAAEDAEFEQKINRLMAAKQKLRQLQDLVAMVQDDDAADHGVISANTSNLDDF-------------YPAEEDN-KQSANNTR--------GNANKTQKDAGINEKA---------------REKFYEAKLQQQQRELRQLQEERKKLIEIQEKIQALQKA---CPDLQLSATSAGNCP---TKKYIPAVTSTPVVNGN-ETSTSK---SAFEPAD-----PSGVDNELWSEMRRHEMLREELRQRRKQLEALMAEHQRRQGLAETTSPLAVSLRSDGSENLCT--PQQSRT-E-KTMATWGGSTQCALDEEGDE----------DGYLSEGVVR----TDEEEEEEE--------QDASSNDNFSMYPPNSANHNSYNIKETKNRWKN-----------SRPFTADGNYRPLAKTRQQ-NISMQRQENLRWMSELSYVEEKE---QWQEQINQ--LKKQLDFSVNICQTLMQDQQTLSCLLQ------TLLTGPYSVMPS-----------------------NVASPQVHLIMHQLNQCYTQLTWQQNNVQRLKQMLNELMRQQNQHP--EKPGSQERGSSAPQP-----SSPSLFCPFSFPSQPVNLFNLPGFTNFSSFAPGMNFSPLFPSNFGEFSQNISTPTEQ-Q---QPLAQNSSGKTEYMAFPKPFESSSSIGAEKQR-NQKQPGEEVENSRTAWLYD-QEGEVEKPFIKTG-FPVSVEKTTNSNRKNQLD---------TGRRRRQFDEESLESFSSMPDPVDPTTVTKTFKT-RKASAQASLASKDKTPK-SKSKKRHSAQLKSR-VKNTGYESASVSSTCEPCKSR---NRHSAQTEEPVQAKVFSRKNLEQLEKIIK-YSRSTEISS----------------------------ETGSDFSMFEALRDTIYSEVATLISQNESHPHFLIELFHELQLLNTDYLRQRALYALQD---IVTRHISENH-----EKEGENV-KSVNSGTW-IA-SNSELTPSESLATTDDETF-----EKNFERET---------HKISEQNDADNASVMSVSSNF---------EPFATDDL--GNTVIHLDQA-----LARMREYERMKTEAESSTNIR--CTCRILEDEDGAAATSMVTNLEETP-IENHGSQQPVSEVSTV--------PCPRIDTQQLDR-----QIKAIMKEVIPFLKEHMDEVCSSQLLTS---VRRMVLTLTQQ--------NDESK---EFVKFFHKQLGSIL-QDSLAKFAGRKLKDCGEDLLVEISEV-LFN-ELAFFKLMQDLDNN---SITVKQKCKRKIEAAGVIQSYAKEAKRILEGDHGSPAGEIDDED---KDKDETETVKPTQ----TSEIYDGDG-PKNVRSDVSDQEEDEES-EECPVSINLSKAE-TQALTNYGSGEDENEDEEIEEFEEGPVDVQTSLQ-ANTEATEETEHDDQVLQHDFEKSGESKNVPSEQDPT-TS----------KGNTPVKPCYLNILENEQPLNSAVQKDSLTTI-DSSKQPNPLPLPLPEIETLVPTVKEVKSAQETPESSLAGSPDTESPVLVNDYEAESGNISQKSDEEDFVKVEDLPLK--LTI-YSEADLRKKMVEEQEKNHLSGEILCEMQT-----EELAGNSQTLKEPETVG-AQSV--------------------  
------------------------------------------------------------------------------------------------------------------------------------------------------------------------------------------------------------------------------------------------------------------------------------------------------------------------------------------------------------------------------------------------------------------------------------------------------------------------------------------------------------------------------------------------------------------------------------------------------------------------------------------------------------------------------------------------------------------------------------------------------------------------------------------------------------------------------------------------------------------------------------------------------------------------------------------------------------------------------------------------------------------------------------------------------------------------------------------------------------------------------------------------------------------------------------------------------------------------------------------------------------------------------------------------------------------------------------------------------------------------------------------------------------------------------------------------------------------------------------------------------------------------------------------------------------------------------------------------------------------------------------------------------------------------------------------------------------------------------------------------------------------------------------------------------------------------------------------------------------------------------------------------------------------------------------------------------------------------------------------------------------------------------------------------------------------------------------------------------------------------------------------------------------------------------------------------------------------------------------------------------------------------------------------------------------------------------------------------------------------------------------HHFLVDISEV-LFH-KLAFFKLVQNLDSN---SVSVKQRCKRKMEKA---ESSAKG------------------------HKDKTERVKQVPLPPPP-KVFSGKEDFKNTMSKMFDQKQDEEI-KGCPVFINLSKAE-TWTLMNYGNGEDETKDEEMKEFEEGSVNLQTYLQ-YNNE---EHLSK-TVLQNEFEKPMGNENTPSEHN---------------HNDSPMMPCSLNVLDNEQQLTTMSPQEELK--------LFF-FYHMAEVEASMSKSTPVKSTLETPKISVAESPNTESPMLLND------------------------------------------------------------------------------------------------------------  
MATGGG---------------------PFEEGRNGQDLPNW--SNESVDDRLNNMDWGGQQKKANRSAEKNKK--------KFGVESEKRVTNDISPESSPGVGRRRT---------------KTPHTFPHSRYMTQMSVPEQAELEKLKQRINFSDLDQRSIGSDSQGRATAANNKRQLA-ENRKPYNFWSMQINTNKSKDVGTSPQARETSGSSQCKELFASALSKDLLQN-----------------CQVS-EEDGRGEPAMDSSQVVSRLVQIRDYIAKASSMRDDLVEKNERS----------------------------------ANVERLTHLIDHLKEQEKSYLKFLQKMLAR-------------------------------------------------------------------------------------------------------------------------------------------------------------------------------DPQQEPREELENLKKQHDLLKRMLQQQEQLRALQGRQAALLALQ-----------HKAEQAIAVMDDSVVTETTGSISGISITS-----------------ELNEELNDLIQRFHNQLHDSQAPAVPDNRRQAESLSLTREVSQSRNS-------------------SVSEHLSDEKVQLFSKMRVLQEKKQKMDKLLGELHTLR--DQHLNNSAFLP-S----PSPQRSVD-QRSIGSVASAPVGLVPATNGDSASHVPSAAYPPDTVVSQNESENEERLNPSEKLQKLNEVRKRLNELRELVHYYEQTSDMMTDAVNENTKDEEE--------TEESE-----YDSDHENPGPVTNIRN---PQGAAGWTEVNSNTN----AQCVTNNRDSRSVTTNCEINNRTATNLCPRNIPSSSSPSSLSSFLACHSSTNTFPSSDTS-----LPVFYF----------EASLSSHRSSLGDDAPEDAEFEHKISRLMAAKQKLRQLQDLVAMVQDDDGD-PGATPAGASNLEDF-------------FPVEEQETKQRSNNTR--------ANTSKTQKDAGINGKA---------------REKFYEAKLQQQQRELKQLQEERKKLIEIQEKIQALQKA---CPDLQLSAANVSNCG--TSKKYTPAVTSTPAVNDGQNSPTNK---SGFVAEDPSATVAVAADNELWSEMRRHEILREELRQRRKQLEALMAEHQRRRDLTETASTAAVSVRSDGSENPCT--PQQSRT-E-KTMATWGGSTQCALDEEEGD---------EDGYLSDG-----VVRGEEEEEEEE-------QDASSNDNFPVYPPNSVTHNPYGVKENKDRWKA-----------SRPLSADGNYRPLAKARQQ-NVSMRRQENLRWVSELSYVEEKE---QWQEQINQ--LKKQLDFSVSICQTLMQDQQTLSCLLQ------TLLTGPYSVMPS-----------------------NVASPQVHLIMHQLNQCYTQLTWQQNNVQRLRQMLSELMRQHEHRGS-DKAGRKGRGSSAPPP-----PSPTLFCPFSFPAQPMSLFNLPGLSHFSSFAPGMNFNPLFTSNFGDFAQN-IPTSSDQQ---QPADQNIPGKTEYVAFPKPFESSSSIGAEKQR-NQKQPEEEVENSRPTWLFE-QEGSLEKP-FVKTGFAVSVQKTASNSHQNQLDATVATTTASTSRRRRQFDEESLESFSSMPDPVDPTTVTKTFKT-RKASAQASLASKDKTPK-SKMKKKNSTQLKSR-VKNTGYESASMSSTCEPCKSR---NRHAVQTEEPVPAKVFSRKNHEQLEKIIK-YSRSTEMSS----------------------------ETGSDFSMFEALRDTIYSEVATLISQNESRPHFLIELFHELQLLNTDYLRQRALYALQD---IVNRHISESN-----EKGGENA-KSVNSATW-IA-SNSELTPSESLATTDDETF-----EKNFERET---------CKMSERNDADNASTLSTSSNF---------EPFATDDL--GNTVIHLDQA-----LARMREYERMKIEAESNLSTEGAGAATAITASPSATTA--STLEESPKT-ETRSAQQPINEVSTV--------PCPRIDTQQLDR-----QIKAIMKEVIPFLKEHMDEVCSSQLLTS---VRRMVASLSLR--------ERESDHVPQFIYLFSCRLTSLVSRDSLAKFAGRKLKDCGEDLLVDISEV-LFN-ELAFFKLMQDLDSN---SVSVKQRCKRKMEAAAVIQSYAKEAKRILEGDLGSPAGEIDDED---KDKDETETVKQVPPPPPPPEVYNGNEAPKNTRSDVSDQEEDEES-EGCPVSFNLSKAE-TQALTNYGSGEDENEDEEMEEFEEGPVDVQTSLQ-ANNEVPEENEQD-QVLQNEFEKPLENESIPAEQEPTNGQSD--------HDDSPTMPCYLNVLDNEPQLSGPSPLDSTRTPVGPPEELKP-SLPITEVEASMPRNTQVKSASETPESSVAGSPDTESPVLVNDYEAGSGHLSQKSDEEDFVTVEDLPLK--LTI-YSEADLMKKIGAEEQSNHLSEEILT--VTHT---EELAGDPQTLKEPETVT-AQSV--------------------  
-------------------------------------------------------------------------------------------------------------------------------------------------------------------------------------------------------------------------------------------------------------------------------------------------------------------------------------------------------------------------------------------------------------------------------------------------------------------------------------------------------------------------------------------------------------------------------------------------------------------------------------------------------------------------------------AVPDNRRQAESLSLTREVLHSRTS-------------------SASQPPADEKVQLFSKMRVLQEKKQKMDKLLGELHTLR--DQHLNNSSFVPVT----ATLQRSADPQRSPAVAAAAPAPRGPAAGGGSNRPRSPGPFPPESRASQNDSEEEEHLNPTEKLQKLNEVRKRLNELRELVHYFEQTSDMMTDAVNENTKDEEEEEEEDEEETEESE-----DDCTRETPQPVTNLRN---AQGTASWGEGNGNTV----APGAANNRDGRSLNTNCEINNRS-ANLRTLNMSSTLDRRYNS---ESDRDGRVVREEEEEEEEGAVEEDGV---------SEASLSSCRSSM---AE-DAEWEQKINRLISAKEKLRHLQDLVAMVQDGEAE-PATESASAPGPSEF-------------FTAGEELAEQHPDNAR--------DGAQATERDARPDDKS---------------R-KFYEAKLQQQQKELKQLQEERKKLIDIQEKIQALQKE---CPDLQLSARNVGHR---STKRRIPVVTSTPAVTANGNPPSRR---AVFEPAD-----SSAVDNELWSEMCRHEILREELRQRRKQLEALMAEHQRRRELAEATSVVAASIRSEGSETQGT--PQRSRS-E-KTVATWGGSPQCVLDEEDRG---------EDSYLSDGIVRGAVAADEEEEEEEEEG---DDQETSSNENFSVHPRQSLSRSPYEPKGNKDRWKK-----------SRPLSAEGNLRPVAKARQQ-NISMRRQENVRWMSELSHVEEKE---QWQEQINQ--LKKQLDFSVSICQTLMQDQQTLSCLLQ------TLLTGPYSVMPS-----------------------NVASPQVHLIMHQLNQCYCQLTWQQSNVQRLKQMLSDLMRQQDE-HP-EKPRRKERGS-APPP-----PSPSLFCPLGFPSQPMNLFPMPGFAACSPFAPGMNFNPVFPSNVGDFPQNTSPHNGQQS----SSGQDTSGKTEHVAFPKPFESNSSLGAEKQR-NTKPPEEEADPPRPAGLAADQEGGMDTQPPVKSGFAVAVRKMASN-RKSQLD---------TSRRRRHWDEESLESFSSMPDPMDPTTVTKTFRT-RKAAARASLAAREKTPK-PKSRKRASAQPKNK-EENSGYESASLSSTCEPCRSRNWNSSQP---EELGPAQVLSGKNPEQLEGTTP-DTRSTEISS----------------------------------------------------------------------------------------------------------------------------------------------------------------------------------------------------------------------------------------------------------------------------------------------------------------------------------------------------------------------------------------------------------------------------------------------------------------------------------------------------------------------------------------------------------------------------------------------------------------------------------------------------------------------------------------------------------------------------------------------------------------------------------------------------------------------------------------------  
MATGGG---------------------PFEEGMNDQDLPSW--TNESLDDRLNNTDWGGQQKKANRSSEKNKK--------KLSGEGETRLTNDISPESSPGMERRKT---------------RTSHSFPHARYMTQMSVPEQAELERLKQRINFSDLDQ----------------------------------INTNKSKDPASGSQKKEGGVSAQCKELFGAALSKDLLQN-----------------CQVSAQEDGRGEQAMDSSQ-------------------------------------------------------------------------------------------AR-------------------------------------------------------------------------------------------------------------------------------------------------------------------------------DPQQEAKEELENLKKQHDLLKRMLQQQEELKALQGRQAALLALQ-----------HKAEQAIAVLDDSVVTETTGSVSGVSLTS-----------------ELNEELNDLIQRFHNQLHDSQTQSVPDNRRQAESLSLTREISQSRNS-------------------SMSEHQSDEKAQLFNKMRMLQGKKQKMDKLLGELHTLR--DQHLNNSSFFPAS----GSPQRSVD-QRSTTSAASGPVGIATVVNGETNSLAS-APYPPDSLVSQNESEEDENLNPTEKLQKLNEVRKRLNELRELVHYYEQTSDMMTDAVNENTKEEEE--------TEESE-----SDSEHEDPQPVTNIRN---PQGISSWSEINSNSN----VQCGTNNRDGRHLNTDCEINNRSAANIRTLKMSSALD-CHNR---ENDKRLDLPQGEDDE-----VEEDRV---------SEDSMSSHRSSLADVAG-DAEFEQKINRLIAAKQKLRQLQNLAAMVQDDDPE-PQGAIANASNISDL-------------LGEVEETKQQ-PNNVR--------ASSNKLKKDVRLNEKA---------------REKFYEAKLQQQQRELKQLQEERRKLFEIQEKIQVLQKA---CPDLQLS-AGLGNC---PANRQTSQATSTPAMNER-NTAG-K---PLFVCDE-----SVPVGSELWSEMRRHEILREELRQRRKQLEALMAEDQRRRELAETISTVAASVKSEGSEAQCT--PQQSRT-E-KTMATWGGSTQCALEEENGD---------EDGYLSDG-------VGQAEEEEE--------DASSLNDSFSVYPNNNIPENGYFVKGNKDRWKN-----------CRPLSADGNYRPVSKTRQQQNISMRRQENFRWMSEFPYVEEKE---RWQEQINQ--LKKQHEFSVSICQTLMQDQQTLSCLLQ------TLLTGPYNMMPN-----------------------NLASSQINLIMHQLNQCYTQLNWQQNNVQRLKQMLSDLMQQQEQ-CQ-EKPSRKERGSSAPPP-----PSP-VFCPFNYPPQPVNLFSIPGFTNFSSFAPGINCNPVFPCGFGDFAHNVSPRNSEQQGQQHPLDPNTSGKTEYMAFPKPFESSSSNGGEKQR-NHRQPEEEMEK-RSTWLDDSQEMKKDDQSQLNAGFAVSVQNIASG-HKSQCD---------MNRKR-EFDEESLESFSSMPDPIDPTTVTKTFRS-RKASAQASLASKDKTPK-SKNKRKNSSQLKGR-IKNTGYESASASSVCEPCKNT--KSRHS---DDVVHAKVFSKRNQEQLEKIIK-YSRSTEMSS----------------------------ETGSDLSMFEALRDTIYSEVATLISQNESRPHFLIELFHELQLLNTDYLRQRALYALQD---IVTRHLCEKN-----EKG-KCA-KSLNSATW-VA-SNSELTPSESLASTDDETF-----GKNFSTEA---------CQNCEQPDADNGSTMSTSSNF---------EPFATDDL--GNTVIHLDKA-----LSWMREYERMKVEAESTLDSE--GCSSNFQGASTAKLE-------GPGTGECQSVPQS-GDVSSV--------PCPRIDTQQLDR-----QIKAIMKEVIPFLKEHMDEVCSSQLLIS---VRRMVLTLTQQ--------NDESK---EFVKFFHKQLGSIL-QDSLAKFAGRKLKDCGEDLLVEISEV-LFN-ELAFFKLMQDLDNN---SISVKQRCKRKIETTEVMQSYAKE------------------------DKDETETAKQVP----DSEVCAGNGVPESIRSDASDQEEDEES-ESGPVAISLSKAE-TQALTNYGSGEDENEDEEI-EFEEGPVDVQTSLQ-ASSET-TENEQT-SNQELSKAKSSEILS--SEQEPVNVKGE--------QDVAAAVHDYLSVMENTPDLTVNSP-ESFITATVKTEGSSS-SLAVNETQTPDTTCAENKS-AASSESSMAGSPDTESPVLVNEYEPGSGNVSQKSDEDDFVKVEDLPLK--LAV-YSEADIMKKMETEAQTKSLSDELLDGDGAQD---QELVGDAQTLKEP-----------------------------  
MATGGG---------------------PFEEGMNDQDLPSW--SNESLDDRLNNTDWGSQQKKANRSSEKNKK--------KLGGEAETRLTNDISPESSPGMGRRKT---------------RTPHTFPHARYVTQMSVPEQAELERLKQRINFSDLDQ----------------------------------INTNKSKDPVSGPQKKESGEPLQCKDLFGAALNKDFLQN-----------------GQLSVQEDGRGEPAMDSSQ-------------------------------------------------------------------------------------------AR-------------------------------------------------------------------------------------------------------------------------------------------------------------------------------DPQQEAKEELENLKKQHDLLKRMLQQQEQLKALQGRQAALLALQ-----------HKAEQAVAVVDESVVTETTGSVSGVSLTS-----------------ELNEELIDLIQRFHNQLHDSQTQSVPDNRRQAESLSLTREISQSRNS-------------------SVSEHRSDEKAQLFNKMRMLQGKKQKMDKLLGELHTLR--DQHLNNSSFFPAS----SSPQRSVD-QRSTTSAASAPVGVVTVINGESNSLAS-APYPSDSLASQNESEEDDNLNPTEKLQKLNEVRKRLNELRELVHYYEQTSDMMTDAVNENTKEEEE--------TEDSG-----SDSEHGDPQPVTNIRN---PQGISSWSEINSNSN----VQCGTNNRDGRHLNTDCEINNRSAANIRTLKISSTLD-CHNR---EDDKDTDLPQGEDDE-----VEEDRA---------SEDSISSHRSSVGDVAG-DAEFEQKINRLMAAKQKLRQLQNLAAMVQDDDPE-PQVLTGNASNMGDF-------------LGEMEEIKQQ-PNNVR--------VSTNKLQKDVGLNEKA---------------REKFYEAKLQQQQRELKQLQEERRKLMEIQEKIEVLQKA---CPDLQS--AGLGNS---PANRQTSPAASTPAMNEC-NTAG-K---LLLDFGE-----SVPVGNELWSEMRRHEILREELRRRRKQLEALMAEHQRRRELAETISTVAASVKSEGSEIQHT--PQQSRT-ENRTMATWGGSTQCALDEEDGD---------EDGYLSDG-------LDQAEEEE---------DAPSINDSFSIYPNNQIPESVYYLKGNKDRWKN-----------CRPLSADGNYRPMSKTRQQQNISMKRQENFRWISELSYVEEKE---QWQEQINQ--LKKQLEFSVSICQTLMQDQQTLSCFLQ------TLLAGPYNMMPN-----------------------NVASSQVHLIMHQLNQCYTQLSWQQNNVQRLKQMLNDLMHQQEQQCQ-EKPSRKERGSSAPPP-----PSP-VFCPFSFPPQPVNLFNIPGFTNISSFAPGINYNPVFPSGFGDFAHSGFPQSSEQQ--QHPLDHNTSGKTEYMAFPKPFESSSSNGAENQR-SHRQPEDEVEK-RSAWLNDSQEVKKDDQSQLKAGFPVSVQSIASG-HKNQSD---------TSRRR-NFDEESLESFSSMPDPVDPTTVTKTFKS-RKASAQASLASKDKTPK-SKNKRKNSSQLKGR-IKNTGYDSASASSVCEPYKST--KSKHS---EEVVHAKVFSKKNREQLEKIIK-YSRSTEMSS----------------------------ETGSDLSMFEALRDTIYSEVATLISQNESRPHFLIELFHELQLLNTDYLRQRALYALQD---IVTRHLSENN-----EKG-KCI-KSLNTATW-IA-SNSELTPSESLASTDDETF-----DKNFPTEA---------CHDCEQNDADNGSTMSTSSHF---------EPFATDDL--GNTVIHLDQA-----LARMREYERMKIEAESTLGSE--GCSSNFQGATAAKLE-------GPSTSECLSVPQS-SEASAV--------PCPRIDTQQLDR-----QIKAIMKEVIPFLK-----------------------------------------------------------------------------------------------------------------------------------------------------------DKDETEIVKPVQ----GLETYDGNEVPESIKSDASDQEEDEES-ESGPVAISLSKAE-TQALTNYGSGEDENEDEEI-EFEEGPVDVQTSLQ-ASNETATENEQT-SSQELSKTKGSDILS--SEEQSVNVKGE--------QDAATVLPHYLNVVENTPPLAVNTP-ESFITASKKAEESSS-SLPENETQMLDAACVVNKSSAGSSESSMAGSPDTESPVLVNEYEAGSGNISQKSDEDDFVKVEDLPLK--LAV-YSEADLVKKIAAEAQTNSLSDELLDGGGQQD---RELVGDAQTLKEPETFG-AQSA--------------------  
MATGGG---------------------PFEEGMNDQDLPSW--SNESLDDRLNNTDWGCQQKKANRSSEKNKK--------KLGGEAETRLTNDISPESSPGMGRRKT---------------RTPHSFPHARYMTQMSVPEQAELERLKQRINFSDLDQRSIGSDSQGRATAANNKRQLN-ESKKPFNFLSLQINTNKSKDPVSGSQKKESGEPLQCKELFGAALNKDFLQN-----------------GQLSIQEDGRGEPTMDSSQIVSRLVQIRDYIAKASYMRDDLVEKNERS----------------------------------ANVERLSHLIDHLKEQEKSYLKFLQKMLAR-------------------------------------------------------------------------------------------------------------------------------------------------------------------------------DPQQEAKEELENLKKQHDLLKRMLQQQEQLKALQGRQAALLALQ-----------HKAEQAVAVVDDSVVTETTGSVSGVSLTS-----------------ELNEELIDLIQRFHNQLHDSQTQSVPDNRRQAESLSLTREISQSRNS-------------------SVSEHQSDEKAQLFNKMRMLQGKKQKMDKLLGELHTLR--DQHLNNSSFFPAS----SSPQRSID-QRSTTSAASAPVGVVTVINGESNSLAS-APYPPDSLASQNESEEDDNLNPTEKLQKLNEVRKRLNELRELVHYYEQTSDMMTDAVNENTKEEEE--------TEDSG-----SDSEHGDPQPVTNIRN---PQGISSWSEINSNSN----VQCGTNNRDGRHLNTDCEINNRSAANIRTLKMSSTLD-CHNR---EDDKHADLPHGEDDE-----VEEDRA---------SEDSMSSHRSSLGDVAG-DAEFEQKINRLMAAKQKLRQLQNLAAMVQDDDPE-PQVLTANASNMGDF-------------LGEMEETKQQ-PNNVR--------VSTNKLQKDAGLNEKA---------------REKFYEAKLQQQQRELKQLQEERRKLMEIQEKIEVLQKA---CPDLQVGRLSLPSY---PANRQTSPATSTPAMNEC-NTAG-K---PLLEFGE-----SVPVGNELWSEMRRHEILREELRRRRKQLEALMAEHQRRRELAETISTVAASVKSEGSEAQRT--PQQSRT-E-KTMATWGGSTQCALDEEDGD---------EDGYLSDG-------LDQAEEEE---------DAPSMNDSFSAYPNNQIPESVYYLKGNKDRWKN-----------CRPLSADGNYRPMSKTRQQQNISMRRQENFRWISELSYVEEKE---QWQEQINQ--LKKQLEFSVSICQTLMQDQQTLSCFLQ------TLLAGPYNVVPN-----------------------NVASSQVHLIMHQLNQCYTQLSWQQNNVQRLKQMLNDLMHQQEQQCQ-EKPSRKERGSSAPPP-----PSP-VFCPFSFPPQPVNLFNIPGFTNISSFAPGINYNPVFPCGFGDFAHSGFPQSSEQQ--QHPLDHNASGKTEYMAFPKPFESSSSTGAENQRRSHRQPEDEVEK-RSTWLNDSQEVKKDDQSQQKAGFPVSVQSIASG-HKNQSD---------TSRRR-NFDEESLESFSSMPDPVDPTTVTKTFKS-RKASAQASLASKDKTPK-SKNKRKNSSQLKGR-IKNTGYDSASASSVCEPCKST--KSKHS---EEVVHAKVFSKKNREQLEKIIK-YSRSTEMSS----------------------------ETGSDLSMFEALRDTIYSEVATLISQNESRPHFLIELFHELQLLNTDYLRQRALYALQD---IVTRHLSENN-----EKG-RCI-KSLNTATW-IA-SNSELTPSESLASTDDETF-----DKNFPTEA---------CQDCEQNDADNGSTMSTSSHF---------EPFATDDLDIGNTVIHLDQA-----LARMREYERMKIEAESTLDSE--GCSSNLQGATAAKLEV-SEENQGPSTSECLSVPQS-SEVSAV--------PCPRIDTQQLDR-----QIKAIMKEVIPFLKEHMDEVCSSQLLTS---VRRMVLTLTQQ--------NDESK---EFVKFFHKQLGSIL-QDSLAKFAGRKLKDCGEDLLVEISEV-LFN-ELAFFKLMQDLDNN---SISVKQRCKRKIETTEEIQSYAKEAKKGLQVDVCSSTEDID-ED---KDKDETETVKPVQ----GLETYDGNEVPESIKSDASDQEEDEES-ESGPVAISLSKAE-TQALTNYGSGEDENEDEEI-EFEEGPVDVQTSLQ-ASSETATENEQI-SSQELSKTKGSDILS--SEQQSVNVKGE--------QDAATILPHYLNVVENTPPLPVNTP-ESFIAASMKTEESSS-SLPGNETQMLDTACVVNKSSAGSSESSMAGSPDTESPVLVNEYEAGSGNVSQKSDEDDFVKVEDLPLK--LAV-YSEADLLKKIASEAQTNSLSDELLGGGGEQD---RELVGDAQTLKEPETFG-AQSA--------------------  
------------------------------------------------------------------------------------------------------------------------------------------------------------------------------------------------------------------------------------------------------------------------------------------------------------------------------------------------------------------------------------------------------------------------------------------------------------------------------------------------------------------------------------------------------------------------------------------------------------------------------------------------------------------------------------------------------------------------------------------------------------------------------------S----GSPQRSVD-QRSATSVASAPVGITAGVNEEPNSLASSVVYHPDSVTSQNESEEDDNLNATEKLQKLNEVRKRLNELRELVHYYEQTSDMMTDAVNENTKGEEE--------TEDSE-----CDSDLENPQPVTNIRN---PQGISNWSEINCNSN----LQCGTNNRSGSHLNTACEINNRS-ANIRMLNMPSTVDCHYNR---EDD-SIEIPQGEDDDIG---PEADRA---------SEASLSSRRSSIVDEAPVDAEFEQKINRLIAAKQKLRQLQNLVAMVQDDEGA-P--ESAGRDNVDQY-------------YLAEEEKEQQQPNNVL--------TSANKSEKAVVLNEKA---------------REKFYEAKLQQQQRELKHLQEERKKLIKIQEKIQVLQKA---CPDLQLS-SSLDNC---PGN------TSAPTANEI--NVGNK---AVDQPEE-----KAMVDSELWSEMRRHELLREELRQRRKQLEALMAEHQRRRDLADTTSAGAASVKSDGSETQCT--PQQSRT-E-KTMATWGGSTQCALDEDEGD---------EDAYLSDG-------LAQAEEEEEE-------EQDSSSNELSICSNN-VDQSSYCAKEDIQSWKN-----------PRPLSADGNYRPLAKAKQQQNISMRRQENHRWMSELSYVEEKE---QWQEQINQ--LKKQLEFSVSICQTLMQDQQNLSCLLQ------TLLTGPYSVMPS-----------------------NVASPQVHLIMHQLNQCYSQLTWQQNNVQRLKQMLNDLMHHQEQ-QQ-AKTAKKKKSSSAPPP-----PSPTVFCPFSFPSQTMNLFNMPGFTNFSSFPPGINFSPMFPPGLGDFSQNCTAQNNEQQ----PLEHNTTGKTDYMAFPKPFESSSTPGAEKPR-GQRQSEEELES-RSAWLNEHQGGGKEKPLFLKTGFAVPVQNTACGSQKGQPD---------TGRRR-EFDEESLESYSSMPDPVDPTTVTKTFKP-RKASAQASLASKDKTPK-SKSKR-STSQFKNR-PRTNGFESASASSTCEPYKRN--RSRHSTRKEEATQAKVFSKKNREQLEKIIK-YSRSSEMSS----------------------------ETGSDLSMFEALRDTIYSEVATLISQNESRPHFLIELFHELQMLNTDYLRQRALYALQD---IVTRHASDDN-----EKG-ECP-KPLSSAAW-AG-SNSELTPSESLATTDDETF-----EKNFDQGA---------CRECEKNDADNGSTMSISSNF---------EPFATDDL--GNTVIHLDQA-----LARMREYERMKMAAESNLDSE-DGCS-NVRSVSSSKPEDKVTYFKQHYVVESHSAAQP-TEVPAV--------PCPRIDTQQLDR-----QIKAIMKEVIPFLKEHMDEVCSTQLLTS---VRRMVLTLTQQ--------NDESK---EFVKIFHKQLGSIL-QDSLAKFAGKKLKDCGEDLLVEISEV-LFN-ELAFFKLMQDLDNN---SLTVKQRCKRKVEADGTMQSYAKEGTVELTFILDKMCESKS-CK---CDKDETETLKQVH----ESAIFNNREVPRTIRSDASDQEE-EEN-ESCPVSISLSKAE-TQALTNYGSGEDENEDEEIDEFEEGPVDVQTSLQ-ANNETTAENEQE-QISQPKSENSDDCITVLSEQESV-AKGD--------LDSTVVVPQYLNIIEDTQAIPVNAA-EDLNPSTVVTEEQDV-TLAENESHALVTVHEEPKSSSGTPESSVVDSPDTESPVLVNEYETGSGNNSQKSDEDDFVKVEDVSLK--LAV-YSEEELMKTIVEEAQTNSITSEI-----------------------------------------------------  
MAAGGG---------------------PFEEGMDDHDLPNW--SNESLDDRLNNTSWGGQPKRPNKSSEKNKK--------KVSSESETRLTNDISPESTPGVGRRKS---------------KTPHSFPHTRYMTQMSVPEQAELEKLKQRINFSDLDQRSIGSDSQGRATAANNKRQLN-ENKKPFNFLSLQINTNK------------------------------------------------------------------------------------------------------------------------------------------------------------------------------------------------------------------------------------------------------------------------------------------------------------------------------------------------------------------------------------GKDGATSSQT-----------H---------------ETSG--------------------------S---------------------------------------------------------------------------------------------------------------------------------------------------------------------------------------------------SQYKDLIT----------------------------------------------------------------------------------------------------------------------------------------------------------------------------------------------------------------------------------------------------------------------------------------------------------------------------------------------------------------------------------------------------------------------------------------AA----------------------------------------------------------------------------------------------------------------------------------------------------LSKD-------------------------------------------------------------------------LLQ------N------------------------------------------------------------------------------------------------------------------------------------------------------------------------------------------------------------------------------------------------------------------------------------------------------------------------------------------------------------------------------------------------------------------------------------------------------------------------------------------------------------------------------------------------------------------------------------------------------------------------------------------------------------------------------------------------------------CP--------------------------------------------------------------------------------------------------------------------------------VS----------------IE-----------------------------ED---------------------------------------------------------------------GRGE------P-------AVDSSQVL---------------------------------------------------------------------------------------------------------------------------------------------------------------------------------------------------------------------------------------------------  
MATGGG---------------------PPDEALSDQDLPNW--SHESLDDRLNNMDWKGQ-KKANRSTEKNKK--------KF-VECDLRLTNDISPESSPGVGRRRA---------------RTPHTFPHTRYVSQMSVPEQAELEKLKQKINFSDLDQRSIGSDSQGRATAANNKRQLA-DNRKPFNFLPLQLNTNKEK-SSKSPPKRESSTISSTKDFLASAFNKDFLSN-----------------SQAFLEEESQREPAIDSSQVVSRLVQIRDYITKASSLRDDLVEKNDLS----------------------------------ANIDRLSNLIEHLKFQEKSYLKFLQKMLASEN-----------------------------------------EEDD---VRTVDSAMGSGSVAESTSLNIDVPSEASDTTG--------------------------------------------------------------------------------------------VDPQQEAKEELKNMKKQHALLTRMLQQQEQLRALKGRQAALLALQ-----------HKAEQTIAKMDESVVTETTGSVSGLSLAS-----------------ELNEELNDLIQRFHNQLHDSEDPPVPDNRRQAESLSLAREVYRSRNS-------------------SSSDTPLEDKSPLFNKVGVLLEKKQKMDTLLGELHTLH--DQHLNNTAFMASS----VSPRRSTD-QRTLGCAISAALSSD---NRAANSPITVGTYQAAS---VNESEDEENRNPAEKLKKLKEVRKRLNELRELVHYYEQTSDMVTDAMNENTKDEDE--------TEDSE-----YESEQEDDEPATNIRN---PQYRSGWAQMNINSN----AQSGTNNRDERQLNTECEINNRSAVNLRSINMSSALDCLYNI---EHSDKEEDGNGEP---DDEDAEDQG----------SRASLSSQNS--VADDVQNVDFEQKFNRLVAAKQKLKQLQDLVAMYGDDSES--EPVAPERSFSGDQ-------------LPPETTTLKQQPNNTR--------PNASK-AKDIALKEQA---------------REKFYESKLQQQQRELRQLQEERKKLIEIQEKIQTLRKA---CPDLQLSTSSAGTNPRNRQNRQMTTTTSTPEVTTN-SKNVVP---AMPDPED-----SSSIDNEVWSEIRKHQILREDLRQRRKQLETLMAEHQRRQGQTDTSG---ASVRSDDSDTQGF--QQQTRT-E-KTTATWGGSTQCALDEDDDDN-------EDDDDDDEGCLSD--VHQIDIEDDE--------QGNTSCEN-SSYHQNSIRKTMLNGRSSKNGWKN-----------QRPFSVEGNHRPSPKTRQQQNVSMRRQENYRWMSELSHVEEKE---HWQGQIDQ--IKKQLDYSTNICQTLMRDQQTLSYLLQ------SMITSPYSVLPS-----------------------NVGAPQVQLIMHQLNQCYTQLNWQQSNVLRLKQMLNDLLAQQQQHLQGESQQREDRGSSAPPPL----TSPNIFTNFGFLPPTMNLLNMP-FGSIPSVVPGVNFNPGFPHGFENFAQNVTSHADTPL---QPHDQNTSGKTEYMAFPKPFESHTSNSTEKER-NEPKKPDEGEQGRRIWVENHQKNDQEK---KAGCFGAGISEGSGTSAK-LAE---------ESWKGKQFDEVSVESLSSMPDPVDPTTVTKMFKS-RKASAQASLASKDKTPK-AKNKKRKIFHQKSKGIKSIGASGFNVS---EPNQTG---CRH-AQTEGATVGKVTTTSSAQRTE---K-ESKATELSS----------------------------EAGSDVSMFETLRDTIYSEVATLISQNESRPHFLIELFHELQLLNTDHLRQKALFALQD---IVTRRITESN-----VKKHETCTKPLESAGW-MA-SNSELTPSESLVTTDDELY-----AKNSDGPA---------CQEIEQNDADNISSLSTSSNF---------EPFATDDL--GNTVIHFDQA-----LARMREYERMKNETEDSLVAD--CCNHL----NAAASSLEGNHLQGTNDESRGHAQHSADDASAI--------PCPYIDSKQLDR-----QIKAIMKEVIPFLKEHMEEVCSPNLLTS---IRRLVLTLTQQ--------NDESK---EFVKFFHKQLGSIL-QDSLAKFSNKKLKDCGEDLLVEISEV-LFN-ELAFFRLMQDLDNN---SAAVQGKVSKKPDAAVVLESFTKEGDKLCQDENFSATREIDDED---KDKDDTEAAQESR----NIDAEVLSG-----KSDIS--EEDDVN-ENLPVSISLSKAE-TQALTNYGSGEDENEEEENYEFEARPVDVQTSLE-TSSEIADETEKEEQFMKWVLIFN----------------------------------FYFHTVSNTLQELS-IHEDQLKSDSDVLPHSSLLLSNGNTTDFSGTAPVNVKSPVDSPGTSGAGSSDTESPVLVNDFETGSGNLSQKSDEDDFVKVEDLPLK--LTMPMPKEQIMKEMEEEEKKNNLCDEILNTSDEGNG-ADQLAGDPGALKEPGKHDWKQSL--------------------  
MATGGT---------------------PFDDG--AEELHNWTVTNGSLEDRLNNLDWGVQQKKANRSSEKNKK-------KLSTSVVESRLTNDISPESTPGAGRRRA---------------RTPHSFPHIKYTTQMSVPDQAELEKLRQRINFTDLDERSIGSDSQGRATAANNQRQLAGENKKPYNFLPLHVNTNKSKALLPPSSSAPATPAITKETKKQSPGRRDMLTP----------------RGPLAHREYGRGEQRIDSSQVVSKLVQIREYISKASSMRDDLVEKNDVP----------------------------------ANVERLSNLIDHLKEQERSYLRFLQKMLTRED-----------------------------------------DEDD---VRTLDSAMGSGSLAESTSLNVEVRSSDASTAT--------------------------------------------------------------------------------------------VTVRADQKEELENLRKQHELLKKMLEQQEQLRALQGRQEALMAMQ-----------DSAEQTLAVIEDTVVTETTGSVSGLSITS-----------------ELNDELNDLIQRFHNQLHDSQTKAVPDNRRQAESLSLSREVCWSRAP------------------------HAVAANVKLTKLQELQDKKQTMDKILRELHSLR--DQTLNNNSCKSRG----LSTRCSVS-------ALDSP-ECASVLCSNAASASTPFH--PSRTQHQNSS------NSTDKLRKLKEVHKRLNELRELVQYYEQTSDMMVDAVNEN--------------TEDGSMFEAMFDSEQENRQPVTNIRN---PHHSGNWTDLNSLTNRHSVRSSATNNRDGR-LNTQCEINNRSAANVRSLNIPSAIECQYNR-----DTSFNQVKDED--EDGLDNEEGAQ----AAAADSDASGSSRRSSLGN----NGGFSQKVHR-QTAKQKLRQLQELVAMVQS-------------DDTDGT-------------TANEDEALHQQPNNTRAPVLGALGADSKQNSRELALSSKA---------------REKLYEEKLRQQKQELMQLHEERQRLIDIQGKIQDLQWA---CPDLQ---------QG-LLRKVPVA-VSTPAPVLASSSSGPQINSTGLKPTAPEPASPSVADNELWSEMRRHQICREELRQRRKHLESLMAEHQRRSGLGD------SPRRADDPEGLAT--PSQPVSRDERTMATW-GSTPCHLDDEDDDDDD-----DDEEYQSETG-------AEEEEEQDD------CVESSSEDDIPIYAS-NRNQCSYSNRKNQGRSDK-----------QQQQQHQSRGLD-HTATSQHGA-TRRQENLRWASELSFAEGSG---QWPEQVSQ--LQKQLDFSTSMCQTLLQDQQTLSYMLQ------TLLTGQYSALPN-----------------------NLSSPQVHLVMHQLNQCYTQLAWQQNNVQRLKQVLNDLLRQQQQASSSAAAWQTQKHGSSQESGSGTSPSPGVFLPFSSTLHP-STNNMSTAA-LSQFPPGFNLYPLFPAAMGEFPQGAASQATPDH-PKQ-LDPNMSIKTEYMSFPPPLQRSPLNTTTGRGYVH--HLCLTGHSPSDWLKASYTDNSVQHVRSKTEPQEP----PSSS-ACASR----------HQRPQEFDRASQDSFSSMPDPVDPATITKTFKAGRKASAQANLASRSKAPTS-KSRRRRSKGHNKSA---DGHESDSVSSTADFVLK-----RAAVPHQKDQNKSLLDKLTREKLDSKTKLGSKRNDISS----------------------------DASSDFSLFEALRETIYSEVATLISQNESRPHFLIEVFHELQLLNTDYLRQRALYSLQD---IVTRHLIEKS-----AAEDQLP--PLGPAVR-DAGSQSELTPSESLATSDAEVV-----EKNLRLTQGTTMMMMMMKKREDAESVGNDSSMSTSSNL---------EPFANDDL--GNTVIHLDKA-----LARIREYERMKLKAEFNPCNAGSAAAGGSEVSFAEQPSA----NPADPMQG--------GAAAYV--------HCPQIDTHQLDR-----QIKAIMTKVIPFLKENMDEVCSLQLLTS---VRRMVLTLTQQ--------NDESK---EFVRFFHRQLGGML-QDSLSKFMGRTLKDCGEDLLVEISEI-LFN-ELAFFRLMQDLDNTNSIALAAKNKNKKS-EQPSSSTHGPTVN-TGVGGDKSTSPAYTDEDK----DQDEAEQ--EDV----LQEQTAMKNSRSSEASEVQEEDKPEEDGQGIPLAISLSKAE-TQSLTNYGSGEDENDEEEEMEFEAGPVDVQTSLQPVSADRPVEQEVGLIVSQRYYFAKQTRHTATPRRPKAKRGAQIMAVRLSDISSVEVRLSD-ISKFHQQVVSVCSTAED------RDATQSPEEDRNVGAGAAS-DGSSHD-QDVLKEPTTTSSPDTDSPVMINVDEIGSGNTSQKSDEEDFVKVEDLPLQ--LTV-MCEEELQMRIVEEQQNNNLSVEILNGNTESL---TGLVGDAEDLKEPDTVG-AQNV--------------------  
MATGGP---------------------PFDDG--EEDLHNWTVTSSSLDDRLNNMDWGVQQKKANRSSDKNKK-------KLSAAVVESRLTNDISPESTPGAGRRRA---------------HTPHSFPHIKYTTQMSVPDQAELEKLRQRINFTDMDERSIGSDSQGRVTAANNQRQFAGEAKKPHNFLPLHVNTNKSKELLHPSASAPTTPVISKEVKKQKATRLG---------------SGDKERRLPSQREYGGEDVKLDSSQVVSKLVQIRDYIGKLSSVRDEMVEKNDVP----------------------------------AKVERLSSFIEHLREKEKSCLRLLQKMLAQEV-----------------------------------------DDCD---MGTLESAVGSGSLAESASVNIEVLSSEVSNAA-------------------------------------------------------------------------------------------VKAASADQREELENLRKQHELLKKMLEQQEQLRALQGRQEALMAMQ-----------HSTEQALALTEDNVVTETTGSISGLSITS-----------------ELNEELNELIQRFHNQLHDSQTKAVPDNRRQAQSLSLSREVCWSRSPLAVGAPQHRPLLHSASGPHAGLDSGASAASAKLTKLQELQDKKQTMDKILRELHSLR--DQTLNNNSC--RG----SSAQHSPG-------AQGSS-ECPSALFSNGALASTSFQ--PSFSQQQDGS------NSADKLRKLKEVHKRLNELRELVQYYEQTSDMMVDAVNENVKDAD----EEEDETEEGSIFEATFDSEQENQEPVTNIRN---SQRSRNWTDLNSLANRCSIR-AATNNQDSR-LNTNCEINNRTAANRHALNIPAAIECQYNR-----DIPYSEVRDDDGEEECACNDGAAR----AVVPDSVSE-SSRRSSLGN----DAGFPQKVHR-QSAKQKLRQLQELVAMVQVKDEI---------DDTDGT-------------TANEDEAMHQQPNNTRAT----FGSGSKQSPRDLAFSSKA---------------REKLYEEKLRQQKEELKQLHEERQRLIEIQNKIQDLQWA---CPDLQSSVSSTASQQG-LLRKAPVM-VSTPAAVKVT-SSGQKTNLAVLKPAAPEAPAASATDDGLWSEMRRRQIMREDLRQRRKHLESLMAEHQRRNGFAD------SSCPIEDHEENAT--PSLSVSRDERTMATW-GSSPCHLDNNEEDDDDGHDAGETDEYHSELG-------AEEEDEEEEVE---ECTESSSDDDLPLFSP-NRNQGSYTNKENQGSDVQL----------HNKTKTKPQTRSFNQSSSQHGG-VRRQENLRWASELSVAEGTS---QWKEQVNH--LQRQLDFSSGMCQTLLQDQQTLSFMLQ------SLLTGQYSVLPN-----------------------NRSSPQVQLVMHQLNQCYTQLAWQQNNIQRLKQVLNDLLGQQLPPSSSAAAWQTQKRGSAQESSCGPSASPGVSLPFSSTLHP-SANNMPAPP-MSPFPSTFTLFPPFPSTLGGFLQEPFGPTTPEH-QKQRPDPNTSFKTEYMSFPPPLQRSPLNTAKKTRSVF--PRAPVCY----LMTAGIYCLLLFCSELLDQQIESTPPTRTTSGSSSHL----------KRLAQEFDK---ESFSSVPDVDDPNTITKAFKAGRKASAQANLASRSRTPKNRHSNVKKSKFPPNSS---AGHDSDSASSTTDFGQQ-----RAASSHHRDQNQMLLDKLTQEKLDSKTKLGRKRNDISS----------------------------DASSDFSLFEALRETIYSEVATLISQNESRPHFLIELFHELQLLNTDYLRQRALYSLQVNGVCLLRMYSFNS-----TAVQRHF--IGTQITN-FFETIYVRLSKNNIFFASQEVV-----EKNLRHARDTK------KRKEDADSVD-DSNMSTSSNL---------EAFANDDL--GNTVIHLDKA-----LARIREYERMKLKAEFNPCNA--SAAG---TSDAKHPPS----TAAESTEGIHPHDAPLSAAGSV--------HCPQIDTQQLDR-----QIKAIMTEVIPFLKENMDEVCSLQLLTS---VRRMVLKLTQQ--------NDESK---EFVHFFHRQLGGIL-QDSLSKFVGCTLKDCGEDLLVEISEI-LFN-ELAFFKLMQDLENS----NSSKKATKQKRRVEGPSKPKRTPEVLGST-EKSLCSAKTDEDK----DQDETLMERSCG----APELYLQTK---SNRSNDASENEDEEQGLEVLLSFSLSKAE-IQALTSYGSGEDENEEEETEEFEAGPVEVQTSLQ-VSADGQVEQGAG---SGEIQLENPKEESLENDGGEVYSNKSIIS-----MFSFQLLMIPFLLEANGLAGAVSSPLEE------NIGEESQIPAEEEKAAAAQRDSAPSPPQNVPRESTSTSSPDTDSPVLINVDEAGSGNTSHKSDEEDFVKVDDLPLQ--LTV-MTEEELQKRIVEEQQNNNLSMEILSDHTELL---TELVGNAQTLKQPGLSL-SLLL--------------------  
MATGGA---------------------PFDDG--TEELHSWTAPSCSLEDRLNNTDWGVQKKKANRSSEKNRK-------KLSASVVESRLTNDISPESTPGAGRRRA---------------RTPHSFPHVKYTTQMSVPDQAELDKLRQRINFTDMDERSIGSDSQGRATAANNQRQLAGENKKPYNFLPLHVNTNKSRDLLGPSSSAPATPAISQEAKRPSPGLRDSSTPLVPMKDAPWPSRGGNDRGAGVKREPGRGDQRIDSSQVVSKLVQIREYISKASSMRDDLVEKNDVP----------------------------------ANVERLSHLIDHLKQQEKSYLRFLQKMLTRDN-----------------------------------------EDDD---VGTLDSAVGSGSLADSTSLNTEARSSEASNALGS----------------------------------------------------------------------------------------WPEMVQADQMEELENLRKQHELLKKMLEQQQQLRALQGRQEALMAMQ-----------ESAEQAISAIEDTVVTDTTGSVSGLSITS-----------------ELNEELNDLIQRFHNQLHDSRTKAVPDNRRQAESLSLSREVCWARSPQAVGPPQHRPLLHSASGPHTDLDPEATAASAKLTKLQELQDKKQTMDKILQELHSLR--DQTLNNSSC--RG----FATQCSLN-------LGGSS-DCS-ALCTNGASASTAFH--PALAQHQDGS------SSKDKLRKLKEVHKRLNELRELVQYYEQTSDMMVDAVNENVKDDDDDEEEEEEETEDGSMFETVFQSEQDNRHSVTNIRN---SQRGGNWVDLNSLTNGHGVRGSASDNQDGR-LNTDCEINNRSTANLRSPNIPSTIACQYNR-----DTPYNEDPDED--EDVLDNEEGAR----AAG--PDSEGSSRRSSLGN----AGEFVHKVHQ-QKVKQKLRQLQELMAMVQS-------------DDTDGT-------------TANEDDVLHQQPNNTR--------AGSLPSPPELSLSSKA---------------REKLYEEKIRQQKQELKQLHDEHKRLTEIQGKIRDLQWA---CPDLQASVCSAGSQQG-LLRKVPVTPVSIPGPLQTAVSSGPK-NGTILRKTAPEPAVPPAADSELWSEMRRHQMLREELRQRRKHLEYLIAEHQRRSGLTD------SPQ--HNRESLAS--PSHTLSRDERTLATW-GCN-SMPTG------E-----EDDEYNSEMM-------LPAKEERRSRM---TLTRSSSDDDIQIYTP-SRNQNSYSNRKNQGSNQNPP-LVSEDGGGHTPLRNKTKAQEPQQSANQGGG-ARRQENVRWATELSFAEGSR---HWQEQVSQ--LQRQLDFSTSMCQTLLQDQQTLSYMLQ------TLLTGQYSVLPN-----------------------NLSSPQVHLVMHQLNQCYTQLAWQQNNVQRLKQVMNELLCQQQQSSPAPAGWQTQKQSSFQESGCPPSA--GVAPTFSSLLNP-LANNMAASP-MPSFSHSLNSLSLFPAPMGEFAQGAAGQASPVQ-QKQQLDPNTSVKTEYMSFPPPLQRSPLNTTAERG--------TTG-----WQKTSNANNIVHCYQSKTQRQNS----RSTSPAFSPE----------RSPPQQFDRVSQGSYSSLADPVDPTTITKTYRAGRKASAQANLAARSKTPN--KARRRLNKGRNKNN---EGPVSDSVGSTADFVQR-----RAPLPRQKDQNKSLLDKLTQEKLDSKSKFGKKRNDLSSAYAWRTPFLS---------NRIACTEAPDASSDFSLFETLRETIYSEVATLISQNESRPHFLIELFHELQLLNTDYLRQRALFSLQD---IVTRHLTEKN-----AADDPSP--PRSPVAW-AADSQSELTPSESLASSDAEVV-----EKNLK------------RRRVGAESV--DSSVSASSNL---------EPFANDEL--GNPVLHLDKA-----LARVRKYERVKLESELSPRSA--SPAGVSELSNAEHPS-------ADKTEE--------GASAEV--------RCPQIDTQQLDC-----LIKAIIAEVISFFKENMHKVCSLQLLTL---VRRLVLNLIQQ--------HDESR---EFVQFFHKQLGGIL-QDTLSKFEGRTLKDCGEDLLVEISEI-LFN-ELAFFRLMQDLDSK---SV-AKHKSKQPGDLGGPTRPGAKVN-AAAAGGKSVSPAYTEEDKPFTQEQDEAQE-VGRT----LQESHSQTERKSS-KSEGSGEDEDEGRGENSPLLISLPKAE-TQALTNYDSREVDNEEEETEESEARPADVQT-----SADGQLEPEP-----------SDSGTTGGDAG----------------EETTEERSSE-NEETNESIEMMDATVVE------CVTARCQSP--EAEAAAPA-DGAPAG-SEGQQVTTPTSSPDTNSPVMVSVEELGSGSNGQRSDEEDFVKVDDLPLQ--LTV-MCEEELQKRIVEEQQNNNLSMEILNGNAETV---TGLVGNAQALKEPDTAG-AENA--------------------  
MASGGA---------------------PFDDG--AEELHNWTAPNCSLEDRLNNMDWGVQQKKANRSSEKNRK-------KLSAPMVESRLTNDISPESTPGAGRRRA---------------RTPHSFPHVKYTTQMSVPDQAELDKLRQRINFTDMDERSIGSDSQGRVTAANNQRQIAAENKKTHNFLPLHVNTNKSRDLLGPSSSAPATPAISQEMKKQGPGLRDALTPLVPMKDTPRPSRGGNGRGAGLKGELGR-D-QIDSSQVVSKLVQIREYISKASSMRDDLVEKNDVP----------------------------------ANVERLSHLINHLKQQEKSYLRFLQKMLTREN-----------------------------------------EDDD---VGTLDSAVGSGSLADSTSLNTEVRSSDASNAVGS----------------------------------------------------------------------------------------WPEMVQAEQMEELENLRKQHELLKKMLEQQHQLRALQGRQEALMAMQ-----------ESAEQAIAAIEDTVVTETTGSVSGLSITS-----------------ELNEELNDLIQRFHNQLHDSRTQAVPDNRRQAESLSLSREVCWSRAPQAIGPPQHRPLLHSASGPHTDLDPEATAASAKLTKLQELQDKKQTMDKILQELHSLR--DQTLNNSSC--RG----FSTQCSLN-------MGESS-DCP-ALCSNEASSSTSFH--PAPTQHQDGS------SSTDKLRKLKEVHKRLNELRELVQYYEQTSDMMVDAVNENVKDDDDEEEEEEDETDDGSMFETMFESEQDNRHSVTNIRN---PQRGGNWVDLNSLTNSHTVRGGNSNNQDGR-LNTECEINNRSAANLRSLNIPSTIGCQYNR-----DTRYNEVKDED--EDVLDNEEGAR----AAAA-PDSEGSSRRSSLGN----AGEFVHKVHQ-QKVKQKLRQLQELMAMVQS-------------DDTDGT-------------TANEDDILHQQPNNTR--------AGPKPSPREPGLSSKA---------------REKLYEEKIRQQKQELKQLHDEHKRLTEIQGKIRDLQWA---CPDLQSSVCSTGSQQG-LLRKVPVTPVSIPGPVQTAVSSGPKPNVSLLKKAAPEPAVAPVADNELWSEMRRHQILREELRHRRKHLEYLIAEHQRRSGPTD------SPR--HNRGSLAS--PSHTVSRDERTMATW-GSSPCQLED------D-----EDEEYNTEMG-------ADEEEEQDE------CTEGSSDDDIQICS---RNQSSYSNRKNQGSNRNPPILISGDGGGNTPLHNKTKAQEPQRSENRHGS-ARRQENVRWASELSFAEGSR---HWQEQVSQ--LQRQMDFSTSMCQTLLQDQQTLSYMLQ------TLLTGQYSILPN-----------------------NLSSPQVHLIMHQLNQCYTQLAWQQNNIQRLKQVMNELLCQQQQSSSAPSGWQTQKQSSFQESNCGPSAPPGVVPIFS-VPNP-SANNMAASP-MPSFSHSLNAFSLFPAPMGDFAQSAACQASPDQ-QKQ-SDPNASMKTEYMSFPPPLQRSPLNTTAERG--------STG-----WQKTSNENNIVHCYQSKSERRDS----RSTSPAFSPD----------RSQLQRFDRGSQESYSSLADPADPTTITKTYKAGRKASAQANLASRSKTPN--KARRRLNKGRNKNN---EG-LSDSVGSTVDFGHK-----RAPLLRLKDQNKSLLDKLTQEKLDSKSKFGKKRNDLSSAYAWRTPFLS---------NRIACTEAPDASSDFSLFEALRETIYSEVATLISQNEARPHFLIELFHELQLLNTDYLRQRALFSLQD---IVTRHLTEKN-----AAEDHLP--SLSPVAW-AADSQSELTPSESLASSDAEVV-----EKNLK------------KKRGGAESV--DSSASASSHL---------EPFA-DEL--GNTVLHLDKA-----LARIGECEHTKLKAEFYPRSA--SAAGAGDVSNAAHPSA----DCADRTEE--------GASADV--------RCPQVDTQQLDR-----QIKAIIAEVIPFFKENMDKVCSLQLLTS---VRRLVLNLTQQ--------HDESR---EFVQFFHKQLGGIL-QDTLTKFEGQTLKDCGEDLLVEISEI-LFN-ELAFFRLMQDLDNS---SHGAKHKGKKPEERPSKTRQAGKPNAAAAAGGRSVSPACADEDDSFIQDQDEATRGANAT----LQEFYYQSEKKSS-RSEGS-EEEAEGQGERLPLSIDLSKAE-TQALTNYGSGEDENEEEEMEEFEAGPVDVQTSLQ-ASADGQPEPEP-----------EQEGPTGDETG----------------EVTTEQRSSD-NDETNESVEMMDSTVVE------CNVARSPSPQAEAEAAAPS-DGASAG-CEGQQVT-PTSSPNTNSPVMISVEEIGSGSTSQKSDEEDFVKVEDLPLQ--LTV-MCEEELQKRIVEEQQNNNLSMEILNGNAESV---TGLVGNAQALKEPDTVG-AQSA--------------------  
MATGGT---------------------PFEDGTDEQEMQNWTISNGSLDDRLNNMDWGVQQKKANRSSEKNRK--------KFSAMSESRLTNDISPESTPGAGRRRA---------------RTPHSFPHVKYSTQMSVPDQAELDRLRQVINFTDLDERSIGSDSQGRVTAANNQRQLSTEAKKPFNFLPIHLNTNKSKE---PTASTSSTPG-GKEHKKQSPG-KELFAPVPAVVKEPFSLDG----AQRLGLEDGRGELSIDSSQIVSKLVQIREYIGKATSMRDDLVEKNDVP----------------------------------ANVERLSLLITHLKEQEKSYLRFLQKMLAREN-----------------------------------------EDDDDDEGATVDSAVGSGSVAESTSLNLEPRSETSSATG--------------------------------------------------------------------------------------------HGVCREQKEELENMRKQHDLLKKMLEQQEQLRALQGRQAALLAMQ-----------QSAELAIAVMDDTVVTETTGSVSGRSITS-----------------ELNDELNDLIQRFHNQLHDSQSQVVPDNRRQAECLSLSREVCRSRTTHN-----SRGQLPSS----APLTTASTSASAKLSKLQELQDKKQTMDKILQELHSLR--DQTLNNSSCKPPF----LTQRMHS--VSQRSARVGMS-ERPSVLGREGNGPRPVRQGSSTYTQIDDNS------HSADKLRKLKEVHKRLNELRELVQYYEQTSDMVVDTVNENVK-------EEDEETEDGSLFEAMFDSEQENHEPVTNIRNPAQPQTPSNWMDMNSLTK---TR-SASNNRDGR-LNTECEINNRSAANLRSLNIPSVIECQHNR-----DRPYVGVKDED-DEEVLEDDDGARGGGGGGGRDSDGSGSSHRSSLGG----DAEYAQKVHRLQTAKQKLRQLQELVAMVQS-------------DDTDGT-------------TANEDEGLKQQPNNTR--------ATAPKAQRDIALCDKA---------------RERLYEEKLKQQQHELKQLHEERQRLLEIQGKIQDIQWA---CPDLQSSVSSSASGQ--MSRKIPAA-ASTPAPVPLQSSSSAKANTSGLKPTT-EPPPVTVTDNELWTEMRRHQMLREELRQRRKQLESLMAEQQRRSTLPD------SPFRSDTHE-------TQSYSRDERTVATWGGSTQCPLGDE-----------DDD-YSSEVG-------AEEDDDDDERD---EGEESSSTDERHACS--TQKQRNYNRNNREG-----EVNTS-----PSPRRSRGRSRQQPQSERGSSSKAKRQENLRWAADLSLAEGAAPA-HWQEQITH--LQKQLNFSTTMCQTLLQDQQTLSYMLQ------ALLTGPCGVVPN-----------------------NVTSPQVPLIMHQLNQCYTQLAWQQNNVNRLKQTLNDLLRQQQSQSSSG---QQQHQSVAHDP-----ASPSMFLNYPSVNAL-NIPGLPN---FSPFPTGFNFAPMFPPSVGDFQQ---NQASSD----QHHDPNISLKTEYMSFPPPLQRSPLNNAEKRY-------------NIYFLKN--NRSSQRPILTSDHPSQVEQNSLSSSPVPHPS----------TSRDRSYIPDSQESMSSLPDRADPTTVTKTFRAGRKAAAQASLASRDKTPN-MKSRRKRGRGQKNTASISASVDSDSVSSDSHFERE-----RSNQVKYKELNQGLLDKLTQEKLDSKTK-NSKPNDLSS----------------------------DVSSDFSLFEALRETIYSEVATLISQNESRPHFLIELFHELQLLNTDYLRQRALYSLQDYQDIVTRHLTEKS-----VADEQAS--SLGPAVW-ATGSQSELTPSESLATSDNDAS-----DKIVTVKSNS-----VLKRVPDRDSMDNESLLSTSSNL---------EPFASDDL--GNTVIHLDKA-----LARMREYERMKLRAECNTDNP--EHSSAIAAATVASPQ----------------GAGHLS--TDA--------HCPQIDTQQLDR-----QIKAIMTEVIPFLKEHVGEVCSQKLLKA---VRHMVLNLTQQ--------NDESK---EFVRFFHRQLEGIL-QDSLSKFVGRKLQDCGEDLLVEISEI-LFN-ELAFFRLMQDLDQN---TSKNKLRTKRRSDHTSPKHSPHTEEMKTLEREKTFSPSYFDEDR----DQDETER----------QEMPHEREEEKEKEAQSSEASEVEEE-EGLPLSISLSKAE-TKALCNYGSGEDENEVEEMEEFEAGPVEVQTSLQ-ASMDNTSEHREVLLQCTSSALQNETPQETKSDQ-----------------DSSE--SSQLNSEPSDPKVIVQPPEEG------GEEEKSASGDEEEEK----EDSTSAPVQETPQSS-DSASPDTDSPVMINTDEAGSGNTSQRSDEEDFVKVEDLPIQ--MSV-LCEEELCKRISEEQQNNNLTAEILNGNTSEL---TGLVGNAQTLKEPVSCF-LRNA--------------------  
MATGGT---------------------PFEDGTDEQEMQNWTISNGSLDDRLNNMDWGVQQKKANRSSEKNRK--------KFSAMSESRLTNDISPESTPGAGRRRA---------------RTPHSFPHVKYSTQMSVPDQAELDRLRQVINFTDLDERSIGSDSQGRVTAANNQRQLSTEAKKPFNFLPIHLNTNKSKE---PTASTSSTPG-GKEHKKQSPG-KELFAPVPAVVKEPFSLDG----AQRLGLEDARGELSIDSSQIVSKLVQIREYIGKATSMRDDLVEKNDVP----------------------------------ANVERLSLLITHLKEQEKSYLRFLQKMLVNNN-------------------------------------------------------------------------------------------------------------------------------------------------------------------------------SIEQKEELENMRKQHDLLKKMLEQQEQLRALQGRQAALLAMQ-----------QSAELAIAVMDDTVVTETTGSVSGRSITS-----------------ELNDELNDLIQRFHNQLHDSQSQVVPDNRRQAECLSLSREVCRSRTTHN-----SRGQLPSS----APLTTASTSDSAKLSKLQELQDKKQTMDKILQELHSLR--DQTLNNSSCKPPF----LTQHTHLKGLLQRSARVGMS-ERPSVLGREGNGPRPVRQGSSSYTQIDDNS------HSADKLRKLKEVHKRLNELRELVQYYEQTSDMVVDTVNENVK-------EEDEETEDGSLFEAMFDSEQENHEPVTNIRNPAQPQTPSNWMDMNSLTK---TR-SASNNRDGR-LNTECEINNRSAANLRSLNIPSVIECQHNR-----DRPYVGVKDED-DEEVLEDDDGARGGGGG--RDSDGSGSSHRSSLGG----DAEYAQKVHRLQTAKQKLRQLQELVAMVQS-------------DDTDGT-------------TANEDEGLKQQPNNTR--------ATAPKAQRDIALCDKA---------------RERLYEEKLKQQQHELKQLHEERQRLLEIQGKIQDIQWA---CPDLQSSVSSSASGQ--MSRKIPAA-ASTPAPVPLQSSSSAKANTSGLKPTT-EPPPVTVTDNELWTEMRRHQMLREELRQRRKQLESLMAEQQRRSTLPD------SPFRSDTHE-------TQSYSRDERTVATWGGSTQCPLGDD-----------DDDDYSSEVG-------AEEDDDD-ERD---EGEESSSTDERHACS--TQKQRNYNRNNREGLKVLSEVNTS-----PSPRGSRGRSRQQPQSERGSSSKAKRQENLRWAADLSLAEGAAPA-HWQEQITH--LQKQLNFSTTMCQTLLQDQQTLSYMLQ------ALLTGPCGVVPN-----------------------NVTSPQVPLIMHQLNQCYTQLAWQQNNVNRLKQTLNDLLRQQQSQSSSG---QQQHQSVAHDP-----ASPSMFLNYPSVNAL-NIPGLPN---FSPFPT-----------VGDFQQ---NQASSD----QHHDPNISLKTEYMSFPPPLQRSPLNNAEKRFQSH--SDNLTDANNSSWLNSSLNRSSQRPILTSDHPSQGEQNSLSSSPVPHPS----------TSRDHSYIPDSQESMSSLPDRADPTTVTKTFRAGRKAAAQASLASRDKTPN-MKSRRKRGRGQKNTA----SVDSDSVSSDSHFERE-----RSNQVKYKELNQDLLDKLTQEKLDSKTK-NSKPNDLSSAYAWRTPFLS---------NRIACTEVPDVSSDFSLFEALRETIYSEVATLISQNESRPHFLIELFHELQLLNTDYLRQRALYSLQD---IVTRHLTEKS-----VADEQAS--SLGPAVW-ATGSQSELTPSESLATSDNDAS-----DKIVTVKSNS-----VLKRVPDRDSMDNESLLSTSSNL---------EPFASDDL--GNTVIHLDKA-----LARMREYERMKLRAECNTDNP--EHSSAIAAATVASPQE----SPVLKVISLITNQKHVMQIMNA--------HCPQIDTQQLDR-----QIKAIMTEVIPFLKEHVGEVCSQKLLKA---VRHMVLNLTQQ--------NDESK---EFVRFFHRQLEGIL-QDSLSKFVGRKLQDCGEDLLVEISEI-LFN-ELAFFRLMQDLDQN---TSKNKLRTKRRSDHTSPKHSPHTEEMKTLEREKTFSPSYFDEDR----DQDETER----------QEMPHEREEEKEKEAQSSEASEVEEE-EGLPLSISLSKAE-TQALCNYGSGEDENEVEEMEEFEAGPVEVQTSLQ-ASMDNTCEHRE------SSALQNETPQETKSDQ-----------------DSSE--SSQLNSEPSDPKVIVQPPEEG------GEEEKSASGDEGEEK----EDSTSAPVQETPQSS-DSASPDTDSPVMINTDEAGSGNTSQRSDEEDFVKVEDLPIQ--MSV-LCEEELCKRISEEQQNNNLTAEILNGNTSEL---TGLVGNAQTLKEPETIG-AQSA--------------------  
---MAGK-SRQLISQSFTEGPSVPDRRPARHNLSDSDMLSL--SAAMMDSRPNNLFSMGAIPRQSKSVNQPSM-------------NNDRRMMGRYPGGPP----------------------LVDSLYRQAGLGPGVSPRSASAPLTMDNQLNVGARMTVGANNAVANNERSATTQVK---PSHQGARVNQLSLPTRAPATATAAAVRREEASSVE--------------------------------------------SLEPDTSHIIGRLMQIRDYLKQAKGMLSQIENSTGTSW-------------------------------NREQRDKLQELVGHLEEQEKGYLGLLQRILAGDR--EGEAAGQ--ADDDTS---------VNG------------SVAYSMSLDLGDARSDISEVTSEATYTARNRPQIEDHLGFH-----------------------------------------------------------------------SSSEMEQDSNGNFGSWDNDGRPVSPGPGARR--DHAARLLAWGMPPEEPERAVSPEPGARRPPR------LAWATPDPEELNNQQDAARAADTTEKASGPEE-------------------EDLEGLRQQKELLRKMLEQQEQLRQLQDRQRM-LVQLQEEVQDTLQSSQ--------AAQGEEGAQGGVIPEAVAD-RLKDKLQVLQGKQEHMADLMRQLHSLQ--HLRQMHEL-----------------------DPTSSSVTSNNRQNESIEEAMKRRESGQQEPAARAPAVSAPRQDTENKLRKLQDVRERLNQLRELVRSYQAS----GFDDETITI-----------------------STTTSQQRDSDYLEN---GVEQDDDDDDDDDEE--------DDDDDDDEEDEDDDSDDEEDDKEEE-EEEGDMGADGAR--------------------------------------GQEDNVSLSSSTLATMAEDPVIKEKVRKLQEAKEKLRRLQDLVSVMQTSSMPGITLPKELADLLG---------------ETEEEDAEEPPPHQAR-APPSLPDS-----TASSQPGQSM---DDQV--------------RRMQEE--ELASLLQEKERLMAIQQELLRLQDAKLTMATAHQQEARTDSAPSSARAYRDRSTGEAPQPQPRVQYQGTSASQRQLPPRQ------RPTDRDTWEELRQQMMLREEIRRKRTELEDLT--SAAEK--------GDFSVISDTSGFP----GYSVMSGGDVTMATWGGSTQRSSDSVQD------QGMDFNSMDEGYPSDGIVQAEEEEEED--GS---HATYTIERG-----------PGGNYLRLSPR---ADQDLGRIYDAPSGSRG----YSSSRDSWRNRQENYRSAEELMQEDVSGWVVKE--CRRLQDLCGH--LQQQMGSTTNLCQALLADQQLLNRLVS------TSLNGSSGYGSGSASPQTPRT--------DLLAYFSGQLQQKQQLLAQLLQCYNQLSQQQHDLVVMQQTWEGAHSLQDPNH----------PAVTSPT-----PTSAPFRPDSNPF------------SHPQLSPFASYPSQPRGS---FAFSPSFPANFPN---PDT-------TQSYRLPMPGSGYRGLHPTSEGMQQTDITIPPEKLRRQSDVPARPPAAAPVSSGKG-------------------------------------RTAQAAAPTSVPKLDFTQLKKARE----------------SSRMQGTPDSKTKKSKKRGHRGTMYEAEGDQLYQSF-----PHVTAGISGTAFQDTTSISSKMSSVTGSERPD-------------------------------ADVLSSHAASEVSLFEALRDTIYSEVATLISQNESRPHFLIELFRELQMLTSDYLRRRALYALQD---LVTRYLTEDS----IKQHRPPISMPVNLRAW-LG-GNSEQTPSDSMVTSDNE-------GGEEGRTMPG--------QYDYMEDAGSASSMSTPQSEGG-----FDP-FANDDL--GNTVIHLDRA-----MHRMREYERLKAEAEGGDDAS--PHSSEPGQANTTSAATSTNSATSNSSAQDVGSESSISDGP-----------YPRVDTQQLDQ-----EIKAIMTEVIPHLKEHMEEVCSPQLLAY---IRRLVMSLTRH-----------RDDSKEFSRFFHKQLGTIL-QDSLQKFVGRKMRDCGEDLLVDISEI-LFN-ELAFFRLMQDLDSS---PSKNWLQPTQSGEEDQTTGSEAAAGGEGEAKS-DSSSSTETGDE------DGDTTPKGTASHTTGTQAGWHRDKASSGVSTQGTGQGSRMYPEVPPVKFDLSVSE-LQPLTSYGSGEEDEEQETEQFEQVTDIPTGVQAERDVSDANTAPSANQPEQTASPQKAGSPAK---------------------------------PDQPGSPVRPAQGEESVVTGAEPDQEVSFKPKSADVNEDGTAEAAGEGQNGEVAVPEQGEDKSEDKEEPIAENGVPDGSEEAPAQVGQGDQVGEVSPR--SLTALSKEVLEQQQAEEDKEN---VLVVEMIADGTM-NPQLAGDPNKLPEVESSA-------------------------  
---MAGK-SRQLISQSFSEGPSVPDRRPARHNLSDSDMLSL--SAAMMDSRPNNLFSMGAIPRQSKSVNQPSM-------------NNDRRMMGRYPGGPP----------------------LVDSLYRQAGLGPGVSPRSASAPLTMDNQLNVGARMTVGANNAVANNERSATTQVK---PSHQGARVNQLSLPTRAPATATAAAVRREEASSVE--------------------------------------------SLEPDTSHIIGRLMQIRDYLKQAKGMLSQIENSTGTSW-------------------------------NREQRDKLQELVGHLEEQEKGYLGLLQRILAGDR--EGEAVGQ--ADDDTS---------VNG------------SVAYSMSLDLGDARSDISEVTSEATYTARNRPQIEDHLGFH-----------------------------------------------------------------------SSSEMEQDSNGNFGSWDNDGRPVSPGLGARR--DHAARLLAWGMPPEEPERAVSPEPGARRPPR------LAWATPDPEELNNQQDAARAADTTEKASGPDE-------------------EDLEGLRQQKELLRKMLEQQEQLRQLQDRQRM-LVQLQEEVQDTLQSSQ--------AAQGEEGAQGGVIPEAVAD-RLKDKLQVLQGKQEHMADLMRQLHSLQ--HLRQMHEL-----------------------DPTSSSVTSNNRQNESIEEAMKRRESGQQEPAARAPAVSAPRQDTENKLRKLQDVRERLNQLRELVRSYQAS----GFDDETITI-----------------------STTTSQQRDSDYLEN---GVEQDDDDDDEDDDD--------DDDDEEDEEDDDDDSDDEEDDKEEE-EEEGDMGDDGAR--------------------------------------GQEDNVSLSSSTLATMAEDPVIKEKVRKLQEAKEKLRRLQDLVSVMQTSSMPGITLPKELADLLG---------------ETEEEDAEEPPPRQAR-APPSLPDS-----TASSQPGQSM---DDQV--------------RRMQEE--ELASLLQEKERLMAIQQELLRLQDAKLTMATAHQQEARTDSAPSSARAYRDRSTGDAPQPQPRVQYQGASATQRQLPPRQ------RPTDRDTWEELRQQMMLREEIRRKRTELEDLT--SAAEK--------GDFSVISDTSGFP----GYSVMSGGDVTMATWGGSTQRSSDSVQD------QGMDFNSMDEGYPSDGIVQAEEEEEED--GS---HATYTIERG-----------PGGNYLRLSPR---ADQDLGRIYDAPSGSRG----YSSSRDSWRNRQENYRSAEELMQEDVSGWVVKE--CRRLQDLCGH--LQQQMGSTTNLCQALLADQQLLNRLVS------SSLNGSSGYGSGSASPQTPRT--------DLLAYFSGQLQQKQQLLAQLLQCYNQLSQQQHDLVVMQQTWEGAHSLQDPNH----------PAVTSPT-----PTSAPFRPDSNPF------------SHPQLSPFASYPSQPRGS---FAFSPSFPASFPN---PDT-------TQSYRLPMPGSGYRGLHPTSEGMQQTDITIPPEKLRRQSDIPARPPAAAPVSSGKG-------------------------------------RAPQAAAPTSVPKLDFTQLKKARE----------------SSRMQGTPASKTKKSKKRGHRGTMYEAEGDQLYQSF-----PHVTAGISGTAFQDTTSISSKMSSVTGSERPD-------------------------------ADVLSSHAASEVSLFEALRDTIYSEVATLISQNESRPHFLIELFRELQMLTSDYLRQRALYALQD---LVTRYLTEDS----IKQHRPPISMPVNLRAW-LG-GNSEQTPSDSMVTSDNE-------GGEEGRTMPG--------QYDYMEDAGSASSMSTPQSEGG-----FDP-FANDDL--GNTVIHLDRA-----MHRMREYERLKAEAEGGDDAS--PHSSEPGQANTTSAATSTNSATSNSSAQDVGSESSISDGPVYNYNVVYPRQYPRVDTQQLDQ-----EIKAIMTEVIPHLKEHMEEVCSPQLLAY---IRRLVMSLTRH-----------RDDSKEFSRFFHKQLGTIL-QDSLQKFVGRKMRDCGEDLLVDISEI-LFN-ELAFFRLMQDLDSS---PSKNWLQPTQSGEEDQTTGSEAAAGGEGEAKS-DSSSSTETGDE------DGDTTPKGTASHTTGTQAGWHRDKASSGVSTQGTGQGS-----------------------SYGSGEEDEEQETEQFEQVTDIPTGVQAERDVSDANTVPSANQPEQTGSPQKTGSPAK---------------------------------PDQSGSPVRHAQGEESVVTGAEPDQEVSFKPKSADVNEDGTAETAGEGQNGEVAVPEQGEDKSEDKEEPIAENGVPDGSEETP-EVGQGDQVGEVSPR--SLTALSKEVLEQQQAEEDKEN---VLVVEMIADGTM-NPQLAGDPNKLPEVESSA-------------------------  
MAA--SK-APQLHRSVMSE-PRGNAVPRYNFTQPMDDFLFNLIGGEKEESKPNNY---YNWKPSSTFAEQPSTVVRSKKSKNKEREQNISVKADTQGAAMMSNGPSRR---------------RTPATYPRTQHVS--SLLDREELENLRQQLTYSDVDDQ-STDSQALGSVSIANRVN---NMRLGDDVASIESGTRRPPSLLDTIFVNE----------------------------------------------------KPDRNKIVARLMQIRDYVKQANAMMSTLQNSIESPE-------------------------------KDDQVGKLGRLQEHLQEQERGYLALLERMLAEELSLAGSISGM--ESSVTSNDLNGLDAAVEGRSSQNSGESFATSVAESASINL-DARSDISEATTEGTFTARTRPKIESHLGNHSCASDNDISDSELDDIRSVSSYASGSFGLDTQELHEQYSSMLQKYQDGGEENLSAAMERITLSRNEGQDESTSSHMSSNSSYSFQEGQQTKQNVSEQIETLEGLRKQHDLLRKMLKQQEELRTLQNRQTALMTLQTQIESRLTNSEQERAEETGSITSTQATTTTAGTSVTEDTEPLMTTAGRFATGVPLTAEDLQERR--RTLMR-MLAEQSDTR-------------SGEIQQARALDW--------MEQAEEEVSPPLVSSEHA--ELRDKLEALQQKKRQMDDLLIQLHALH--P--DMANM-----------------------VGDTSSVASTTSNANEYSGLMVPCE----EPNESLDENEATLTNGTESIRKLHDVRERLTELRDLVQQYQDTSQVLGDQDEDSQR-----------------------KQQMQQLGLGANYED---PVLMSNLRRLERGEP--------VGRPVQMRPNHMSYGGDSAHVQVGSGQTTTTVTETETE--------------------------------------TETETESNHSSILAAWGDDPEIKEKVRKLQEAKHKLRQLQSLVAMVQQTPEVASALPDDLAELAAGLTAGLTADYSESNNEDDDEDEDEDEEDETEEGDETVEESGSELAVAGATGDHRMPNRDEQIPEEFVRETREAYYEAKMNQQRRELQNLMSERHRLLGVQQQLKELNEN---LPPVRKQPAKPG----------VKTVNIIP---PTKRDASTNVQSVATPSRE-----------EVYSEMRRNRALQDELREKKSELSDLL--HIVKMNRERLRDGSEYPLQTDLAGYDNDLFRLSMRSADITAAATWGGSTQVSED---------------EGLEDGYPSDGILQVEEEEEEQSIDT---SDTYTIESGDKPPSRRRKALPYPGVKRVTME---DDGETSSMDAARHGIRYPKHINSALDRQSHLRQENIRSAEELVSDGYEDPSMDDPTVQALQGHIGQ--LQRQLSNSMGMCQSLISDQQSMSNLLS------GSLNAGHGGLQPTPTPPNPFVPPGEGHLDDLVQNYDLRMQHQQLMLN-LNSAYTQLYQQQNQIQGLRSQLEERHSEASFDVGRGGQGGMASTNTNSNN-----NAPPMYRNQARPMGMGYPGQAPSFGRSPGMGPEAMYGPPPYNRGMPFNVNPTFPPGFAQ---PSPGNYPSPYANPYASNNLNEGGSAGQRDTYNTYARQPPHPQQRYGGNAASYHVPPNTAQVSNDDDEFETAYLNGAISSKQMKMMGGYTLDSGIGDINDLLAREPTESREEIAVPPLDITSLLKRTDQQRRLRDELGSQRSNTSSNRRGKHPMPSSASQARAQLSSQSTQPSARLAKLLGPKMGTGLSSSISGTAFLDTASITSTVSMSSLPGDSDRGNNGAKSKGKGQQKRQQQIQQDTDRSIMSQTSELESDAGSEFSLFEALRESIYSEVASLISQNETRPHFLIELFRELQMLNTDYLRQRALYALQD---LVSKYLTDDNNTNASASEAPTLPSERPMPAW-MNFAGSEQTPSESFITSDEEEIKARLFGANLQKPHESSYALLEDDQYDYIENVDSASTLSTPPSLGR-----AEFGFANEDL--GDTVIHLDKA-----LKRMREYERMKAEVEAASGIS--SIDNTKEGG-----AMSASDMTSNSSAQDMGSESSFSDLP-----------YPRIDTRQLDL-----QIKGIMQEVIPYLKEHMDDVCSPQLLAY---IRRLVLGLARQ-----------KDESREFVRFFNRQLGSIL-EDSLAKFSGRKMRECGEDLLVDISEI-LFN-ELAFFRLMQDLDTA---GESSSDDESSSDEEDTVQQQSHPAGRQQQQQGGDGSGGGGAMME------DELESAMAVASRFEEEELGNTRDDMFSTQVDISDREEDSEPQGVDHLQIELSMSE-SKGVTYIGSGEEEDDPDSLG-EAVEETITSIGAAN--ADG-EIDSVDDTTMS-SEIRVESDAS---------------------------------QKENIS--R----QESLEKDPAPAPAPVPAPESNETNETP------ESQ--EVPEFQETPEASETPEATTPQATVGENNNEEL-------TVDDLPPK---LTVITQNSLDNQMMKEQTNTDGVQAVLETMVEG---EAQLAGDPMALSAPEGAPVENGIIGGTPGEIGSE---------  
MATGRQKVSRGTCSSSEPRRPLLYRPNPTEEDQQLNS-------NELFDDRLNNW---TNWRPSTTFANQLQR-------QKKKKEGNRDRELPLGLESPPTSALLRH---------------HTPATYPRTQFISGVPAAERAALESLKQRLTFSETDEQSVD---MEGAQAAANNER---EVRRRTAAHPLQQQSNILNDVDA-----------------------------------------------------------PDRNQIVARLMQIRDYQKQAGAMLSNLQNSSDRAS-------------------------------NTDQIRKLERLIDHLKDQERGYMNLLQRMLTDDSGIGIMTENQ--QNNSGESN--------------------STSLAESASINM-DARSDISEATTEGEFTMSTRPRIESRLG-----------------------------------------------------------------------ELLPEAEQSHASPRAAGTNELNKYYMEMLNEYRTESPFNETLNETVIRRRTRSVSSGETSSLSSTN-----RSSSSGAESDDEVEQLETSQASTTDFTTTGTTTTD-----------------QSESSLPEPGLDLDAERRRLNLLQLLAEDREM-R---AAELMRAREEDR--------RER-EVHVEDTLQAMASERLELQDKLHSLQDKKQQMDDLLQHLQGLR--A-QQINNL-----------------------DADSAASGSSSQLR--INESIQATASANQTPHDATQELLDAL-DAKEKLEKLREVRDRLNHLRDLVQYYQNGN---EFIHTETNP-----------------------NENYEETSSMADYED---PILMRNLRGLQRSGF--------LSSQARDQNEGFALSGNTRRDPRQD--------TTDDE--------------------------------------DEEDENDNLADEEDDDDDDKDESENVEKSDSENDEYDTESNHSSLLGAWGE-----------------------------DPEIKEKVKEAYYEAK-----------------------------------------------MKQQRGELEKLMEERQRLLSVQFQLQKLNRQ---MPSLSATSTSLGNTP-------RGSKNSNP---SSRKIEDSPRVSLPEGSEV------ESSNRELWSEMRRHKILREELRQKRKELEELLEESRRQRNSADTSDRGTYSIQSDGND------GMG-MSADVTTQATWGGSTTHMSDMDDE---------DEEDVDDGYPSDGIVQVEEEEEQEGSSD---HDTYTIEADYRHRSRRPLTPPDLTFIRNKKRKHEEDDPTNRRPYRPNNLFSSRRAFNTSRMSGSARQENVRSAEELIREDERRRNPTETERQLLLWQCQQ--LQQQLNTSTNLCHTLLRDQQSIIHLLGNNNPTFSSIGGPYTLPTPTGTSPSPFTPTSP-YGGDSLSNYNFQLQQQQLLLN-INHAYNQLYQQQLEIRALQEHFQQLTTRDNHNHN----------NSALDD-----LRYGEYDSDRPSLFAS---------HFQSPHRLTTFNPPYATMNSTFNLSPQFPSTFSH---PTN----YASAHNERLYDSGNRRAAEAAGYPKYERQDASTRYDYLRQFEDANQNPWQNDSIRTFPRRHEAEDDENEEECYE--------------------DDDIVNQLNQPRIPPLNLAEILKRAEKRRATRSQQATDKSTQEPDIIGTKFPSQSKATATRSLAKKDRRQKPKISNPHDFR--PGLSAGISGTAFVDTASLSSAISSVPGGINEDRMAKMAR----------------------KAVSDGESDAQSDFSLFEALRENIYSEVATLISQNESRPHYLIELFRELQMLNTDYLRQRALYSMQD---LVSRYLTEEN----------------------------------------------------------------------------------------------------------------LTRP-----IKSV----------------------------------------------------------------------YPRIDTGQLDK-----QIKGIMQEVIPYLKEHMDDVCSPQLLAY---IRRLVLTLTRQ-----------RGDSQEFVRFFNKQLGSIL-QDSLAKFAGRKMRECGEDLLVDVSEI-LFN-ELAFFRLMQDLDSA---GAGS---APKRKYDSETTGTDTTTGQEGLAEG---------------------------------------------------------------------------------ESSSDEDEEDD---------------DEDSSDEESDTGSSEGDEELQDVETAMAIT---------------------------------PAE----------EEELGKDAITTEVIVKQKEQSDEEKETDSTEKGE----VVIVPDTIEKPIEDKEVTVSPQMNGGTEVEEE----QEVTIDDLPPE---FTSLSQVELEEKIAEEQAENTAVKAALEALESGG--ESELVGNPENLKVPETAPEIAPETDGVPKVQINQINADESDES  
--------------------------------------------------------MGDESCYFVPINSTSLE-------SVRQSDSEVRALNGPRPQSVQSISTTHS---------------ATPVQTPSYDDLRFKMESQSKSMQALKEQQAHLLRLQQAARQQLQEMEAIRNQTVT---------STIPLD-NFESVEQVQDGIS--------------------------------------------------G----IMERMRVLATFIQNQQELGNMLGVDNDDVLNEQVM---------------------------------------LQQKFQELRDKKAQMHNLVSELQNLN-------------------------------------------------------------VEASRQFEGAASKPVERNIP---------------------------------------------------------------------------------------------IELTNAPASAADMRQATKTFNHSSVDHGMMNGTGSRGGSITPKV----------ENVAGERVANLHEEEDDENAEAISGTAEMLN----------------EKINEINAMKSQLR-RLKEMMDTVKLIEMKTGESIDEEEDAPEENDENP--------DSVENSRSPSVASQISDHGAAVGGST---DPRREQLNQRVEALHAMT--RDLREQAKSIAAERDALKNAHTEIQRRRNNVTDLQHQAEAQTAEKLTNHVASFVSSIPPGPKERQQMALKAELEQKKRELERIANMTQSIKKNTELSRHNTEVAPPKVPSVSSADSLRSQ---------SQPPVIPPPPAASAVNNTAGSNITNHSKNSADSGVTDIFANAHLESGSYQSSSTRSLNMVPPMPDICNRPERYRKSEDVGTSTARTDRT---------------------------------------SPWPAHLFSGAGPSSSNTGPQSPHIHPFAGSDIHVSSAYPTYNTYPTYSNL----------------------------LPPHTPNSAPHPSDPL------------------------------------------MFQHFMQTQQMLMNSITQCNQLLWIQQREINNLNNA---VLLLQERILNSANNSILINDSGLVSGAGSHIRAES---------------------------TPPNNTLNSAPSMSGSLYSRARSEQPAMVHQQGPTSPYHTIPPSVHQPVYGNLMPSNQFPSFTPSSTQPQQPHLQQSNQQYSINQQIQR--------NNTNMSNSNATA--------------------NSSSSSHRSYRNLRHVNINTANNAIYEQQHQLHQQQLYDH-----HVPLHSNNYMNQNGTGTDDSNLQPPPQQQQFNNASASGAGSLGPSNMINNLANG--PTSGASMAPPPCLNNLNNSSNLNNVSTQQQTTTQALNNQVLPGVRAN---------------------NYWDNFRSNINQQSNATDLGPNLQQPNVSSHNPTLSQHFQTISQSHQQQQQQQQQQAAQQDSS--------------------------------------------QFQPVSSQSTFLEHQSAPQQSQQHPLQQHHQQPPPQQHHHTPLSQSTS-------------------------------------------------------------------------------FDLGELQFHTNPIN-----------------LDLANKSGHPK-ASGNKKYPMSLRSCRDGN-NLGGESSSNFYGVG----------------AHAEMVSDLNLATASNYQH--------------------------------------DSKSTSKLFEALKENVYQEVKNLITANESRPHFLIQLFRELQLISSDPLRQRTLQSIQE---LYNRYIESTI------QQEQQEG------------HVNNVGSNNLLSS-SDVAG-----------------------GGQTANGAGDAENIEVV---------------------------EVAQN-----FTNVRQQQQQQ-FQFAPSAES-----TPITGGSASGTGANLSAPTKDLEAN-------------------------DLGGMASSE-----IISIIMGDIVGVINSV--DYINDSVLCK---IAGVICNHATG----------------ASNGLF---QQLPAQQEPEQGLLGSPMLGPSMAAFLTQNETDVFS-REDFLRYLESWNRT---DKDEFISNLENLLNNILLRSSAAEGDTAVVSS-----------------------------------------------SNMNGDEEGQMR------SQQLQQQQ-HDSLSSFSNGNNNTTGDISTDNNETFQPYAQGNENPF-PPQVATVSGAVGG--STTRRVY-------------------------------------------ATSESDKMSNG---ISSSNGYALTTYDLAEADQICD---------MDKVHPVGDMAGGAVGGVS-NSGSDD-----QMAL---RNEDTRRWMLLIDRIEMKR-AEKAAQEQPSMDSDDEELPIFY------------------------------------------------  
-------------------------------------------------------------------------------------------------------------------------------------------------------------------------------------------------------------------------------------------------------------------------------------------------------------------------------------------------------------------------------------------------------------------------------------------------------------------------------------------------------------------------------------------------------------------------------------------------------------------------------------------------------------------------------------------------------------------------------------------------------------------------------------------------------------------------------------------------------------------------------------------------------------------------------------------------------------------------------------------------------------------------------------------------------------------------------------------------------------------------------------------------------------------------------------------------------------------------------------------------------------------------------------------------------------------------------------------------------------------------------------------------------------------------------------------------------------------------------------------------------------------------------------------------------------------------------------------------------------------MQPTWDRIFSN------------------------------------------------------------------------------------------------------------------------RTQSTS-------------------------------------------------------------------------------FDLGELQFHTNPIN-----------------LDLANKSGHPK-ASGNKKYPMSLRSCRDGN-NLGGESSSNFYGVG----------------AHAEMVSDLNLATASNYQH--------------------------------------DSKSTSKLFEALKENVYQEVKNLITANESRPHFLIQLFRELQLISSDPLRQRTLQSIQE---LYNRYIESTI------QQEQQEG------------HVNNVGSNNLLSS-SDVAG-----------------------GGQTANGAGDAENIEVV---------------------------EVAQN-----FTNVRQQQQQQ-FQFAPSAES-----TPITGGSASGTGANLSAPTKDLEAN-------------------------DLGGMASSE-----IISIIMGDIVGVINSV--DYINDSVLCK---IAGVICNHATG----------------ASNGLF---QQLPAQQEPEQGLLGSPMLGPSMAAFLTQNETDVFS-REDFLRYLESWNRT---DKDEFISNLENLLNNILLRSSAAEGDTAVVSS-----------------------------------------------SNMNGDEEGQMR------SQQLQQQQ-HDSLSSFSNGNNNTTGDISTDNNETFQPYVQGNENPF-PPQVATVSGAVGG--STNRRVY-------------------------------------------ATSESDKMSNG---ISSSNGYALTTYDLAEADQICD---------MDKVHPVGDMAGGAVGGVS-NSGSDD-----QMAL---RNEDTRRWMLLIDRIEMKR-AEKAAQEHPSMDSDDEELPIFY------------------------------------------------  
------------------------------------------------------------------------------------------------------------------------------------------------------------------------------------------------------------------------------------------------------------------------------------------------------------------------------------------------------------------------------------------------------------------------------------------------------------------------------------------------------------------------------------------------------------------------------------------------------------------------------------------------------------------------------------------------------------------------------------------------------------------------------------------------------------------------------------------MS-----ECLPV--------------------E-------------------------------------------------------------------------------------------------------------------------------------------------------------------------------------------------------------------------------------------------------------------------------------------------------------------LVRLGYSRQ---------N----------LLSS---NSCKSN---EDSCALGGGGGG---------------------------------------GSTST---------------AHGSGGGANNGGGPALPVGGAATGSG---------------------SGNCTNNINNQIQQR--------NNSNVMNNNYGS--------------------GGSTSSNKYPPN---FNRNNSFSNINQQQQQ-----------------LDVGPNLQQPSCGSHQHYQSMPPEAAAASG-------------------------------------------------------------------------------------------------SFQMSQSAFVHQQQVQQQQQQVQP-------------------------------------------------------------------------------------------------------------------HHTPLNQSSS-------------------------------------------------------------------------------FDLGELQFHTNPIN-----------------LGLANKSGHPK-GSAHKKYPLSLRSCRDANLAAAGEGSSNFYGVG----------------AHAEMVSDLNLASASNYQH--------------------------------------DSKSTSKLFEALKENVYQEVKNLITANESRPHFLIQLFRELQLISSDPLRQRTLQSIQE---LYNRYIESTLA-----QQQQQQGGDVQQQLQDGGVHVNNVGSNNLLSSGANEPV-----------------------AGPSSQLA--AENVEVVD-------------------------MEVTQN-----YTNVRQQAQQQGFAFVPSAES-----TPIAGGSA-----------KERDRN-------------------------DLG-LPSSE-----IINIIMGDIVGVINSV--DYINDSVLYK---IAGVICNHATG----------------ASNGLFHYQQQQQLPQQDQDRILGNPMLGPSMAAFLAQNDSDVIS-QEDFLRHLESWNRT---DKDEFISNLENLLNNILLRSSAAEGDSAAVSS-----------------------------------------------SNLNGDEEAQMR------AQQ-QQHQ-HQHDSTLSAGDVSTDNN------ETFNP----SENPFSPPGVVTAAAAAAVPLSLTRRVYGAV--------------------------------------ASSTSESDKMSNGGGGVSSSNGYASTTYDLAEADQICDTSLPGAGGAVGGAQPVAGPSGSSAGGVVGQEGFEDRWRVMQKKLDDD----------LADIIERNRNAERVAQEQQQQDQRHQQQQQQRRSNEGWQDENGEGNLDPEVRLRKWLEEL--------------------  
-------------------------------------------------------------------------------------------MNRSSTGTVPKIKCRN----------------N-TTSCPNER-DS--PRSWTNNLTGDFHRLNLSDYLRNSRRNHCAMNVGTVEEQR--------------LNPALNGSITPSSSSSRRNSSGTVP---------------------------------------KCDNITKYPDKRQIEDKLSQIQEYLQITTSLMSSMKNTDDQL----------------------------------GDANNLMQMINDLRDSEAKLVNILGNIEAEEQ----------------------------------------------------------------------------------------------------------------------------------------------------------------------------VKADRNDNYSESATSHHNDLDEKIDHTNREMEMLRDQQMSLLCLQ-----------QKAE------------------------------------------NKLKDARQIQEKLLTASQYDVNSSINQRNKNLSSVQ-----------------------------------------DFDIAIRELEERSKRLNEPRGSNSNLQ--DKLLAEMD------------------------SLQSQIVTMHNINDDRNQLIQVLD-------NRDTELRAQHLELQNKLSELQNKKSQVDQLVAQLQVLEETD--------------------------------------------------------------------------------------------------------------------------------------------------------------------------EDDVGAQVRRIVTMKEQLSKLKDMLEIVKT----------------------------------------TELNSNAS------------------------------------------------QEEQDLACGICTKAENFLQKDGEKRRTPLN----KQVNKNACDRGFG------------GAKPKSRTN----------------------------------SNKVALQVELEAKKRELEEIMGKHKAGTSNLN------HDVGTDNKS---------EFSCSSNAYFDWAPIMSDHNRFDSSD---------SDEYSDPNQ-----------------------QFVLPTLNYPKSDHDYVAHHLQDTTTDR----------------LSSIPRTPERRTPRAASEQGKNQVQKQLELIRSVCDSMLDQQG---GTNVRNNL--TPSSLYFEPRPMNTTAIVDPNYQSWLA-----------------------------------------TNTLQTQAFMLNTLNQCCQMLWLQQRELISLRQTVNQLQDYGGNLAS---PLQSECRSRSNQK-----GGNQVAAACSMPNLN-----------QYNIPPSVNLDP--SSSYQNNMQNARLLDHCLN--------NVSDHNNSVLHAVNANTNQIPPTHMWNGQALNNQVAPGNRANNYWDNFRSYSRQNLLSTKS--------------------------------NEGFQNISNNPFTLVTTPKCNSD---------------QMSSQENTPR-RRTQFRNNNSVVPPDVLNVNKVDQRNINECDYNNE-----SNSTISEEHTIFKESASRRNEWHEEQVD--------------------------------------DHHTKSKLFEELRENVYKEVVTLISANETRPHFLIQLFRDLQMISSDPLRLKILQAIQT---IITHNLTTN----------------------------------------------------------------------------------------------------------------HTNRQ-----------------------------------------------------------------------------------D----DN-----R---VIKEIIPFLNDHEDDSITHPFLVS---LKQVLLESESF--------QETVR-----DSVFKKHFSNVL-DEVLAQYQGKKVHDVKMHFIQTVSDL-LRG-ELSFIQLIQDTCPD-----------------GGELETFETN-----------------------------------------------------VNQEVTIQNGD---------------------LAEADQGRVEEDDGEVE--EEGAVG-------GFWELPLEVSED---------------------------------------------------------------------------------------------------VEVPSTEFQNNAEAQTQFIKQG-------------------LDQVPTR--LPT---------KSPSNTPSKGI---------------------------------------------------------  
------------------------------------------------------------------------------------------------------------------------------------------------------------------------------------------------------------------------------------------------------------------------------------------------------------------------------------------------------------------------------------------------------------------------------------------------------------------------------------------------------------------------------------------------------------------MQMM--TKELEQTTAAMKEERDKLEAARVELQNMA-----------RKLPLNKSRVDSLSPDDDDSEME-----------------------PPCLKLSSVDSGYR-PLSRENIHSPHSSHNNATTSTNKGKIRQQTGN---------------------GNVPSSSGLDRQSKNDTLS-RCSHPSSSVGGSHNLT--------------------------------RESRWSPLSSVTNTNDRTNFSLQAQNQMEMSGNFCDYALMETIKPDEISLQGMFQQHQMLTKTVAQC-S-----QLL--------------------------------------------------------WIQ-----------------------------------------------------------------------------------------------------------------------------------------------------------------------------------------------------------------------------------------------Q-KQIIELRNALMFQNENYSNIN-----FPTFPTSSYYHGAS--------------------------------------------HSNYTNQFSTFPPH--VQQQQHHHHHHQNPLNNKSNYRGGGGE-------------------------------SSTSSSNVPNQEIQEQQQ------------------------------------------QQQYLNQGLINFSVPDIMDYDMNRMGNVNRIN-------------IPSHGNQNQNSGMNNRNPNGIGCLNPDDSGLALNNQVPPGNRANNYWDN------------FRSYSRQNLLSHTK---------------------------------------------------SNEGAEALRRPTSVATQMRQQRDLLSSHNNQSAGIGTSQHP----------------------------------------------------------------SQTIGN-----------------------AESSTHRDHQYLFSSSQNVGMNHHG---------------------------------------------------------------------------HISRTRSSDSQGNNDSRHFNTASRKNEGIGVGGGGGGGGGGGGIARTRNPEPGCSDNLRSVRNDVNQNLNTTAHN-----------SSQMVERTRGRKESSAATTHLN--------------------------------------NRGYNNS---NLWDNISSEVSNFIRSNEARPQFLIQLFRGLQQITTDPLRERTLESIQE--TVCANNLSDRS-----SNTSGNLNLIENRNNIRENRSNLNRNLSEDPNRIEALFCQLRENQNTIRQNIGISSFVLGGYNNLENPNISNIRDLANPRS----------NNIVNEDL--RDLRTYFEAT-----LCNFGGNHSTLENPNVSNIRENLTRGQGMSDNEHHVPKNSYLNICDNPTLDLRRNLEPSVASSGLEFTN----DPVRLSNNQVESGSNFGEMRGNRDHHFPLENEGRNSSNARQFLAN---ANDSGIDINFN--------NEERK-----RNYGNDESSSCNDTNNKHKKNEERTEGAGHHSSMMMAPP-QWDRNQPGIRENNAFESS---TFNPVGENSFIPVSFLPSSDRTDLAEADQSRNDENSGAESDNDMLLASASEPDNDIGNAQ----NRHNFDNSSIPRSVAQSESSATSTKRNQGFEDCMQNFPMAEPTNDFSISGRSRESSENNFFHIIPESERNFTEARNYESNENAVAAAAAALDFIMKKNSSSGGQSE-------------------------------EMKIGLDEAASTNNHSSNNNNNNNHQTIHNHLYPSSESETSLTGTKYQTAACSAGATGGEDCVEESGGSETKEEDIGNGGYMSNSVLEHYVGGTSSNGL-IIQQDYKLSNSKPLTHSNALGQGDGSEETYSDWEYS---SGLDEVPTKLKKKKKKIATK----------------------  
MAMASGGRKPFSLSKLPQSDSRPTRYSAGDDQNDAEPQMNSSKKNVPKEVRINNWDF-ADWRPNSASTDSKRR-------KKSKGNPEREREQSLSLDSPPQRDKKP----------------RTPSTFPRTKIGPSTPTSQKRALENLRSRMTFSETDEGSTDGEPNNERVVSKRRLN-------PPNFSRQDTNSTEEGASQANLTNRNNN------------------------------------------RDNSVQDLGLDSNQIVGRLMQIRDYIKQANTMRDTLNKSKEARDKEKVIKLKHVIDNLVKQEIYTLETLHQLSKSNKDDMDKLKKLVDNLKDQERGYMGLLQRMLALRDEGVLENGNGTLHALLPDLEDNDYSDNNNDNDNDDDNEDNKSESDDTVSVDLEVQTNASDTTTEERTNSSSSRPYIEDKLGDTDDENESRRSSKESNNMDN--------------------------------------------TLVAQRNAQAVEWDFGVDSIDGLDNLLPGSLDAMRANELAALQQQQNLLKILAEKQEQLRALQGRQDALVAMHKEAEKKLHIAQNKENKAKAAVKTASEAVRHASNSNPNLTP---------------TDDVEEDPEAELSSTQTQLEQLVRMKNRLEMLKNASTELAAEEEQAGNDMA---------------ILQTATDDEPGRQQLQDKLEDLQDKKDRMNDLLMELQTLR--SQRFETLL---------QNDSEGAS----ATAPVVAPPAPNSRSSSQIKLTDALSTLDQADSTAESAEQVLSMMDAQTKLEKLQEVKGRLDQLRGLVQYYQGN--------------------------------------KTRSDGDGGEIRDQSSAPTYSDYGDTESLEN-----ARRAPIDAEKIFQTGSQLNVLGSVPGATSRRRADLDSQQSD-------------------------------------EENASVNDSQMSSLGPWGDEPEIREKVRQLKATKEKLQQLQDLVSMCQQSPDLAQSLPDNITEDDRPS-------------LLTAQQTEERNISLSE---------GEIPNDSQSGLPEAES-------------------LFSVKEQIKELSELKKERERLLALQNQLQSLQGH--FDPEDNDNISDDGNDT---ETNKKSRSQSEPGPVVT-----------------------FASNDELYSKMRKQRMLREELRSKKKELEAIMKKDRNKRQYFK-----NQDNQSDTVSYSTD--AFGASASADATMATWGGSTVDNLENITEGRDDNDDDDDNDDEDDAYPSDGIVQVEEEEEVNGSDNNTYTIEADARQRRAARSKPTQPDVRPKARKIATFPRSQSAREEVLMASQSGNVGKKGAVRKTKANKRTKQENYRSAEEIVRDVEVEDSSRLDLIQQQVQQTYQ--LLQEQHHNNTLQGTLSPNTSGLTMG--------NPMRPAAGMYPD---------------------MLQQQMYQQQMMMS-INQCHQQLNLQQIEMSNLNHHIQQISLYIQEQQ-----------------------HHHHHHPINSTTDD---------------RDHSRLTPLATSSQTHISPHHHLPPPPTR---------QSTFTLNPQFPLSFAPVEGLSPRQHSSHRITSSSPSRQ------------------------------------------------------------DVPQPQTNNLRPIPPPRLDSYQQNSNVNNFLGRDSQRDARPK-TNGEYGKPKKSKDRPEEDYERSSQGHGGQAGKDGRYRPGLSSGISGTAFSDSNSVTDALSARLGEPRN------------------------------------RDGDAMELSLFETLRETIYSEVATLISQNESRPHFLIELFRELQLLSSDYLRQRGLYAIQE---LVSKTLSSED------NPYAVQSRWSRNGGG-DNLTASELTPSESLLTSEDESKE--FVEKKKKKLLLNRGGSLKNNQYDYSEQAENTSTLSTPSTG------LAESPFANESL--GDTVIHLDKA-----LERMREMEMKEIE-EGRLDEESLQVVYNRVREARQAARILEDNKLVPGSGTDNGSQGSIPDIM-----------YSRINSSQLDD-----QVKEIIREIIPVIKEHTDDICSPQLLAY---IKRLVLSLTRQ---------NDSR--HEFTSFFHSQLGSIL-HDSLARFEGRKVRECGDDLLDSMSDA-LYD-ELAFFRMMQDLNNP---NLNNKTKSNQSSKTSSPQKSPLHTRIVNKADLISDDGNDVSSSDSDVDSQNNLRMLVAEEEN-LGKERDDELANEMPIQDADEKDQQSESPYKTQGLKIELAVSE-TKPFTRIGSDEDDDNDSDESHSAEDPSETAVS-----RDAKLSSQPD------------------------------------------------------------------------RAVEINGNLTSPSRPVTNNVTSQANGNINGIQSEEDGDVTADKPTVDNHTTLMNGDVDNNNE----VTIDDLPTS--LNITVSK-EIEKKVVDEQNELTGENGILSTMEPQ----QELAGDGTALKDPDSFGNLN----------------------  
MTSNSGASGGRLPPSDARNLAAMNRLINSVERADSAERGSA--GSSWLDERPNNWDF-SSWQPSTTFDNTGAR-------RMKKANEAKEQNHGVERERPRDTEQRRPAAEHFTPEEGRGNHRHRPATFPRTK-GP---LSQKAALENLRQELSYSDIEEVTMDTGEWNNERSMHRDVR--------SGLTPLQDMERNEMDHKEAEEG-------------------------------------------------------PDSNQIVGRLMQIRDYIKQASTMMERLRKVPD-AK-------------------------------SCEQFEKMSGIVCHLKEQEKGYLSLLQKILVSREEDVLMRQNS--STLCQETP----------------SESEATTELDAFVINSSEDDENIARNAMSLNLN-NTRPMIEDRLGRHGDTDNEEAINS----------------------------------------------ARSSNPGNDEELSWDDGNTFPAINELLSKKSELQSYLENKNGELAALRQQQELLKQMLQQQEQLKALHGRHAALTALQEEAEQGIDAQGATALNNEGSVKSDNRDDLGDGACGGGDDE--------------LIPQQIVELRRQLEFLKNTEQMNGHPSPPPAKKTP-PPPRPPPPHPPPASIQ--------PDQNAAELEQLSIASTERR-QLQDKLLQLQHKKEHMDSLLQELHSMR--AERLVLNN-----------------------EVENGSNCSSVGR---MADGMLATASADHT-AEEAEELLEML-EARNRLKKLQDVKERLNELKNLVQYYETG-------NEFIHG-----------------------NQGQGQIEVHDLEEV---SSLVANDFEDLPPAK--------NMSLKKESENRRRSMNDGAGSSRQP---------IDPS--------------------------------------DTTETTSQMSSSAEGWNEDPQVQEKVERLKEAKEKLRRLQELVAMVQHSPDLAHALPNDLADLAA----------------SVQDDTESQFSSQARSPAAAAVAAVSRANNISAADGAAALSFQDR----------EQLYQRKMDEQKDELHDLMQERDRLLRVQGELQHLHDALPAQPTERSRTESVNDS--SRRSVDKCSKGRSEPPRPARQAPQDVTVTFQEPLET------VVSNEELFDRMRQQRILREELRERKKELEQFMRKGNDRPRR---HYGRNQDNQSDNVSYSNKSYQDGASASEDVTMATWGGSTAGNLESIEEGEAGRRMAHEDHDEDDAYPSDGVVQVEEEEEANGDSD---RDTYTIEDED---------------YRLSAR-----RDNRHQHQPMEQAPAPLGEASQQARRRQHREAESLSATAPFQDGSYTFNNWSTNNQVLNNTRNT--RVKSARRRRRQQRGLSAEDNPILYKMQ------QDIRELRTQLDSQNSPSSS----------EGLQHQCSQLQQQQLLMGLNHCYYQLLQQHQQLIERFGLLENYIVEQ----------------NSDSRS-----PRAGRHPSDP---------------------------------------EPRYPFDVVS----------------ERLLATASIPRKVAPKRSPLNDLSSQIPIDTIQARDMEVPLRLNLDEAVR-----------------------------------------PKKQRSPKSKPQ----------------------------DVVIGRRFPTPSRAGAAAMLAQQKTAAAPYR---------PGLSAGISGTAFQATSSVSSAVSSLPGPDNDR---------------------------------PVRREEDVPLSLFETLHETIYSEVATLISQNENRPHFLIELFRELQLLSSDYLRQRALYSIQE---LVTRYLTDEK----VTQTKEKPWEVHSNASN-LRQPDPTSTDDEDLKQTTKEICE----SGD---------------RFDYAENVATTSSMSTPTSSTQPNGSWAETPFAQESL--GDTVIHLDQLRHEYNIKRAAYFTRKALRRMRDYERK--LATAGQPDPQGASPTVSSAPPPAVPSRDDPQSDGSMSDVP-----------YPRIDTQQLDK-----QIKSIMTEIIPVLKEHMGDVCSQQLISY---IRRLVLSLAEHPPESPAAMVQQPPHSQEFVRFFKNQLASIL-QDSLAKFEGRKMRDCGEDMLVEMSEI-LFN-ELAFFRLMQDLDDP---KAKVKARLKQWREDSCTSQTSTETAVTQDEPQFPISMANQETED------DEEEEEVSSLTSSSSRDED------------------------------------------GLTS-ETEDNEKKLP-----------------QPQDEEEELGKTRDDAFAMELAATAE----------------------------------------------EKKVNITLAPSETKPFTRIGSDEDDEAEDEVASNGEPSETAVSRDAERAAASAPSD-SQSEEEKNDGEKDDDVEELLSPNDLPSQ--LSV-ISKDELASQMEAEQ--LTLENDLLTSLQD----VQELAGDPMALKAPGDDSTIANGSLTLNLTEEQPANNSSEA--  
-----------------------------------------------------------------------------------------------------------------------------------------------------------------------------------------------------------------------------------------------------------------------------------------------------------------------------------------------------------------------------------------------------------------------------------------------------------------------------------------------------------------------------------------------------------------------------------------------------------------------------------------------------------------------------------------------------------------------------------------------------------------------------------------------------------------------------------------------------------MTEQQGLNHNQSLPFPY-----------------------------------------------------------------------------------------------------------------------------------------------------------------------------------------------------------------------------------------------------------------------------------------------------------------Q--------------------------------------------------------------------------------------------------------------------LR------------------------------------------MSEYN----------------------------------------------------------------------------------------------------PLSYQGNFAN------------------------------------------------------------------------------------------------------------------THTGNVMTMSRMS-----MNNQHAETNISEHHEKTIWP-----------------------------------------------------------------------------------------------------SFFKIEDPAMGGNDSELEKNK-----------------------------------------------------------------------------------DITP----------------------------------------------------------------------------------MTANAPESTG--------------------------------------------AVNFSLFESMRDLIYSEVATLIAMNESRPHYLLELFRELQLLNSDYLRQRGLYSLQE---LVTKFLTDSNTDSSPAKSAFVQETVSNQQVY-DNWVESAQTPSESGTTTEDEG------ERHALQSLAKQMENNEMYDYAEIVEQQSDGNFSTPRSS------IVDLPFASEDL--GTTVINLDEA-----LQKMKLYELKMAETQKVFEQASNNLSEFIPYTKQSQKQ-----CFNDAQNSNLSSEISFDCHN------------STLDAPLLNQ-----TVKSLMMELVPFLNEHISEICTHDLLDA---IRCKIVHLAHQN----ELQVLNKPESEQFHRFFHNQLESVL-KDSLSNFSSRKLKDCGEDILIVVSEI-LFN-ELAFFRLMNNLDSH----------PNNKAEVVNNMQASKSSSNSS----------------------------------------------------------------------------------SNFVIVDSSELTDEENESLRNFVKVELAVSEIHPTQSDRDDSE------------------------------------------------------------------------------------------------------NEMSCARTAVPITSLNYYSQDVNGTHFNTKDVNGTHFNTKDVNGT----------HFN-T-------CIVLL-------------------------------------------------------  
----------------------------------------------------------------------------------------------------------------------------------------------------------------------------------------------------------------------------------------------------------------------------------------------------------------------------------------------------------------------------------------------------------------------------------------------------------------------------------------------------------------------------------------------------------------------------------------------------------------------------------------------------------------------------------------------------------------------------------------------------------------------------------------------------------------------------------------------------------------------------------------------------------------------------------------------------------------------------------------------------------------------------------------------------------------------------------------------------------------------------------------------------------------------------------------------------------------------------------------------------------------------------------------------------------------------------------------------------------------------------------------------------------------------------------------------------------------------------------------------------------------------------------------------------------------------------------------------------MRKKRERGRQQIR-YEVS--------------------------------------------------------------------------------------------------------------------------------------------------------------------------------------------------------------------------------------------------------------------------------------------------------------------------------------------------------------------------DAGSEFSLFEALRDSIYSEVATLISINESRPHFLIELFRELQLLTSDYLRQRGLYALRD---IVTRFLTEDS----LTTN----------------------NVMDSAIKQACQ-------AGE-------------------------------------------SP-FASEGL--GDTVIHLDQT-----LSKMREYERMREEGRLRELKQ--LQYGATGGG---AEGGAVKDDVTTSSAGDVGSESSMSDVQ-----------YPRIDTQALDH-----QIKSIMAEVIPYLNEHMEDTCSMELLGY---IRNLVLTRIRV-----------KEE-QEFGRFFHKQLSAIL-LDSLSKFEDKKMKDCGEDILVDMSEI-LFN-ELAFFRLMQDLDA--------------------------------------------------------------------------------------------------------------------------------------------------------------------------------------------------------------------------------------------------------------------------------------------------------------------------------------------------------------------------------------------------  
--------------------------------------------------------MASNLKYSSSSEPRPKH-------LRVSRITNDRTLAQSAPKPSS----------------------KKNATTPLATMQSAPSNESHRLKDTINRKIAF-DVDEEEINSMENGIDDIES-------------------FTTENQSDIS----------------------------------------------------------EAIDSQMVYERLMQVRKFLKQVTQRYKELQTIEVNDDGRK--------------------------NLYAQQKIRLAGFIKTLKGQEQGYLDLMRKVMNTEN----------------------------------------------------------------------VRSEADT-----------------------------------------------------------------------------------------------ESNLSETLRYDDQIETNNPTNDLVIDDSKLLTILQLDCFFAYSLR--------------SKSNALLQADATEDAAEEMNALRNKY-----------------AIMQNILTQQKELNELRSRQAALVALQQRSQDRQNDLKDETDKSSEP----------------------ESANKRQAELLNKMLLLKHRQQHI----------Q--D---------------------------------TESMEKFADVNDDP--------------------------ELLQKLRLLQESRSRVARLKELI--------------------------------------------------PEVVIDD-------------------------EEENAGSSSIDDSLAISTEDPEIQEKLTKFAYWLLLISR-----------------------------------------------------------------KLNVAKKRLQKLKELMKIVQEVSS-------------------------------------INRPRLKK---------------------------------------SEKLHTEESNKSDDVDHFQEEQLHKLQEKQEKLLHLKKV---LEELKSSLSDSLNLP--------------PAPTDDLAAKSDKRNDLALNNSN----------SALWGELRKRRELQEELKSKRKQLQQLLAVEKAKEMEG----------LANGPSVNGTEEVMGYGYDGATTQATWGGSTPDVGDFNAN------------------------------------------LEDDLDDGYVATRGMEEDSASSESNEIES---------------DDRKEFKSDRNRSRDYRNVDNDLLSRKELLAVKRQYAGLGKQMDG--MKSLLQS--LAVNLRVPSKSDEEMYLSEPNERPNFG------AVRKGQ-----------------------------DQVYMRQLEMSNTLNQCLLQQMKQQQEIQEVQKQTNQL--------------------------------------------------------------------------------------------------------YWMFMDRRHKYTDQALGRLY-------------------------------------------------------------------------SDSSLTSGPESTN-------------------------------NERSFQRSKKGK-----------------------------------SHEDLLRRIASDRESGITS----------------------------------------ATDQSLFETLREPIYSEVAALISQNEDRPHFLIELFRELQQLTTDYLRQRAIFSLQD---LTRRYLREDT-----IATREPQ---RNQRPWALSNATSEHTPSESIATIDDEEMQAQLGGRRSNRRHTQD-DDISCLSYDYMEVVDSATSMATPPSNSSP----YDLPFASDSL--GDTIIHLDKA-----LKEVRAVDERARRGTFTIETE---MAKEGVDKISKASSIESHKRKGDATLSNNSSAYDVESSSSLS-----DVPYPRVNTQELDR-----RIHVILKELVPYIKEHSQESCSSPMLSA---ICARVLSLAQN--------QTENE---EFARFFHRQLNSTL-IDSLGKYIGRSHEKSSQE----IPSY-LPS-GSGNQQYTSEDGND---NSEIDSETEAESAEDKPVDSVHEREVKT--------------------------------------------------AVDMLSKDKSDQS-------------------MDVEMEELGKDRDDAMANQFEERSIELMQETEFIDCTGDSAQT---------------------------------------------------------------EDFNKISTGNNLDGKQHSTEGNSTISNETITDDNGNSADMEVKSFSKLDDED----DQHVANTGTADFENDE---LGVEHLPSK--LDV-MTKEQLLEKIEDENRQNDPVVAVINTSSDKDRDTTGKCAFHTLFLIFNVVFKL-----------------------  
---------------------------------------------------------------------------------------------------------------------------------------------------------------------------------------------------------------------------------------------------------------------------------------------------------------------------------------------------------------------------------------------------------------------------------------------------------------------------------------------------------------------------------------------------------------------------------------------------------------------------------------------------------------------------------------------------------------------------------------------------------------------------------------------------------------------------------------------------------------------------------------------------------------------------------------------------------------------------------------------------------------------------------------------------------------------------------------------------------------------------------------------------------------------------------------------------------------------------------------------------------------------------------------------------------------------------------------------------------------------------------------------------------------------------------------------------------------------------------------------------------------------------------------MCP--------------------------------------------------------HLVQ--------------------------------------------------------------------------------------------------------------------------------------------------------------------------------------------------------------------------------------------------------------------------------------------------------------------------------------------------------------------------------------------------FEGLRDVIYSEVAALIAQNEERPHYLIELFGKLQLLNTDYLRQRGLYAIQD---ITSRFLTEVN-------------------------------QGKASKRSNSA-------------------------KFDYVEKVDSASSLSTP-------------PPP-NNN--NNNNEDMEEP-----LHNVVVVDPLSVMVSGGS---------------------------------------------------------SDINS-LMDD-----KIKIIMTEVIPLLKDHLNTICNVELLQY---IQESILNIVIKS--DKEVLNVDESGGGQLKNMFLKQIEISL-SSAIGKYINKRLGDIGEDLLVDVSEI-LFN-ELSYYQLLKEQX---------------------------------------------------------------------------------------------------------------------------------------------------------------------------------------------------------------------------------------------------------------------------------------------------------------------------------------------------------------------------------------------------  
--------------------------------------------------------------------------------MILLRLLSSELISARMAESQPL---------------------NTKAAAPTEEAPTEEIIDDDDTNQEINNS------------------------------------------------------------------------------------------------------------------------------GYIEDAEELYEQLQDQSD----------------------------------NEEQMERVSELIRQLKTQELNYLQVLLKVMEDND----------------------------------------------------EDDDDVDEEISKENESLSLRDEINK------------------------------------------------------------------------------EEEEEEGEELVDDDDEEFDLLA--------LQEQHQLLKKLLEQQQQMELLHAKQAQLLSMK-----------KEAEERLAAVMIEKESLNTTTTS-----------------------QKEEEI-------------------------------------------------------EEET-----SVNIEELDIIQQLDEMKEKRAQVEKLLLQIQSIK--NSGGEGDI-----------------------VPVSVSDADGTSER-------------------------VEPEDTSEKTKVFDDVKSNLRSMHDQLLKIESA-------------------------------------------------------------------------------------PNLKATPGPGTG-----------------------------------------------------------------TDPIKRAASGPEVSAN-EELKRKRNELKNLEKMLN---------------------------------------------------------------------------------------------------------ELQSLTSDKVGDVSIQRKLEN--------------------------------------------------------------------------ELIRQKQLYTTLLKKTSQLQQLQKDIEKLSRG-----------------------SDQESGVVDTTAGTWGGSSEDEIEQ---------------------------------------------------------------------------------------------------STATIENKAKTNDGQLFNYSVHKHSSKLPQPTMANDNTNSQDEF--LRDQLRKSTDMCG--------------------------------------------------------QLMDQQSLLLNTLHQRLDSLPYIQEQMSQLQQYHSYLQG------------------------------YNEYLNNAY----------------------------------------------------------------WQVCSEATG----HMNSSTLKEENETTANSSFQEG---------------------------------------------------------VIPPPPAPFPPMN--------------------------QYAGATPFSPFARPTN----------------------------PHLQYTPLFKQQDVPPSVS--------------------------------------------ASGKQVQ---FEGLRDVIYSEVAALIAQNEERPHYLIELFGKLQLLNTDYLRQRGLYAIQD---ITSRFLTEVN-------------------------------QGKASKRSNSA-------------------------KFDYVEKVDSASSLSTP-------------PPPPNNN--NNNNEDMEEP-----LHNVVVVDPLNVMVSGGS---------------------------------------------------------SDINS-LMDD-----KIKIIMTEVIPLLKDHLNTICNVELLQY---IQESILNIVIKS--DKE-LNEDESGGGQLKNMFLKQIEISL-SSAIGKYINKRLGDIGEDLLVDVSEI-LFN-ELSYYQLLKEQDDL---PVEWKPLPISPKKPASTVLHGISLSQ---------------------------------------------------------------------------------------CE---EEEKEKLG---------------NTRDDELAKSWHQPMTDEGNGAKINGIS-----------------------------------------------DEVEGNPVTNSSET--------------------------------------------------------------------------------------------------------------------------------------------------------  
----------------------------------------------------------------------------------------------------------------------------------------------------------------------------------------------------------------------------------------------------------------------------------MSLMFFQDDSEPRDRDRETETER-------------------------------------ETKQQQQQQQQQQQQEQYLFNMAATAPHT-------------------------------------------------------------------LPSSRRGTSGQPPR--------------------------------------------------------------------------------------------RLSFQDRPPQESAVDRRPELMQFLANELSRMSAAADDNQG----E-------------ADAVIEVLDESTTASQGERPSATQ--------------------EAADENDD-----QGDDEDADTDIDWDTIQMIQN--MPDEVFAQLDP-----------------------NTRQQVMHLRGKFADLQQKQAQLQALRDQMAMLTLMAERAAAEEAQNQDIDYDEEEEQPPEYAEDEANADDDEADMAALVAAQDLTSNFDDMLSGAAAQGASPEQLAVLREKFEQLQRLKQEAQRLFELKDHLTR---------------------------------------------QAAAMQDMQD--------------------------ADEEEG-----DEDVD------------------------------------------------------------------------------DEEIDEEEEAFANANDPTTRI---------------------------------------------------------------------------------------------------------------RAMLAEAQQQ----GQQLQQLR------SHMASTADATAS---------AAAVDRGDATEED--------------------------RQVLMAELSRQRALKDQLEDKLKQLHALQAEADRLRRVKQEAD---TRYKSQLEEYEEQLAATEAQGTPERRRVRYAADGPAASATSPS-----------TAAAAATLQR----------------------MEAMMSQLQGYKAMKRGEAPESRFASDAQWAVL----------AQPIRPEEAFNEEHDGSQADFLPGRISPRSGSVSMAPLTNATIHTQDLEELEEERVAQEQMQFDDVMASAYDDLESAMATQLV------AALESRSR--PN-----------------------GVAREPLAMYLEE-DDYDVDEDDQDEDDQDEDDEEDDIILLDNE------------GDDADAS-----EGDAYVAPHRRDS--------------------------LIADLGALRQRTSNLRNQIR---------------FVKSASIVDEDEFNDTSDSPAPRHMDVYDPEERRREELHN---------------------------------------------------YNLAMSFASMLDPSDRADFFAMFFTRFRQNPYGAFAEAACAYSLIAADLHQGQDSLCLNAAMHRRVEAGSNGDMAS---------SSLLVDAAVGPEPSQEDAQAQSYNEQR---------------------------------------EQTA----QHLRMAVYDEVATVITLNEDRPNFLLRVFHALQGMDNDYVRQRAIEALDA---IASDYLHDTT------IDGTASYRGLYDEAL-------SAVEDGSMNSQDDNT------DRGSDVEA---------SPTPAQPRPSLPRVFGHSLDQ-------VIHTLQAPRE--GQDLAEEDES-----HMLVDEFRAELVLHREIMDVIHDAVDVAPEDAVVDASVIDAICHVIQRAGEALADVEEDADAR-----------VPAAATAEAEE-----LSGESMEEIAE-VRTAIDGLKGDES-QL---LRRRFLDMKRLR-------EERIR---RYQTLIEERRVALS-GTGSATAEGSSRPNTSAQNDASDSEFDDVV-EEAWRRSGNDQGVD----EDCEYNFDGNNDDDDDDDEANEDDEDVNDDIDDDVDDDVDDDE----DDDDDDD-----------------------DDDDEDDEDDQVGELSGAMQAARKRAE-SYQLTQSELGEESGADSAEAMSDGAPDGVQRGLFDITLDDLPADFRLDNDALDALTQSMALD--------------------------------------------------------LAENPDLAALARADFGDAASPLAFGRAAGRTKKHDDDEDEDGE------EYDEDEDEDQYGEEDEDEYDQEDAESDG--YFDHDEDFLDEHDSPVEGALDSASTPATDGDPDMSSATVAEFKPPTNAIEPTVATEDD---------------------  
-------------------------------------------------------------------------------------------------------------------------------------------------------------------------------------------------------------------------------------------------------------------------------------------------------------------------------------------------------------------------------------------------------------------------------------------------------------------------------------------------------------------------------------------------------------------------------------------------------------------------------------------------------------------------------MDSSNLSIP---VAAENHLITSDIPIGYNN--------------------STCLINSDNYEFGVKLNLLLQQEADLALAEQQLKALEKLKLDMMMTG--------------PFN-----STITTDEFALKSSHNDSVANVIPKPHWNPRFASLSCP------IAPDILLQSNSLVSEHSSQNTNLV-----------------------------------------------DPNTVEDHAK-------NVWSD---------------------------------------------------------------------------------------------------------------------------KQLEMYS-------------------------------------------------------------------------------------------------------------SLTALTQELE-LLE-RRQMN----------------------------------------------------------------------------LMNNNVLSDSNHASTNPLSLLKNENGAVQGLMD-------------------------RLAAASVHDQLGPNILTQYSSN----------------------------------------------PVSTQGVPVCEEMSPNPCQSTDTTVLS----------------IQQTSPDSVHQLCRHDSIDSIQTPLAANNLLKSAVSLAEPVS------NNLNQ--MTTALGVDVDETNAEIQDGMN-------------QINSQIMILEK-----------------------ARAYARNPTEIAQIDGMLEKLVTQAHELHTVEKSLAQYHKLLAEQQS-----------------------------------------------LQALLSATENIQLLNANDSVFLSSNVMVTDMP-------DSLPKLPAKKLAHSLPLLKHNMNLESSKSIKKKPLGRASTK-----------------------------------------------------------VAALQSISPLAHATSIANIDHCNASVSTKHSASVFDSNPIKSKTHGTLFKKTVASPSAPLTQIPIADIEPESYINMS------------IQSINDMTDVPSSANPATDSFVF--------------------------------------ESDHINRLFAQHKDEIYRRAADTISTHDASPYFLLSVFKTLSKLDSQYARERVMIALDE---IVDQVQDIKT--------------------------DSSISTKPTMKSVGSQ------------------------------QKKTQDANIKLKPNL----------PIHSNSLPPHSVSINQDQP------------KPLK----------------------------------------------------------------PRLSD---KS-----QLQQLIHQA---LQDHITQICNQAILSASTTAKSAVFTLPILED----LMIQTHSSIYSYVSMLHIKADGTTGEDAHS-HAMKSVASQRSAVESVLGK---FK-GLSVEKYCHELSNT----VSTLLNTMFDKELNGNNKDEVASQFCTQPSSESTSNVAWKAVR------NASIPSNPTP----------SAPSLVLLKSDSEIDAECEID-DFYDDTYASRYNH-ETGSHPFDDVEHDSNGDQIES-RSNPSDMYTSVNTGLATTDYDCEANLNYANDDCSELESDED----------------------------------------------------YEDEYDAECQLGFDVTDNNKSNSSITLPCTISINDETEMDREDERAADLLEERVELVKQQMEIDLALDDMIRKEHVAGT------FTDLQSPQKIKSARHYINVDCNNPIDPNVIIVEGGKIQSKPNAMVDSVYTGSAL----------------------  
------------------------------------------------------------------------------------------------------------------------------------------------------------------------------------------------------------------------------------------------------------------------------------------------------------------------------------------------------------------------------------------------------------------------------------------------------------------------------------------------------------------------------------------------------------------------------------------------------------------------------------------------------------MTAFIEDAKALASEEYIATSSCPRSSNPSPDMKLIDAAEIDDIMKAIDA---------------------IASSELEVSPALKQLVELKQMHEVRDTQLRQLQALR--EQYERNE--------------------------------------ERANEQLSVFA---------------------ERQAELEELRDQFAIVKAMRGY-------------------------------------------------------------------------------------EGS----------------------------------------------------------------------------------------DDIDPGERDIVSGEQRTG--KQTLEMLTD---------------------------------AEQQD------------------------------------------------------HSKVAALRRELAFLEDIKRG---LDAKKKALQDA---YPAENVTVASVDQLAN--------------------------------------------LDSAMSDQHRVAEAANVHFQQNEDPREFALEELMRRLEAAS--------VGGNGQ-------MRRAEPRVPSHLHYYAGNAEQRVDQS-------------------------------------------------------------------------------------------VPEDVSDADIQNIEQGMQEVLAQMGTVEKAREMATDEQKQ---QFDELFAR--LKGQLG-------ELVQVQNKVTYFRN-------------------------------------------------LLKTQASVGQSSL-QQERNIEQVQSAPSRARAGNGPSN-----------ANSKRP-----------------------------------------GPNFPAVHN---------------------------------LLPYDHRSASPGQYRS-----------------------------------------------------------------------VEVDQAVRGLNQYKEGGFIE---------------------------NRGDASWVEAAEVDEYDIKEEVPDVYAEG-----------------------TNMSGNDMETSIT--------------------------------------PEAKR-FLFDQCKDKIYRCAASLISKHETEPYFLLQLFRGAEKLGNTYLRQRLLLALDD------------------------------------------------------------------------------------------------------------------------------------------------------------------------------------------------------------------------------------VLEE------------------A-----------------------------------------------EGLERERGR----------------------------------------------------------------------------------------------------------------------------------------------------------GEERPTKRGRFFEENQV---------------------------------------------------------------------------------------------------------------------------------------------------------------------------------------------------------------------------------------------------------
