# Supplementary material for: Coiled-Coil Proteins Facilitated the Functional Expansion of the Centrosome
Source: PLoS Comput Biol. 2014 Jun 5;10(6):e1003657. doi: 10.1371/journal.pcbi.1003657 (PMC4046923; doi:10.1371/journal.pcbi.1003657)
Supplement: Dataset S1 — Multiple-sequence alignments. This file contains alignments for the protein families spd-5, AKAP9/PCNT, PCM1, HAUS7 and HAUS8 in FASTA format and as HTML pages with highlighted coiled-coil domains. (ZIP) [file pcbi.1003657.s021.zip › alignments/spd-5.html]

Multiple Alignment


1  
|

5  
|

10  
|

15  
|

20  
|

25  
|

30  
|

35  
|

40  
|

45  
|

50  
|

55  
|

60  
|

65  
|

70  
|

75  
|

80  
|

85  
|

90  
|

95  
|

100  
|

105  
|

110  
|

115  
|

120  
|

125  
|

130  
|

135  
|

140  
|

145  
|

150  
|

155  
|

160  
|

165  
|

170  
|

175  
|

180  
|

185  
|

190  
|

195  
|

200  
|

205  
|

210  
|

215  
|

220  
|

225  
|

230  
|

235  
|

240  
|

245  
|

250  
|

255  
|

260  
|

265  
|

270  
|

275  
|

280  
|

285  
|

290  
|

295  
|

300  
|

305  
|

310  
|

315  
|

320  
|

325  
|

330  
|

335  
|

340  
|

345  
|

350  
|

355  
|

360  
|

365  
|

370  
|

375  
|

380  
|

385  
|

390  
|

395  
|

400  
|

405  
|

410  
|

415  
|

420  
|

425  
|

430  
|

435  
|

440  
|

445  
|

450  
|

455  
|

460  
|

465  
|

470  
|

475  
|

480  
|

485  
|

490  
|

495  
|

500  
|

505  
|

510  
|

515  
|

520  
|

525  
|

530  
|

535  
|

540  
|

545  
|

550  
|

555  
|

560  
|

565  
|

570  
|

575  
|

580  
|

585  
|

590  
|

595  
|

600  
|

605  
|

610  
|

615  
|

620  
|

625  
|

630  
|

635  
|

640  
|

645  
|

650  
|

655  
|

660  
|

665  
|

670  
|

675  
|

680  
|

685  
|

690  
|

695  
|

700  
|

705  
|

710  
|

715  
|

720  
|

725  
|

730  
|

735  
|

740  
|

745  
|

750  
|

755  
|

760  
|

765  
|

770  
|

775  
|

780  
|

785  
|

790  
|

795  
|

800  
|

805  
|

810  
|

815  
|

820  
|

825  
|

830  
|

835  
|

840  
|

845  
|

850  
|

855  
|

860  
|

865  
|

870  
|

875  
|

880  
|

885  
|

890  
|

895  
|

900  
|

905  
|

910  
|

915  
|

920  
|

925  
|

930  
|

935  
|

940  
|

945  
|

950  
|

955  
|

960  
|

965  
|

970  
|

975  
|

980  
|

985  
|

990  
|

995  
|

1000  
|

1005  
|

1010  
|

1015  
|

1020  
|

1025  
|

1030  
|

1035  
|

1040  
|

1045  
|

1050  
|

1055  
|

1060  
|

1065  
|

1070  
|

1075  
|

1080  
|

1085  
|

1090  
|

1095  
|

1100  
|

1105  
|

1110  
|

1115  
|

1120  
|

1125  
|

1130  
|

1135  
|

1140  
|

1145  
|

1150  
|

1155  
|

1160  
|

1165  
|

1170  
|

1175  
|

1180  
|

1185  
|

1190  
|

1195  
|

1200  
|

1205  
|

1210  
|

1215  
|

1220  
|

1225  
|

1230  
|

1235  
|

1240  
|

1245  
|

1250  
|

1255  
|

1260  
|

1265  
|

1270  
|

1275  
|

1280  
|

1285  
|

1290  
|

1295  
|

1300  
|

1305  
|

1310  
|

1315  
|

1320  
|

1325  
|

1330  
|

1335  
|

1340  
|

1345  
|

1350  
|

1355  
|

1360  
|

1365  
|

1370  
|

1375  
|

1380  
|

1385  
|

1390  
|

1395  
|

1400  
|

1405  
|

1410  
|

1415  
|

1420  
|

1425  
|

1430  
|

1435  
|

1440  
|

1445  
|

1450  
|

1455  
|

1460  
|

1465  
|

1470  
|

1475  
|

1480  
|

1485  
|

1490  
|

1495  
|

1500  
|

1505  
|

1510  
|

1515  
|

1520  
|

7209.LOAG\_12060T0  
7209.LOAG\_12058T0  
7209.LOAG\_07437T0  
6279.BM21319  
6287.DimmContig81\_DIMM04910  
6287.DimmContig81\_DIMM04915  
6293.WUBG\_11008T0  
6293.WUBG\_04256T0  
31234.CRE16385  
31234.CRE20025  
6239.F56A3.4  
6238.CBG23743  
281687.CJA01271  
135651.CBN07378  
135651.CBN18152  
37862.contig14568.p7  
6326.BUX.s01147.232  
36090.scaffold402.1\_reconstructed.m18  
6253.GS\_18507

Loa loa  
Loa loa  
Loa loa  
Brugia malayi  
Dirofilaria immitis  
Dirofilaria immitis  
Wuchereria bancrofti  
Wuchereria bancrofti  
Caenorhabditis remanei  
Caenorhabditis remanei  
Caenorhabditis elegans  
Caenorhabditis briggsae  
Caenorhabditis japonica  
Caenorhabditis brenneri  
Caenorhabditis brenneri  
Heterorhabditis bacteriophora  
Bursaphelenchus xylophilus  
Globodera pallida  
Ascaris suum

----------------------------------------------------------------------------------------------------------------------------------------------------------------------------------------------------------------------------------------------------------------------------------------------------------------------MLEVLIRRPTESKELLPCNPHQHRYTMKGEKEAAEMVKEKFLEPIGVAE---------EGKMMK---DKKFSNDKKLHYKLNRNIGKRMAFGKTTRDN--EFEYNFVNSNRNNQGDMQDLLMEEATPVATA-FSKGTSSLPRRRPYVSIAETM--------EENKQLKAEMF----DLKA-----------------------------------------------------------------------------------------------------------------------------------------------------------------------------------------------------------------------------------------------------------------------------------------------------------------------------------------------------------------------------------------------------------------------------------------------------------------------------------------------------------------------------------------------------------------------------------------------------------------------------------------------------------------------------------------------------------------------------------------------------------------------------------------------------------------------------------------------------------------------------------------------------------------------------------------------------------------------------------------------------------------------------------------------------------------------  
---------------------------------------------------------------------------------------------------------------------------------------------------------------------------------------------------------MMAMKPDNEKVRRIKMEQM---WQSEKKKFDDERWKFENEENDWTMKREGILMDRNKLAP-------------------------------------------------------RLKELRQQSS--GRNDQRD--SKDQSDSSLSTSSLISKT-----------------------------DIRMALFVVDQLLKMLQGN-TKTA--FTSPSAMYRCE--------------EAS----------------------------N------Q--------------------------------------------------------------------------------------------------------------------------------------------------------------------------------------------------------------------------------------------------------------------------------------------------------------------------------------------------------------------------------------------------------------------------------------------------------------------------------------------------------------------------------------------------------------------------------------------------------------------------------------------------------------------------------------------------------------------------------------------------------------------------NKRKS------------------------------------------------------------------------------------------------------------------------------------------------------------------------------------DSNVKP------------T----------------------------------------------------EACGR----------------------------------------  
----------------------------------------MASGNIAGDSDIEHNFETSD-RDGQ-DATQDSLTEEAKS--FL--NARKPIRVDISQKEQ----LDCRSSALTPNTVKHTRNVSSDATP---V-AAAFSEGALSFPHRRTYLNIAETKEENKQLKAEVFDLKSQLFMLKRKLPSILNNEGK---DFS----LEVNHVDFPLIPNLFVR-----CLEGTVKNEKERSKFENERNDWEKKRERLLMDRDELAS-------------------------------------------------------ELKELRQQLS--ERHGERDDGNESQGDSSLSTISLISERGMEI------ERLRSVVLQQSVDEKKLRMKMEKDMIELQDQLKLAQNNLTKQA--VDYAKEKTRCETLEAEMTKLQQIADEAKQEV-ECQKQISEEQLAKAHAVNEV--ALN----KRDRTIRILLK----KLKSADAETSSNSKIEGNSSSPLHHDVPNLNPYAQTVLDQINKFTIE---------NDAIRKKVIEWGTINIGLTSEIES-------------------SETPSAEDENSEWNTKRCERENEKLAVFLAQN-KAIPDGLNEEELGEQLALFDAV-------------------NGSEGRLSKVQQDDTARSIGAS-----------EMLDI-TSVADLLSKSLVLTEDIGEVKKKLSVLQSACTRLFEKLRGTANFLQTLLDELGAGDRGRELLDEIEALRIDLDHSLATAIDISRDVEAAEESIGNLSTHLQRSLLNCSFGMSSVPSSENQQNASSLSRAHRAY---RRLNADLANEKMKANEATELN-EKLQAHVTDLEELKQKLINELDELRRNLVKKENEMISLVKDMEIQLQHKNQQYDELHGKMEELQQRHKTKEILAAEREAKMKQLTDQMTGKCHKLEIQVAEVTHALKMKDESI---------KKLLIDIESYNDQLLSRNNELTHFKNEIIGLDNKMCELIGTVKASLGIVEEQVDKPIIEAANTEIAALTQLSQIGADLE-ELTKVSHYIADLKNQMIKLQQDLDLCERSRNAISLKCHGLSDQ--NTCLEAERNEA--------KQKLLTLQQQVQMEKESVKMMERDLQRLESENSCVKENNERLTKMLVNVEAKY----EEMTSKLNRSSLCEGQHS-ERYTVTIGTMTLLSSDDFIELTDKAKTLSSFTKRMYSLAARWNEEAAVTPRVSYKSVDLDHLYHTYSRILNRLSDGLNELRDKIISDKVKLQSIISDEQRNSQQTKIESSRSNNDELDASSFIIVDGS-------------QNEPLSIDLRTLFSDADRIVQKLKAISYR----LHNAEILELLSNMRSLRFQIDCLCRNARQLEKTKENVAP---MEALQLENDRLQTVLIDAK-ILLQRAHE-RLSKLPNSK------------------------DMCEQILREMNEISKAMKATTREVHRVGSRSRGP--IGAKSSK--  
----------------------------------------MASGNIGRDNDMELNFEITD-QNGR-DATQDSLTEEAKF--LL--TTHKPTRIDIFQKERAL--SDHRSNTLTPSTVKRTRSVLSDATP---VSAAAFSKDTLSSPQHHTYLNIAETKEENKQLREEIFDSKSQLLLLKRKLLPILDSQGK---DFSEEYLMMLKGLDNEKIRQAELKQI---CQLRNKKLDEGKSQTLSERNDWEKERERLLMNMNELTS-------------------------------------------------------ELKKARQQLL--EKHGERDDCNESQGDSSLSTISLISERGMEI------ERLRSAVLQQNVDEKKFRMKMEQNIIQLQEQLKLAQDNLAKQN--INYTKEKTRCETLKTEMTKLQQVVTEARQEV-EYQKQISEEQLAKAHAINEI--ALN----KRDRTIRILLK----KLKSVDPQASSTSKIEASNLSSLRHDVPNLNPYAQAILHQINEFTVE---------NDAIRKKVIEWGAINIDLTAEIES-------------------SETASTEDESSEWNTKHCERENEKLAVFLAQN-KTIPDISNEEELREKLALFDAV-------------------SNSGRRCSKEELDDTPRNMRAS-----------EMLNI-TSTADLLSKSLTLTEDLGEIKKKLSVLQSSCTRLFEKLRGTANFLQTLLDELGSGDQGRELLAKIEALRIDLDHSLATAIDISRDVEAAEEGIGDLSAHLQQSLLNCSFGVSSVPYNENQ-DASALSRAQRAC---RQLNVDLANEKMKVNESTKLN-EKLQAHVADLEESKEKLIDELNELKKNMAEKKSEMISSVKDMEIQLKHKNQQYDELCRKVEELQQTVKAKEVFAAEREFEVKQLKDQMNKKCHELEVQVKEITNNLKVKDESI---------EKLLIDVESYNDQLSSRNNELAHYRNEIIALENKMCELIGTLRENLGIVQKQVDKPVVE--NMEIAALTQLSQIAADFE-ELTKVSNYIADLKSQMANLQQKLDLCERSRNAISLKCRGLSDQ--NAYLEAERNGD--------KQKLFTLQQXIQIEKESIKMMERDLQRLKSENSYVKENNERLTNMLVNMETKY----EELISKPNHSSLSERQHS-ERYTMTVSTMTVMSSHDFVELTDKAKTLASFTKRMYTLAARWNGETVVTTMISYKQADLDHLYHTYARILNCLSDGFSELRNRIVLGKVKLQSIISEEQQNSQQTKSGNSRSSNEELSTNCFIPVDGS-------------QKELLSVDLRTLFNEADQIIEKLSAISNK----LHNAEILEVLSNMRLLQFQIDYLHEHSRHFEKTKENFAP---MEALQLENDRLQTALTDAR-ILLQRAHE-RLNKLPNSK------------------------DMCEQILCEMNEISKVMKATTREVHRIGSHSRV-----VKSYK--  
LKLVFKCWCVNLKCEKSWEVGLFIVPYISTKEETEMDPEMMRMNTAADDVERNFVNEISN-RNKN-DTMQDSLNEEAIS--LL--NIRKPMQTDISEKELTS--LDYQSNTSTPNTVKNTRNVSSDATP---V-AAAFSEGALSLPHHRAYLNITETMEENKKLKAEVFDLKSQMFMLRRNLPSILDNEGK---DFIEEYLTIQTELDNEKVRRVEMEQI---WHSQKKEFDEERSKFESERYDWEKKCERLLMDRDELVA-------------------------------------------------------ELKELHQQLS--ERRGERDDGSESQGDSSLSTISLISERGMEI------ERLRSVVLQQSVDEKKYRMKMKHEIIQLQEQLKLAQDSFAKQT--AHYMKEKMRSESLKVEMTKLQEIADEAKQEV-EYQKQISEEQLAKAYAINEV--ALN----KRDRAIRILLK----KLKS-NAETSSSSKFEGNDSCLFHRDIPNLNPYAQGILDQINEFTVE---------NDVIRNKVIEWGVMNIDLTSEIEP-------------------SETPSVEDENSEWNTKRCERENEKLAAFLAQN-KTISDGSNEEEVGGKLALFDAI-------------------NSSGEKYSKELLNDTARNMGAS-----------EMLNI-TSTAELLSKSLVLTEDLGEVKKKLSVLQSSCARLFEKLRGTANFLQTLLDELGAGDRGKELLAEIEGLRIDLDHSLTTAIDISHDVEAAEESIGNLSAHLQRSLLNCSFGISSVASNENQQNASALSRAHRAY---RRLNADLANEKMKANEVIELN-EKLQAHITDLQDSKQKLVDELDDLKKKSIEKENEMISSMKDIETQLQHKNQQYDELYREVQELQQEIKAKEIFVATREAEMEQLTDQMAEKCRELENQVAEITCALKIKDESIKISCFLFNFKKLLIDVESYNNQLLSHNNELIHYRNEIIGVDNKMCQLISVVKESLGIVVEQVDKPIIETANAELGILARLSQIGADIE-EFTKVSHYIADLRNQMIKLQQDLDLCERNRSVINTKCHGLSKQ--NAYLEAERNEA--------KQKLLALQQQVQMEKESVKMIERDLQRLKSENSYVKENNERLTNMLVSVEAKY----EELISKSNCICFSERQCS-ERYTVTIGTMTVLSSHDFLELNSKAKTLSSFMKRMYALAARWNDKATVISKISYKSTDLDHLYHTYAQILRRLSDGLNELRNKITSDRVKLQSIISDEQQNPQKIGIENSRSDNDELDAPPFITVNGS-------------RNELVSIDMQALFSDADQIVQKLKIISNR----PHNAEVLEVLSNMRSLRFQIDWLRKYSHYFKKTKENVAP---MEALQLENERLQAVVVDAR-ILLQRAHE-RLSKLPNSR------------------------EMCDQILREMSEIGKAMKATTREVRHVRNRS--------------  
LKLVFKCWCVNLKCEKSWEVGLFIVPYISTKEETEMDPEMMRMNTAADDVERNFVNEISN-RNKN-DTMQDSLNEEAIS--LL--NIRKPMQTDISEKELTS--LDYQSNTSTPNTVKNTRNVSSDATP---V-AAAFSEGALSLPHHRAYLNITETMEENKKLKAEVFDLKSQMFMLRRNLPSILDNEGK---DFIEEYLTIQTELDNEKVRRVEMEQI---WHSQKKEFDEERSKFESERYDWEKKCERLLMDRDELVA-------------------------------------------------------ELKELHQQLS--ERRGERDDGSESQGDSSLSTISLISERGMEI------ERLRSVVLQQSVDEKKYRMKMKHEIIQLQEQLKLAQDSFAKQT--AHYMKEKMRSESLKVEMTKLQEIADEAKQEV-EYQKQISEEQLAKAYAINEV--ALN----KRDRAIRILLK----KLKS-NAETSSSSKFEGNDSCLFHRDIPNLNPYAQGILDQINEFTVE---------NDVIRNKVIEWGVMNIDLTSEIEP-------------------SETPSVEDENSEWNTKRCERENEKLAAFLAQN-KTISDGSNEEEVGGKLALFDAI-------------------NSSGEKYSKELLNDTARNMGAS-----------EMLNI-TSTAELLSKSLVLTEDLGEVKKKLSVLQSSCARLFEKLRGTANFLQTLLDELGAGDRGKELLAEIEGLRIDLDHSLTTAIDISHDVEAAEESIGNLSAHLQRSLLNCSFGISSVASNENQQNASALSRAHRAY---RRLNADLANEKMKANEVIELN-EKLQAHITDLQDSKQKLVDELDDLKKKSIEKENEMISSMKDIETQLQHKNQQYDELYREVQELQQEIKAKEIFVATREAEMEQLTDQMAEKCRELENQVAEITCALKIKDESIKISCFLFNFKKLLIDVESYNNQLLSHNNELIHYRNEIIGVDNKMCQLISVVKESLGIVVEQVDKPIIETANAELGILARLSQIGADIE-EFTKVSHYIADLRNQMIKLQQDLDLCERNRSVINTKCHGLSKQ--NAYLEAERNEA--------KQKLLALQQQVQMEKESVKMIERDLQRLKSENSYVKENNERLTNMLVSVEAKY----EELISKSNCICFSERQCS-ERYTVTIGTMTVLSSHDFLELNSKAKTLSSFMKRMYALAARWNDKATVIS--------------------KRLSDGLNELRNKITSDRVKLQSIISDEQQNPQKIGIENSRSDNDELDAPPFITVNGS-------------RNELVSIDMQALFSDADQIVQKLKIISNR----PHNAEVLEVLSNMRSLRFQIDWLRKYSHYFKKTKENVAP---MEALQLENERLQAVVVDAR-ILLQRAHE-RLSKLPNSR------------------------EMCDQILREMSEIGKAMKATTREVRHVRNRS--------------  
---------------------------------------------------------------------------------------------------------------------------------------------------------------------------------------------------------------------------------------------------------------------------------------------------------------------------------------------------------------------------------------------MTSGNIATDNDMELNFEITDQDGRDAT-----QDSLTEEAKFLLTTHKPTRADIFQKE-------RTPSDYRSNTLTPSTV---------KRTRSVLSDAT----PVSAAFSEGDL----------------------------------------------------------------------------------------------------------------------------------------------------------------------------------------------------------------------------------------------------------------------------------------------------------------------------------------------------------------------------------------------------------------------------------------------------------------------------------------------------------------------------------------------------------------------------------------------------------------------------------------------------------------------------------------------------------------------------------------------------------------------------------------------------------------------------------------------------------------------------------------------------------------------------------------------------------------------------------------------------------------------------------------------------------------  
----------------------------------------------------------------------------------------------------------------------------------------------------------------------------------------MLDSQGK---DFSEEHMLVLRGLDNEKIRQAELKQI---CQLRNKKLDEGKSQSVSERNDWEKERERLLMNMNELTS-------------------------------------------------------ELKKARQQLF--EKHGERDDCNESQGDSSLSTISLISERGMEI------ERLRSIVLQQNVDEKKLRMKMEQNIIQLQDQLKLAQDNLAKQN--INYAKEKTRCETLKTEMTKLQQVVTEARQEV-EYQKQISEEQLAKAHAINEF--ALN----KRDRTIRILLK----KLKSVDAQASSTSKVEASNLSSPRHDVLNLNPYAQAILDQINEFTVE---------NDAIRKKVIEWGAVNIDLT-EIES-------------------SETASTEDESSEWNTKHCERENEKLAAFLAQN-KTIPDISNEE-LREKLALFDAV-------------------NDSGRRCSKEKLDDTPRSMGAS-----------EMLNV-TSTADLLSKSLTLTEDLGEIKKKLSVLQSSCTRLFEKLRGTANFLQTLLDELGSGDQGRELLAQIEALRIDLDHSLATAIDISRDVEAAEEGIGDLSAHLQQSLLNCSFGVSSVPSNENQ-DASALSRAQRAC---RRLNAALANEKMKVNDSTKLN-EKLQAHVTDLEESKEKLIDELNELKKNMARKK-------------LQHKNQQYDELCRKVEELQQTVKAKEVFVVEKEFEMKQLKDQMNKKCHELEVQVKEITHNLKVKDESI---------EKLLIDVESYNDQLLSRNNELAHYRSEIIGLENKMCELIGTLRGNLGIVQKQVDKPVVEAENMEIAALAQLSQIAADFE-ELTKVSHYIADLKNEMANLQRDLDLCERSRNAISLKCRGLSDQ--NAYLEAERNGD--------KQKLFTLQQQVQIEKESVKMMERDLQRLKSENSYVKENNERLTNMLVNVETKY----EELISKPNRSSLSERQHS-ERYTVAVSTMTLMSSHDFVELTDKAKTLASFTKRMYTLAARWNGETVVTTMISYKPADLDHLYHTYARILNCLSDGFSELRNKIVSGKVKLQSIISEEQQNSQQTKSGNSRSSNDELGTNCFIPVDGS-------------QKELVSVDLRTLFNDADQIVQKLSAISNR----LHNAEILEVLSNVRLLQFQIDYLRGHSRHFEKTKENFVP---VIFFA------------------------------------------------------------------------------------------------------  
-------------------------------------------------------------MEDN-SVLNEDSNLEDVAGKFIDFSLFFNKIIDFS------AASAPRKSMSQPSLAGLGEP-------------KKGLSNAFSASNVRS-IPIIQTWHENEEMKGQIYTLRCEVQMHERKYLEAKGLCHRNVQDVMDEYVEMKIAQDDWEQKMRQFK-------QMEMDIDECSR----QKTEMEQRIRELEISLTEQQEQRATVTSDVNNTTCGSLRGTLDDIMKRNDPDFTLTSGYDE-KIAELEGKLMSEMDKVAELEDQVK--NLLEEVKDQSARLAQSENARAQLEEAASHGV-----SVMVPNSTFVIGNARQSQTEQQIKYIDELETKLTDAKSESEKAR--LALVEYMNKCSKLENEMHRIKKNQTFDTSSL-LIG-QTSEELKAQITKVNGELNSLR----AENRELRIRCH----QLNGETDDGNLSSSLLGHSRLMAGISSTDLA--DSN---ETGGTSMRIVPSS--GGASHLEAELEESKLPLMDTSAAVRS------------------KQAFDAAYDDFESLKDGLLNNDNNTLESS-FNSSMPPPDRDATQSFLSQKS-FKNSPLAA--PQRPKPLQQLMTSSGA-DQIQNHSFSTK-ATPSPHPSHLP---ILQDVQQILDSSAILLEGQHEAAANVEKMQAKMSQIRDALSRLFERLKSSAALFEDILE--------------------------------------------------------KSIVAESFIAGVSRRFTIAPDAEDVA-------SSSLLNAS--YSPIFKFS-N-NVMEVEKLQNEVAELRNELEKARVRGDMKSPLQNSS-GRLSDVQLKAAQNFEDLEVCQATLNK--------VEAEKAELMNELSRLEDSHRRLVAELDEAKRQM----------------ENTLVRCAAEQDARLNAEEILEDTKRSVEVLKR---EQTNAMMIEVDYRVTEAKELVEREKKAIIESLS--SELQTALA-EENAVRDHLERMEHEMELLRRRAREAEDRVERTANEKHEMVEH--IVSLEEK------------MEKAAAFEKESADYAGKLAARKKEIEVSKKREDMVNAAIEGLERVRKEV--------VELTKKTLKTQIILGNAS---------SIRLVCDELCRRLTRERELQHESAETMKYVNDNIEQLQKENLEMQSKIRESLGVSKKSSPSISNNKENAPPPVASSEA--PVTAP---PSSSQKTSTTASNFVSPTRQLLHESTMAVDS----------------------IVQKLKKTYTMSGMGAELKET---------IGNLIIESRGLR---DFLHQKLILFKGIDMTMWK---NDSVDQLVEKLAQYLQDNL-ILEEQIKKYKKE-LKLTKDVIP----------------NLGADVQERIKREIGGIASDM-GAVKAL---RNKK--------------  
-------------------------------------------------------------MEDN-SVLNEDSNLEDIA------------------------ASAPRKSMSQPSLAGVGEP-------------KKGLSNAFSASNVRS-IPIIQTWHENEEMKGQIYTLRCEVQMHERKYLEAKELCHRNVQDVMDEYVEMKIAQDDWEQKMRQFK-------QMEMDIDECSR----QKTEMEQRIRELELSLTEQQEQRATVTSDVNNTTCGSLRGTLDDIMKRNDPDFTLTSGYDE-KIAELEGKLMNEMDKVAELEEQVK--NLLEEVRDQSARLAQSENARAQLEEAASHGV-----SVMVPNSTFVIGNARQSQTEQQIKYIDELETKLTDAKSESEKAR--LALVEYMNKCSKLENEMHKIKKNQTFDTSSL-LIG-QTSEELKAQITKVNGELNSLR----AENRELRIRCH----QLNGETDDGNLSSSLLGHSRLMAGISSTDLV--DSI---ETGGTSMRIGPSS--GGAGHLEAELEESKLPLMDTSAAVRS------------------KQAFDAAYDDFESLKDALLNNDNNTLES-----------------------------------------------------------------------------------------------------------------------------------------------------------------------------------------------------------------------------------------------------------------------------------------------------------------------------------------------------------------------------------------------------------------------------------------------------------------------------------------------------------------------------------------------------------------------------------------------------ENAS---------SIRLVCDELCRRLTREREQQHESAQTMKYVNDNIEKLQKENLEMQAKIRESQGASKKSS--TSNNKENAPPRVASSEA--PITAP---PSSSQKTSTTASNFVSPTRQLLHESTMAVDS----------------------IVQKLKKTYTMSGMGAELKET---------IGNLIIESRGLR---DFLHQKLILFKGIDMTMWK---NDSVDQLVEKLAQYLQDNL-ILEEQIKKYKKE-LKLTKTVIP----------------NLGADVQERIKREIGGIASDM-GAVKAL---RNKK--------------  
-------------------------------------------------------------MEDN-SVLNEDSNLEHVE-------------------------GQPRRSMSQPVLNVEGDK-RTSSTS---ATQQQVLSGAFSSADVRS-IPIIQTWEENKALKTKITILRGELQMYQRRYSEAKEASQKRVKEVMDDYVDLKLGQENVQEKMEQYKLMEEDLLAMQSRIETSEDNFARQMKEFEAQKHAMEERIKELELS----ATDANNTTVGSFRGTLDDILKKNDPDFTLTSGYEERKINDLEAKLLSEIDKVAELEDHIQ--QLRQELDDQSARLADSENVRAQLEAATGQGILGAAGNAMVPNSTFMIGNGRESQTRDQLNYIDDLETKLADAKKENDKAR--QALVEYMNKCSKLEHEIRTMVKNSTFDSSSM-LLGGQTSDELKIQIGKVNGELNVLR----AENRELRIRCD----QLTGG--DGNLSIS-LGQSRLMAGIATNDVD--SIGQGNETGGTSMRILPR-----ESQLD-DLEESKLPLMDTSSAVRN------------------QQQFASMWEDFESVKDSLQNNHNDTLEGS-FNSSMPPPGRDATQSFLSQKS-FKNSPIVMQKPKSLHLHLKSHQSEGAGEQIQNNSFSTKTASPHVSQSHIP---ILHDMQQILDSSAMFLEGQHDVAVNVEQMQEKMSQIREALARLFERLKSSAALFEEILERMGSSD---PNADKIKKMKLAFETSINDKLNVSAILEAAEKDLHNMSLNF--SILEKSIVSQA--AEASRRFTIAPDAEDVA-------SSSLLNAS--YSPLFKFT-S-NSDIVEKLQNEVSELKNELEMARTR-DMRSPLNGSS-GRLSDVQINTNRMFEDLEVSEATLQK--------AKEENSTLKSQFAELEANLHQVNSKLGEVRCEL----------------NEALARVDGEQETRVKAENALEEARQLISSLKHE--------ENELKKTITDMGMRLNEAKKSD-EFLK--SELSTALE-EEKKSQNLADELSEELNGWRMRTKEAENKVEHASSEKSEMLER--IVHLETE------------MEKLSTSEIAADYCSTKMTERKKEIELAKYREDFENAAIVGLERISKEI--------SELTKKTLKAKIIPSNIS---------SIQLVCDELCRRLSREREQQHEYAKVMRDVNEKIEKLQLEKDALEHELKMM-----------SSNNENVPPVGTSVSG---------MPTKTSNQKCAQPHYTSPTRQLLHESTMAVDA----------------------IVQKLKKTHNMSGMGPELKET---------IGNVINESRVLR---DFLHQKLILFKGIDMSNWK---NETVDQLITDLGQLHQDNL-MLEEQIKKYKKE-LKLTKSAIP----------------TLGVEFQDRIKTEIGKIATDMGGAVKEI---RKK---------------  
-------------------------------------------------------------MEDN-SVLNEDSNIEDVVGR-----PTANLQQSGH------SSGNPRKSMSQPSLAIIGESGETSSTA---AARKPGMSGSFSNSNVRS-IPIIQTWHENEELKGQLYSLRCEVQMHERRYLEAKGVCHKKVQDVLDEYVEMKIAQEDYDEKMRDYKMLQKELQDMQIKLDNSESNFAGQVDQFDKQRKDMEQRIRDLEA---TTAPDPNNTTYGTLRGTLDDIMKKDDPDFTLTSGYEERKIAELENKLLSEMDKVAELEDQIK--SLLAEVEDQSARLAQSENIRIQLEAAASQVIP----SANVPNSTFVIGNARESQTEEQIKYIDELETKLTDAKNESEKAR--LALVEYMNRCSKLENEIHRLKKS-TFDSSSV-LIGGQSSEELKAQIEKVTGELKELR----NENRYLRIRCD----HLTGG--DGNLSMS-LGQSRLMAGISPSDLV--TSMQENETGGTSMRMLPR-----ESQLD-DLEENKLPLMDTSAAVRSVSSKFETRCKNKNDAIFQKEAFDAAYNDFESLKQGLT--------SS-FNASMPPPDRDATQSFLSHKS-FKNSPMMK--PKPLHMLLKSHQNETA-EQIQNHSFSTKTASLQASHCHMP---LLQDVQHILDSSAILLEGQHEAAANTEKMQAKMTQIRDALSRLFERLKSSAALFEDILEKMGSSS---PLADRIKQMKLAFETSISDHADVSGLLEAAEKDLHTMSVNF--SILEKSIITESFVADVSRRFTIAPHSEDVA-------SSSLLNAS--YSPIFKFS-S-NTIEVEKLQNEVAELRSELDKARAR-ELKSPLQGSP-GRLSDVQIKAAQNFEDLEVCQATLKK--------IESEKAELESEIAKHELTHHRLVNELEEIRREL----------------EKASIRVSTERKARLQAEEILEETKRSVQQLKK---EHENAMMIEVDYRVTEAREQVERESKQVIESLS--VELRTALA-EENAVRDHLEEMKDEMQKLKRRTKEAEDKVEFSANEKQEMMGH--IANLEEH------------LEKVSLQEKEFTDCDAKLTARKKEIELIKSRDDMELAAIEGLERVRKEI--------AELTKKTLKVPLIKGDSS---------SIQHVCDEICRRISRESQLQHESAETLKYVNDKIESLQKDNNELKASLKAA----------VVNN-ENVPP--VEANG--PKTAS---TSTKPKE---PSNFVSPTRQLLHESTMAVDA----------------------IVQRLKKTHNMSGMNSELKET---------IVNLINESRTLR---DYLHKKLILFKGIDMTKWE---NASVDKLVEKLGQYQQDNL-ILEEEIKKYKKE-LKLTKAVVPVSSNSTDDLTTIIFFQNLGADVQERIKREIGGIAKDM-GAVKEL---RKK---------------  
-------------------------------------------------------------MEDN-SVLLEDSNMEETLS--------------AN------PPLPPRKSISQPTLLPNDEAELTNS------AQKQGLSNNFPASNVRS-IPIIETWRQNEELKGQMYTLRTQVQMFERKYLEAKEASHKSVQEVMDEYVLLKIAQDDMEEKARQFADLENEIRALKEKNSLEKEHFFRLSAQFEQQKQVMEMRIRELEQV--TSTVDGNNTTFGSLRGTLDDIMKKNDPDYTLT-GYEDKKINDLENRLLNEMDKVSELEDLAR--ELRKELDDQSARLADSENHRAQLEAAAGQGILG----AAGASSTFVIGNARESQTAEQIRYIDELETKLTAANNDSEKAR--LALVEYMNRCSKLENDIYTMRKNTTFDTSSM-LIEGKTSEELKAQIDKVNGELNSLR----AENRELRIRCD----QLTGG--EGNLSTS-LGQSRLMAGISATELA---SRAENETGGTSMQILRRDSHSDENFSDSHIQDSKLPLMDTTAALKN------------------QEEFDAAWQEFEAEKNRLKFMTNDTADCSDFDMSMPPPGANATQSFLIQKG-FKNSPVA----------LSVKKTQQSGEQIQNNSFSTKDNTSLVGSAAAVGPTLLQDVQQILDSSQVLLDGQHDAAVNVERMQEKMSQIREALARLFERLKSSAALFEDILEKMGSSS---PLADRIKQMKLAFETSMCDHADVSVFLEAAEKDLTNMSLNF--SVLEKSIVGQSFVADVSRRFTIAPGADEIA-------SSSLLNASS-YSPIFKFQGTGRLAEIEKLQKEVADLKNELELARVR-ELRSPLQASP-GRLYDVQIKAAQNFEELEVCQATLRK--------AEAEKAVLAQELAELETAHRHLTAQLSDVREEL----------------HRASESVEAERNHREKAETALFESEQMVQVLQSGTDEKYKEIMLDVERRHAEVR----EHHKTIIEGLQ--AELKTSLD-EENSIRDYLEEIKVENGRLRQRTHEAELTIEKQASEKQTLVTQ--LVALENK------------LEHLEVFEQKATNYGKKLEEKKVELAEAKYRFEVEKESISGLERIQKQM--------AELTKKNAKAQIVLGEAS---------SIRSVCDEICRRISQECNRQHEYAQTLSAVNDKIEQLTIEK-------------------------ENASPSPSPYQISVKTPS-----SSSAPKPPSSSKFLCPTRQLLHEATLAVDA----------------------IVQRLKKTHTMPGMGSELKET---------VVKLLTDSRALR---DFLHQNLNLFKGIDVNNWK---NETVEQLLERLEQCYQDNL-ILEEENRKFKKE-LKQMKAAIP----------------NLGADVQERIKREIGGIAKDM-GAVKAL---RKK---------------  
-------------------------------------------------------------MEDN-SVLNEDSSIDEAA-------------------------APPRKSMSQPSLAVIGEPSTPPSNS---TPPKQSLSR--STSNVRS-IPIIQTWHQNEELKGQIYSLRCEVQMHERKYLEAKEACHKSVSDVLDEYVMLKLEQEKYESMTSQVGSLERELEETRAKLENSEYNFAKQAEEFEREKLLMEQRIRELEAT--TPVNDPNNTTYGSLRGTLDDIIKKNDPDFTLTSGYEEKKIRELEEKLLREQDKVAELENHIK--DLQDEIEDQSARLVQSENLRIQLETASGQGV------LPVPNSTFIIGNARESQTEQHLKYIDELETKLTDAQNESEKAR--LALVEYMNRCSKLENENHKLKKNSEFDSSSI-LIGGKTSEELKAQIDKVNGQLNTLR----AENRELRIRCD----QLTGG--DGNLSTS-LGQTRLMAGISSTDLASASGAQENETGNTSIRMIPR-----ESEFD-VLDESKLPLMDTSAAVRN------------------KTEFNTAYDEFESLKSQLQSNAHDTLESS-FNGSMLPPDKDATQSFLSQKGGYKNSPLVVQKPKQLQQLLDIHQEESA-DQIQNNSFSTKNASPRSYNN--P---ILQDMQHILDSSAILLEGQHDAAANVEKMQEKMTKIREALSRLFERLKSSAALFEDILEKMGSSS---PLAERIKQMKLAFETSINDHADVSVILEAAEKDLNNMSLNF--SILEKSILTQSFVADVSRRFTIAPDSEDVA-------SSSLLNAS--YSPVFKFPGK-SVAEIEKLQQEVSELKSELEKARTR-DLRSPLQNTSQGRLSDVQIKANQNFEELEVCQATLKR--------VETEKAALAQEHERLQATHHQILNQLEEIRAEL----------------DNALSRVEQEADARCYAEEALHEAKKTVNSLQQKFESHAHDVNSDLETRVAEACIKIEEEHRIVIESLK--QELATSLA-EETAIREQMDEFRGEIERLRRRTREAEDKIEQGANEKQTLVEQ--IIALEDQ------------LDKASLFELQATDCASKMAAKKKELETAQRHEEIEKEAVEGLERIQKEV--------IELTKKTLKAQIIKGNAS---------SIRVVCNEMCHRITREREQQHEASETMKVVNANIEEMVKENAELKKELNQL---------ATTTNNENAPP--AEQKQ--PESSQNLTPGSSSKQ--KSSTFVSPTRALLHESTMAVDG----------------------IVQRLKKTHSMSGMSAELKNA---------IASIIMESRAVR---DFLHQKLILFKGIDMTKWK---NESVDQLVEKLGQYHQDNL-MLEEQIKKYKLE-LKQTKAVIP----------------SLGLDVEERMKREIGGIAKDM-GAVKAL---LNKKK-------------  
-------------------------------------------------------------MEDN-SVLNEDSSIDEAA-------------------------APPRKSMSQPSLAVIGEPSTPPSNS---TPPKQSLSR--STSNVRS-IPIIQTWHQNEELKGQIYSLRCEVQMHERKYLEAKEACHKSVSDVLDEYVMLKIEQEKYESMTSQVGSLEKELEETRAKLDNSEYNFAKQAEEFEREKLLMEQRIRELEAT--TPVNDPNNTTYGSLRGTLDDIIKKNDPDFTLTSGYEEKKIRELEEKLLREQDKVAELENHIK--DLQDEIEDQSARLVQSENLRIQLETASGQGV------PPVPNSTFIIGNARESQTEQNLKYIDELETKLTEAQNESEKAR--LALVEYMNRCSKLENENHKLKKNSEFDSSSI-LIGGKTSEELKAQIDKVNGQLNTLR----AENRELRIRCD----QLTGG--DGNLSTS-LGQTRLMAGISSTDLASASGAQENETGNTSIRMIPR-----ESEFD-VLEESKLPLMDTSAAVRN------------------KTEFNTAYDEFESLKSQLQSNAHDTLESS-FNGSMLPPDKDATQSFLSQKGGYKNSPLFVQKPKQLQQLLDIHQEESA-DQIQNNSFSTKNASPRSYNN--P---ILQDMQHILDSSAILLEGQHDAAANVEKMQEKMTKIREALSRLFERLKSSAALFEDILEKMGSSS---PLAERIKQMKLAFETSINDHADVSVILEAAEKDLNNMSLNF--SILEKSILTQSFVADVSRRFTIAPDAEDVA-------SSSLLNAS--YSPVFKFPGK-SVAEIEKLQQEVSELKSELEKARTR-DLRSPLQNTSQGRLSDVQIKANQNFEELEVCQATLKR--------VETEKAALAQEHERLQATHHQILNQLEEIRAEL----------------DNALSRVEQEADARCYAEEALHEAKKTVNSLQQKFESHAHDVKSDLETRVAEACIKIEEEHRIVIESLK--QELATSLA-EETAIREQMDEFRGEMERLRRRTREAEDKVEQGANEKQTLVEQ--IIALEDQ------------LDKASLFELQATDCASKMAAKKKELETAQRQEEIEKEAVEGLERIQKEV--------IELTKKTLKAHIIKGNAS---------SIRVVCNEMCHRITREREQQHEASETMKVVNANIEEMVKENAELKKELKQL---------AKT-NNENAPPTYSEQQQQPPESSQNLTPGSSSKQ--KSSNFVSPTRALLHESTMAVDG----------------------IVQRLKKTHSMSGMSAELKNA---------IASIIMEARAVR---DFLHQKLILFKGIDMTKWK---NESVDQLVEKLGQYHQDNL-MLEEQIKKYKLE-LKQTKAVIP----------------SLGLDVEERMKREIGGIAKDM-GAVKAL---LKKK--------------  
----------------------------------------MSEFNWKDEGTLDMTSDVSSIASECPDDQDSNASSSTIKNRTLESILDSPYAPTLDSTPVLVVKESGRLSISQPVLCSDRTPMDSQPTN----YSTSALNSTGHRREHTQKVPLMATINENKALKEEIVILKSKLSCEAYKVNELKALQSRDVQEILNEYFHLQMTKSDVEEERRMYG-----------------ETIERMQKDNESKERLMNVKVKDLET-------------------ICTNLVQEK---------------KALESNILSLTRRVNELEEDLY--SARNHETPSCLEAVKGEDSDWTIRSESD-------------ESTFVIGSSGEAGAVDYLKRIDELEMQLQTTEEQRIKSS--SALVAFMSRCRDMEKQL----QLANISTNNV-SIKMQNREEMLAVVEKIRMDLLGLR----KKNTVLREECA------------------------RIIRDEKQDLSSTESNIKDSLMAETFER--------SLLLKEDYEKKQLEIMANLDKIVG------------------------DLVDDGLFNQSVLDILKEVSEST--------SNTDNLSSFSAFQG-------------------------------------------------------------------IHIEGKDEQGDN-HAIIDNSSIIHKEAADQLSFLGSGSEDSKKVTD-------------HIRNMHLEWAHVFNETGGIINAINDAERSVVDLHGHL--SMLEQSLNQSSFIFEASSRFSLENVATKKD---------------------AGIDIDEVKKQLEKKIAENTELNNLIEETTKARDIALSTLDNIKKQMKAEELIVNILRENAIKSEQELDK--------REKTVKQLEIDLSQAVDAKGELTVKVSELQQAV------------------ISMRDEMKEDAERRRQEMILRMQRQYCLVLY-IYAYLLIHHIALQRTAAALSSELDRANKKAHQNALFTEDLKQCMEREKKHLIEEVQIAKANEERLSIRLSELESEHDGLRYEPKKKTAED-IVDLSLTKQ----------SRELPSRSCQTELTIRSLNAFEALNEKQLKELSCLQTAHECLVTTIMN-----------FMDKPHKSALVYGGVD---------TVETNCSEIIRLLSLEKRHREKLADEVANLRERLEKIMSNR-----------------------IKQAEVLVAERQESEQIIFEKRAVNDQKYEHPLRQGFYLMTSKAADLVNALRKVSS----------------------VQATGHKYCLRLFAILIKVS---------LSEVIDMARELR---NELNDRFAVLKVDKENTGS---EKTVEELIVKMRLIEKENS-TMYNALKMWKSK-FEELQRQSL-----------------NNPDLVDRIARELKDIQSVMGDTKRAAQTLINEKSLPGSKGKKSVKKL  
------------------------------------------------------------------------------------------------------------------------------------------------------------------------------------------------------------------------------------------------------------------------------------------------------------------------------MDETSFCS----GISTARTYESIPITAQNRIQHLESENYNL------------NAQLAQVMDMKDVENVDKIRTLSVKLNRQNEEIESLR--KQLEE---KEEHYRKTKRLKDKEITALRTDLVRLRDE-NAELKQEKMEMEAA---------KSDDESRIYRN------------------LLNHSNSARGPGVNEQVHPAPPQERFQSGSQ----------------------KIPFISNSQP---------------------KSAVSDYRNENPDFFRKQCLDMDSVCSESVASESRVHLSIDAELSMVNGSG-----------------------------------------------------------------FREILNLTNAAQSNVRNYKKVLHTWKSNIHILFDQVRSTANFLGEMATQMGRDSEHKETIERILAKINGMNFTLNASLDQTREIMEGAKGMEESLAEL-----------SNVIDDSLRRSMSILEGQTIA----------------------------QNEREIDLNKDVVDAHNELAAQREK-------TQRLEKELQEERAKIEELYRELEQKQDAYAN-------EIQAKGTDYKKNVELLETSLVRNRNEAEEGK--------------------EALIDLSELRAALEASQNEVHKQRMQNDKLNR----QVEELQVELQECVEKLDNALTAYDKVFDECGS---------------RGQMINELQVELEKANEAMKEVDELKMK--------------------------------LQQLESQNRALQQVKHESPEALQQLALVQKQLDVANEKVRKYNEWVIKI-----------------AEKVPEDMG----------IRPAADGIADIVGKIKGYIEKSVKDKKDLDELHETMKKGNQILRKKIELG------------AARHGVELDLGQMSP----------VRTDPLKIDSSKEFFNYFDGLVFMAKV--------------------------LVKKLKSEPEGPG-----------------QQQLLEDARQLR---------LALMSGEEFFKKN---KENLKLRLQGMGPDIRDHLGHIHQVLTQVRDN-VRKTR---------------------------------------------------------------------  
---------------------------------------------------------------------------------------------------------------------------------------------------------MAET--EHNVTVGSMAAVN-----LANRTSNLVEACVR----MFDK---LRGSAEFFQNLLKSLG-------VTAEELGED------IVGKIEAMKLDMDNSVGEAQT-------------------------------------------------ILT---EVKDAEKNWR--SILHDQSQAAVSFNSSGNTSSRNESKHEQQLS-----GNPLEEVQTPPTPLTAKCVLKTDENAELKMMLDNERTAKEELEGRVGVFEKLAAQTRAENDQLLLKIQVFLHFEKI-FFKNFEWLGFLYFDFIVSVRLQELKLKPCEKCAVLRDLYE-----TTKAALDDELATEVELRQRVEEQLQSIKVLQDELERISSSSKENML---------SAELKKTLVEHSRTELNQIFDTMS-----------------------FAKSRCSSVVGGSQAGSKKGRDGK----------KQQSKQIDQKK-----------------------------------------------------------------------------------------------------------------------------------------------------------------------------------------------------------------------------------------------------------------------------------------------------------------------------------------------------------------------------------------------------------------------------------------------------------------------------------------------------------------------------------------------------------------------------------------------------------------------------------------------------------------------------------------------------------------------------------------------------------------------------------------------------------------------------------------------------------------------------------------------------------------------  
------------------------------------------------------------MSDVN-DVMDASAVDQDLSQGSLNENVKELLERRFQEKAARTPVTAFSDGALSSAVGRTSSIVSAQWTPHATLSTPLLPQSSNSSTNRRMYVDIKEKLEENKILKAENFELKKKLFIIMRELPTMKDPKGN---DFTEDYLECRDMLYKEQTRRMEAEDE---LSSVKSEIAELKKKHEQEADEWVARNKRLLEDRNRLSED------------YSRQR-------------------------RELCTKAL----QVNRLTAQVESLSRQMMADDGNESQADTSLSNVSIMSSRDRII------EELKGTIVEMTVKEEQ-RAVMMKRMDTLNSDLARTASELEEQQ--KALREERQLNADATQKLVKLQHELDEAKNSASALKQEMTKREERYEAELAKVRKCIE----TRDRAINALMNKMPYKLTLSNFPDDMPQEVETHR-CVASDAAGDDNPVVQKLLERIGNISTT---------NNELRDQVERLKKEG-AFDKDFDP-------------------LEDPSTDDESSVWDLHRLQTENKKATAVLAREGMTDIDWSCQQMLRMKMREFDRT-------------------SSDAPLVPKIATTNRDSSLRSSSQAQL------EFPEM-SAMADLYSQSVTALDDLNTLRKRVSLLRRISLRLFEKLRGSAAFLQSLLDELGYSEKGRAFINEIEAMRIEFSRSATTATEILNGVSVAEQSISEFRAQVERS-MNFSMSLSSTRIDVTTAAAKVVSTSSSTVGTDRSSKYVLVEAQHGTSSQAEAELFVMKAKVIELERGRLQLEQELKAAAKQKAEYEKK-ITELQEVN---AEKCERELALSGQTQALTKKIEELQARSAENEFELRRVADEAEIRYRDLEVKKSELELLLEGSQQVV---------KEKAAELGKVNDSLRTREEEFSRYKRECALLEMKTRELIDTVKESIEAMPAKVVKDIATSSREIIDTRSSIAHEQLS-A-QIALMDVHMTELKTELERVRTVLEECEAGRKTAEDKCRILTDELKSQQVEMTRYKRGFEAAAQRREHVKVVGTQSEMSMDSINALEADFKKLQIANATTTENLRAMCETVVELESKARNVGQNLKPKTIDGCTSTTMAPLNLHKVNAAMMTSMGGMDIKEMEEKLNGYSFFAGKIYEFLGRWSKKEHSLSKTSFSSGDLERMYQKMVRMQKITDDEIISLKTQMEANKERICDFAKDRRKRSSSSSQRSSPGRRKLAASDGAMTSQSSSPASLEAEQLEVIRSELSAGDVNEMYRSSHSVVELIKDMLTSNAAAFKEEEVTEALQKARTIRSQLAALCTRLNQFEKAKENVDPNDSMEALKAENVKLQLALNDAKAMLMSSH-E-KLRIQPNSE------------------------AMCEAIVRELGKISKAMKSTTRDVCRYRRMRRGG--AGARTESS-
